# New Partners in Regulation of Gene Expression: The Enhancer of Trithorax and Polycomb Corto Interacts with Methylated Ribosomal Protein L12 *Via* Its Chromodomain

**DOI:** 10.1371/journal.pgen.1003006

**Published:** 2012-10-11

**Authors:** Anne Coléno-Costes, Suk Min Jang, Augustin de Vanssay, Julien Rougeot, Tahar Bouceba, Neel B. Randsholt, Jean-Michel Gibert, Stéphane Le Crom, Emmanuèle Mouchel-Vielh, Sébastien Bloyer, Frédérique Peronnet

**Affiliations:** 1 Université Pierre et Marie Curie-Paris 6, UMR7622, Laboratoire de Biologie du Développement, Equipe Chromatine et Développement, Paris, France; 2 Centre National de la Recherche Scientifique, UMR7622, Laboratoire de Biologie du Développement, Equipe Chromatine et Développement, Paris, France; 3 Institut Pasteur, Département de Biologie du Développement, Unité de Régulation Epigénétique, Paris, France; 4 Centre National de la Recherche Scientifique, URA2578, Paris, France; 5 INSERM Avenir, Paris, France; 6 Université Pierre et Marie Curie-Paris 6, UMR7622, Laboratoire de Biologie du Développement, Equipe Répression Épigénétique et Éléments Transposables, Paris, France; 7 Centre National de la Recherche Scientifique, UMR7622, Laboratoire de Biologie du Développement, Equipe Répression Épigénétique et Éléments Transposables, Paris, France; 8 Plateforme d'Ingénierie des Protéines, Service d'Interaction des Biomolécules, IFR83, Université Pierre et Marie Curie-Paris 6, UMR7622, Paris, France; 9 École Normale Supérieure, Institut de Biologie de l'ENS, IBENS, Plateforme Génomique, Paris, France; 10 INSERM, U1024, Paris, France; 11 CNRS, UMR 8197, Paris, France; 12 Université Pierre et Marie Curie-Paris 6, UMR7622, Laboratoire de Biologie du Développement, Equipe Analyse des Données à Haut Débit en Génomique Fonctionnelle, Paris, France; 13 Centre National de la Recherche Scientifique, UMR7622, Laboratoire de Biologie du Développement, Equipe Analyse des Données à Haut Débit en Génomique Fonctionnelle, Paris, France; University of Birmingham, United Kingdom

## Abstract

Chromodomains are found in many regulators of chromatin structure, and most of them recognize methylated lysines on histones. Here, we investigate the role of the *Drosophila melanogaster* protein Corto's chromodomain. The Enhancer of Trithorax and Polycomb Corto is involved in both silencing and activation of gene expression. Over-expression of the Corto chromodomain (CortoCD) in transgenic flies shows that it is a chromatin-targeting module, critical for Corto function. Unexpectedly, mass spectrometry analysis reveals that polypeptides pulled down by CortoCD from nuclear extracts correspond to ribosomal proteins. Furthermore, real-time interaction analyses demonstrate that CortoCD binds with high affinity RPL12 tri-methylated on lysine 3. Corto and RPL12 co-localize with active epigenetic marks on polytene chromosomes, suggesting that both are involved in fine-tuning transcription of genes in open chromatin. RNA–seq based transcriptomes of wing imaginal discs over-expressing either CortoCD or RPL12 reveal that both factors deregulate large sets of common genes, which are enriched in heat-response and ribosomal protein genes, suggesting that they could be implicated in dynamic coordination of ribosome biogenesis. Chromatin immunoprecipitation experiments show that Corto and RPL12 bind *hsp70* and are similarly recruited on gene body after heat shock. Hence, Corto and RPL12 could be involved together in regulation of gene transcription. We discuss whether pseudo-ribosomal complexes composed of various ribosomal proteins might participate in regulation of gene expression in connection with chromatin regulators.

## Author Summary

Chromatin, the combination of DNA and histones, strongly impacts transcriptional regulation of genes. This is achieved thanks to various protein complexes that bind chromatin and remodel its structure. These complexes bind specific motifs, also called epigenetic marks, through specific protein domains. Among these domains, chromodomains are well known to bind methylated histones. Investigating the chromodomain of the *Drosophila melanogaster* chromatin factor Corto, we found that it interacts with methylated ribosomal protein L12 rather than with methylated histones. This is the first time that such an interaction is shown. Moreover, Corto and RPL12 co-localize with active epigenetic marks on polytene chromosomes, suggesting that both are involved in fine-tuning transcription of genes. Our results represent a major breakthrough in the understanding of mechanisms by which ribosomal proteins achieve extra-ribosomal functions such as transcriptional regulation. Genome-wide analysis of larval tissue transcripts reveals that Corto and RPL12 deregulate large sets of common genes, which are enriched in ribosomal protein genes, suggesting that both proteins are implicated in dynamic coordination of ribosome biogenesis.

## Introduction

Chromatin structure strongly impacts on regulation of gene expression. Indeed, post-translational histone modifications (methylations, acetylations, phosphorylations *etc…*) called epigenetic marks, are recognized by protein complexes that shape chromatin (reviewed in [1]). A number of protein domains specifically interact with these modifications, thus inducing recruitment of chromatin remodeling or transcriptional complexes. Bromodomains recognize acetylated histones (reviewed in [2]) whereas 14-3-3 domains recognize phosphorylated histones (reviewed in [3]). Methylated histones are recognized by chromodomains (chromatin organization modifier) [4], which therefore belong to the Royal family of domains, known for their methylated lysine or arginine binding activity (reviewed in [5]). Chromodomains share a common structure encompassing a folded three-stranded anti-parallel ß-sheet supported by an α-helix that runs across the sheet. This structure contains two to four well-conserved aromatic residues that form a cage around the methylated ligand [5], [6].

Chromodomains were first identified in Polycomb (PC) and Heterochromatin Protein 1 (HP1) [4]. They are found in many other chromatin-associated proteins that belong to three classes according to their global structure: (1) PC/CBX family proteins harbor a single N-terminal chromodomain, (2) HP1 family proteins have an N-terminal chromodomain followed by a region termed a chromoshadow domain, and (3) CHD (Chromodomain/Helicase/DNA-binding domain) family proteins present two tandem chromodomains (reviewed in [5]). Most chromodomains specifically recognize particular methylated residues on histones. For instance, the chromodomain of PC, which is a subunit of the PRC1 complex (Polycomb Responsive Complex 1), binds specifically H3K27me3 [7], [8]. Once recruited, PRC1 prevents RNA Polymerase II recruitment or transcriptional elongation and therefore mediates gene silencing (reviewed in [9]). The chromodomain of HP1 binds H3K9me2 and H3K9me3, which are epigenetic marks characteristic of heterochromatin, and thus participates in heterochromatin shaping [10], [11]. Very few cases of non-histone chromodomain substrates are known [12]. For example, the HP1 chromodomain also recognizes an autocatalytically methylated residue of the G9a histone H3 methyl-transferase [13].

The *D. melanogaster corto* gene encodes an Enhancer of Trithorax and Polycomb (ETP), *i.e.* a Polycomb (PcG) and Trithorax (TrxG) complex co-factor, involved in both silencing and activation of gene expression [14], [15]. Indeed, Corto participates in transcriptional regulation of several homeotic genes together with these complexes and other ETPs [16], [17]. Corto binds chromatin and contains in its N-terminal part a single structured domain identified by hydrophobic cluster analysis and structural comparison as a chromodomain [18]. Hence, Corto would be closer to CBX proteins of the PcG class [5]. However, its chromodomain is rather divergent, since only two aromatic residues are conserved among the four that make a cage around the methylated residue. How Corto anchors to chromatin and more specifically, whether the chromodomain addresses Corto to chromatin, is not known. Here, we address this question by expressing a tagged Corto chromodomain in flies or in S2 cells. We show that the Corto chromodomain is a functional chromatin-targeting module. Surprisingly, peptide pull-down, mass spectrometry and Biacore show that the Corto chromodomain interacts with nuclear ribosomal proteins, and notably binds with high affinity RPL12 tri-methylated on lysine 3 (RPL12K3me3). Co-localization of Corto and RPL12 with active transcriptional epigenetic marks on polytene chromosomes suggests that both proteins are involved in fine-tuning transcription of genes located in open chromatin. Investigation of Corto and RPL12 transcriptional targets by RNA-seq reveals that many are shared by both factors. Analysis of *hsp70* occupancy by chromatin immunoprecipitation suggests that Corto and RPL12 cooperate in transcriptional regulation. Interestingly, the potential common targets of Corto and RPL12 are enriched in genes involved in heat response and ribosomal biogenesis.

## Results

### The Corto chromodomain genetically mimics full-length Corto function

**Figure 1:**
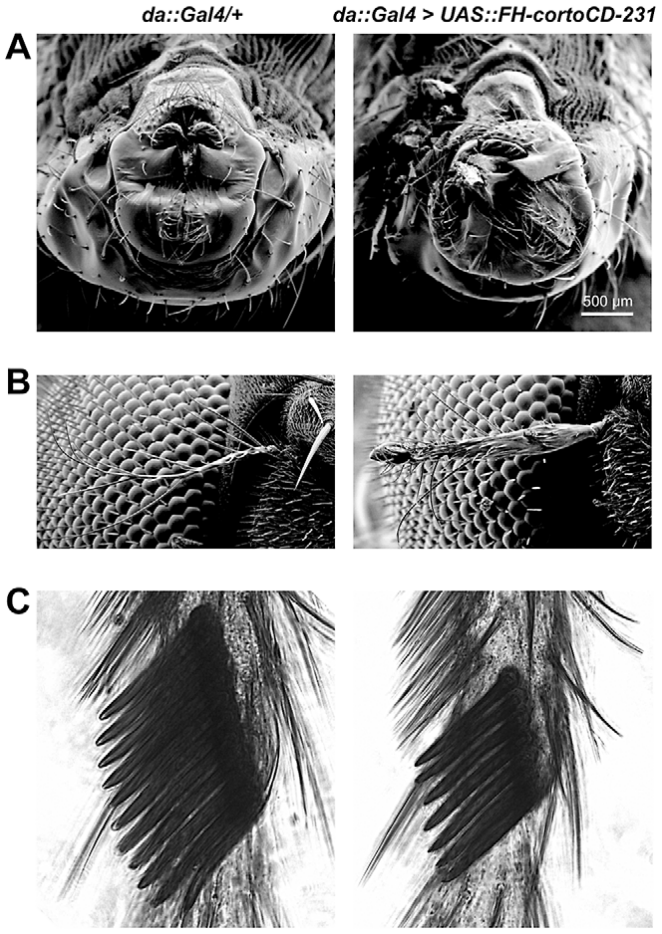
Phenotype of transgenic flies over-expressing *cortoCD*. Scanning electron microscopy images of male genitalia (A), aristae (B) and male sex combs (C). On the left, control *da::Gal4/+* flies. On the right, *da::Gal4>UAS::FH-cortoCD-231* flies. Males over-expressing *FH-cortoCD* present rotated genitalia and sex combs with a reduced number of teeth. Aristae of flies over-expressing *FH-cortoCD* are partially transformed into leg, a phenotype called *aristapedia*. Strengths of these phenotypes are shown in Table S1.

To address the role of the Corto chromodomain *in vivo*, we used germline transformation and the binary *UAS/Gal4* system to produce transgenic flies. These lines expressed either FLAG and HA double-tagged *cortoCD* fused to a nuclear localization signal coding sequence to force its entry into nuclei (*FH-cortoCD*), FLAG and HA double-tagged *corto* deleted of the chromodomain sequence (*FH-cortoΔCD*), or *corto* full-length (both FH-CortoΔCD and Corto full-length spontaneously enter the nucleus although no nuclear localization signal was detected, data not shown). Whereas transgenic flies ubiquitously over-expressing *cortoΔCD* [using either *Actin5C* (*Act::Gal4>UAS::FH-cortoΔCD*) or *daughterless* (*da::Gal4>UAS::FH-cortoΔCD*) drivers] were perfectly viable and had no visible phenotype, over-expression of *corto* using the same drivers was 100% lethal. Over-expression of *cortoCD* using again these drivers also induced high lethality at all developmental stages (from 63% to 100% depending on the transgenic line and the driver, Table S1). Escaper flies displayed rotated genitalia and duplicated macrochaetae as well as very penetrant homeotic phenotypes (Figure 1). Many flies presented a partial transformation of arista into leg, a homeotic phenotype called *Aristapedia* that could reflect down-regulation of the *spineless-aristapedia* gene [19]. Similar phenotypes were observed when over-expressing full-length *corto* using the weaker ubiquitous driver *armadillo* (*arm::Gal4*) (Table S1). Males over-expressing *cortoCD* also displayed smaller sex combs, a phenotype opposed to that of *corto* mutant males who have ectopic sex combs [15], [20], and which could reflect reduced expression of the homeotic gene *Sex combs reduced* (*Scr*) [21]. Taken together, these results suggest that the chromodomain is critical for Corto function.

### The Corto chromodomain is a chromatin-targeting module

**Figure 2:**
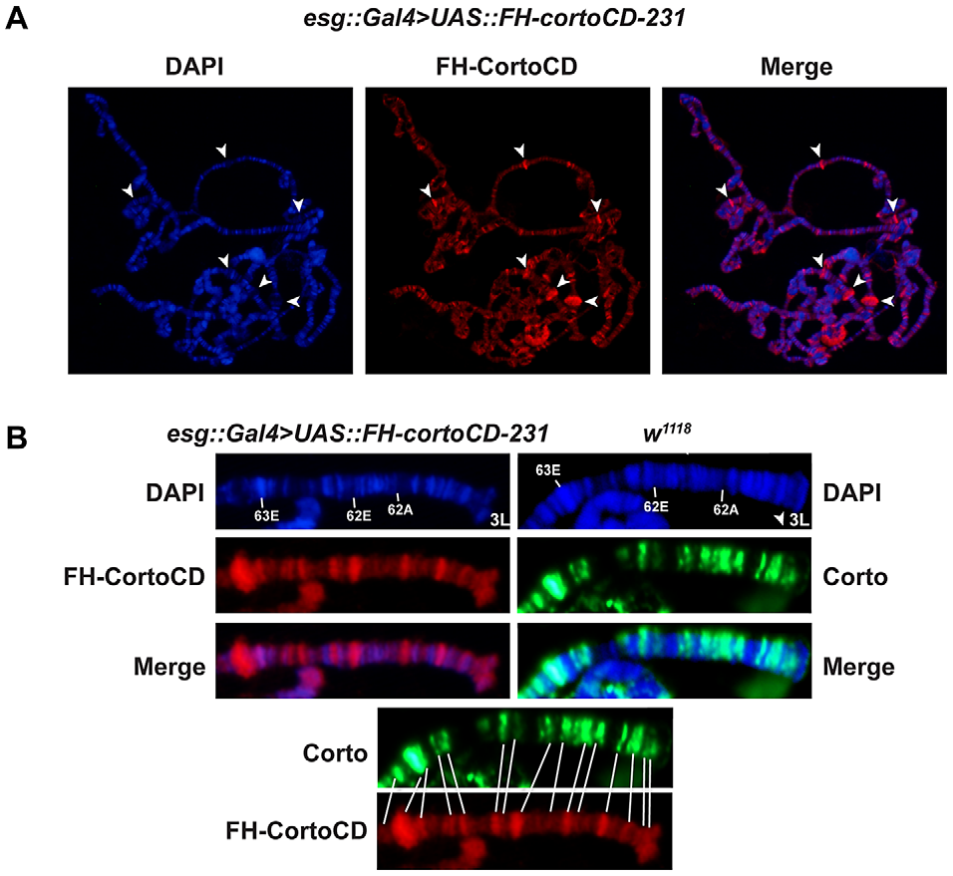
The Corto chromodomain is a chromatin-targeting module. (A) Squash of salivary gland polytene chromosomes from an *esg::Gal4>UAS::FH-cortoCD-231* third instar larva. Anti-FLAG immunostaining shows that FH-CortoCD binds chromatin at many sites (left: DAPI, middle: anti-FLAG, right: merge). Arrow-heads point to DAPI-free Corto bands. (B) Magnifications of the tip of polytene chromosome 3L (bands 61 to 63) from third instar larvae, either *esg::Gal4>UAS::FH-cortoCD* immunostained with anti-FLAG antibody or *w^1118^* immunostained with anti-Corto antibody (DAPI, immunostaining, merge). Bottom: conformity between endogenous Corto and FH-CortoCD binding sites.

Corto binds polytene chromosomes of third instar larva salivary glands at many sites [18]. To test the role of Corto chromodomain in chromatin binding, we immunostained polytene chromosomes of larvae over-expressing *cortoCD* in salivary glands [*escargot* Gal4 driver, (*esg::Gal4>UAS::FH-cortoCD*)] with anti-FLAG antibodies. FH-CortoCD bound polytene chromosomes at many discrete sites (Figure 2A). Like endogenous Corto, FH-CortoCD preferentially bound DAPI interbands and puffs, *i.e.* regions corresponding to open or actively transcribed chromatin. Comparison of endogenous Corto binding in wild-type larvae and FH-CortoCD binding in *esg::Gal4>UAS::FH-cortoCD* larvae at the tip of chromosome 3L showed that these proteins shared most of their binding sites (Figure 2B).

These results indicate that FH-CortoCD mimics Corto binding on polytene chromosomes and that the Corto chromodomain is a genuine chromatin-addressing module.

### The Corto chromodomain interacts with Ribosomal Protein L12

**Figure 3:**
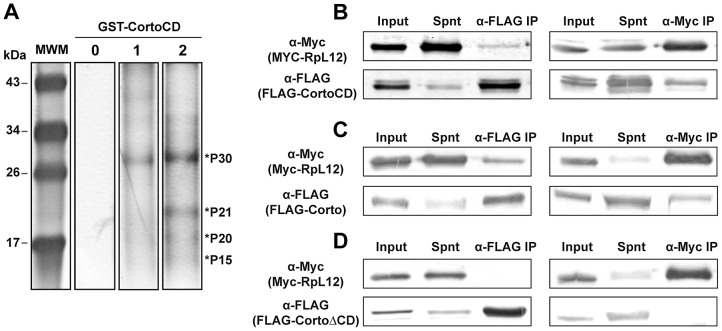
Corto interacts with nuclear ribosomal proteins and co-immunoprecipitates with RPL12 *via* its chromodomain. (A) Silver stained polyacrylamide gel showing polypeptides pulled-down by GST-CortoCD covalently linked on agarose beads from nuclear or cytoplasmic embryonic extracts. Four bands consistently appearing after incubation are enriched in nuclear extracts are shown by asterisks (P30, P21, P20 and P15). Line 0: no extract, 1: cytoplasmic extract, 2: nuclear extract. (B, C, D) Co-immunoprecipitation experiments. S2 cells were co-transfected with plasmids expressing FLAG-CortoCD, FLAG-Corto or FLAG-CortoΔCD and Myc-RPL12. Immunoprecipitations were performed with either anti-FLAG (α-FLAG) or anti-Myc (α-Myc) and revealed by Western blot with the same antibodies. Spnt: supernatant, IP: immunoprecipitation. (B) FLAG-CortoCD co-immunoprecipitated with Myc-RPL12 and conversely. Extracts were run on a 15% acrylamide gel. (C) FLAG-Corto co-immunoprecipitated with Myc-RPL12 and conversely. Extracts were run on a 12% acrylamide gel. (D) FLAG-CortoΔCD did not co-immunoprecipitate with Myc-RPL12 and conversely. Extracts were run on a 12% acrylamide gel.

These results prompted us to identify the anchor(s) of Corto chromodomain on chromatin. We incubated GST-CortoCD covalently bound on agarose beads with nuclear or cytoplasmic extracts from embryos and resolved retained polypeptides by SDS-PAGE. Four bands between 30 and 15 kDa (P30, P21, P20 and P15) were consistently retained by GST-CortoCD and were enriched in peptide pull-down experiments performed with nuclear extracts *versus* cytoplasmic extracts (Figure 3A). The contents of the bands were identified by mass spectrometry. Surprisingly, all four bands contained ribosomal proteins (RPs): RPL7 for P30, RPS11 for P21, RPS10, RPL12 and RPL27 for P20, and RPS14 for P15 (Table S2). Although RPs are usually considered as contaminants, their consistent enrichment after incubation with nuclear extracts as well as the previously shown association of RPS11, RPL12 and RPS14 with polytene chromosomes [22] prompted us to consider their binding to CortoCD. These proteins might then interact with Corto directly on chromatin. To verify the interaction between RPs and CortoCD, we generated vectors to produce FLAG-tagged CortoCD supplied with a nuclear localization signal and Myc-tagged RPs in *Drosophila* S2 cells. Co-immunoprecipitations were performed on cell extracts from transfected cells, using either anti-FLAG or anti-Myc antibodies. No co-immunoprecipitation was observed between CortoCD and RPL7, RPS10 or RPS14 (Figure S1). However, anti-FLAG co-immunoprecipitated Myc-RPL12 with FLAG-CortoCD whereas anti-Myc co-immunoprecipitated FLAG-CortoCD with Myc-RPL12 (Figure 3B). In a similar experiment using FLAG-tagged full-length Corto, co-immunoprecipitation was again observed in both directions (Figure 3C). However, no co-immunoprecipitation was observed between FLAG-tagged CortoΔCD and Myc-tagged RPL12 (Figure 3D). These experiments demonstrate that RPL12 and Corto interact and that the Corto chromodomain is necessary and sufficient for this interaction. The identification of other RPs among the pulled-down polypeptides suggests that CortoCD interacts with a complex of RPs *via* a direct interaction with RPL12.

### The Corto chromodomain interacts with RPL12 tri-methylated on lysine 3

**Figure 4:**
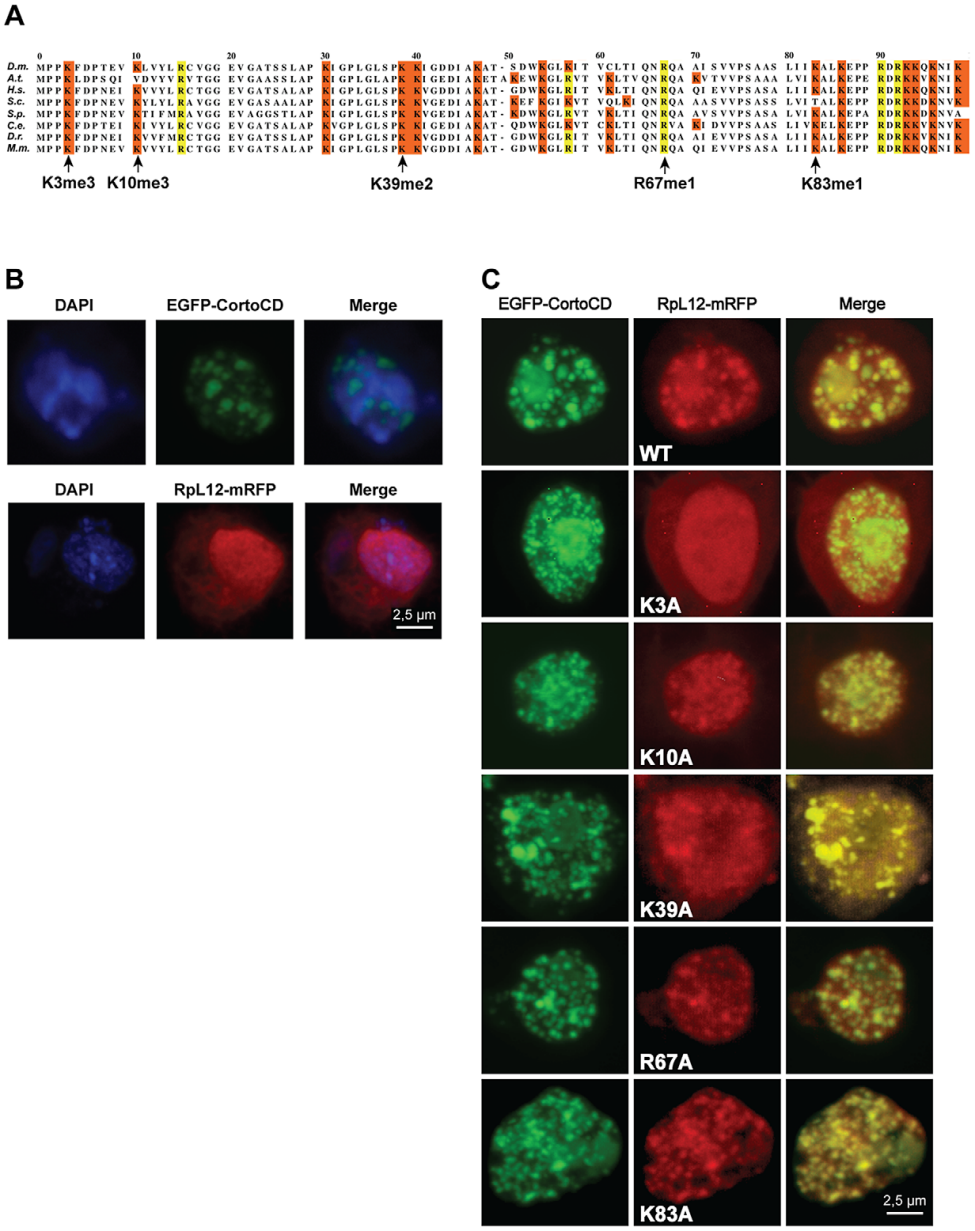
Lysine 3 of RPL12 is essential for interaction with Corto. (A) PRALINE multiple sequence alignment of the 100 first residues of RPL12 from different eukaryotes showing conserved lysines (highlighted in orange) or arginines (highlighted in yellow) known to be methylated in several species. *Drosophila melanogaster* (*D.m.*; AE013599), *Arabidopsis thaliana* (*A.t.*; AAD18140), *Homo sapiens* (*H.s.*; NM_000976), *Saccharomyces cerevisiae* (*S.c.*; NP_010860), *Schizosaccharomyces pombe* (*S.p.*; NP_587897), *Cænorhabditis elegans* (*C.e.*; NP_502542), *Danio rerio* (*D.r.*; AAI65413) and *Mus musculus* (*M.m.*; CAM22324). Lysine 3 can be trimethylated in *H.s*, *S.c*, *S.p.* and *A.t.*; lysine 10 can be trimethylated in *S.c.*; lysine 39 can be dimethylated in *S.p.*; lysine 83 can be monomethylated in *E. coli*, arginine 67 can be δ-monomethylated in *S.c.* and *S.p.* (see references in the text). (B) *Drosophila* S2 cells expressing either EGFP-CortoCD (top) or RPL12-mRFP (bottom). CortoCD was provided with a nuclear localization signal to force its entry into the nucleus. Note its punctuated pattern in the nucleus. RPL12-mRFP was present in both nuclear and cytoplasmic compartments. (C) Simultaneous expression of EGFP-CortoCD and wild-type or mutant RPL12-mRFP in *Drosophila* S2 cells. EGFP-CortoCD perfectly co-localized with wild-type RPL12-mRFP, as well as with RPL12K10A, RPL12K39A, RPL12R67A and RPL12K83A mutants within nuclei, exhibiting a punctuated pattern. Note that wild-type RPL12 and these mutant forms were not detected in the cytoplasm. RPL12K3A did not present a punctuated nuclear pattern and was detected in the cytoplasm. Scale bar: 2.5 µm.

Since chromodomains typically recognize methylated lysines, we asked whether Corto chromodomain could bind a methylated form of RPL12. *D. melanogaster* RPL12 was aligned with RPL12 from several other species to identify conserved residues described to be methylated in some of them [23]–[25] (Figure 4A). Lysines 3, 10, 39 and 83, as well as arginine 67 fulfilled these criteria. Using site-directed mutagenesis, we replaced their codons with alanine codons in the *Drosophila* RPL12 cDNA, thus generating a series of mutants (RPL12K3A, RPL12K10A, RPL12K39A, RPL12R67A and RPL12K83A). These mutant cDNAs were introduced into a plasmid allowing their expression as mRFP-tagged proteins in *Drosophila* S2 cells. Similarly, the *cortoCD* cDNA, supplied with a nuclear localization signal, was introduced into a plasmid allowing its expression as an EGFP-tagged protein in S2 cells. When expressed in these cells, EGFP-CortoCD artificially entered the nucleus where it exhibited a punctuated pattern that recalled Polycomb bodies (Figure 4B) [26]. A similar nuclear pattern was observed after immunostaining untransfected S2 cells with anti-Corto antibodies. However, these “Corto bodies” did not overlap with Polyhomeotic (PH), a component of the PRC1 complex, but with RNA Polymerase II suggesting that they were transcriptional factories rather than Polycomb bodies (Figure S2). RPL12-mRFP expressed alone was present in the cytoplasm and the nucleus, where it appeared slightly punctuated (Figure 4B). Interestingly, when co-expressed with EGFP-CortoCD, all RPL12-mRFP localized in the nucleus (Figure 4C). Both proteins perfectly colocalized in a punctuated nuclear pattern, corroborating the interaction between CortoCD and RPL12 and suggesting that Corto could drive RPL12 in the nucleus. Similar experiments were carried out using the RPL12 mutant forms. Whereas RPL12K10A, RPL12K39A, RPL12R67A and RPL12K83A co-localized with CortoCD, RPL12K3A did not, strongly suggesting that RPL12 lysine 3 is required for Corto chromodomain-RPL12 interaction (Figure 4C).

**Figure 5:**
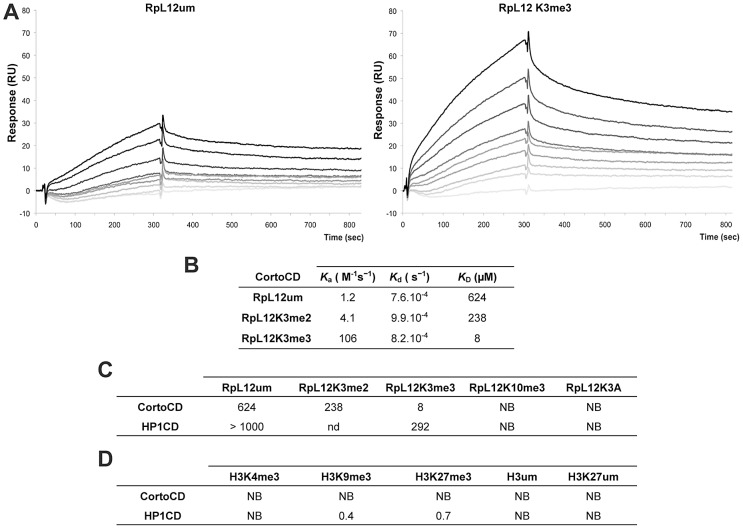
Preferential binding of Corto chromodomain to RPL12 trimethylated on lysine 3. (A) Biacore sensorgrams showing binding of either RPL12 unmethylated peptide (RPL12 um, left panel) or RPL12 peptide trimethylated on lysine 3 (RPL12K3me3, right panel) to CortoCD. Increasing concentrations of RPL12 um or RPL12K3me3 peptides were used [from 0 (light grey lines) to 10 µM (darker grey to black lines]. Binding (Y-axis, Response) is expressed in Resonance Units (RU) relative to time (X-axis). Note the response due to end of injection of the peptides at 300 s. (B) Kinetic parameters of interaction between CortoCD and RPL12 um, RPL12K3me2 or RPL12K3me3 peptides. Note that CortoCD interacts specifically with RPL12 trimethylated on lysine 3 (K_D_ = 8 µM). (C) Equilibrium dissociation constant (K_D_) calculated for CortoCD or HP1CD in interaction with RPL12 um peptide, RPL12 methylated peptides, or RPL12K3A peptide. Note that CortoCD specifically binds to RPL12K3me3 (K_D_<100 µM). (D) Equilibrium dissociation constant (K_D_) calculated from CortoCD or HP1CD interacting with unmethylated or trimethylated histone H3 peptides. For H3K9me3, peptide concentration was increased from 0 to 1 µM. For RPL12 and H3K27me3 peptide concentration was increased from 0 to 10 µM. NB: no binding; nd: not determined.

To test whether Corto directly interacted with RPL12 lysine 3, we measured real-time binding between CortoCD and several RPL12 peptides using Biacore. GST-CortoCD and GST were immobilized on a CM5 sensor chip. Then, several RPL12 peptides [unmodified (RPL12um), methylated on lysine 3 (RPL12K3me2, RPL12K3me3), methylated on lysine 10 (RPL12K10me3) or lysine 3 mutated (RPL12K3A)] were assayed for their binding to GST-CortoCD or GST (Figure 5, Figure S3). None of these peptides bound GST. Furthermore, unmodified RPL12, RPL12K3me2, RPL12K10me3 and RPL12K3A peptides did not interact with CortoCD (no binding or unspecific binding *i.e.* K_D_>200 µM; Figure 5C). Only RPL12K3me3 interacted with high specificity with CortoCD (K_D_ = 8 µM).

To investigate whether RPL12K3me3 could bind to other chromodomains, we repeated these experiments using that of HP1 (HP1CD). GST-HP1CD was immobilized on the sensor chip and binding of either RPL12, RPL12K3me3, RPL12K10me3 or RPL12K3A was tested. None of these peptides specifically interacted with HP1CD (K_D_>200 µM) (Figure 5C, Figure S3). Although no histones were revealed among peptides pulled down by CortoCD, we monitored binding of several histone H3 peptides to CortoCD. No binding of unmodified H3, H3K27me3, H3K9me3 or H3K4me3 peptides was observed (Figure 5D) while, as expected, the H3K9me3 peptide bound HP1CD with high affinity (K_D_ = 0.4 µM). Surprisingly, the H3K27me3 peptide bound HP1CD with a similar affinity (K_D_ = 0.7 µM), probably because sequences adjacent to the chromodomain (*i.e.* the hinge region) are required for selective targeting [27].

Altogether these data demonstrate that the Corto chromodomain specifically recognizes RPL12 trimethylated on lysine 3 (RPL12K3me3).

### Chromatin environment of Corto and RPL12

**Figure 6:**
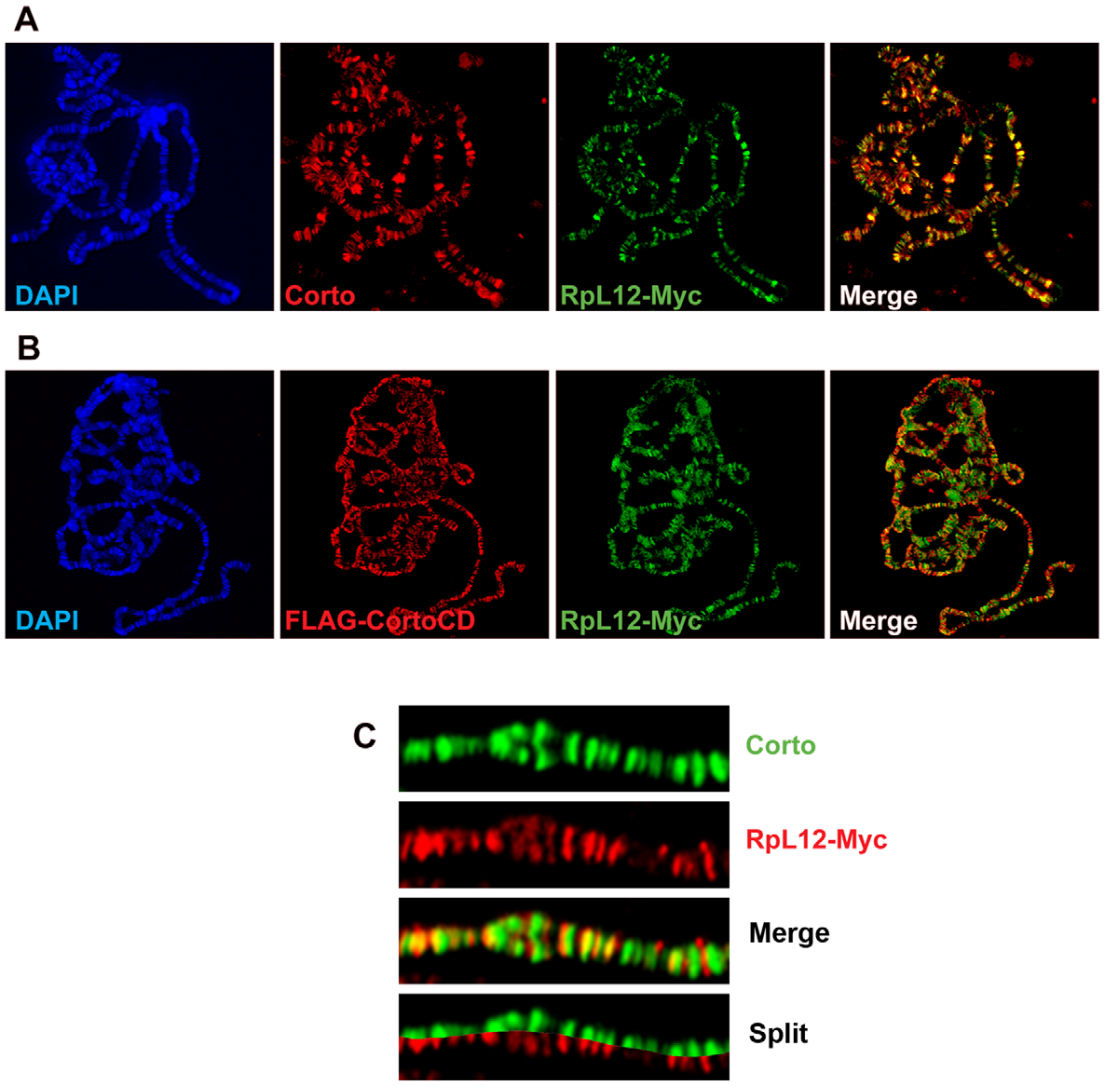
Corto and RPL12 share many sites on polytene chromosomes. (A) Polytene chromosomes from an *esg::Gal4>UAS::RpL12-Myc* larva immunostained with anti-Corto (red) and anti-Myc (green) antibodies. Many sites were common to Corto and RPL12-Myc. (B) Polytene chromosomes from an *esg::Gal4>UAS::FH-cortoCD*,*UAS::RpL12-Myc* larva immunostained with anti-FLAG (red) and anti-Myc (green) antibodies. Many sites were common to CortoCD and RPL12-Myc. (C) Close-up showing numerous co-localizations of RPL12 and endogenous Corto.

RPL12, along with 19 other ribosomal proteins, is known to bind polytene chromosomes of *Drosophila* larval salivary glands where it specifically associates with sites of transcription [22]. To investigate the potential role of the Corto-RPL12 interaction in gene expression regulation, we first analyzed the binding of these proteins on polytene chromosomes. For this, we generated Myc-tagged RPL12 transgenic fly lines (*UAS::RpL12-Myc*). Unlike *corto* or *cortoCD*, *RpL12-Myc* over-expression using ubiquitous Gal4 drivers (*da::Gal4>UAS::RpL12-Myc* or *Act::Gal4>UAS::RpL12-Myc*) induced no lethality and adult flies presented no visible phenotype except a shortened development (data not shown). *RpL12-Myc* was then expressed in salivary glands with the *esg* driver (*esg::Gal4>UAS::RpL12-Myc*) to test its binding to polytene chromosomes. RPL12-Myc bound polytene chromosomes at numerous sites, preferentially at DAPI interbands and puffs, suggesting that it mimics the binding of endogenous RPL12 [22] (Figure 6A). Co-immunostaining of RPL12 and the endogenous Corto protein showed that about 40% of the Corto sites were bound by RPL12 (Figure 6A, 6C). Simultaneous over-expression of FH-CortoCD and RPL12-Myc (*esg::Gal4>UAS::FH-cortoCD,UAS::RpL12-Myc*) established that CortoCD co-localized with RPL12 on a similar number of sites (Figure 6B).

**Figure 7:**
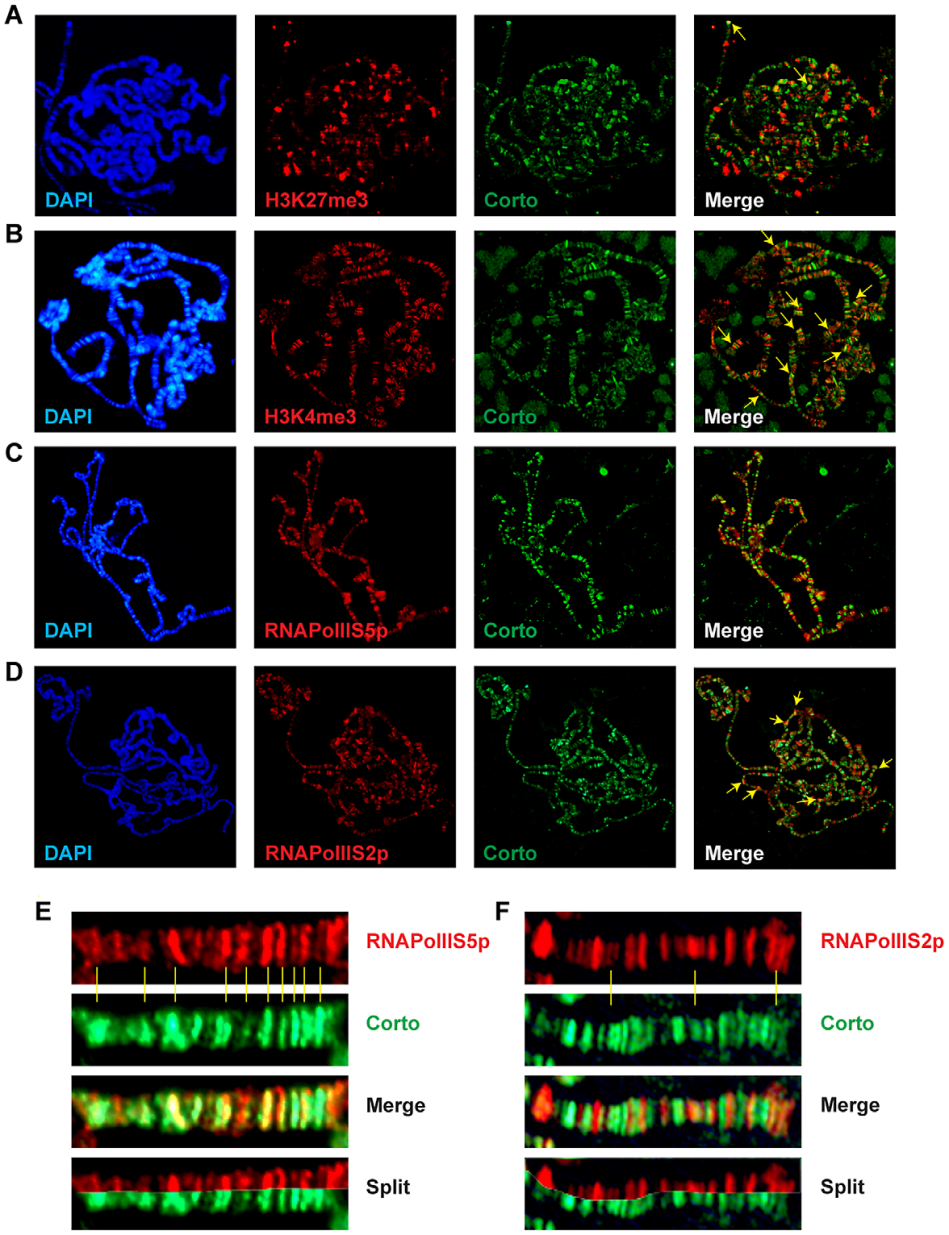
Chromatin environment of Corto. Co-immunostainings of salivary gland polytene chromosomes from *w^1118^* larvae using anti-Corto antibodies (green) and anti-H3K27me3 (A), anti-H3K4me3 (B), anti-RNAPolIIS5p (C) or anti-RNAPolIIS2p (D) antibodies (red). The two arrows in A point to X chromosome tips from two nuclei. Some co-localizations between Corto and H3K4me3 or RNAPolIIS2p are shown with yellow arrows on the merged pictures. (E) Close-up showing co-localizations of Corto and RNAPolIIS5p. (F) Close-up showing rare co-localizations of Corto and RNAPolIIS2p.

**Figure 8:**
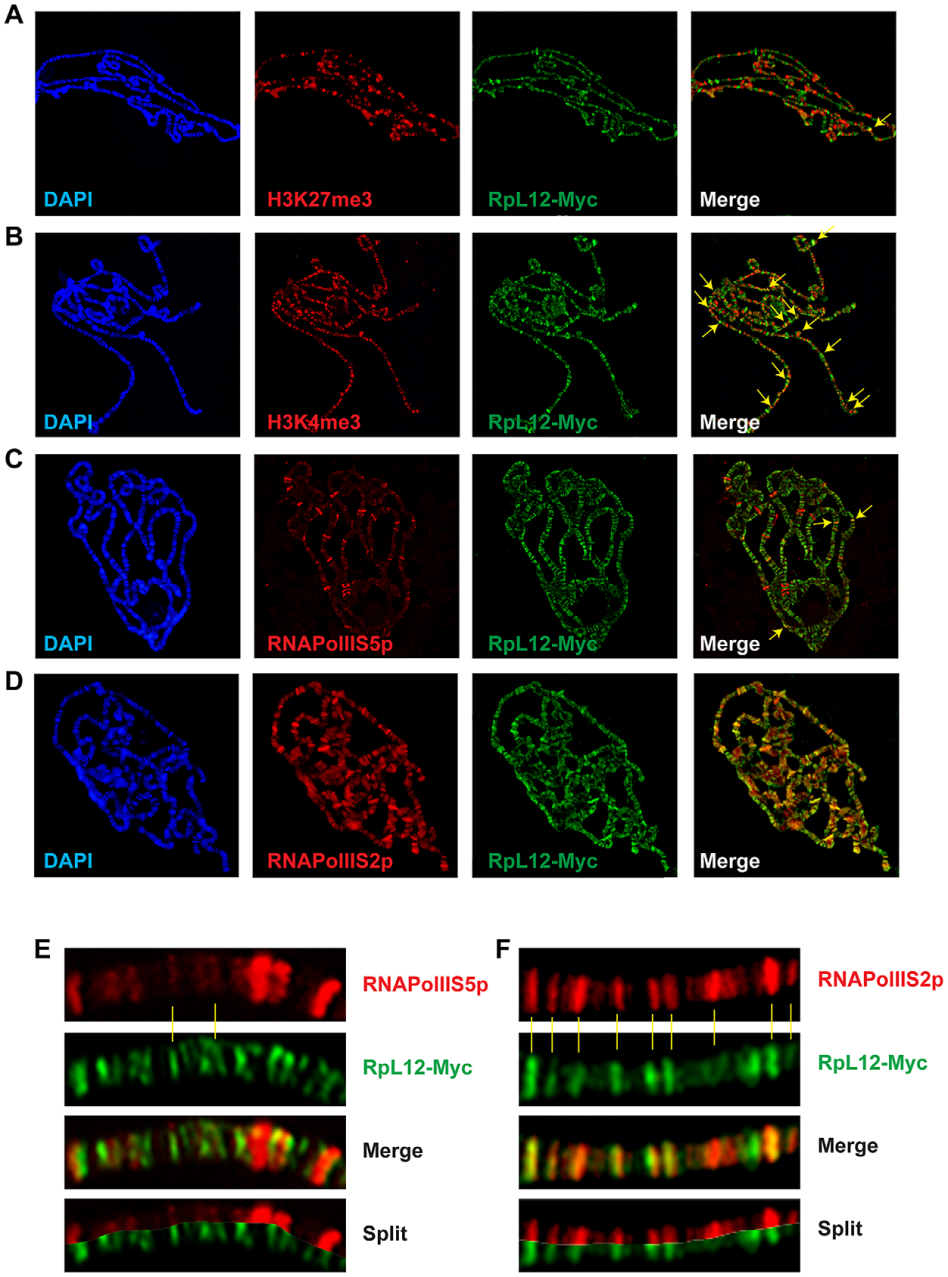
Chromatin environment of RPL12. Co-immunostainings of salivary gland polytene chromosomes from *esg::Gal4>UAS::RpL12-Myc* larvae using anti-Myc antibodies (green) and anti-H3K27me3 (A), anti-H3K4me3 (B), anti-RNAPolIIS5p (C) or anti-RNAPolIIS2p (D) antibodies (red). Some co-localizations between RPL12-Myc and H3K27me3, H3K4me3 or RNAPolIIS5p are shown with yellow arrows on the merged pictures. (E) Close-up showing few co-localizations of RPL12 and RNAPolIIS5p. (F) Close-up showing numerous co-localizations of RPL12 and RNAPolIIS2p.

Chromatin environment of Corto and RPL12 was further analyzed using antibodies against epigenetic marks (H3K27me3, H3K4me3) and RNA Polymerase II (paused, *i.e.* phosphorylated on serine 5: RNAPolIIS5p; elongating *i.e.* phosphorylated on serine 2: RNAPolIIS2p) (Figure 7 and Figure 8). In agreement with our Biacore analyses, Corto did not bind centromeric heterochromatin – marked by H3K9me3 – and did not overlap with H3K27me3 (except at the tip of chromosome X) (Figure 7A). Similarly, very few co-localizations with H3K27me3 were observed for RPL12-Myc (Figure 8A). Corto, as well as RPL12-Myc, partially co-localized with H3K4me3 (Figure 7B, Figure 8B). However, whereas Corto showed preferential co-localization with RNAPolIIS5p *versus* RNAPolIIS2p (Figure 7), RPL12 shared but few sites with RNAPolIIS5p and strongly co-localized with RNAPolIIS2p (Figure 8), as previously described [22].

Taken together, these data suggest that Corto and RPL12 mostly bind open, transcriptionally permissive chromatin.

### Genome-wide transcriptome analysis of wing imaginal discs over-expressing *cortoCD* or *RpL12*

**Figure 9:**
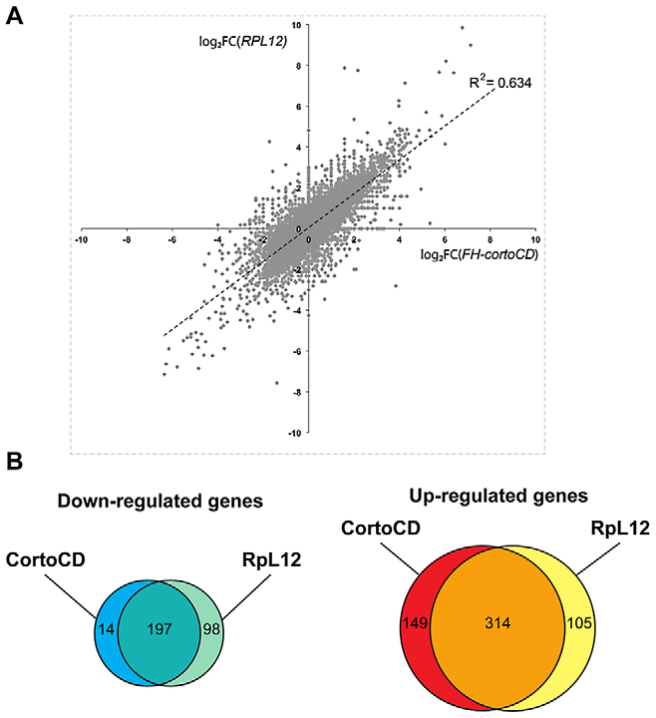
Comparison of genes deregulated by *cortoCD* and *RpL12* over-expression. (A) Scatter plot of log_2_ fold changes (FC) showing (*sd::Gal4>UAS::FH-cortoCD vs sd::Gal4/+*) on X-axis and (*sd::Gal4>UAS::RpL12-Myc vs sd::Gal4/+*) on Y-axis before cutoff. Note the high correlation coefficient (R^2^ = 0.634). (B) Venn diagrams showing the intersection of genes deregulated in *FH-cortoCD* and *RpL12-Myc* over-expressions after cutoff [*P*-value<4.10^−18^; absolute log_2_(assay/control)>1]. See Tables S3, S4, S5, S6, S7 for detailed gene lists.

**Figure 10:**
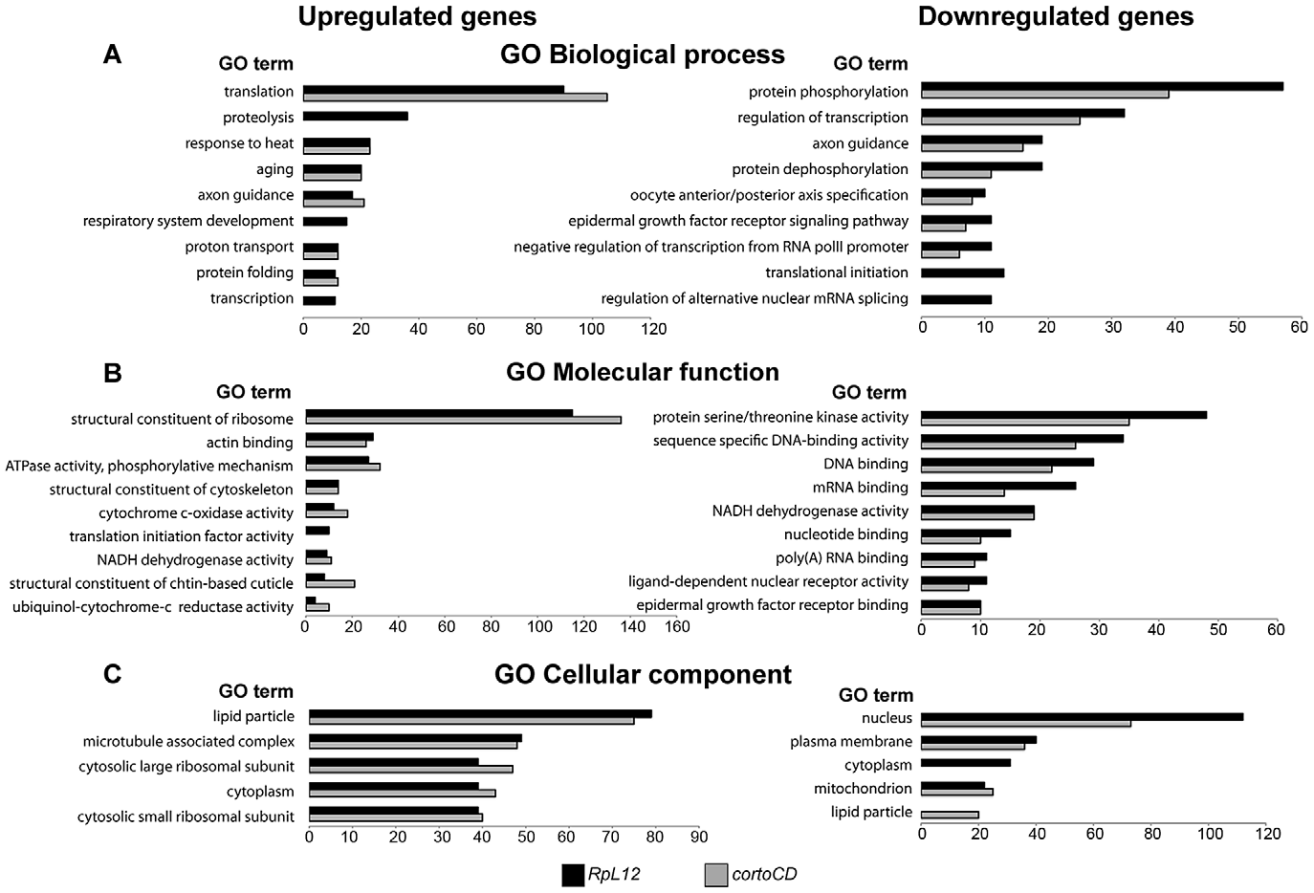
Ontology of genes deregulated in wing imaginal discs over-expressing *cortoCD* or *RpL12*. Gene Ontology (GO) term enrichment of genes deregulated by CortoCD and RPL12 considering DAVID identification [67]. Highly represented, non-redundant categories were selected according to the hypergeometric test adjusted *P*-values. The number of genes in each category is shown on the X-axis. GO of biological process (A), molecular function (B) and cellular component (C) of genes upregulated (left) and down-regulated (right) by over-expression of *RpL12* (black) or *cortoCD* (grey). Full GO data (GO ID, description, number of genes in each category, enrichment and adjusted *P*-values) for each category are presented in Tables S8, S9, S10, S11.

To address the role of Corto and RPL12 in transcriptional regulation, we deep-sequenced transcripts from wing imaginal discs of third instar larvae over-expressing either *FH-cortoCD* or *RpL12-Myc* under control of the wing-specific *scalloped::Gal4* driver (*sd::Gal4>UAS::FH-cortoCD* or *sd::Gal4>UAS::RpL12-Myc*) (hereafter called assays). Total RNA from the assays, the *sd::Gal4/+* control or a *w^1118^* reference line were isolated from pools of wing imaginal discs and subjected to RNA-seq on an Illumina high throughput sequencer. Sequence reads were aligned with the *D. melanogaster* genome to generate global gene expression profiles. Sequence reads of the assays were compared to sequence reads of the *sd::Gal4/+* control. Differential analyses were performed to obtain adjusted *P*-values associated to expression changes for the assays compared to the *sd::Gal4/+* control. In addition, sequence reads from the *w^1118^* reference line were compared to sequence reads of the *sd::Gal4/+* control. This reference was used to fix the threshold of the adjusted *P*-value to get only 1% of transcripts as differentially expressed in this control experiment (false discovery rate). By doing so, we obtained an adjusted *P*-value cutoff of 4.10^−18^. Using this threshold, we retrieved the highest expression variations from the two assays [with absolute log_2_(assay/control)>1]. 463 genes were upregulated when over-expressing c*ortoCD* (Table S3). Among them, 314 were also upregulated when over-expressing *RpL12*, representing 75% of all genes upregulated by *RpL12* over-expression (Table S4). Furthermore, 211 genes were down-regulated when over-expressing *cortoCD* (Table S5). Among them, 197 were also down-regulated when over-expressing *RpL12*, representing 67% of all genes down-regulated by *RpL12* over-expression (Table S6). These results are summarized on Figure 9 and Table S7. They suggest that Corto and RPL12 share many transcriptional targets. Strikingly, analysis of Gene Ontology (GO) revealed that common upregulated genes were enriched in the “translation” (54.4% for Corto and 38.3% for RPL12) and “response to heat” (11.9% for Corto and 9.8% for RPL12) categories (Figure 10 and Tables S8, S9, S10, S11).

### *hsp70* is a direct transcriptional target of Corto and RPL12

**Figure 11:**
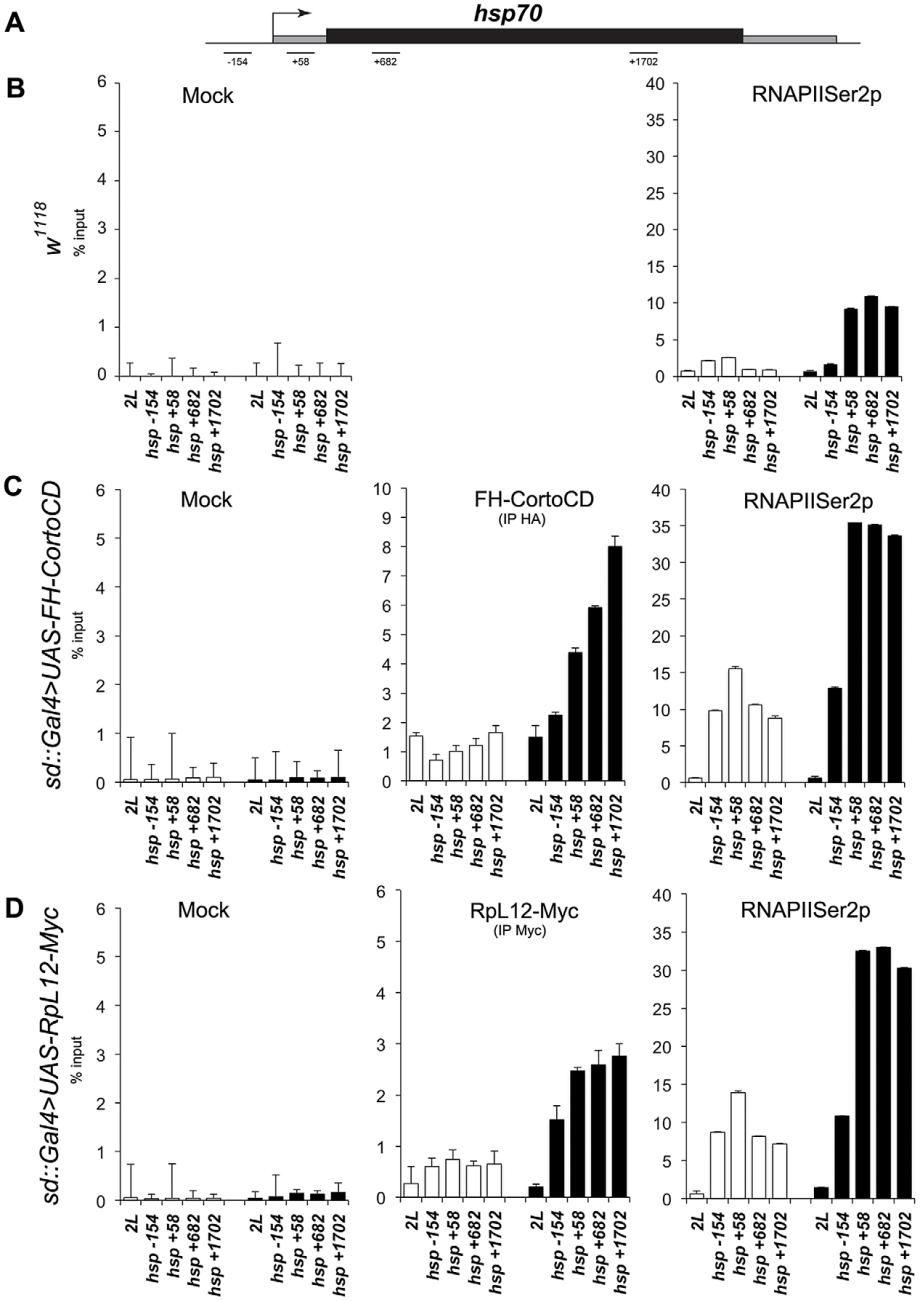
Occupancy of the *hsp70* gene by CortoCD and RPL12 is enhanced upon heat shock. (A) General structure of *hsp70* genes. qPCR amplicons are indicated. Coordinates of qPCR amplicons relative to *hsp70* transcriptional start site are indicated (from [28]). (B) ChIP-qPCR analysis of *hsp70* from *w^1118^* wing imaginal discs using mock or anti-RNAPolIISer2p antibodies. (C) ChIP-qPCR analysis of *hsp70* from *sd::Gal4>UAS-FH-CortoCD* wing imaginal discs using mock, anti-HA, or anti-RNAPolIISer2p antibodies. (D) ChIP-qPCR analysis of *hsp70* from *sd::Gal4>UAS-RpL12-Myc* wing imaginal discs using mock, anti-Myc, or anti-RNAPolIISer2p antibodies. White and black histograms represent ChIP-qPCR data without or with 5 minutes heat shock, respectively. 2L: negative control amplified from an intergenic region located on the left arm of chromosome 2 (coordinates 11.413.862 to 11.413.946) that does not present any read in our RNA-seq analysis and is enriched in H3K27me3 in wing imaginal dics [64]. Error bars represent coefficients of variation obtained from 4 independent experiments.

The high correlation between genes deregulated when over-expressing either *cortoCD* or *RpL12* (R^2^ = 0.634) (Figure 9) as well as the numerous co-localizations of CortoCD and RPL12 on polytene chromosomes suggest that some deregulated genes were direct targets of Corto and RpL12. To test this hypothesis and to get insight in the functional interaction between Corto and RPL12, we focused on *hsp70* that was one of the shared upregulated genes (Figure S4). We analyzed binding of CortoCD and RPL12 by chromatin immunoprecipitation before and after heat shock in wing imaginal discs (Figure 11). qPCR analyses were performed using a set of primers that cover the promoter and gene body of *hsp70* [28].

At 25°C, in control *w^1118^* discs, higher RNAPolII occupancy of the promoter as compared to the gene body suggests that *hsp70* was paused, corroborating previous results [28]. In wing imaginal discs overexpressing either *cortoCD* or *RpL12* (*sd::gal4>UAS-FH-CortoCD* or *sd::gal4>UAS-RpL12-Myc*), CortoCD and RPL12 bound *hsp70* indicating that this gene was a direct target of both proteins. Simultaneously, RNAPolII binding was increased but kept the same profile suggesting that the gene was still paused but more loaded with RNAPolII. This could explain why more transcripts were generated. Thus, these data suggest that Corto, as well as RPL12, favors recruitment of RNAPolII on *hsp70* in absence of heat shock.

After a short heat shock (5 minutes), CortoCD, as well as RPL12, were massively recruited on *hsp70*. Interestingly, CortoCD and RPL12 displayed the same binding profile *i.e.* increased binding from 5′ to 3′ of the gene body. CortoCD and RPL12 recruitment followed *hsp70* transcription as revealed by enhancement of RNAPolII on gene body. Strikingly, recruitment of RNAPolII was higher in wing discs expressing *cortoCD* or *RpL12* than in control wing discs, suggesting that Corto and RPL12 control transcriptional activation of *hsp70*.

## Discussion

Chromodomains play a critical role in addressing transcriptional regulators to chromatin. Investigating the role of the ETP Corto's chromodomain, we found that it is a typical chromodomain, acting as a chromatin-targeting module. Surprisingly, the Corto chromodomain does not bind methylated histones, as most known chromodomains do, but Ribosomal Protein L12 trimethylated on lysine 3 (RPL12K3me3). In agreement, RPL12 and Corto share many sites on polytene chromosomes. Transcriptomic analyses of wing imaginal tissues in which either *cortoCD* or *RpL12* were over-expressed reveal that a large fraction of deregulated genes are common. Chromatin immunoprecipitation experiments reveal that CortoCD and RPL12 similarly bind one of these shared upregulated genes, *hsp70*, and are massively loaded on the promoter and gene body after heat shock. Hence, the ETP Corto and RPL12 might indeed be partners in regulation of some transcriptional targets.

### RPs and regulation of gene expression

The ETP Corto is a partner of Polycomb and Trithorax complexes and participates in epigenetic maintenance of gene expression, notably of homeotic genes [15],[17]. Multiple Corto binding sites on polytene chromosomes as well as pleiotropic phenotypes of *corto* mutants show that Corto transcriptional targets are numerous and involved in many developmental pathways. The interaction reported here between Corto and RPL12 raises the interesting possibility of a connection between RPs and epigenetic regulation of gene expression. Our previous investigations into Corto partners have highlighted its interaction with several PcG proteins, leading to the conclusion that Corto might regulate PRC1 and PRC2 functions [18]. Strikingly, RPs also co-purify with PRC1 [29]. Moreover, the ETP DSP1, that binds Corto, directly interacts with RPS11 [30]. Another ETP, ASXL1, belongs to the repressor complex H1.2 that also contains RPs [31]. Presence of RPs in the direct environment of chromatin binding factors, notably ETP, seems then to be a widespread situation. However, the role of RPs in these cases could be related to structure preservation and not to transcriptional regulation *per se*.

Apart from protein synthesis, RPs are involved in many cellular functions referred to as “extra-ribosomal” (reviewed in [32]). The first report on an RP's role in transcriptional regulation came from *E. coli* where RPS10 is involved in anti-termination of transcription [33]. Many eukaryotic RPs, notably RPL12, regulate their own transcription, basically by regulating their own splicing (reviewed in [34]).

For more than 40 years, many genetic screens to isolate new *Polycomb* (*PcG*) and *trithorax* (*trxG*) genes in flies have identified *Minute* mutants as *PcG* and *trxG* modifiers [14]. Indeed, *Minute* mutations suppress the ectopic sex comb phenotype of *Polycomb* or *polyhomeotic* mutants [35], [36]. *D. melanogaster Minute* loci are disseminated throughout the genome and many correspond to RP genes ([37] and references therein). *Minute* mutations might indirectly suppress phenotypes of *PcG* mutants by lengthening development, thus globally counteracting homeosis. However, *Minute* mutants can exhibit *PcG* mutant phenotypes, which is at variance with this assumption. For example, mutants in *stubarista* that encodes RP40 exhibit transformation of arista into legs [38].

Quasi-systematic presence of RPs at sites of transcription on Drosophila polytene chromosomes [22] as well as direct interaction between several RPs and histone H1 in transcriptional repression [39], suggest that RPs could actively participate in transcription modulation. Massive recruitment of Corto and RPL12 on *hsp70* upon transcriptional activation as well as similarity between their occupancy profiles and the one of RNA polymerase II suggest that these two proteins could travel along the gene body together with the transcriptional machinery. Interestingly, BRM, the catalytic subunit of the SWI/SNF TrxG complex, associates with components of the spliceosome [40] that contains several RPs including RPL12 [41]. Overall, these findings lead us to favor the hypothesis of an active involvement of RPs in regulation of gene expression.

### RP combinations and post-translational modifications, a ribosomal code for transcription?

Whether individual RPs regulate transcription independently of other RPs or in the context of a ribosome-like complex is an interesting and much debated question (reviewed in [42]). Many data point to a collaborative role of RPs in transcription. In *D. melanogaster*, at least 20 RPs as well as rRNAs are present at transcription sites on polytene chromosomes, suggesting that they could be components of ribosome-like subunits [22]. Genome-wide ChIP-on-chip analyses of RPL7, L11 and L25 in *S. pombe* reveal a striking similarity of their binding sites, suggesting that they might bind chromatin as complexes [43]. Along the same line, mass spectrometry of Corto partners identified not only RPL12 but also RPL7, L27, S10, S11 and S14, indicating that Corto might interact *via* RPL12 with several RPs that could form a complex. Interestingly, RPL12 and L7 form a flexible protruding stalk in ribosomes that acts as a recruitment platform for translation factors [44]. Our results might point to the existence of pseudo-ribosomes composed of several RPs on chromatin. The role of RPs in nuclear translation has been very much debated and whether these pseudo-ribosomes are involved in translation is still unknown [45]. However, this possibility seems unlikely in view of the numerous data showing lack of translation factors in nuclei as well as association on chromatin between RPs and both nascent coding and non-coding RNAs [46]. Overall, these data suggest that pseudo-ribosomal complexes composed of various RPs are associated on chromatin and could thus participate in transcriptional regulation.

Like histones, RPs are subjected to a plethora of post-translational modifications including ubiquitinylations, phosphorylations, acetylations and methylations ([47] and references therein). We show here that the Corto chromodomain binds RPL12K3me3. Strikingly, the chromodomain protein CBX1, a human homolog of *Drosophila* HP1β, also interacts with RPL12 [48], suggesting that chromodomain binding to methylated RPL12 might be conserved. It is tempting to speculate about a role for RPL12 methylation in chromodomain protein recruitment to chromatin. This mechanism might be analogous to the one by which histone methylation marks, such as H3K27me3, recruit the PRC1 complex, *i.e.* by binding of the Polycomb chromodomain to methyl groups. Under this hypothesis, RPL12K3me3 might recruit Corto to chromatin. In yeast and *A. thaliana*, RPL12 can be trimethylated on lysine 3 by methyl-transferase SET11/Rkm2 [25], [47], [49]. Rkm2 is conserved in *Drosophila* and abundantly transcribed in S2 cells as well as all along development [50]. It would be interesting to determine whether this enzyme is an RPL12K3 methyl-tranferase in *Drosophila*.

Based on the existence of a panel of ribosomes composed of diverse RPs bearing various post-translational modifications, it was proposed that selective mRNA translation might depend on a ribosome code similar to the histone code [51]. Our results lead us to suggest that such a ribosome code might also concern regulation of gene transcription.

### Ribosomal homeostasis by coordinated transcriptional regulation of RPs

Surprisingly, GO analysis of RPL12 and Corto upregulated genes reveals that the “translation” and “structural component of ribosomes” categories are over-represented. Interestingly, the expression of RP genes decreases in *RPL12A* mutants in yeast [51]. Our finding that over-expression of *Drosophila RpL12* increased RP gene expression reinforces the idea that RPL12 can activate RPs at the transcriptional level. Moreover, up-regulation of ribosome related genes is also observed in mutants of *ash2* that encodes a TrxG protein, and that genetically interacts with *corto* [15], [52]. Hence RPL12, Corto and chromatin regulators of the TrxG family might all participate in dynamic coordination of ribosome biogenesis thus controlling cell growth. Intriguingly, we have recently shown that Corto interacts with an atypical cyclin, namely Cyclin G that also binds chromatin. This cyclin is suspected to control transcription of many genes, and controls cell growth [17], [53], [54]. These combined findings provide new avenues of research concerning transcriptional regulation of tissue growth homeostasis. Global regulation of genes involved in ribosome biogenesis could be a way to maintain this homeostasis. Co-regulation of genes involved in a given function has already been documented in eukaryotes. In *Drosophila*, housekeeping genes are co-regulated by the NSL complex and, in yeast, RPL12 coordinates transcription of genes involved in phosphate assimilation as well as RP genes [51], [55], [56]. As regulation of ribosome biogenesis is essential for cellular health and growth homeostasis [57], such a transcriptional co-regulation of RP genes might have evolved to insure that the cell's protein synthesis capacity can be rapidly adjusted to changing environmental conditions.

## Materials and Methods

### Cloning and site-directed mutagenesis

Clones and site-directed mutagenesis are described in Text S1. Primers are described in Table S12.

### Drosophila strains and genetics

*D. melanogaster* stocks and crosses were kept on standard medium at 25°C. *UAS::FH-cortoCD*, *UAS::FH-cortoΔCD* and *UAS::RpL12-Myc* transgenic lines were established by standard *P*-element mediated transformation. Over-expression was carried out using *Gal4* drivers either ubiquitous [*daughterless* (*da::Gal4*); *Actin5C* (*Act::Gal4*)], expressed in salivary glands [*escargot* (*esg::Gal4*)], or wing-specific [*scalloped* (*sd::Gal4*)]. Five females bearing the *Gal4* driver were crossed with three males bearing the *UAS* transgene or *w^1118^* as a control. Crosses were transferred to new vials every third day. The *sd::Gal4*, *UAS::FH-cortoCD* and *UAS::RpL12-Myc* lines were isogenized for six rounds with the isogenic *w^1118^* line, prior to deep-sequencing, as described [58]. Lethality was calculated as described [59].

### Peptide pull-down experiments and mass spectrometry

Cytoplasmic and nuclear extracts were prepared from 0–16 h embryos as described in [60]. GST or GST-CortoCD were covalently linked on agarose beads using the GST orientation kit (Pierce) following the manufacturer's instructions. 1 mg of protein extract was incubated with 200 µg of purified GST or GST-CortoCD in binding buffer [0.5 mM DTT, 0.1 mM EDTA, 4 mM MgCl_2_, 0.05% Igepal, 20 mM Hepes, 300 mM KCl, 10% glycerol, protease inhibitor cocktail (Roche)] for 1 h at 25°C. After 5 washes in binding buffer, bound polypeptides were resolved on a large 15% SDS–polyacrylamide gel and stained either with EZblue (Sigma) or with SilverQuest staining kit (Invitrogen). Bands were excised from the gel and were analyzed by LC-MS/MS mass spectrometry.

### Cell culture and transfection

S2 cells were cultured at 25°C in Schneider's *Drosophila* medium (Lonza) supplemented with 10% heat inactivated fetal bovine serum and 100 units.mL^−1^ of penicillin and streptomycin. Cells were transfected using Effecten (Qiagen) as described [61].

### Co-immunoprecipitations

Co-immunoprecipitations were performed as described [61] using anti-FLAG (Sigma F-3165) or anti-Myc (Santa Cruz, sc-40).

### Confocal imaging

S2 cells were harvested 24 h after transfection and treated as described [62]. For each transfection, 30 to 60 nuclei were analyzed with an SP5 confocal microscope (Leica microsystems) using LAS (Image Analysis Software).

### Real-time protein interaction assays (Surface Plasmon Resonance)

GST or GST fusion proteins were dialyzed using a Slide-A-Lyser cassette (Thermo Scientific) in running buffer (10 mM Hepes pH7.4, 150 mM NaCl, 3 mM EDTA, 0.005% P20 surfactant) (GE Healthcare). Real-time protein interaction assays were performed using a Biacore 3000. Kinetics and binding tests were first performed on empty surfaces. Data presented here result from substraction of empty surface RU (1 to 5 depending on the experiment) from active surface RU. GST was covalently coupled to a CM5 sensor chip (GE Healthcare) *via* its N-terminal amino acid. The carboxymethylated dextran surface was activated by injecting a mixture of 0.2 M 1-ethyl-3-(3-dimethylaminopropyl) and 0.05 M N-hydroxysuccinimide. GST was immobilized on the chip by injecting a 30 µg.mL^−1^ solution in NaAc pH5 buffer. GST-CortoCD and GST-HP1CD were immobilized by injecting a 100 µg.mL^−1^ solution in the same buffer. Binding tests were performed by injecting peptides at 1 or 10 µM in running buffer at a flow rate of 5 µL.min^−1^ during 5 min. Kinetic assays were performed only when the binding test was positive. Real-time monitoring was displayed in a sensorgram as the optical response (RU) *versus* time (s). To calculate association constants, peptides were diluted in series from 1 to 10 µM in running buffer and dilutions were injected sequentially at a flow rate of 5 µL.mn^−1^ during 5 min. Dissociation kinetics were then run during 10 min to calculate dissociation constants. Between assays, the chip was regenerated with 10 mM glycine pH2.0. Kinetic constants were calculated with BIAevaluation Software (Biacore) using the Fit kinetic simultaneous ka/kd (1∶1 binding; Langmuir algorithm). RPL12 peptides were synthesized at the IFR83 Peptide synthesis facility (Table S13). Histone peptides were provided by Diagenode (H3K27me3: sp-069-050; H3K4me3: sp-003-050; H3K9me3: sp-056-050; H3K4/K9um: sp-999-050; H3K27um: sp-998-050).

### Immunostaining of polytene chromosomes

Polytene chromosome immunostainings were performed as described [63] for all antigens except RNA Pol II, for which we used experimental conditions described in [64]. Mouse anti-FLAG (1∶20) (Sigma, F-3165), mouse anti-Myc (1∶20) (Santa Cruz, sc-40), rabbit anti-H3K4me3 (1∶40) (Diagenode, pAB-003), rabbit anti-H3K27me3 (1∶70) (Diagenode, pAB-069), rabbit anti-RNA Pol II Ser2p (1∶200) (Abcam, an5095), rabbit anti-RNA Pol II Ser5p (1∶40) (Covance, MMS-134R) and rabbit anti-Corto (1∶30) [18] were used as primary antibodies. Secondary antibodies [Alexa Fluor 488 goat anti-rabbit IgG (Molecular Probes, A-11008), Alexa Fluor 594 goat anti-mouse IgG (Molecular probes, A-11005) and Alexa Fluor 488 goat anti-mouse IgG, IgA and IgM (Molecular Probes, A-10667)] were used at a 1∶1000 dilution.

### RNA–seq and bioinformatics analysis

Wing imaginal discs of third instar female larvae (one disc per larva) were dissected by batches of 50 in ice-cold PBS and frozen in liquid nitrogen. 300 discs (6 batches) were pooled and homogenized in lysis buffer using a FastPrep-24 during 20 s at 4 m.s^−1^ (MP Biomedicals, Lysing Matrix D). Total RNA were extracted using RNeasy kit (Qiagen). Library preparation and Illumina sequencing (multiplexed 50 bp paired-end sequencing on HiSeq 2000) were performed at the BC Cancer Agency Genome Sciences Center (Canada). Messenger (polyA+) RNAs were purified from 4 µg of total RNA with oligo(dT). Libraries were prepared using the bi-directional RNA-Seq library preparation kit (Illumina). A mean of 46±11 million reads was obtained for each of the 4 samples (*w^1118^*, *sd::Gal4/+*; *sd::Gal4>UAS::cortoCD*; *sd::Gal4>UAS::RpL12*). Detailed informations on Paired-End read counts at each step of the analysis workflow are available in Table S14. Before mapping, poly N read tails were trimmed, reads ≤11 bases were removed, and reads with quality mean ≤12 were discarded. Reads were then aligned against the *D. melanogaster* genome (dm3 genome assembly, BDGP Release 5.38) using Bowtie mapper (version 0.12.7) [65]. Alignments from reads matching more than once on the reference genome were removed using Java version of samtools. To compute gene expression, *D. melanogaster* GFF3 genome annotation from FlyBase (version 5.38) was used. All overlapping regions between alignments and referenced exons were counted.

Technical replicates coming from paired-end reads were first summed. Then, all samples were normalized together. Data were normalized according to the scaling normalization proposed by Robinson and Oshlack and implemented in the edgeR package version 1.6.10 [66]. A Fisher's Exact Test was then performed using the sage.test function of the statmod package version 1.4.6. Finally, a Benjamini and Hochberg (BH) *P*-value adjustment was made. The RNA-Seq gene expression data and raw fastq files are available at the GEO repository (www.ncbi.nlm.nih.gov/geo/) under accession number: GSE38435.

### Chromatin immunoprecipitation

Wing imaginal discs of third instar larvae were dissected by batches of 100 in serum-free Schneider medium at room temperature. They were fixed in 500 µL of paraformaldehyde 1% in PBS for 10 minutes at room temperature under gentle agitation. Cross-link reaction was stopped by adding 50 µL of glycine 1.25 M. Fixed wing discs were washed 3 times with PBS, dried, flash-freezed in liquid nitrogen and stored at −80°C. Cell lysis was performed by adding 100 µL of lysis buffer (140 mM NaCl, 10 mM Tris-HCl pH8.0, 1 mM EDTA, 1% Triton X-100, 0.1% sodium deoxycholate, Roche complete EDTA-free protease inhibitor cocktail) complemented with 1% SDS, and sonicated in a Bioruptor sonifier (Diagenode). Conditions were established to obtain chromatin fragments from 200 to 1000 bp in length (30″ ON 30″ OFF, high power, 15 cycles). Pooled chromatin was centrifuged for 10 min at 13000 g at 4°C. The supernatant (soluble chromatin) was recovered and 5 µL were kept as input sample. For each IP, 10 µl of 50% (v/v) protein A or G coated paramagnetic beads (Diagenode) were washed once in lysis buffer, 1 µg of antibody was added, and beads were incubated for 2 h at 4°C on a rotating wheel. After washing, antibody coated beads were resuspended in 450 µL of lysis buffer and 50 µl of chromatin were added. After incubation on a rotating wheel overnight at 4°C, beads were washed at 4°C five times for 10 min each in lysis buffer, once in LiCl buffer (Tris-HCl 10 mM pH8, LiCl 0.25 M, 0.5% NP-40, 0.5% sodium deoxycholate, 1 mM EDTA) and twice in TE (10 mM Tris-HCl, pH 8.0, 1 mM EDTA). Immunoprecipitated as well as input DNAs were purified with the IPure kit following the manufacturer's instructions (Diagenode). Elution was performed twice with 35 µl of water. 2 µl of DNA were used per PCR. Real-time PCR data were normalized against Input sample and depicted as percentage of Input (see Table S12 for primers).

Antibodies used for chromatin immunoprecipitation were anti-RNA Polymerase II S2p (Abcam, ab5095), anti-HA tag (Abcam, ab9110) and anti-Myc tag (Abcam, ab9132). Mouse IgGs were used as a negative control (Mock, Diagenode).

Heat shock treatments were performed as previously described [28]. Briefly, wing discs were subjected to instantaneous heat shock by addition of an equal volume of 48°C pre-heated Schneider medium. After keeping tubes at 37°C for 5 minutes, discs were immediately cooled down by addition of 1/3 total volume of 4°C medium.

## Supporting Information

Figure S1 CortoCD does not co-immunoprecipitate with nuclear ribosomal proteins RPL7, RPS10 and RPS14. (A) Transfection of FLAG-CortoCD and RPL7-Myc in S2 cells. (B) Transfection of FLAG-CortoCD and RPS10-Myc in S2 cells. (C) Transfection of FLAG-CortoCD and RPS14-Myc in S2 cells. Immunoprecipitations were performed with anti-Myc and revealed by Western blot with either anti-Myc (α-Myc) or anti-FLAG (α-FLAG). Spnt: supernatant, IP: immunoprecipitation.
(TIF)

Figure S2 Corto overlaps with transcriptional factories. (A) Immunostaining of S2 cells with anti-Corto (green) and anti-PH (red) antibodies showing that Corto bodies and Polycomb bodies did not overlap. Blue: DAPI. Close-up of a nucleus. (B) Immunostaining of S2 cells with anti-Corto (green) and anti-RNAPolII (red) antibodies showing that Corto bodies and transcriptional factories overlapped. Blue: DAPI. Close-up of a nucleus.
(TIF)

Figure S3 Real-time interaction binding assays. (A) Biacore sensorgram showing binding of H3K9me3 peptide (1 µg.mL-1) to GST-HP1CD, GST-CortoCD or GST. Binding (Y-axis, Response) is expressed in Resonance Unit (RU). Note that H3K9me3 bound GST-HP1CD as expected but did not bind GST-CortoCD or GST. (B) Biacore sensorgram showing binding of RpL12K3me3 or RpL12K3A peptides (10 µg.mL-1) to GST-HP1CD, GST-CortoCD or GST. Binding (Y-axis, Response) is expressed in resonance unit (RU).
(TIF)

Figure S4 *hsp70* genes are upregulated when either *RpL12-Myc* or *FH-CortoCD* are expressed in wing imaginal discs. (A) Read count data and log_2_ fold change (FC) analysis relative to *sd::Gal4/+* for each *hsp70* transcript. (B) IGV (Integrative Genomics Viewer) screenshot showing read alignments along the *hsp70Ab* locus (3R:7,781,701–7,788,799) in control *w^1118^*, *sd::Gal4>UAS-RpL12-Myc* and *sd::Gal4>UAS-FH-cortoCD* wing imaginal discs. Sense reads appear in pink and reverse reads in blue.
(TIF)

Table S1 Phenotypes of flies overexpressing *cortoCD* using ubiquitous Gal4 drivers. Three different insertions of the *cortoCD* transgene (named 231, 41 and 45) and one full-length *corto* transgene were analysed. nd: not determined. *: difference with driver alone highly significant (*p<1.5 10^−10^*, T Test). **: difference with driver alone significant (*p = 0.03*, Fisher's exact test); ***: difference with driver alone highly significant (*p<0.005*, Fisher's exact test).
(PDF)

Table S2 Mass spectrometry analysis of peptides pulled down by CortoCD. Four bands (P30, P21, P20 and P15) were excised from the gel (see Figure 4) and analyzed by mass spectrometry.
(PDF)

Table S3 Genes up-regulated in *sd::Gal4>UAS::FH-cortoCD vs sd::Gal4/+*. FC: Fold Change.
(PDF)

Table S4 Genes up-regulated in *sd::Gal4>UAS::RpL12-Myc vs sd::Gal4/+*. FC: Fold Change.
(PDF)

Table S5 Genes down-regulated in *sd::Gal4>UAS::FH-cortoCD vs sd::Gal4/+*. FC: Fold Change.
(PDF)

Table S6 Genes down-regulated in *sd::Gal4>UAS::RpL12-Myc vs sd::Gal4/+*. FC: Fold Change.
(PDF)

Table S7 Genes de-regulated in sd::Gal4>UAS::FH-cortoCD *vs sd::Gal4/+* and *sd::Gal4>UAS::RpL12-Myc vs sd::Gal4/+*.
(PDF)

Table S8 Ontology of genes up-regulated in *sd::Gal4>UAS::FH-cortoCD vs sd::Gal4/+*.
(PDF)

Table S9 Ontology of genes down-regulated in *sd::Gal4>UAS::FH-cortoCD vs sd::Gal4/+*.
(PDF)

Table S10 Ontology of genes up-regulated in *sd::Gal4>UAS:RpL12-Myc vs sd::Gal4/+*.
(PDF)

Table S11 Ontology of genes down-regulated in *sd::Gal4>UAS::RpL12-Myc vs sd::Gal4/+*.
(PDF)

Table S12 Primers used for constructs and qPCRs. Note that an ATG and two nuclear localization signals (underlined) were added to the forward and reverse primers of *cortoCD*, respectively. For *RpL12* mutants, the mutated triplet is underlined.
(PDF)

Table S13 Sequences of the RpL12 peptides used in this study.
(PDF)

Table S14 Read count data for sequencing experiments. For each edge of Paired-End sequenced samples (PE1 and PE2), the number of raw reads (raw), reads passing quality control filters (QC filter), uniq alignments (unig align) and the number of alignment used to estimate transcript abundance (transcript) are given.
(PDF)

Text S1 Supporting methods.
(DOC)
