# Supplementary figures and images for: New Partners in Regulation of Gene Expression: The Enhancer of Trithorax and Polycomb Corto Interacts with Methylated Ribosomal Protein L12 Via Its Chromodomain
Source: PLoS Genet. 2012 Oct 11;8(10):e1003006. doi: 10.1371/journal.pgen.1003006 (PMC3469418; doi:10.1371/journal.pgen.1003006)

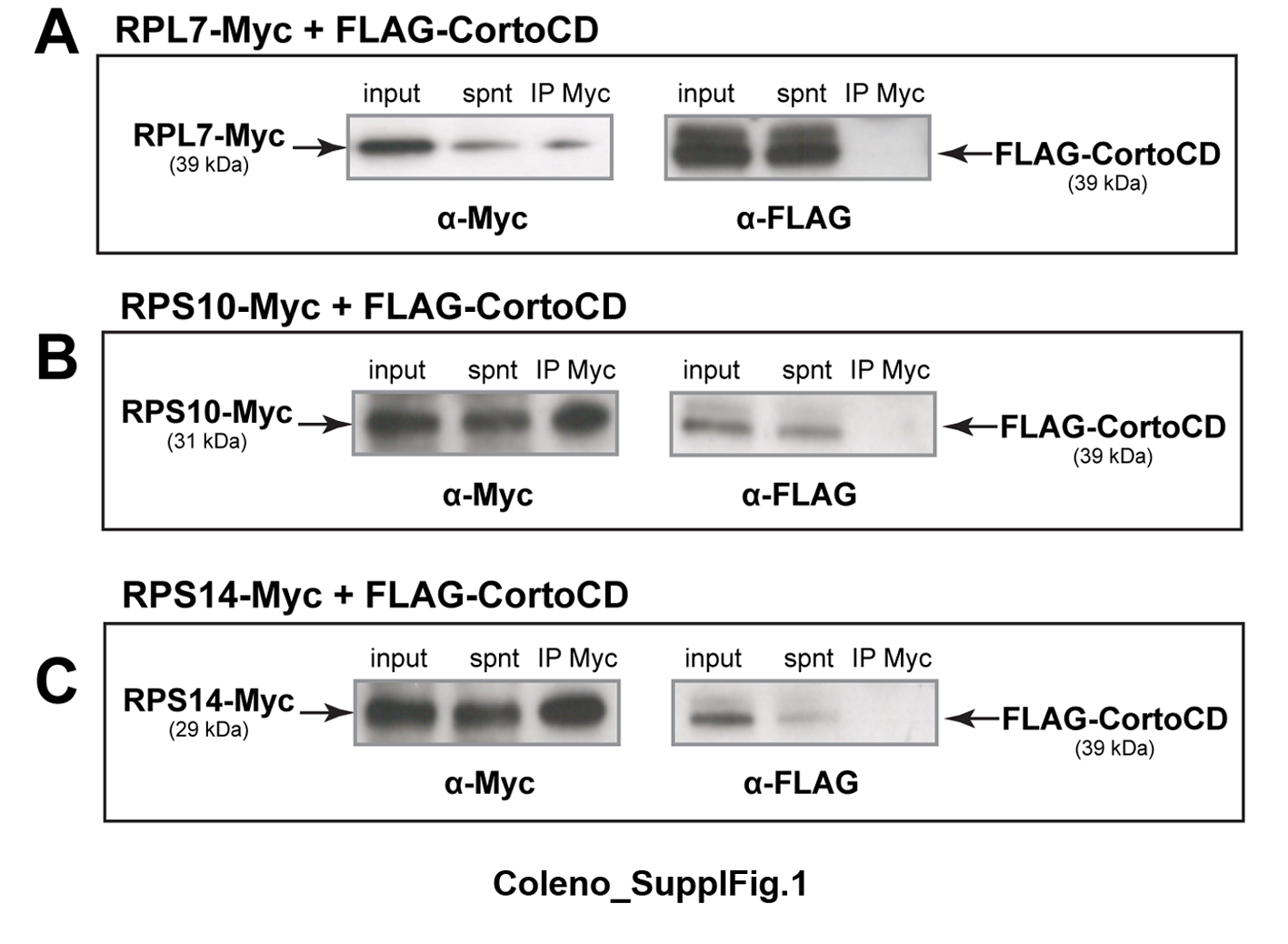

Supplement: Figure S1 — CortoCD does not co-immunoprecipitate with nuclear ribosomal proteins RPL7, RPS10 and RPS14. (A) Transfection of FLAG-CortoCD and RPL7-Myc in S2 cells. (B) Transfection of FLAG-CortoCD and RPS10-Myc in S2 cells. (C) Transfection of FLAG-CortoCD and RPS14-Myc in S2 cells. Immunoprecipitations were performed with anti-Myc and revealed by Western blot with either anti-Myc (α-Myc) or anti-FLAG (α-FLAG). Spnt: supernatant, IP: immunoprecipitation. (TIF) [file pgen.1003006.s001.tif]

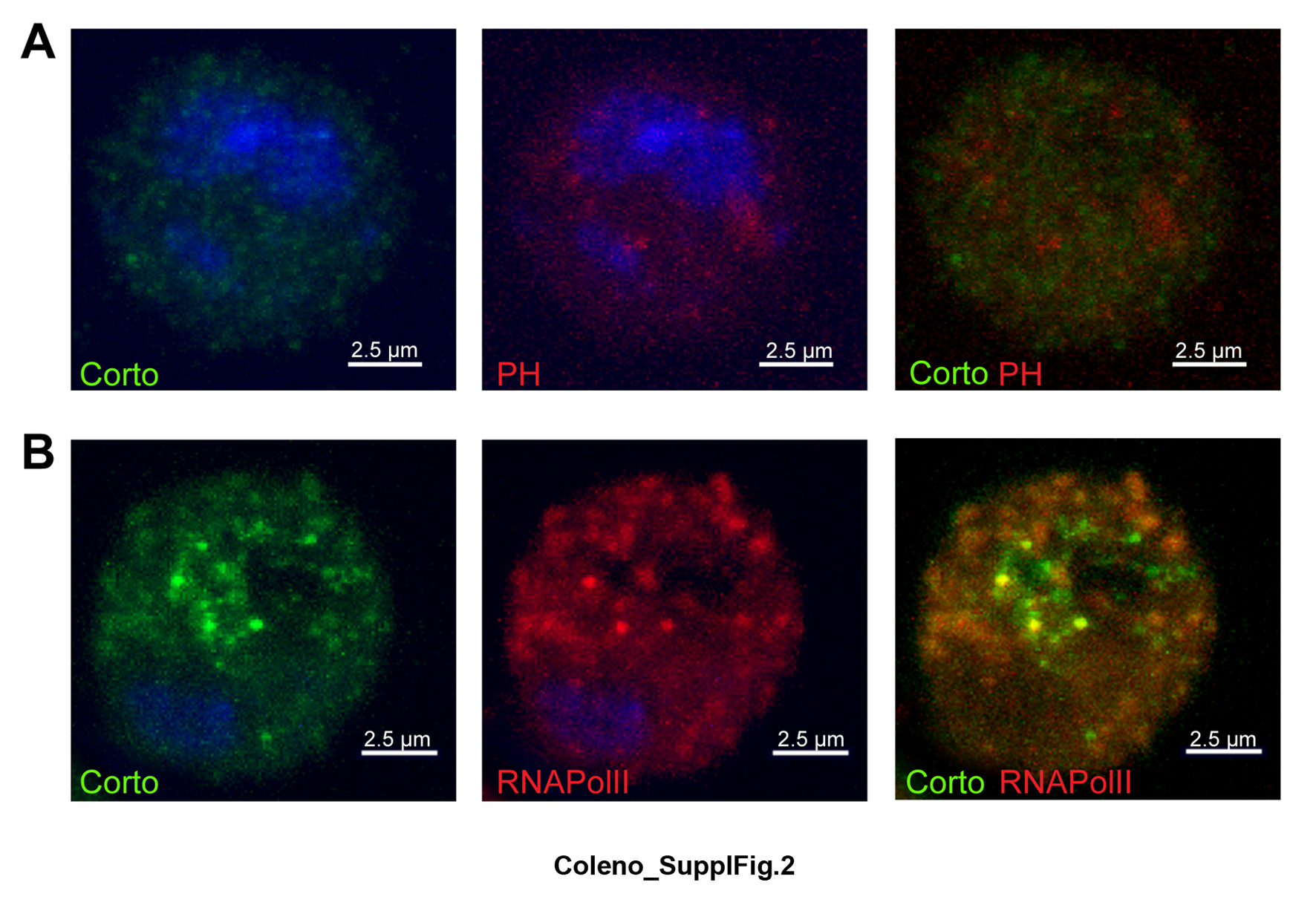

Supplement: Figure S2 — Corto overlaps with transcriptional factories. (A) Immunostaining of S2 cells with anti-Corto (green) and anti-PH (red) antibodies showing that Corto bodies and Polycomb bodies did not overlap. Blue: DAPI. Close-up of a nucleus. (B) Immunostaining of S2 cells with anti-Corto (green) and anti-RNAPolII (red) antibodies showing that Corto bodies and transcriptional factories overlapped. Blue: DAPI. Close-up of a nucleus. (TIF) [file pgen.1003006.s002.tif]

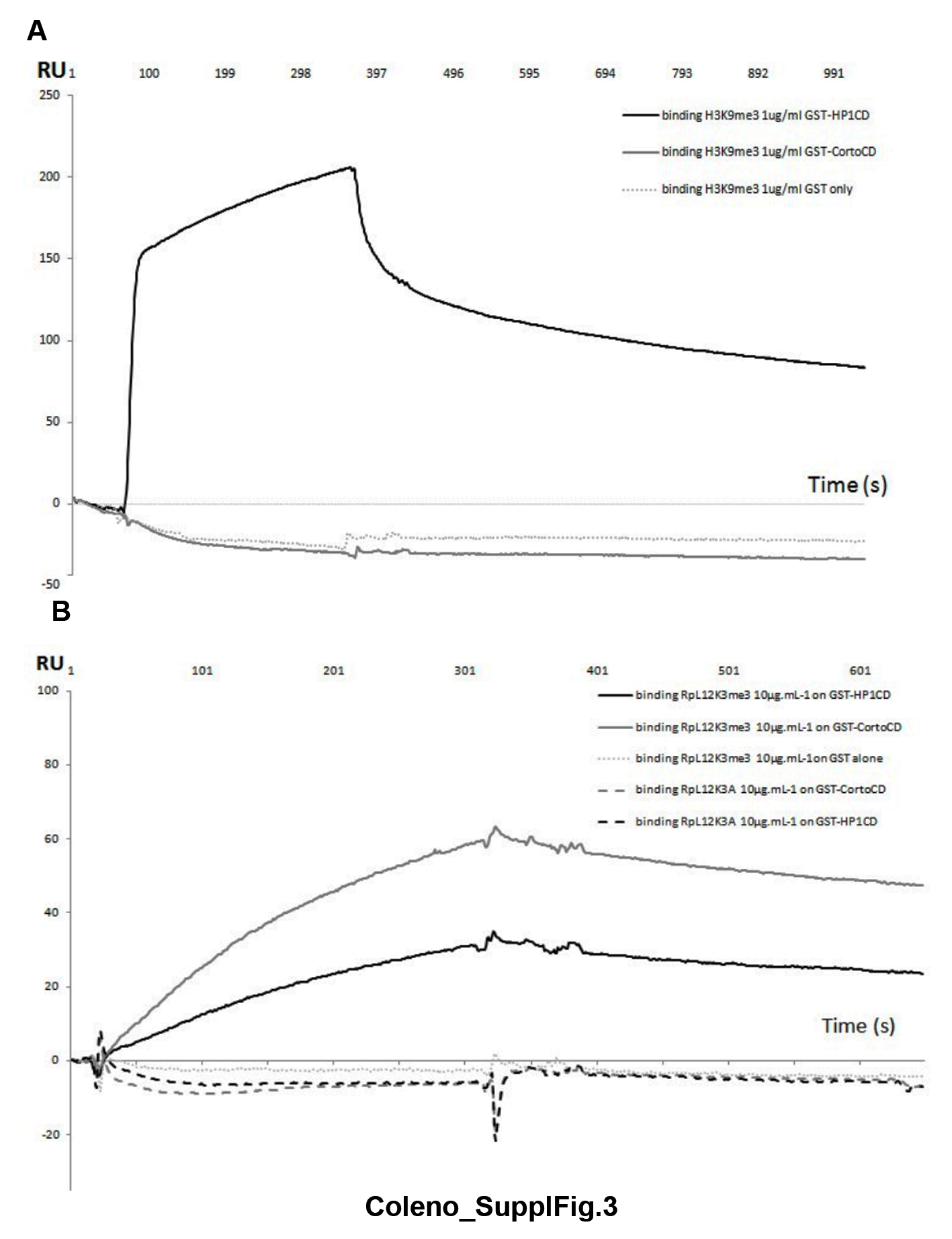

Supplement: Figure S3 — Real-time interaction binding assays. (A) Biacore sensorgram showing binding of H3K9me3 peptide (1 µg.mL-1) to GST-HP1CD, GST-CortoCD or GST. Binding (Y-axis, Response) is expressed in Resonance Unit (RU). Note that H3K9me3 bound GST-HP1CD as expected but did not bind GST-CortoCD or GST. (B) Biacore sensorgram showing binding of RpL12K3me3 or RpL12K3A peptides (10 µg.mL-1) to GST-HP1CD, GST-CortoCD or GST. Binding (Y-axis, Response) is expressed in resonance unit (RU). (TIF) [file pgen.1003006.s003.tif]

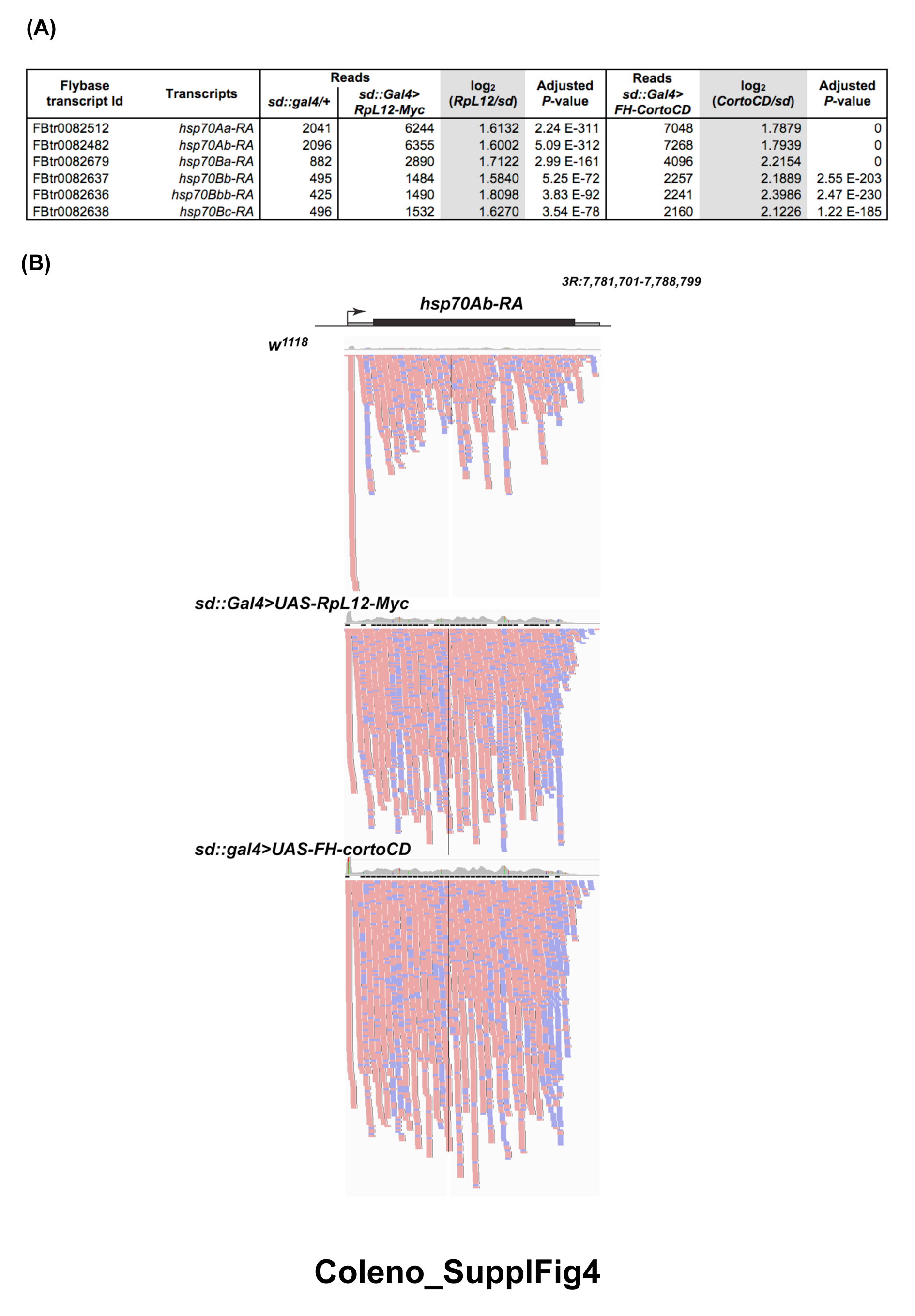

Supplement: Figure S4 — hsp70 genes are upregulated when either RpL12-Myc or FH-CortoCD are expressed in wing imaginal discs. (A) Read count data and log2 fold change (FC) analysis relative to sd::Gal4/+ for each hsp70 transcript. (B) IGV (Integrative Genomics Viewer) screenshot showing read alignments along the hsp70Ab locus (3R:7,781,701–7,788,799) in control w1118, sd::Gal4>UAS-RpL12-Myc and sd::Gal4>UAS-FH-cortoCD wing imaginal discs. Sense reads appear in pink and reverse reads in blue. (TIF) [file pgen.1003006.s004.tif]
