# Supplementary material for: New Partners in Regulation of Gene Expression: The Enhancer of Trithorax and Polycomb Corto Interacts with Methylated Ribosomal Protein L12 Via Its Chromodomain
Source: PLoS Genet. 2012 Oct 11;8(10):e1003006. doi: 10.1371/journal.pgen.1003006 (PMC3469418; doi:10.1371/journal.pgen.1003006)
Supplement: Table S1 — Phenotypes of flies overexpressing cortoCD using ubiquitous Gal4 drivers. Three different insertions of the cortoCD transgene (named 231, 41 and 45) and one full-length corto transgene were analysed. nd: not determined. *: difference with driver alone highly significant (p<1.5 10−10, T Test). **: difference with driver alone significant (p = 0.03, Fisher's exact test); ***: difference with driver alone highly significant (p<0.005, Fisher's exact test). (PDF) [file pgen.1003006.s005.pdf]

Genotype	Flies observed	Lethality (%)	Duplicated macro-chaetae (%)	Aristapedia (%)	Sex comb teeth (n = number of male first legs observed)	Rotated genitalia (%) (n = number of males observed)
Act::Gal4>UAS::FH-cortoCD-231	73	63.0	32.8***	35.6***	8.8 ± 0.8* (n=46)	nd
Act::Gal4>UAS::FH-cortoCD-41	143	79.0	30.0***	34.3***	9.2 ± 1.1* (n=35)	nd
Act::Gal4>UAS::FH-cortoCD-45	67	76.0	31.3***	43.3***	8.6 ± 0.5* (n=10)	nd
Act::Gal4/+	164	22.5	3.6	0.6	10.8 ± 0.7 (n=41)	nd
da::Gal4>UAS::FH-cortoCD-231	93	91.0	9.7***	38.7***	8.8 ± 0.8* (n=54)	55.0*** (n=45)
da::Gal4>UAS::FH-cortoCD-41	34	nd	8.8**	67.6***	8.7 ± 0.9* (n=30)	59.0*** (n=16)
da::Gal4>UAS::FH-cortoCD-45	0	100.0	-	-	-	-
da::Gal4/+	202	19.0	2.0	0	11.2 ± 0.8 (n=80)	0
arm::Gal4>UAS::corto	210	58.0	8.1	15.0***	10.9 ± 0.8 (n=58)	19.4*** (n=110)
arm::Gal4/+	200	5.0	6.5	0	11.2 ± 0.9 (n=50)	0 (n=100)

Table S1
