# Supplementary material for: New Partners in Regulation of Gene Expression: The Enhancer of Trithorax and Polycomb Corto Interacts with Methylated Ribosomal Protein L12 Via Its Chromodomain
Source: PLoS Genet. 2012 Oct 11;8(10):e1003006. doi: 10.1371/journal.pgen.1003006 (PMC3469418; doi:10.1371/journal.pgen.1003006)
Supplement: Table S2 — Mass spectrometry analysis of peptides pulled down by CortoCD. Four bands (P30, P21, P20 and P15) were excised from the gel (see Figure 4) and analyzed by mass spectrometry. (PDF) [file pgen.1003006.s006.pdf]

Table S2

Band	Protein	CG	MW (Da)	Score	Peptides	Coverage %	Sequences
P30	RpL7	CG4897-PA	29534	107	2	10	R.QRVPITDNFVIER.K<br>R.IAEPYITWGYPNLK.S
P21	RpS11	CG8857	18101	38	2	9	K.QFGVNLNR.K<br>R.DYLHFVR.K
P20	RpS10	CG14206-PC	17867	42	1	8	K.GDVGPGAGEVEFR.G
	RpL12	CG3195-PA	17585	56	2	14	K.IGPLGLSPK.K<br>R.CVGGEVGATSSLAPK.I + carbamidomethyl (C)
	RpL27	CG4759-PA	15893	79	2	15	R.YTAHDISFEK.L<br>K.SLNYNHLMPTR.Y + oxidation (M)
P15	RpS14	CG1524-PB	16255	82	3	17	K.EEVQVQLGPQVR.D<br>R.IEDVTPIPSDSTR.R<br>R.IEDVTPIPSDSTRR.K
