# Supplementary material for: New Partners in Regulation of Gene Expression: The Enhancer of Trithorax and Polycomb Corto Interacts with Methylated Ribosomal Protein L12 Via Its Chromodomain
Source: PLoS Genet. 2012 Oct 11;8(10):e1003006. doi: 10.1371/journal.pgen.1003006 (PMC3469418; doi:10.1371/journal.pgen.1003006)
Supplement: Table S3 — Genes up-regulated in sd::Gal4>UAS::FH-cortoCD vs sd::Gal4/+. FC: Fold Change. (PDF) [file pgen.1003006.s007.pdf]

Table S3

Flybase Gene Symbol	Flybase ID Genes	Flybase ID Transcripts	Number of read *sd::Gal4>UAS::FH-cortoCD*	Number of read *sd::Gal4/+*	Adjusted *P*-value	log_2(FC)
RpS10b	FBgn0261593	FBtr0074731,FBtr0074732,FBtr0308192	44201	22084	0	1,00108
pck	FBgn0013720	FBtr0100198	2899	1448	5,81826E-60	1,00149
CG10527	FBgn0034583	FBtr0071541	1920	959	5,93532E-40	1,00150
CG32212	FBgn0052212	FBtr0074932	2549	1273	7,35002E-53	1,00170
cic	FBgn0262582	FBtr0305026,FBtr0305027,FBtr0305028, FBtr0305029,FBtr0305030,FBtr0305031	8336	4162	5,78156E-167	1,00208
pck	FBgn0013720	FBtr0070255,FBtr0070256	2899	1446	3,26466E-60	1,00349
Cchl	FBgn0038925	FBtr0084185	1548	772	1,50462E-32	1,00373
Vps28	FBgn0021814	FBtr0088825	1470	733	5,79182E-31	1,00393
sli	FBgn0003425	FBtr0087302,FBtr0087303,FBtr0087304	953	475	2,24757E-20	1,00455
SmF	FBgn0000426	FBtr0088010	2456	1224	2,70685E-51	1,00471
Taf10	FBgn0028398	FBtr0077717	1503	749	1,01672E-31	1,00481
RpL3	FBgn0020910	FBtr0082347,FBtr0082346,FBtr0302586	4538	2260	2,31682E-94	1,00573
CG4036	FBgn0032149	FBtr0303447,FBtr0303448	952	474	1,66979E-20	1,00607
Updo	FBgn0033428	FBtr0088524	2632	1309	2,64901E-55	1,00769
pcx	FBgn0003048	FBtr0070395	5417	2692	2,81048E-113	1,00882
mRpL18	FBgn0026741	FBtr0087852	872	433	5,54362E-19	1,00996
rhea	FBgn0260442	FBtr0076627,FBtr0305210,FBtr0305211, FBtr0305212	7452	3695	1,07596E-152	1,01205
CG18343	FBgn0033683	FBtr0087982	1309	649	3,57281E-28	1,01217
CG4036	FBgn0032149	FBtr0079870	964	477	4,40730E-21	1,01504
CG13349	FBgn0033886	FBtr0087610,FBtr0087611,FBtr0114493,F Btr0114494	3145	1556	4,99432E-67	1,01522
CG30410	FBgn0050410	FBtr0301852	3657	1809	6,96792E-78	1,01547
CG10664	FBgn0032833	FBtr0081300,FBtr0081301	5363	2652	5,97598E-114	1,01596
CG13349	FBgn0033886	FBtr0114495	3147	1556	3,19282E-67	1,01614
CG8891	FBgn0031663	FBtr0079048	1568	775	6,70087E-34	1,01666
RpS30	FBgn0038834	FBtr0083969,FBtr0083970	20690	10221	0	1,01740
hang	FBgn0026575	FBtr0074276,FBtr0074277	4445	2195	4,82424E-95	1,01796
CG1707	FBgn0033162	FBtr0089013	4362	2152	1,39310E-93	1,01931
Cpr49Ah	FBgn0033731	FBtr0087924	987	486	5,76268E-22	1,02209
CG34347	FBgn0085376	FBtr0290046	1020	502	1,01302E-22	1,02281
Rpb12	FBgn0262954	FBtr0112379	1685	828	4,30839E-37	1,02505
CG15012	FBgn0035528	FBtr0073240	810	398	3,06267E-18	1,02515
CG9034	FBgn0040931	FBtr0071343	922	453	1,10836E-20	1,02526
PDCD-5	FBgn0036580	FBtr0075483	3710	1822	6,87942E-81	1,02590
larp	FBgn0261618	FBtr0301180,FBtr0306389,FBtr0306390	1908	937	5,27586E-42	1,02594
mRpS11	FBgn0038474	FBtr0089894	1001	491	1,71900E-22	1,02765
Prosbeta1	FBgn0010590	FBtr0087262	6364	3114	9,22842E-140	1,03116
rut	FBgn0003301	FBtr0073992,FBtr0305581	3391	1659	6,95553E-75	1,03140
CG15012	FBgn0035528	FBtr0300776	808	395	1,54694E-18	1,03250
bou	FBgn0261284	FBtr0071004	2997	1465	1,94792E-66	1,03262
obst-B	FBgn0027600	FBtr0079934	3357	1640	2,02164E-74	1,03348
Rbcn-3A	FBgn0023458	FBtr0303341	10392	5071	1,08035E-223	1,03513
mei-P26	FBgn0026206	FBtr0071303	4958	2419	5,24173E-110	1,03535
CG30423	FBgn0050423	FBtr0300122	3189	1555	3,45868E-71	1,03619
CG30185	FBgn0050185	FBtr0072060	1526	744	1,62240E-34	1,03638
Ten-a	FBgn0259240	FBtr0300201,FBtr0300202,FBtr0300203, FBtr0300204,FBtr0299858,FBtr0299859, FBtr0299860,FBtr0308221,FBtr0308222, FBtr0308223	22588	11010	0	1,03674
m2	FBgn0002592	FBtr0084981	3400	1657	6,12686E-76	1,03696
Arp11	FBgn0031050	FBtr0074760	1705	830	1,31642E-38	1,03859
Spase22-23	FBgn0039172	FBtr0305044	10017	4874	1,47481E-217	1,03927
CG6878	FBgn0036488	FBtr0075643	1252	609	1,10402E-28	1,03972
ATPsyn-Cf6	FBgn0016119	FBtr0084432,FBtr0305982	3169	1541	2,33932E-71	1,04016
RpS13	FBgn0010265	FBtr0079724,FBtr0100541	79412	38614	0	1,04023
CG5548	FBgn0030605	FBtr0073960	1533	745	5,57960E-35	1,04105
dom	FBgn0020306	FBtr0071602,FBtr0071603,FBtr0071604, FBtr0301350	4443	2159	5,51313E-100	1,04117
CG6746	FBgn0032394	FBtr0080313	1774	862	2,01221E-40	1,04125
fwd	FBgn0004373	FBtr0072516	826	401	2,49966E-19	1,04254
Bruce	FBgn0037808	FBtr0082239	16056	7785	0	1,04434
CG11999	FBgn0037312	FBtr0078807	1977	957	2,06572E-45	1,04672
l(2)37Cg	FBgn0086447	FBtr0081161	798	386	6,16178E-19	1,04779
CG5446	FBgn0032429	FBtr0080388	3732	1804	1,77975E-85	1,04875
Tpi	FBgn0086355	FBtr0085584	4221	2040	1,30830E-96	1,04902
tweek	FBgn0261671	FBtr0304927,FBtr0304928,FBtr0304929, FBtr0304930	6595	3187	1,37337E-150	1,04917
Act5C	FBgn0000042	FBtr0070823	7261	3508	2,57214E-161	1,04952
Act57B	FBgn0000044	FBtr0300946	3932	1899	2,85891E-90	1,05002
Act57B	FBgn0000044	FBtr0071519	3931	1898	2,63649E-90	1,05042
Spase12	FBgn0040623	FBtr0085435	1830	883	1,61959E-42	1,05136
CR41548	FBgn0085802	FBtr0114256	2815	1357	3,44527E-65	1,05271
CG2862	FBgn0031459	FBtr0077718	5162	2486	2,82840E-119	1,05410
sni	FBgn0030026	FBtr0071183	840	404	3,97114E-20	1,05603
Dhc64C	FBgn0261797	FBtr0273370,FBtr0073359	26306	12641	0	1,05728
CG13185	FBgn0033661	FBtr0113071	5574	2677	7,47688E-130	1,05810
CG32276	FBgn0047135	FBtr0073107	1048	503	4,45874E-25	1,05901
CG13751	FBgn0033340	FBtr0088644	1342	644	6,61190E-32	1,05925
Nxt1	FBgn0028411	FBtr0072241	1041	499	5,13712E-25	1,06086
CG9603	FBgn0040529	FBtr0081855,FBtr0305680	7612	3647	2,24491E-173	1,06157
Nurf-38	FBgn0016687	FBtr0305985	2779	1331	1,08241E-65	1,06206
CG32276	FBgn0047135	FBtr0073106	1086	520	3,29877E-26	1,06244
PHGPx	FBgn0035438	FBtr0073066,FBtr0073067,FBtr0073068	5639	2700	1,31506E-132	1,06248
CG11505	FBgn0035424	FBtr0073045,FBtr0073046,FBtr0308061	7684	3679	2,31078E-175	1,06254
CR40959	FBgn0085777	FBtr0114228	4012	1920	1,30836E-94	1,06322
CG3621	FBgn0025839	FBtr0070386	1114	533	7,17632E-27	1,06354
CG13185	FBgn0033661	FBtr0113072	5755	2748	2,00142E-136	1,06643
Tpi	FBgn0086355	FBtr0085582,FBtr0085583	4179	1994	1,87672E-99	1,06749
CR40621	FBgn0085757	FBtr0114207	5659	2700	1,86684E-134	1,06759
nocte	FBgn0261710	FBtr0071461	2003	955	3,18899E-48	1,06859
tutl	FBgn0010473	FBtr0089271,FBtr0089269,FBtr0089270, FBtr0301863,FBtr0301864	1544	736	2,56970E-37	1,06890

CG13630	FBgn0039219	FBtr0084745	2361	1123	4,69705E-57	**1,07204**
CG6891	FBgn0030955	FBtr0074631	14114	6711	0	**1,07253**
br	FBgn0000210	FBtr0300430	3537	1680	1,75454E-85	**1,07406**
CG8206	FBgn0030679	FBtr0074085	906	430	1,41203E-22	**1,07517**
Pros29	FBgn0261394	FBtr0071522	9558	4531	1,91552E-224	**1,07688**
CG5703	FBgn0030853	FBtr0074452	2013	953	1,98151E-49	**1,07880**
CG5903	FBgn0038400	FBtr0083281	5124	2423	3,85832E-125	**1,08048**
RpS5a	FBgn0002590	FBtr0074406	78164	36960	0	**1,08054**
trx	FBgn0003862	FBtr0082950,FBtr0082949,FBtr0082947, FBtr0082948,FBtr0100277	13801	6521	0	**1,08161**
CG34439	FBgn0085468	FBtr0112740,FBtr0112741	999	472	4,58604E-25	**1,08170**
CG9669	FBgn0036667	FBtr0075339,FBtr0305272	1488	703	5,63998E-37	**1,08178**
RpS19a	FBgn0010412	FBtr0074312,FBtr0074313,FBtr0074311	75397	35551	0	**1,08462**
CG11753	FBgn0037603	FBtr0081879	895	422	1,16551E-22	**1,08464**
malpha	FBgn0002732	FBtr0084956	5935	2798	4,30082E-146	**1,08485**
RFeSP	FBgn0021906	FBtr0077914,FBtr0077915	722	340	1,55017E-18	**1,08646**
CG15237	FBgn0033104	FBtr0086212	1143	538	5,88978E-29	**1,08715**
Spase22-23	FBgn0039172	FBtr0084564	9906	4659	5,95698E-238	**1,08828**
Tina-1	FBgn0035083	FBtr0072411	4210	1978	6,35904E-105	**1,08978**
		FBtr0273184	2365	1111	2,86998E-59	**1,08998**
Roc1a	FBgn0025638	FBtr0070122	2363	1110	3,35908E-59	**1,09006**
CG11267	FBgn0036334	FBtr0075875	6335	2969	1,32459E-158	**1,09337**
RpS6	FBgn0261592	FBtr0071135,FBtr0071136	108886	50993	0	**1,09445**
CG12203	FBgn0031021	FBtr0074712	1921	899	7,57751E-49	**1,09546**
CG17680	FBgn0062440	FBtr0086781	1302	609	2,11499E-33	**1,09622**
eIF-3p40	FBgn0022023	FBtr0079030,FBtr0079031	19533	9133	0	**1,09675**
Smr	FBgn0024308	FBtr0073706,FBtr0073707,FBtr0073708	21018	9819	0	**1,09798**
RpS9	FBgn0010408	FBtr0076424	15140	7070	0	**1,09858**
CG3500	FBgn0034849	FBtr0072038	892	416	2,62942E-23	**1,10046**
CG17202	FBgn0038043	FBtr0082587	2685	1252	1,25176E-68	**1,10069**
CG2812	FBgn0034931	FBtr0072140	1622	756	8,77155E-42	**1,10132**
CG5569	FBgn0034919	FBtr0072128	717	334	5,63781E-19	**1,10213**
Neb-cGP	FBgn0083167	FBtr0071449	5476	2550	9,09946E-140	**1,10263**
CG31548	FBgn0051548	FBtr0078736	1164	542	2,20963E-30	**1,10273**
CG9240	FBgn0030669	FBtr0074060	1113	517	3,07163E-29	**1,10622**
N	FBgn0004647	FBtr0070507,FBtr0304659	44071	20453	0	**1,10752**
Rpb11	FBgn0032634	FBtr0081011	1341	621	2,24716E-35	**1,11064**
RpL14	FBgn0017579	FBtr0076633	42610	19705	0	**1,11263**
Got2	FBgn0001125	FBtr0077867,FBtr0077868	911	421	2,18921E-24	**1,11363**
RpS4	FBgn0011284	FBtr0075884,FBtr0075885	104248	48111	0	**1,11558**
SmD2	FBgn0261789	FBtr0081737	5038	2324	3,76841E-132	**1,11624**
mRpL22	FBgn0030786	FBtr0074330	1702	785	3,98575E-45	**1,11647**
Arf79F	FBgn0010348	FBtr0078574	1835	846	1,07618E-48	**1,11705**
CG2862	FBgn0031459	FBtr0077719	5573	2569	2,08954E-146	**1,11725**
RpS9	FBgn0010408	FBtr0076423,FBtr0076425	14743	6794	0	**1,11770**
skpA	FBgn0025637	FBtr0070124	1637	753	7,80107E-44	**1,12033**
SdhC	FBgn0037873	FBtr0082372	2140	983	2,89397E-57	**1,12235**
CG3560	FBgn0030733	FBtr0074216	2826	1298	2,27455E-75	**1,12247**
poe	FBgn0011230	FBtr0079545	26949	12354	0	**1,12525**
CG42377	FBgn0259744	FBtr0299980	660	302	1,97716E-18	**1,12792**
RpS26	FBgn0261597	FBtr0081090,FBtr0081089	18376	8397	0	**1,12988**
CR31144	FBgn0051144	FBtr0303864	32532	14849	0	**1,13149**
RpL3	FBgn0020910	FBtr0082347	32534	14850	0	**1,13149**
Trxr-1	FBgn0020653	FBtr0071168	1028	468	1,23313E-28	**1,13526**
B52	FBgn0004587	FBtr0082804,FBtr0082806	3909	1779	1,53914E-106	**1,13573**
RpL9	FBgn0015756	FBtr0080163,FBtr0080164	57751	26270	0	**1,13643**
CG5941	FBgn0029833	FBtr0070867,FBtr0305344	1761	801	1,75916E-48	**1,13652**
mRpL27	FBgn0053002	FBtr0077451	975	443	2,81006E-27	**1,13810**
14-3-3zeta	FBgn0004907	FBtr0088412	773	351	7,68583E-22	**1,13900**
cic	FBgn0262582	FBtr0305029	7999	3622	2,10832E-213	**1,14303**
mRpL55	FBgn0038678	FBtr0083754	815	369	3,64282E-23	**1,14318**
B52	FBgn0004587	FBtr0082801,FBtr0082802,FBtr0082803, FBtr0300586,FBtr0300588,FBtr0300589, FBtr0308195,FBtr0308196,FBtr0308197	3866	1747	1,82879E-107	**1,14596**
CG11015	FBgn0031830	FBtr0079288	6391	2887	2,08576E-177	**1,14647**
wb	FBgn0261563	FBtr0302731,FBtr0305063	658	297	4,47687E-19	**1,14762**
CG13993	FBgn0031776	FBtr0079191	2756	1243	3,04272E-77	**1,14875**
Cypl	FBgn0035141	FBtr0072564	1003	452	1,17629E-28	**1,14993**
CG11873	FBgn0039633	FBtr0085365	14712	6623	0	**1,15144**
mRpS16	FBgn0033907	FBtr0087578	1069	481	1,29678E-30	**1,15215**
CG11699	FBgn0030311	FBtr0073510	1954	879	2,13060E-55	**1,15250**
Cctgamma	FBgn0015019	FBtr0083338	816	367	1,40345E-23	**1,15279**
CG9603	FBgn0040529	FBtr0081855	2274	1021	1,13655E-64	**1,15525**
Elongin-C	FBgn0023211	FBtr0086541	987	442	1,21186E-28	**1,15900**
TfIIA-S	FBgn0013347	FBtr0084526	5528	2474	2,41248E-157	**1,15991**
RpII15	FBgn0004855	FBtr0082925	2492	1114	1,60129E-71	**1,16155**
CG7603	FBgn0036726	FBtr0075220	1772	792	3,42111E-51	**1,16181**
CG30499	FBgn0050499	FBtr0088954	2692	1203	2,52268E-77	**1,16204**
osa	FBgn0261885	FBtr0301487,FBtr0089582,FBtr0089581	1092	487	5,02737E-32	**1,16498**
Cpr66D	FBgn0052029	FBtr0076567	747	333	3,53073E-22	**1,16559**
larp	FBgn0261618	FBtr0301180	633	282	5,87390E-19	**1,16651**
RpS3	FBgn0002622	FBtr0084410	128804	57350	0	**1,16731**
sdk	FBgn0021764	FBtr0302259	703	313	6,18479E-21	**1,16736**
CG12384	FBgn0033624	FBtr0304698	701	312	5,52714E-21	**1,16787**
asrij	FBgn0034793	FBtr0071902	1329	591	4,60990E-39	**1,16911**
Actn	FBgn0000667	FBtr0070345,FBtr0070343,FBtr0070344	758	337	1,13129E-22	**1,16945**
Elongin-C	FBgn0023211	FBtr0086540	937	416	6,68169E-28	**1,17147**
CG9674	FBgn0036663	FBtr0075341,FBtr0075344,FBtr0305915, FBtr0305916	4623	2052	1,26264E-134	**1,17180**
mRpL49	FBgn0030433	FBtr0073691	762	338	7,53277E-23	**1,17277**
CG3566	FBgn0029854	FBtr0089869,FBtr0089870,FBtr0305177	787	349	1,36289E-23	**1,17314**
CG3731	FBgn0038271	FBtr0083001	774	343	2,64726E-23	**1,17412**
RpL10Ab	FBgn0036213	FBtr0302135	5163	2286	2,84297E-151	**1,17538**
Tao-1	FBgn0031030	FBtr0074771,FBtr0303999	1108	490	3,95967E-33	**1,17710**
tyf	FBgn0026083	FBtr0070643,FBtr0300931,FBtr0305180, FBtr0305181,FBtr0305182	1771	783	1,29241E-52	**1,17748**
RpS15	FBgn0034138	FBtr0087124	659	291	3,95890E-20	**1,17926**
HP4	FBgn0035829	FBtr0076765	2170	958	1,82191E-64	**1,17960**

CG2200	FBgn0030447	FBtr0073721	1236	545	3,38571E-37	**1,18135**
CG3446	FBgn0029868	FBtr0070924	3564	1571	6,96157E-106	**1,18181**
CG34422	FBgn0085451	FBtr0112723	2761	1217	4,05808E-82	**1,18186**
HP4	FBgn0035829	FBtr0076764	2169	956	1,14015E-64	**1,18195**
Arf79F	FBgn0010348	FBtr0078573	1807	796	3,55313E-54	**1,18276**
GstD1	FBgn0001149	FBtr0082607	24617	10835	0	**1,18396**
CG8036	FBgn0037607	FBtr0081881	4979	2191	2,91866E-148	**1,18427**
CG5261	FBgn0031912	FBtr0079444	909	400	9,98471E-28	**1,18428**
CG12432	FBgn0030843	FBtr0074433	1032	454	2,22501E-31	**1,18468**
LRP1	FBgn0053087	FBtr0088791	11637	5118	0	**1,18507**
CG8036	FBgn0037607	FBtr0081882	4979	2188	9,60122E-149	**1,18624**
Got2	FBgn0001125	FBtr0100359	1712	750	4,28699E-52	**1,19072**
CG3609	FBgn0031418	FBtr0077791,FBtr0303027	1532	671	1,22177E-46	**1,19103**
CG30415	FBgn0250838	FBtr0072030,FBtr0072031	2360	1031	6,25220E-72	**1,19474**
RpL37A	FBgn0261608	FBtr0302570,FBtr0079016	10786	4711	2,3426E-315	**1,19505**
awd	FBgn0000150	FBtr0085864	56390	24628	0	**1,19514**
RpL37A	FBgn0261608	FBtr0079017	10833	4731	8,1482E-317	**1,19522**
l(1)G0255	FBgn0028336	FBtr0070953	733	320	5,31699E-23	**1,19574**
vfl	FBgn0259789	FBtr0307536,FBtr0307537	15475	6742	0	**1,19869**
RpL30	FBgn0086710	FBtr0302491,FBtr0081181	7996	3480	5,95090E-236	**1,20019**
mRpL48	FBgn0031357	FBtr0077882	669	291	3,72146E-21	**1,20099**
sea	FBgn0037912	FBtr0100464	6494	2819	5,92255E-200	**1,20392**
fabp	FBgn0037913	FBtr0100320,FBtr0100321,FBtr0100323	6494	2819	5,92255E-200	**1,20392**
olf186-F	FBgn0041585	FBtr0086796,FBtr0086797	694	301	4,62433E-22	**1,20517**
RpS7	FBgn0039757	FBtr0089425,FBtr0089422,FBtr0089423	69140	29982	0	**1,20542**
Aldh-III	FBgn0010548	FBtr0300477	595	258	4,85430E-19	**1,20552**
RpS15	FBgn0034138	FBtr0087123	677	293	9,35480E-22	**1,20826**
p16-ARC	FBgn0031437	FBtr0077694	3717	1608	1,12935E-115	**1,20887**
CR40728	FBgn0085769	FBtr0114219	3815	1648	3,30772E-119	**1,21097**
vsg	FBgn0045823	FBtr0076399,FBtr0076401	1503	649	1,79102E-47	**1,21155**
mRpL42	FBgn0033480	FBtr0088403	856	369	1,77519E-27	**1,21399**
mRpL42	FBgn0033480	FBtr0305670	856	369	1,77519E-27	**1,21399**
mRpL42	FBgn0033480	FBtr0304041	856	369	1,77519E-27	**1,21399**
Arc-p20	FBgn0031781	FBtr0079200	2176	935	3,68671E-69	**1,21864**
stv	FBgn0086708	FBtr0075806,FBtr0075807,FBtr0075809	2559	1098	1,75260E-81	**1,22070**
shot	FBgn0013733	FBtr0301964,FBtr0087621,FBtr0087617,FBtr0087618,FBtr0087619,FBtr0087620,FBtr0087616,FBtr0273222,FBtr0273223,FBtr0273224,FBtr0273225,FBtr0273226,FBtr0301591,FBtr0301592,FBtr0301593,FBtr0304847,FBtr0304848,FBtr0304849,FBtr0304850,FBtr0304851,FBtr0304852,FBtr0304853	15882	6807	0	**1,22230**
stv	FBgn0086708	FBtr0075808,FBtr0113425	2770	1187	1,88171E-88	**1,22257**
GstD1	FBgn0001149	FBtr0100410	24045	10303	0	**1,22267**
betaTub56D	FBgn0003887	FBtr0086536	24093	10277	0	**1,22919**
CG10418	FBgn0036277	FBtr0075954	1130	482	5,34231E-37	**1,22922**
CG8191	FBgn0030675	FBtr0074083	952	406	2,84011E-31	**1,22948**
HSPC300	FBgn0061198	FBtr0072194	1456	619	8,38802E-48	**1,23400**
mRpL51	FBgn0032053	FBtr0079696	1482	630	1,16560E-48	**1,23412**
zormin	FBgn0052311	FBtr0089864,FBtr0110856,FBtr0110857,FBtr0110858	3974	1689	3,42259E-129	**1,23442**
w	FBgn0003996	FBtr0070490	18794	7981	0	**1,23563**
CG11438	FBgn0037164	FBtr0078504	2040	865	4,89774E-67	**1,23780**
RpL13	FBgn0011272	FBtr0079888,FBtr0100164	70213	29771	0	**1,23783**
br	FBgn0000210	FBtr0070261,FBtr0070262,FBtr0070263,FBtr0070265,FBtr0070266,FBtr0300427,FBtr0300428,FBtr0300429,FBtr0300430,FBtr0303562,FBtr0303563,FBtr0303564	33663	14266	0	**1,23858**
RpL11	FBgn0013325	FBtr0086533	48731	20647	0	**1,23891**
Tfb1	FBgn0033929	FBtr0305616	687	291	3,72772E-23	**1,23929**
nej	FBgn0261617	FBtr0302722,FBtr0302723,FBtr0071402	10966	4642	0	**1,24022**
RpL10Ab	FBgn0036213	FBtr0076032,FBtr0304648	4417	1869	6,02289E-145	**1,24080**
CG2310	FBgn0039665	FBtr0085433,FBtr0085434	4466	1889	8,77298E-147	**1,24136**
Tfb1	FBgn0033929	FBtr0087548,FBtr0087549	688	291	2,83518E-23	**1,24139**
skpA	FBgn0025637	FBtr0070126	1447	612	4,80097E-48	**1,24146**
CG11455	FBgn0031228	FBtr0301888	1328	561	3,06078E-44	**1,24318**
CG11876	FBgn0039635	FBtr0085366,FBtr0085367,FBtr0085368,FBtr0085369	635	268	1,33986E-21	**1,24452**
RpL8	FBgn0261602	FBtr0072925	1729	729	9,98643E-58	**1,24595**
Tim10	FBgn0027360	FBtr0071677,FBtr0071676	1089	459	1,22184E-36	**1,24644**
skpA	FBgn0025637	FBtr0070127	1468	618	2,73795E-49	**1,24817**
CR41602	FBgn0085813	FBtr0114268	10829	4558	0	**1,24843**
CG42574	FBgn0260794	FBtr0303911,FBtr0303912,FBtr0303913,FBtr0303914,FBtr0303915,FBtr0303917,FBtr0301437	2389	1004	5,06869E-80	**1,25065**
Mlc2	FBgn0002773	FBtr0089746	538	226	1,18046E-18	**1,25128**
vsg	FBgn0045823	FBtr0076398,FBtr0076400	1576	662	4,29687E-53	**1,25136**
Pcd	FBgn0024841	FBtr0085458	1475	619	7,82159E-50	**1,25270**
CG7630	FBgn0040793	FBtr0075217	2295	962	1,99017E-77	**1,25439**
RpS9	FBgn0010408	FBtr0076423,FBtr0076424	16388	6868	0	**1,25468**
RpS9	FBgn0010408	FBtr0076425	16381	6861	0	**1,25553**
CG34417	FBgn0085446	FBtr0112703	4397	1839	2,34443E-148	**1,25760**
RpL8	FBgn0261602	FBtr0072924	1705	713	5,60524E-58	**1,25780**
RpS28b	FBgn0030136	FBtr0071360	50264	21009	0	**1,25852**
CG9027	FBgn0033631	FBtr0089939,FBtr0089938,FBtr0110897	1494	624	5,13403E-51	**1,25956**
CG34383	FBgn0085412	FBtr0112621,FBtr0112622,FBtr0112623,FBtr0112624,FBtr0290011	6391	2669	3,96959E-216	**1,25974**
Tfb1	FBgn0033929	FBtr0087550	731	305	2,04145E-25	**1,26106**
Cctgamma	FBgn0015019	FBtr0083337,FBtr0302370,FBtr0302371	749	312	3,93723E-26	**1,26342**
cl	FBgn0000318	FBtr0079095	5171	2154	4,80201E-176	**1,26343**
CG1354	FBgn0030151	FBtr0089384,FBtr0089385,FBtr0089383	1239	516	8,79730E-43	**1,26373**
scu	FBgn0021765	FBtr0074511	10494	4370	0	**1,26386**
CG7181	FBgn0037097	FBtr0078382	5258	2184	3,85754E-180	**1,26754**
CG42574	FBgn0260794	FBtr0303916	2503	1039	2,84960E-86	**1,26846**
CG7267	FBgn0030079	FBtr0071354	2912	1207	1,20782E-100	**1,27058**
CR40469	FBgn0058469	FBtr0307364	5465	2265	3,64451E-188	**1,27071**
Mlc2	FBgn0002773	FBtr0089747	553	229	7,96628E-20	**1,27193**

pix	FBgn0086706	FBtr0076558	1367	566	9,81140E-48	1,27214
Smr	FBgn0024308	FBtr0073708	2197	909	2,01539E-76	1,27318
CG18809	FBgn0042132	FBtr0074810,FBtr0074811	1385	573	1,89890E-48	1,27328
mask	FBgn0043884	FBtr0084562,FBtr0084563,FBtr0305043	32566	13467	0	1,27394
CG11455	FBgn0031228	FBtr0301887	1317	544	2,96396E-46	1,27558
CG11455	FBgn0031228	FBtr0078117,FBtr0306542	1305	539	6,42800E-46	1,27569
mRpL17	FBgn0035122	FBtr0072503	1087	448	1,41092E-38	1,27878
ox	FBgn0011227	FBtr0087861	2029	835	1,87746E-71	1,28092
uif	FBgn0031879	FBtr0079354,FBtr0307072,FBtr0307073, FBtr0307074,FBtr0290119,FBtr0302162	47563	19565	0	1,28156
RpS7	FBgn0039757	FBtr0089424	59156	24301	0	1,28351
RpL34a	FBgn0039406	FBtr0084972,FBtr0084973	2207	904	1,49172E-78	1,28769
RpL34a	FBgn0039406	FBtr0302369	2203	902	2,00494E-78	1,28827
RpL24	FBgn0032518	FBtr0080524	42747	17445	0	1,29301
CG5938	FBgn0046247	FBtr0085159,FBtr0085160	761	310	5,73526E-28	1,29563
HERC2	FBgn0031107	FBtr0301344	10192	4147	0	1,29730
CG13044	FBgn0036599	FBtr0075470	1321	537	3,85383E-48	1,29864
capt	FBgn0261458	FBtr0100022	1191	484	1,78304E-43	1,29909
Hsc70-4	FBgn0001219	FBtr0083057	18800	7616	0	1,30363
spen	FBgn0016977	FBtr0078122,FBtr0078121,FBtr0078123, FBtr0306341	23146	9374	0	1,30403
hoip	FBgn0015393	FBtr0079846	6828	2765	1,01545E-247	1,30418
LBR	FBgn0034657	FBtr0071713	857	347	8,23581E-32	1,30436
Mi-2	FBgn0262519	FBtr0100394	670	271	4,11576E-25	1,30587
Mi-2	FBgn0262519	FBtr0074919,FBtr0302046	670	271	4,11576E-25	1,30587
CG12384	FBgn0033624	FBtr0088155	654	264	1,20926E-24	1,30875
CG32069	FBgn0052069	FBtr0076241	825	333	9,46662E-31	1,30887
CG3214	FBgn0031436	FBtr0077745,FBtr0273393	1851	747	3,47361E-68	1,30912
CG17343	FBgn0032751	FBtr0081151	1341	541	1,16381E-49	1,30961
B52	FBgn0004587	FBtr0308196	522	210	6,38859E-20	1,31366
CR43334	FBgn0263039	FBtr0306918	660	265	4,28974E-25	1,31647
sta	FBgn0003517	FBtr0070289,FBtr0070290,FBtr0070291	94998	38139	0	1,31663
Nurf-38	FBgn0016687	FBtr0273253	2233	896	3,60835E-83	1,31741
Nurf-38	FBgn0016687	FBtr0072343	2233	896	3,60835E-83	1,31741
futsch	FBgn0259108	FBtr0112628,FBtr0307597,FBtr0307598	481	193	1,54168E-18	1,31744
RpS20	FBgn0019936	FBtr0083964	56978	22860	0	1,31758
Pcmt	FBgn0086768	FBtr0078758	4031	1617	1,31060E-149	1,31782
LBR	FBgn0034657	FBtr0071711,FBtr0071712	833	334	1,40353E-31	1,31847
RpL34b	FBgn0037686	FBtr0082049	6567	2633	1,36396E-243	1,31853
RpL34b	FBgn0037686	FBtr0082050	6575	2634	2,68329E-244	1,31974
RpL8	FBgn0261602	FBtr0072924,FBtr0072925	76066	30467	0	1,32000
Ef2b	FBgn0000559	FBtr0085911	10797	4304	0	1,32688
RpL37a	FBgn0030616	FBtr0074027	76903	30634	0	1,32791
CG11151	FBgn0030519	FBtr0073819	2418	963	1,88537E-91	1,32821
B52	FBgn0004587	FBtr0082801,FBtr0082802,FBtr0082803, FBtr0300586,FBtr0300588,FBtr0300589, FBtr0308197	543	216	3,29820E-21	1,32992
Hsp26	FBgn0001225	FBtr0076496	17674	7030	0	1,33003
ATPsyn-d	FBgn0016120	FBtr0083728	10108	4018	0	1,33095
B52	FBgn0004587	FBtr0308195	544	216	2,45758E-21	1,33258
ATPsyn-d	FBgn0016120	FBtr0083727	9926	3934	0	1,33522
CG13364	FBgn0026879	FBtr0070159	3588	1418	1,50839E-137	1,33932
CG3625	FBgn0031245	FBtr0078136	793	313	4,39624E-31	1,34116
Rack1	FBgn0020618	FBtr0079565	139923	55225	0	1,34124
Smr	FBgn0024308	FBtr0073707	2147	846	4,70490E-83	1,34359
RpS11	FBgn0033699	FBtr0088006,FBtr0088007	60034	23650	0	1,34394
CG14235	FBgn0031066	FBtr0074815,FBtr0074816,FBtr0074817	960	378	1,07575E-37	1,34465
RpS15Ab	FBgn0033555	FBtr0088226	34526	13574	0	1,34684
levy	FBgn0034877	FBtr0072061	8802	3457	0	1,34831
RpS11	FBgn0033699	FBtr0088007	13337	5237	0	1,34862
ft	FBgn0001075	FBtr0077478	42317	16531	0	1,35606
RpS15	FBgn0034138	FBtr0087123,FBtr0087124	56637	22078	0	1,35914
Pdsw	FBgn0021967	FBtr0077520,FBtr0077521	3076	1199	1,51208E-121	1,35922
CG9350	FBgn0034576	FBtr0071530	1489	578	1,08001E-59	1,36520
RpL37A	FBgn0261608	FBtr0302570	25185	9765	0	1,36687
RpL35A	FBgn0037328	FBtr0078769	28461	11033	0	1,36716
CG12935	FBgn0033547	FBtr0088221	1659	643	1,05239E-66	1,36742
CG13926	FBgn0035243	FBtr0072823	756	293	8,30855E-31	1,36749
CG4692	FBgn0035032	FBtr0072339,FBtr0072340	5360	2077	8,52063E-214	1,36773
CG5794	FBgn0039214	FBtr0084667,FBtr0084668,FBtr0306172	7438	2874	2,76307E-285	1,37185
RpL37A	FBgn0261608	FBtr0079017,FBtr0079016	25102	9689	0	1,37338
Smr	FBgn0024308	FBtr0073706	2125	820	6,36333E-86	1,37377
pix	FBgn0086706	FBtr0076557	2243	865	1,05033E-90	1,37466
CG11455	FBgn0031228	FBtr0301886	2288	882	1,30196E-92	1,37524
CG11455	FBgn0031228	FBtr0078118	2349	904	2,28922E-95	1,37765
CG9205	FBgn0035181	FBtr0072621,FBtr0072622	1528	588	2,53988E-62	1,37776
CG42239	FBgn0250868	FBtr0290307	1483	569	6,02045E-61	1,38202
CG3321	FBgn0038224	FBtr0082931,FBtr0082932,FBtr0303795	5102	1931	1,30016E-213	1,40171
CG32038	FBgn0052038	FBtr0076462	1185	448	2,61223E-50	1,40332
Sod	FBgn0003462	FBtr0076229	16857	6364	0	1,40534
Ten-m	FBgn0004449	FBtr0078509,FBtr0306107	13753	5180	0	1,40872
RpL18A	FBgn0010409	FBtr0086947	65888	24791	0	1,41020
RpL26	FBgn0036825	FBtr0075066	56833	21381	0	1,41040
Oscp	FBgn0016691	FBtr0083064	6231	2342	2,76402E-264	1,41172
RpL40	FBgn0003941	FBtr0077470	60926	22857	0	1,41442
janA	FBgn0001280	FBtr0085596	627	235	1,90738E-27	1,41580
CG10320	FBgn0034645	FBtr0071663,FBtr0113101,FBtr0113102,F Btr0300679,FBtr0300680	1061	397	5,90476E-46	1,41821
trol	FBgn0261451	FBtr0091965,FBtr0305158,FBtr0305159, FBtr0305160,FBtr0305161,FBtr0305162, FBtr0305164,FBtr0305167,FBtr0305168, FBtr0305169,FBtr0305170,FBtr0305171, FBtr0305172,FBtr0305173,FBtr0305174, FBtr0305175,FBtr0305176	436	163	1,88709E-19	1,41946
l(1)G0230	FBgn0028342	FBtr0071438	8914	3325	0	1,42272
CG34242	FBgn0085271	FBtr0300028	551	205	1,41524E-24	1,42643
Oscp	FBgn0016691	FBtr0083063	7510	2791	2,2539E-310	1,42803
Rcd4	FBgn0032034	FBtr0303472	526	195	1,15396E-23	1,43159

CG8204	FBgn0034033	FBtr0087349	421	156	3,87958E-19	1,43227
Rcd4	FBgn0032034	FBtr0079652	535	198	3,96334E-24	1,43404
Idh	FBgn0001248	FBtr0076667,FBtr0076668,FBtr0114548	592	219	1,46466E-26	1,43467
RpS18	FBgn0010411	FBtr0086273,FBtr0086274	69553	25697	0	1,43651
CG34242	FBgn0085271	FBtr0112436	547	202	1,17639E-24	1,43719
CG8184	FBgn0030674	FBtr0074082	31112	11476	0	1,43885
primo-2	FBgn0040076	FBtr0091746	510	188	3,20466E-23	1,43976
Cpr65Ec	FBgn0035737	FBtr0076909	513	189	2,75162E-23	1,44057
kis	FBgn0086902	FBtr0299837	1781	654	1,50505E-79	1,44533
Dsp1	FBgn0011764	FBtr0089260	537	197	1,29357E-24	1,44673
CG42574	FBgn0260794	FBtr0303911,FBtr0303913,FBtr0303915, FBtr0303916,FBtr0301437	1483	544	2,20424E-66	1,44684
primo-1	FBgn0040077	FBtr0091747,FBtr0091748	510	187	1,95790E-23	1,44746
CG14184	FBgn0036932	FBtr0074872,FBtr0302366	1141	418	2,16274E-51	1,44872
Mgstl	FBgn0025814	FBtr0077243	1152	422	6,36610E-52	1,44883
CG31126	FBgn0051126	FBtr0084663	604	221	1,19105E-27	1,45050
mRpS14	FBgn0044030	FBtr0074785	719	263	8,78820E-33	1,45093
ksh	FBgn0040890	FBtr0300214	798	291	1,76418E-36	1,45537
Msp-300	FBgn0261836	FBtr0303385,FBtr0303387,FBtr0303388, FBtr0303389	20374	7412	0	1,45879
CG12859	FBgn0033961	FBtr0087440	1380	502	6,50361E-63	1,45891
CG42574	FBgn0260794	FBtr0303914	851	308	1,71701E-39	1,46623
rhea	FBgn0260442	FBtr0305210,FBtr0305211,FBtr0305212	413	149	1,13874E-19	1,47083
RpL32	FBgn0002626	FBtr0114555,FBtr0114556	48531	17501	0	1,47147
RpL32	FBgn0002626	FBtr0085592,FBtr0085593,FBtr0085594	48533	17501	0	1,47153
CG1607	FBgn0039844	FBtr0085786	527	190	7,11910E-25	1,47180
CG34306	FBgn0085335	FBtr0112502	464	167	4,80272E-22	1,47428
Got2	FBgn0001125	FBtr0077867	717	258	1,00809E-33	1,47460
RpL35	FBgn0029785	FBtr0070801	8662	3106	0	1,47964
CoVa	FBgn0019624	FBtr0082474	8129	2914	0	1,48008
CG6543	FBgn0033879	FBtr0087644	603	216	9,88919E-29	1,48113
CG17508	FBgn0039970	FBtr0086071,FBtr0307172	394	141	4,94653E-19	1,48250
CG13053	FBgn0040801	FBtr0075480	450	161	1,12352E-21	1,48286
		FBtr0113128	797	285	8,65983E-38	1,48362
CG7414	FBgn0037135	FBtr0303246,FBtr0303247	1183	423	9,38629E-56	1,48372
RpL18	FBgn0035753	FBtr0076892	57566	20582	0	1,48383
CG7414	FBgn0037135	FBtr0078441,FBtr0303245	1185	423	4,92643E-56	1,48616
CG9065	FBgn0030610	FBtr0073966	580	207	9,49841E-28	1,48642
RpL7A	FBgn0014026	FBtr0070915	398	142	2,24666E-19	1,48688
CG34417	FBgn0085446	FBtr0112703,FBtr0112704,FBtr0112706,FBtr0112707,FBtr0303205,FBtr0303206,FBtr0301323,FBtr0301324	656	234	2,14424E-31	1,48719
CG13551	FBgn0040660	FBtr0072035	953	339	1,68022E-45	1,49119
RpL28	FBgn0035422	FBtr0073095,FBtr0073097,FBtr0073098, FBtr0302300,FBtr0302301,FBtr0306567	64655	22974	0	1,49276
CG13551	FBgn0040660	FBtr0072033,FBtr0072034	949	337	2,23387E-45	1,49366
janA	FBgn0001280	FBtr0085595,FBtr0085597	597	212	6,59566E-29	1,49367
CoVa	FBgn0019624	FBtr0082473	8026	2850	0	1,49372
glo	FBgn0259139	FBtr0089918,FBtr0089919	372	132	2,45200E-18	1,49476
Mgstl	FBgn0025814	FBtr0077242,FBtr0077243	1239	439	2,71972E-59	1,49688
Ten-a	FBgn0259240	FBtr0300202,FBtr0300203,FBtr0300204, FBtr0299858,FBtr0299859,FBtr0299860, FBtr0308221,FBtr0308222,FBtr0308223	466	165	9,16055E-23	1,49786
RpS14a	FBgn0004403	FBtr0071095	15929	5603	0	1,50738
CG14104	FBgn0040786	FBtr0299563	966	339	3,25808E-47	1,51074
CG14566	FBgn0037127	FBtr0078453	1199	419	8,61389E-59	1,51681
		FBtr0091745	459	160	4,98359E-23	1,52042
		FBtr0091749	459	160	4,98359E-23	1,52042
CG14997	FBgn0035515	FBtr0073225	465	162	1,92081E-23	1,52124
Doa	FBgn0259220	FBtr0299747	833	290	2,23775E-41	1,52226
dikar	FBgn0261934	FBtr0303751	948	330	5,88637E-47	1,52242
kek5	FBgn0031016	FBtr0074707	839	292	1,22891E-41	1,52270
mRpL52	FBgn0033208	FBtr0088942	1057	367	1,71659E-52	1,52612
RpS14a	FBgn0004403	FBtr0071094	15835	5493	0	1,52745
Hsp60	FBgn0015245	FBtr0073434	2133	739	1,22739E-105	1,52924
mask	FBgn0043884	FBtr0305043	3551	1230	1,62207E-175	1,52957
mask	FBgn0043884	FBtr0084562,FBtr0084563	3549	1227	4,65767E-176	1,53228
CR40502	FBgn0085737	FBtr0114187	1563	540	6,62857E-78	1,53329
Cpr78E	FBgn0037114	FBtr0078463	597	206	2,91016E-30	1,53509
vnc	FBgn0263251	FBtr0076348,FBtr0076350,FBtr0076349	584	201	7,93250E-30	1,53877
CG42455	FBgn0259932	FBtr0300238,FBtr0300239	584	201	7,93250E-30	1,53877
CG14997	FBgn0035515	FBtr0073226	462	159	1,08224E-23	1,53887
RpS7	FBgn0039757	FBtr0089423	5880	2021	3,32251E-294	1,54075
CG11752	FBgn0030292	FBtr0073452	1532	526	3,15868E-77	1,54228
CG6543	FBgn0033879	FBtr0087643	648	222	2,94334E-33	1,54543
kis	FBgn0086902	FBtr0078144	764	261	3,26085E-39	1,54952
RpL13A	FBgn0037351	FBtr0078705	71431	24399	0	1,54973
RpL36A	FBgn0031980	FBtr0079546	48536	16561	0	1,55127
Vha68-2	FBgn0020367	FBtr0080418	985	336	1,64027E-50	1,55166
mamo	FBgn0263115	FBtr0112554,FBtr0307299,FBtr0307298, FBtr0112555,FBtr0303571	660	225	3,65954E-34	1,55254
CG9669	FBgn0036667	FBtr0075339	494	168	7,76434E-26	1,55605
CG9669	FBgn0036667	FBtr0305272	494	168	7,76434E-26	1,55605
RpS7	FBgn0039757	FBtr0089424,FBtr0089425,FBtr0089422	5715	1937	6,11376E-293	1,56093
CG42238	FBgn0250867	FBtr0290305	828	280	2,90338E-43	1,56420
RpS26	FBgn0261597	FBtr0081091	74875	25311	0	1,56472
RpS26	FBgn0261597	FBtr0081090	74866	25308	0	1,56472
RpS26	FBgn0261597	FBtr0081089	74866	25308	0	1,56472
trol	FBgn0261451	FBtr0305158,FBtr0305159,FBtr0305160, FBtr0305161,FBtr0305167,FBtr0305168, FBtr0305169,FBtr0305170,FBtr0305171, FBtr0305172,FBtr0305173,FBtr0305174, FBtr0305175	738	249	1,18581E-38	1,56748
Vha68-2	FBgn0020367	FBtr0080419	983	331	1,52864E-51	1,57036
kek5	FBgn0031016	FBtr0074708	823	277	3,04931E-43	1,57101
CR33222	FBgn0053222	FBtr0300652	3218	1083	3,68146E-167	1,57113
CG42574	FBgn0260794	FBtr0303912,FBtr0303915,FBtr0301437	2196	739	2,63352E-114	1,57123
CG42574	FBgn0260794	FBtr0303913	2197	739	1,87443E-114	1,57189

CG42574	FBgn0260794	FBtr0303911,FBtr0303914,FBtr0303916, FBtr0303917	2210	742	1,63127E-115	**1,57456**
Cpr49Ag	FBgn0033730	FBtr0087922	1193	400	8,48189E-63	**1,57652**
CG5527	FBgn0039564	FBtr0085255	472	158	2,10920E-25	**1,57886**
Not1	FBgn0085436	FBtr0112674	398	133	1,35884E-21	**1,58134**
CG13319	FBgn0033781	FBtr0087788	1208	403	3,72812E-64	**1,58377**
CG13319	FBgn0033781	FBtr0300890	1208	403	3,72812E-64	**1,58377**
pncr002:3R	FBgn0063127	FBtr0091948	2537	845	2,92109E-134	**1,58610**
CG15523	FBgn0039727	FBtr0085505,FBtr0300825	401	133	4,87925E-22	**1,59218**
Bet5	FBgn0260860	FBtr0085570	766	254	2,49300E-41	**1,59252**
Cyt-b5	FBgn0033189	FBtr0088905	1065	353	4,06283E-57	**1,59311**
RpL17	FBgn0029897	FBtr0070980	341	113	8,26233E-19	**1,59345**
CG11455	FBgn0031228	FBtr0078117,FBtr0301887,FBtr0301888	1007	333	2,41441E-54	**1,59647**
RpL7A	FBgn0014026	FBtr0070916	487	161	1,30023E-26	**1,59686**
RpL35	FBgn0029785	FBtr0070800,FBtr0070801	57876	19116	0	**1,59818**
Hsp68	FBgn0001230	FBtr0084589	3477	1148	2,74089E-186	**1,59872**
CR12628	FBgn0042701	FBtr0085952	1099	362	1,37283E-59	**1,60213**
RpS18	FBgn0010411	FBtr0086273	854	281	1,31080E-46	**1,60367**
CG9775	FBgn0037261	FBtr0301297,FBtr0078894	942	308	9,81866E-52	**1,61280**
CG4692	FBgn0035032	FBtr0072340	1640	536	1,00487E-89	**1,61339**
trx	FBgn0003862	FBtr0082950,FBtr0082947	872	284	2,71852E-48	**1,61844**
rg	FBgn0086911	FBtr0110993,FBtr0290088,FBtr0299589	348	113	7,26088E-20	**1,62276**
RpL36	FBgn0002579	FBtr0070155	1419	460	1,11182E-78	**1,62517**
CG14482	FBgn0034245	FBtr0086905	2548	824	2,92025E-141	**1,62865**
Megalin	FBgn0261260	FBtr0302153	15429	4974	0	**1,63317**
RpS14b	FBgn0004404	FBtr0071096	21107	6788	0	**1,63666**
eIF-3p40	FBgn0022023	FBtr0079030	3068	986	1,04374E-171	**1,63764**
CG12848	FBgn0040666	FBtr0072437	582	187	3,26108E-33	**1,63798**
CG30415	FBgn0250838	FBtr0072031	1767	565	4,92965E-100	**1,64498**
RpL17	FBgn0029897	FBtr0070982	830	265	2,06949E-47	**1,64712**
CG4692	FBgn0035032	FBtr0072339	1511	481	4,08546E-86	**1,65139**
tweek	FBgn0261671	FBtr0304928,FBtr0304929	645	205	2,93590E-37	**1,65368**
tweek	FBgn0261671	FBtr0304927,FBtr0304930	642	204	3,34417E-37	**1,65400**
RpL36	FBgn0002579	FBtr0070158	2636	837	1,92276E-150	**1,65505**
RpS18	FBgn0010411	FBtr0086274	950	300	3,86823E-55	**1,66297**
RpL36	FBgn0002579	FBtr0070156	2678	845	2,79763E-154	**1,66413**
btsz	FBgn0053555	FBtr0091530,FBtr0273200,FBtr0306612	2799	883	2,93476E-161	**1,66443**
CG2310	FBgn0039665	FBtr0085433	419	132	7,07603E-25	**1,66641**
CG30415	FBgn0250838	FBtr0072030	1706	534	8,45147E-100	**1,67571**
RpL36	FBgn0002579	FBtr0070157	2645	825	4,07556E-155	**1,68080**
RpS6	FBgn0261592	FBtr0071135	19764	6147	0	**1,68492**
brat	FBgn0010300	FBtr0081159	647	201	9,19244E-39	**1,68657**
RpS6	FBgn0261592	FBtr0071136	19557	6034	0	**1,69650**
Mical	FBgn0053208	FBtr0082213	1141	352	2,58563E-68	**1,69665**
Mical	FBgn0053208	FBtr0082210,FBtr0082211,FBtr0082212, FBtr0082206,FBtr0082207,FBtr0082208, FBtr0082209,FBtr0273185,FBtr0306607, FBtr0306608,FBtr0306609,FBtr0306610	1141	352	2,58563E-68	**1,69665**
Abi	FBgn0020510	FBtr0082910,FBtr0307077	308	95	4,57949E-19	**1,69693**
sun	FBgn0014391	FBtr0074176,FBtr0074177	7933	2444	0	**1,69862**
RpL17	FBgn0029897	FBtr0070980,FBtr0070981,FBtr0070982, FBtr0070983	69258	21266	0	**1,70343**
eIF-3p40	FBgn0022023	FBtr0079031	2926	891	9,29318E-178	**1,71543**
RpL12	FBgn0034968	FBtr0072175,FBtr0072174	8791	2676	0	**1,71595**
Obp99a	FBgn0039678	FBtr0085450	337	102	2,56603E-21	**1,72418**
RpS13	FBgn0010265	FBtr0079724	1540	466	1,21993E-94	**1,72453**
RpS13	FBgn0010265	FBtr0100541	1540	466	1,21993E-94	**1,72453**
CG11943	FBgn0031078	FBtr0074798	526	159	7,21940E-33	**1,72604**
CG11943	FBgn0031078	FBtr0074799	526	159	7,21940E-33	**1,72604**
CG13393	FBgn0032035	FBtr0079719	4746	1434	3,55532E-291	**1,72667**
dikar	FBgn0261934	FBtr0303748,FBtr0303751	414	125	4,19100E-26	**1,72770**
RpL12	FBgn0034968	FBtr0072176	8813	2652	0	**1,73255**
CG1753	FBgn0031148	FBtr0077244	326	98	8,37932E-21	**1,73402**
Vha100-2	FBgn0028670	FBtr0083656	291	87	7,80707E-19	**1,74193**
CG14057	FBgn0036696	FBtr0075286	292	87	5,39415E-19	**1,74688**
CG14057	FBgn0036696	FBtr0300718	293	87	3,74343E-19	**1,75181**
RpS17	FBgn0005533	FBtr0076479	37720	11037	0	**1,77298**
Hsp70Aa	FBgn0013275	FBtr0082512	7048	2041	0	**1,78794**
RpS21	FBgn0015521	FBtr0273293	28457	8236	0	**1,78877**
Hsp70Ab	FBgn0013276	FBtr0082482	7268	2096	0	**1,79392**
RpL41	FBgn0066084	FBtr0100231	88787	25328	0	**1,80962**
CG9336	FBgn0032897	FBtr0081427,FBtr0307498	348	99	6,16860E-24	**1,81359**
CG42497	FBgn0260223	FBtr0300626	4208	1191	1,10687E-282	**1,82096**
RpS12	FBgn0260441	FBtr0075878	48860	13793	0	**1,82472**
CR41583	FBgn0085805	FBtr0114259	267	75	8,61536E-19	**1,83188**
CG3214	FBgn0031436	FBtr0273393	924	259	2,12054E-63	**1,83494**
CG2310	FBgn0039665	FBtr0085434	622	174	4,76114E-43	**1,83783**
fd68A	FBgn0036134	FBtr0300783,FBtr0301967	401	112	5,68224E-28	**1,84010**
sls	FBgn0086906	FBtr0072848,FBtr0072849	4029	1125	1,22380E-275	**1,84050**
RpS21	FBgn0015521	FBtr0077621,FBtr0077623	27931	7783	0	**1,84347**
Vha44	FBgn0262511	FBtr0290032,FBtr0087173	318	88	1,15506E-22	**1,85345**
cher	FBgn0014141	FBtr0089471,FBtr0290014	731	202	3,20752E-51	**1,85552**
RpS8	FBgn0039713	FBtr0085489,FBtr0306645	98829	27184	0	**1,86218**
Tom7	FBgn0033357	FBtr0088657	6086	1661	0	**1,87344**
Wnk	FBgn0037098	FBtr0305329	323	88	1,67089E-23	**1,87596**
CG3214	FBgn0031436	FBtr0077745	997	271	5,86287E-71	**1,87930**
RpL17	FBgn0029897	FBtr0070981	736	200	1,13119E-52	**1,87971**
Wnk	FBgn0037098	FBtr0305328	324	88	1,13741E-23	**1,88042**
rno	FBgn0035106	FBtr0072532,FBtr0305548,FBtr0305549	1243	336	4,60125E-89	**1,88729**
RpS23	FBgn0033912	FBtr0087575	62416	16835	0	**1,89045**
CG16936	FBgn0027590	FBtr0305309	670	180	1,04143E-48	**1,89616**
nej	FBgn0261617	FBtr0302722	7300	1956	0	**1,89999**
nej	FBgn0261617	FBtr0302723,FBtr0071402	7300	1955	0	**1,90073**
RpS30	FBgn0038834	FBtr0083969	1653	442	8,53087E-120	**1,90297**
CG16936	FBgn0027590	FBtr0072436	653	174	5,50887E-48	**1,90800**
CG1969	FBgn0039690	FBtr0085475,FBtr0306104	418	111	5,41749E-31	**1,91294**
Wnk	FBgn0037098	FBtr0305330	279	74	6,00346E-21	**1,91467**
l(2)35Di	FBgn0001989	FBtr0080771,FBtr0080772	2673	702	1,27392E-197	**1,92892**

primo-1	FBgn0040077	FBtr0091748	332	87	3,45493E-25	**1,93210**
primo-1	FBgn0040077	FBtr0091747,FBtr0091749	332	87	3,45493E-25	**1,93210**
		FBtr0091745,FBtr0091746	332	87	3,45493E-25	**1,93210**
dikar	FBgn0261934	FBtr0303748,FBtr0303749,FBtr0303750,FBtr0303751	626	164	5,75895E-47	**1,93247**
CG10320	FBgn0034645	FBtr0300679,FBtr0300680	532	139	4,55073E-40	**1,93634**
CG10320	FBgn0034645	FBtr0113101,FBtr0113102	532	139	4,55073E-40	**1,93634**
sta	FBgn0003517	FBtr0070291	51560	13449	0	**1,93875**
CG1746	FBgn0039830	FBtr0085774,FBtr0306237	3190	832	1,05288E-237	**1,93890**
CG10320	FBgn0034645	FBtr0071663	533	139	3,03553E-40	**1,93905**
sta	FBgn0003517	FBtr0070289,FBtr0070290	51484	13414	0	**1,94038**
CG1746	FBgn0039830	FBtr0085772,FBtr0085773,FBtr0305120,FBtr0305121	3190	830	1,65127E-238	**1,94237**
RpL17	FBgn0029897	FBtr0070983	711	184	4,89507E-54	**1,95014**
CG17776	FBgn0040899	FBtr0070390	1774	458	4,45333E-134	**1,95359**
RpS10b	FBgn0261593	FBtr0308192	6988	1792	0	**1,96331**
trol	FBgn0261451	FBtr0305158,FBtr0305159,FBtr0305160,FBtr0305161,FBtr0305163,FBtr0305167,FBtr0305168,FBtr0305169,FBtr0305170,FBtr0305171,FBtr0305172,FBtr0305173,FBtr0305174,FBtr0305175	623	159	3,24932E-48	**1,97021**
RpS10b	FBgn0261593	FBtr0074731,FBtr0074732	6946	1765	0	**1,97651**
NP15.6	FBgn0027785	FBtr0083712	4754	1205	0	**1,98011**
rg	FBgn0086911	FBtr0070745,FBtr0110993,FBtr0290088,FBtr0299588	401	101	9,47462E-32	**1,98925**
CG2021	FBgn0035271	FBtr0072791	1284	322	1,52835E-100	**1,99551**
stan	FBgn0024836	FBtr0303223,FBtr0304899,FBtr0304900	1879	471	5,13918E-147	**1,99617**
CR41540	FBgn0085797	FBtr0114251	773	193	5,34087E-61	**2,00187**
brm	FBgn0000212	FBtr0075525	239	59	1,22731E-19	**2,01822**
RpS30	FBgn0038834	FBtr0083970	1607	394	5,17838E-129	**2,02810**
RpLP1	FBgn0002593	FBtr0078056	84340	20638	0	**2,03091**
LBR	FBgn0034657	FBtr0071711	224	54	5,57374E-19	**2,05247**
CG7834	FBgn0039697	FBtr0085536	646	155	1,70738E-53	**2,05927**
CG7834	FBgn0039697	FBtr0085535	661	158	8,23674E-55	**2,06473**
Mhc	FBgn0086783	FBtr0080895,FBtr0080896,FBtr0080897,FBtr0080898,FBtr0080899,FBtr0080900,FBtr0080901,FBtr0080902,FBtr0080903,FBtr0080905,FBtr0080906,FBtr0080907,FBtr0301827,FBtr0301828,FBtr0301829,FBtr0307492,FBtr0307493,FBtr0307494,FBtr0307495,FBtr0307496	459	109	1,33232E-38	**2,07417**
CG7637	FBgn0033548	FBtr0088252	3018	711	2,07713E-252	**2,08567**
Tim10	FBgn0027360	FBtr0071676	3119	732	5,88566E-262	**2,09117**
stan	FBgn0024836	FBtr0088214,FBtr0300578	1590	369	2,46706E-135	**2,10733**
Tim10	FBgn0027360	FBtr0071677	3073	713	3,99202E-261	**2,10767**
CG34250	FBgn0085279	FBtr0112444	281	65	1,19654E-24	**2,11206**
RpS21	FBgn0015521	FBtr0077621,FBtr0077623,FBtr0273293	1777	409	1,45148E-152	**2,11927**
Hsp70Bc	FBgn0013279	FBtr0082638	2160	496	1,22290E-185	**2,12262**
sphinx	FBgn0083990	FBtr0111044,FBtr0111045	577	131	9,90892E-51	**2,13900**
rg	FBgn0086911	FBtr0290088	203	46	2,65205E-18	**2,14177**
rg	FBgn0086911	FBtr0070745,FBtr0110993,FBtr0299588,FBtr0299589	203	46	2,65205E-18	**2,14177**
kis	FBgn0086902	FBtr0078144,FBtr0299837	25872	5823	0	**2,15156**
CG13041	FBgn0036605	FBtr0075432	348	78	4,03172E-31	**2,15754**
Hsp70Bb	FBgn0013278	FBtr0082637	2257	495	2,55133E-203	**2,18891**
mRpL33	FBgn0040907	FBtr0070655	820	177	2,10745E-75	**2,21187**
snRNA:U12:73B	FBgn0041721	FBtr0075315	278	60	6,47495E-26	**2,21205**
CR40560	FBgn0085743	FBtr0114193	510	110	3,27652E-47	**2,21299**
Hsp70Ba	FBgn0013277	FBtr0082679	4096	882	0	**2,21537**
CR40677	FBgn0085765	FBtr0114215	275	59	6,31482E-26	**2,22064**
CG13731	FBgn0036717	FBtr0306800	304	65	1,30386E-28	**2,22556**
Obp83g	FBgn0046875	FBtr0078617	253	53	2,13383E-24	**2,25507**
RpL32	FBgn0002626	FBtr0085594	13427	2806	0	**2,25855**
RpL32	FBgn0002626	FBtr0085592,FBtr0085593,FBtr0114555,FBtr0114556	13427	2805	0	**2,25906**
RpL9	FBgn0015756	FBtr0080164	6292	1250	0	**2,33159**
RpL9	FBgn0015756	FBtr0080163	6217	1230	0	**2,33756**
RpL29	FBgn0016726	FBtr0071592,FBtr0071593,FBtr0305669	68236	13437	0	**2,34432**
LBR	FBgn0034657	FBtr0071712	194	38	4,95286E-20	**2,35199**
dp	FBgn0053196	FBtr0290006,FBtr0305136,FBtr0305137,FBtr0305138,FBtr0305139,FBtr0305140,FBtr0305141,FBtr0305142,FBtr0305143,FBtr0305144,FBtr0305145,FBtr0305146	24756	4829	0	**2,35798**
CR41544	FBgn0085799	FBtr0114253	805	157	3,43329E-81	**2,35822**
CG7580	FBgn0036728	FBtr0075222,FBtr0302509,FBtr0302510	1672	324	6,85330E-169	**2,36751**
RNaseP:RNA	FBgn0046696	FBtr0085775	575	111	1,04906E-58	**2,37300**
Hsp70Bbb	FBgn0051354	FBtr0082636	2241	425	2,46665E-230	**2,39861**
Doa	FBgn0259220	FBtr0299751	2064	391	2,13191E-212	**2,40020**
dp	FBgn0053196	FBtr0290006,FBtr0305136,FBtr0305137,FBtr0305138,FBtr0305139,FBtr0305140,FBtr0305141,FBtr0305143,FBtr0305144,FBtr0305145,FBtr0305146	886	165	7,61881E-93	**2,42484**
CR40639	FBgn0085758	FBtr0114208	958	171	1,18640E-103	**2,48603**
CR40641	FBgn0085760	FBtr0114210	928	158	3,57339E-104	**2,55420**
CR40640	FBgn0085759	FBtr0114209	895	149	2,68610E-102	**2,58658**
RpL27	FBgn0039359	FBtr0084932	157054	26028	0	**2,59312**
shot	FBgn0013733	FBtr0087617,FBtr0087619,FBtr0087616,FBtr0273222,FBtr0273223,FBtr0273224,FBtr0273225,FBtr0273226,FBtr0301591,FBtr0301592,FBtr0301593,FBtr0304847,FBtr0304848,FBtr0304849,FBtr0304850,FBtr0304851,FBtr0304852,FBtr0304853	297	49	1,44072E-34	**2,59961**
Doa	FBgn0259220	FBtr0299743	678	111	1,32812E-78	**2,61073**
		FBtr0302095,FBtr0302097,FBtr0302098,FBtr0302099,FBtr0302100,FBtr0302101,FBtr0302102,FBtr0302103	222	36	2,10090E-26	**2,62449**
RpL31	FBgn0025286	FBtr0088525	27890	4332	0	**2,68664**
RpL31	FBgn0025286	FBtr0088526,FBtr0088527	27815	4294	0	**2,69547**

CR41539	FBgn0085796	FBtr0114250	1168	179	2,65979E-141	**2,70601**
spen	FBgn0016977	FBtr0078122	412	63	2,80057E-50	**2,70922**
spen	FBgn0016977	FBtr0078121,FBtr0306341	412	63	2,80057E-50	**2,70922**
CR40642	FBgn0085761	FBtr0114211	1042	159	1,51184E-126	**2,71226**
Lcp2	FBgn0002533	FBtr0088761	375	57	5,66580E-46	**2,71786**
CR41613	FBgn0085822	FBtr0114280	978	143	3,91405E-122	**2,77382**
RpL31	FBgn0025286	FBtr0088527	63967	9249	0	**2,78996**
RpL31	FBgn0025286	FBtr0088526,FBtr0088525	63967	9247	0	**2,79027**
CR34335	FBgn0085364	FBtr0307366	116759	16764	0	**2,80010**
CG5021	FBgn0035944	FBtr0301140	295	42	8,52333E-38	**2,81225**
CG5021	FBgn0035944	FBtr0076513,FBtr0301139	295	42	8,52333E-38	**2,81225**
CG5021	FBgn0035944	FBtr0076514	295	42	8,52333E-38	**2,81225**
CR40766	FBgn0085773	FBtr0114223	606	86	2,54423E-77	**2,81691**
shot	FBgn0013733	FBtr0301964,FBtr0087621,FBtr0087617,FBtr0087618,FBtr0087619,FBtr0087620,FBtr0087616,FBtr0273222,FBtr0273223,FBtr0273224,FBtr0273225,FBtr0273226,FBtr0301591,FBtr0301592,FBtr0301593,FBtr0304847,FBtr0304848,FBtr0304849,FBtr0304850,FBtr0304852,FBtr0304853	3232	451	0	**2,84123**
Msp-300	FBgn0261836	FBtr0303383,FBtr0303385,FBtr0303388,FBtr0303389	1405	196	2,23395E-180	**2,84164**
CG7580	FBgn0036728	FBtr0075222,FBtr0302510	4472	604	0	**2,88830**
shot	FBgn0013733	FBtr0087621	2299	309	7,42956E-302	**2,89533**
RpS19a	FBgn0010412	FBtr0074313	5770	770	0	**2,90564**
l(2)efl	FBgn0011296	FBtr0072101	136	18	1,16224E-18	**2,91754**
l(2)efl	FBgn0011296	FBtr0072100	136	18	1,16224E-18	**2,91754**
CG7580	FBgn0036728	FBtr0302509	5172	678	0	**2,93137**
ple	FBgn0005626	FBtr0076956,FBtr0076957	313	41	4,67102E-42	**2,93247**
Msp-300	FBgn0261836	FBtr0303384	10170	1318	0	**2,94790**
Msp-300	FBgn0261836	FBtr0303387	10155	1307	0	**2,95786**
CR41535	FBgn0085795	FBtr0114249	1735	220	8,22182E-236	**2,97936**
CR40668	FBgn0085764	FBtr0114214	1511	189	5,45978E-207	**2,99905**
shot	FBgn0013733	FBtr0087617,FBtr0087618,FBtr0087619,FBtr0087616,FBtr0273222,FBtr0273223,FBtr0273224,FBtr0273225,FBtr0273226,FBtr0301591,FBtr0301592,FBtr0304849,FBtr0304850	226	27	3,11258E-32	**3,06529**
Lcp4	FBgn0002535	FBtr0088744	486	58	1,12664E-68	**3,06683**
zye	FBgn0036985	FBtr0078191	147	17	1,72053E-21	**3,11221**
Lcp3	FBgn0002534	FBtr0088743	183	21	1,24018E-26	**3,12338**
CG42500	FBgn0260226	FBtr0300627	282	32	3,83167E-41	**3,13955**
Msp-300	FBgn0261836	FBtr0303383,FBtr0303384,FBtr0303385,FBtr0303386,FBtr0303387,FBtr0303388,FBtr0303389	704	76	3,04342E-104	**3,21150**
CG7580	FBgn0036728	FBtr0075222	700	74	7,99058E-105	**3,24176**
CG7580	FBgn0036728	FBtr0302510	700	74	7,99058E-105	**3,24176**
RpS19a	FBgn0010412	FBtr0074312,FBtr0074311	5476	576	0	**3,24898**
CR41609	FBgn0085819	FBtr0114277	651	66	2,20914E-99	**3,30212**
CG34227	FBgn0085256	FBtr0112420	158	16	1,26353E-24	**3,30378**
Lin29	FBgn0262636	FBtr0307043,FBtr0307044,FBtr0307045,FBtr0307046,FBtr0089129,FBtr0089128	230	23	1,44564E-35	**3,32193**
CG6793	FBgn0036242	FBtr0076051	172	17	4,96306E-27	**3,33880**
CR40546	FBgn0085742	FBtr0114192	1356	133	8,49863E-210	**3,34986**
dp	FBgn0053196	FBtr0290006,FBtr0305136,FBtr0305138,FBtr0305139,FBtr0305140,FBtr0305141,FBtr0305142,FBtr0305143,FBtr0305144,FBtr0305146	594	56	1,17436E-93	**3,40696**
Msp-300	FBgn0261836	FBtr0303383,FBtr0303385	152	14	1,69611E-24	**3,44057**
shot	FBgn0013733	FBtr0087617,FBtr0087616,FBtr0273222,FBtr0273223,FBtr0273224,FBtr0301591,FBtr0304849,FBtr0304850	243	21	5,13686E-40	**3,53250**
CR40596	FBgn0085753	FBtr0114203	1657	137	4,78875E-275	**3,59633**
Lcp1	FBgn0002531	FBtr0088763	789	63	4,96932E-133	**3,64660**
CG11854	FBgn0039299	FBtr0084811	113	9	1,40418E-19	**3,65025**
shot	FBgn0013733	FBtr0087618,FBtr0087619,FBtr0273225,FBtr0273226,FBtr0301592	362	28	8,52855E-62	**3,69249**
RpS4	FBgn0011284	FBtr0075884	1107	82	1,67400E-191	**3,75489**
CR40963	FBgn0085779	FBtr0114230	112	8	4,26876E-20	**3,80735**
GstS1	FBgn0010226	FBtr0087006	100	7	3,82447E-18	**3,83650**
7SLRNA:CR42652	FBgn0261504	FBtr0302398	689	44	3,01389E-125	**3,96893**
CG17374	FBgn0040001	FBtr0305959	216	13	3,81913E-40	**4,05445**
RpS4	FBgn0011284	FBtr0075885	1058	62	4,68864E-197	**4,09293**
dp	FBgn0053196	FBtr0290006,FBtr0305136,FBtr0305137,FBtr0305138,FBtr0305139,FBtr0305140,FBtr0305144,FBtr0305146	446	26	1,70905E-83	**4,10046**
dp	FBgn0053196	FBtr0290006,FBtr0305136,FBtr0305137,FBtr0305139,FBtr0305140,FBtr0305141,FBtr0305142,FBtr0305143,FBtr0305144,FBtr0305146	457	25	7,59119E-87	**4,19219**
dp	FBgn0053196	FBtr0290006,FBtr0305136,FBtr0305137,FBtr0305138,FBtr0305139,FBtr0305140,FBtr0305143,FBtr0305146	397	21	3,73179E-76	**4,24068**
7SLRNA:CR32864	FBgn0000003	FBtr0081624	1163	57	2,25588E-227	**4,35075**
dp	FBgn0053196	FBtr0290006,FBtr0305136,FBtr0305137,FBtr0305138,FBtr0305139,FBtr0305140,FBtr0305141,FBtr0305142,FBtr0305143,FBtr0305144	3523	157	0	**4,48797**
Or2a	FBgn0023523	FBtr0070397	961	31	5,00750E-205	**4,95420**
dp	FBgn0053196	FBtr0290006,FBtr0305137,FBtr0305138,FBtr0305139,FBtr0305140,FBtr0305141,FBtr0305142,FBtr0305143,FBtr0305144	121	3	3,55179E-27	**5,33390**
Cyp4g1	FBgn0010019	FBtr0070099	204	5	1,64451E-45	**5,35050**
RNaseMRP:RNA	FBgn0065098	FBtr0091662	641	11	3,72336E-149	**5,86475**
Obp56d	FBgn0034470	FBtr0086477	1032	16	2,51983E-242	**6,01123**
CG8664	FBgn0030836	FBtr0074445	133	2	1,57588E-31	**6,05528**
CG14302	FBgn0038647	FBtr0083673	84	1	2,82643E-20	**6,39232**
CG30025	FBgn0050025	FBtr0088158	109	1	9,98671E-27	**6,76818**

Jon65Aiv	FBgn0250815	FBtr0077040	704	5	7,35623E-175	**7,13750**
yip7	FBgn0040060	FBtr0077038	282	2	6,30492E-70	**7,13955**
CG30031	FBgn0050031	FBtr0088123	243	1	2,03050E-61	**7,92481**
Jon65Aiii	FBgn0035665	FBtr0077041	585	2	2,34171E-149	**8,19229**
CG33333	FBgn0053333	FBtr0083441	119	0	5,38256E-31	**Infinity**
gammaTry	FBgn0010359	FBtr0088159	123	0	4,24171E-32	**Infinity**
Jon25Bi	FBgn0020906	FBtr0100432	201	0	1,27844E-52	**Infinity**
Jon25Bi	FBgn0020906	FBtr0079054	201	0	1,27844E-52	**Infinity**
deltaTry	FBgn0010358	FBtr0088124	255	0	7,45484E-67	**Infinity**
CG42834	FBgn0262023	FBtr0303853	278	0	6,67100E-73	**Infinity**
CG14332	FBgn0038509	FBtr0083446	394	0	1,97948E-103	**Infinity**
