# Supplementary material for: New Partners in Regulation of Gene Expression: The Enhancer of Trithorax and Polycomb Corto Interacts with Methylated Ribosomal Protein L12 Via Its Chromodomain
Source: PLoS Genet. 2012 Oct 11;8(10):e1003006. doi: 10.1371/journal.pgen.1003006 (PMC3469418; doi:10.1371/journal.pgen.1003006)
Supplement: Table S4 — Genes up-regulated in sd::Gal4>UAS::RpL12-Myc vs sd::Gal4/+. FC: Fold Change. (PDF) [file pgen.1003006.s008.pdf]

Table S4

Flybase Gene Symbol	Flybase ID Genes	Flybase ID Transcripts	Number of reads		Adjusted *P*-value	log_2(FC)
			sd::Gal4>UAS::RpL12-Myc	*sd::Gal4/+*		
MrgBP	FBgn0033341	FBtr0088684	1356	678	2,02845E-24	**1**
verm	FBgn0261341	FBtr0074954,FBtr0074957,FBtr0302334, FBtr0302335	1130	565	1,50215E-20	**1**
Ing3	FBgn0030945	FBtr0074614	1072	536	1,55490E-19	**1**
RpL34a	FBgn0039406	FBtr0084972,FBtr0084973	1808	904	3,01288E-32	**1**
RFeSP	FBgn0021906	FBtr0077915	3402	1700	3,88114E-60	**1,00085**
CG6769	FBgn0030878	FBtr0074493	2136	1067	4,27646E-38	**1,00135**
RpL34a	FBgn0039406	FBtr0302369	1806	902	2,16063E-32	**1,00160**
CG12125	FBgn0030037	FBtr0071246,FBtr0300383	1588	793	1,35688E-28	**1,00182**
betaTub56D	FBgn0003887	FBtr0086536,FBtr0086537	235587	117616	0	**1,00218**
Pglym78	FBgn0014869	FBtr0085384,FBtr0100482,FBtr0100483	3008	1501	1,51797E-53	**1,00288**
raptor	FBgn0029840	FBtr0070885	4271	2129	5,56110E-76	**1,00440**
Smr	FBgn0024308	FBtr0073706,FBtr0073707,FBtr0073708	19702	9819	0	**1,00469**
px	FBgn0003175	FBtr0071838	1130	563	8,78571E-21	**1,00512**
eIF-2alpha	FBgn0261609	FBtr0074300	9500	4730	2,15489E-164	**1,00609**
kst	FBgn0004167	FBtr0073070,FBtr0073071,FBtr0073072, FBtr0303857,FBtr0300017	25776	12802	0	**1,00966**
mRpL35	FBgn0038923	FBtr0084171	1019	506	5,40967E-19	**1,00994**
CG10527	FBgn0034583	FBtr0071541	1934	959	1,58821E-35	**1,01199**
ATPsyn-b	FBgn0019644	FBtr0076405,FBtr0076406	4670	2315	1,23433E-84	**1,01241**
PHGPx	FBgn0035438	FBtr0073066	1050	520	8,72547E-20	**1,01381**
brat	FBgn0010300	FBtr0304151	19588	9699	0	**1,01406**
betaTub56D	FBgn0003887	FBtr0086539	258292	127875	0	**1,01427**
skpA	FBgn0025637	FBtr0070124	1521	753	3,11475E-28	**1,01430**
blw	FBgn0011211	FBtr0071883	87603	43350	0	**1,01495**
Updo	FBgn0033428	FBtr0114567	2213	1095	7,50417E-41	**1,01507**
CG32529	FBgn0052529	FBtr0074800,FBtr0306520	2474	1224	1,47043E-45	**1,01524**
Ef1beta	FBgn0028737	FBtr0087041	36596	18103	0	**1,01546**
chic	FBgn0000308	FBtr0079235	2806	1388	1,45487E-51	**1,01551**
CG9027	FBgn0033631	FBtr0089939,FBtr0089938,FBtr0100658, FBtr0110897	6020	2977	8,94467E-110	**1,01590**
CG9436	FBgn0033101	FBtr0086134	2187	1081	1,63834E-40	**1,01659**
CG4769	FBgn0035600	FBtr0077144,FBtr0306086	2803	1385	9,77260E-52	**1,01709**
CG9027	FBgn0033631	FBtr0100658	3822	1888	2,87431E-70	**1,01747**
CG17121	FBgn0039043	FBtr0084361	1857	917	1,34507E-34	**1,01798**
Spn27A	FBgn0028990	FBtr0079321	10705	5284	7,61289E-191	**1,01858**
RpS9	FBgn0010408	FBtr0076423,FBtr0076425	13789	6794	3,50698E-247	**1,02118**
nocte	FBgn0261710	FBtr0071461	1940	955	1,51231E-36	**1,02248**
		FBtr0071291,FBtr0071289	16918	8328	4,02654E-304	**1,02252**
Tao-1	FBgn0031030	FBtr0074771,FBtr0303999	996	490	2,97305E-19	**1,02336**
betaTub56D	FBgn0003887	FBtr0086538	248380	122143	0	**1,02398**
RpL10Ab	FBgn0036213	FBtr0302135	4650	2286	8,23109E-87	**1,02441**
stan	FBgn0024836	FBtr0088214,FBtr0300578,FBtr0303223, FBtr0304899,FBtr0304900	9416	4628	1,98716E-170	**1,02473**
Oscp	FBgn0016691	FBtr0083064	4767	2342	3,56421E-89	**1,02534**
Oscp	FBgn0016691	FBtr0083063	5681	2791	3,97931E-106	**1,02536**
br	FBgn0000210	FBtr0300430	3420	1680	3,21195E-64	**1,02554**
RpS7	FBgn0039757	FBtr0089425,FBtr0089422,FBtr0089423	61115	29982	0	**1,02743**
Fer1HCH	FBgn0015222	FBtr0085631,FBtr0085632,FBtr0085633, FBtr0085634,FBtr0306587,FBtr0306588	2693	1321	5,64049E-51	**1,02758**
Smr	FBgn0024308	FBtr0073708	1854	909	1,84214E-35	**1,02829**
Spp	FBgn0031260	FBtr0078080	9160	4489	1,77101E-167	**1,02895**
CG8399	FBgn0034067	FBtr0087207	5734	2810	5,60370E-108	**1,02897**
CG5446	FBgn0032429	FBtr0080388	3683	1804	8,45765E-70	**1,02968**
CG5903	FBgn0038400	FBtr0083281	4957	2423	2,48674E-94	**1,03267**
Dph5	FBgn0024558	FBtr0084280	4202	2052	2,54709E-80	**1,03405**
CG13185	FBgn0033661	FBtr0113072	5630	2748	1,50516E-107	**1,03475**
EfTuM	FBgn0024556	FBtr0301389	7014	3422	2,35071E-130	**1,03540**
pcx	FBgn0003048	FBtr0070395	5518	2692	1,48674E-105	**1,03547**
EfTuM	FBgn0024556	FBtr0087670	7016	3422	1,62204E-130	**1,03581**
CG30410	FBgn0050410	FBtr0305675	4286	2090	2,81962E-82	**1,03613**
CG12859	FBgn0033961	FBtr0087440	1030	502	1,75967E-20	**1,03689**
l(2)tid	FBgn0002174	FBtr0089503,FBtr0089504	1700	828	2,87285E-33	**1,03783**
CG13185	FBgn0033661	FBtr0113071	5497	2677	8,27084E-106	**1,03803**
bou	FBgn0261284	FBtr0071004	3011	1465	1,74781E-58	**1,03934**
l(2)tid	FBgn0002174	FBtr0089505	1700	827	2,17364E-33	**1,03958**
l(2)tid	FBgn0002174	FBtr0306253	1700	827	2,17364E-33	**1,03958**
Sop2	FBgn0001961	FBtr0080569,FBtr0080570	4838	2353	1,11810E-93	**1,03991**
RpS26	FBgn0261597	FBtr0081090,FBtr0081089	17268	8397	0	**1,04016**
Aats-ala	FBgn0027094	FBtr0079721	7560	3676	4,88035E-142	**1,04025**
mRpS29	FBgn0034727	FBtr0071842	2019	981	1,10317E-39	**1,04132**
sdt	FBgn0261873	FBtr0308217,FBtr0308218,FBtr0308220	1554	754	7,06316E-31	**1,04335**
RpS5a	FBgn0002590	FBtr0074406	76228	36960	0	**1,04436**
RpL36	FBgn0002579	FBtr0070155	949	460	2,73177E-19	**1,04477**
l(1)G0334	FBgn0028325	FBtr0070711,FBtr0070708,FBtr0070709, FBtr0070710	2644	1281	3,09608E-52	**1,04545**
CG12203	FBgn0031021	FBtr0074712	1856	899	5,77592E-37	**1,04580**
mRpL27	FBgn0053002	FBtr0077451	915	443	1,13579E-18	**1,04647**
B52	FBgn0004587	FBtr0082804,FBtr0082806	3676	1779	1,38073E-72	**1,04707**
Ubp64E	FBgn0016756	FBtr0077095,FBtr0306259	1292	625	4,29989E-26	**1,04768**
Aats-ala	FBgn0027094	FBtr0079722	7373	3565	3,85872E-141	**1,04835**
RpL24	FBgn0032518	FBtr0080524	36081	17445	0	**1,04843**
CG9172	FBgn0030718	FBtr0074151	3146	1521	1,89804E-62	**1,04850**
CG9172	FBgn0030718	FBtr0074152	3128	1512	3,74710E-62	**1,04878**
Arc-p20	FBgn0031781	FBtr0079200	1935	935	8,41494E-39	**1,04930**
vav	FBgn0040068	FBtr0074677,FBtr0074678,FBtr0113316	2326	1123	1,57235E-46	**1,05049**
Zw	FBgn0004057	FBtr0074748,FBtr0074749	1413	681	7,80737E-29	**1,05303**
Ten-a	FBgn0259240	FBtr0300201,FBtr0300202,FBtr0300203, FBtr0300204,FBtr0299858,FBtr0299859, FBtr0299860,FBtr0308221,FBtr0308222, FBtr0308223	22871	11010	0	**1,05471**
Roc1a	FBgn0025638	FBtr0070122	2306	1110	1,20580E-46	**1,05483**
RpL34b	FBgn0037686	FBtr0082050	5473	2634	1,21542E-109	**1,05508**
l(2)03709	FBgn0010551	FBtr0086614,FBtr0086615,FBtr0086616, FBtr0112861,FBtr0112862,FBtr0112863	6680	3214	9,00205E-134	**1,05548**
		FBtr0273184	2311	1111	6,74669E-47	**1,05666**
RpL34b	FBgn0037686	FBtr0082049	5477	2633	3,80051E-110	**1,05668**

Tig	FBgn0011722	FBtr0079275	986	474	1,45030E-20	**1,05670**
Updo	FBgn0033428	FBtr0088524	2724	1309	2,88788E-55	**1,05726**
B52	FBgn0004587	FBtr0082801,FBtr0082802,FBtr0082803, FBtr0300586,FBtr0300588,FBtr0300589, FBtr0308195,FBtr0308196,FBtr0308197	3637	1747	1,45863E-73	**1,05787**
CR41548	FBgn0085802	FBtr0114256	2827	1357	1,68458E-57	**1,05885**
CG1943	FBgn0037468	FBtr0081635	2292	1100	7,39472E-47	**1,05910**
CG9603	FBgn0040529	FBtr0081855	2129	1021	1,12543E-43	**1,06019**
Tsp	FBgn0031850	FBtr0079311,FBtr0079312,FBtr0079313, FBtr0079314,FBtr0114488	3059	1466	1,60260E-62	**1,06117**
mRpL22	FBgn0030786	FBtr0074330	1639	785	6,61194E-34	**1,06205**
RpL3	FBgn0020910	FBtr0082346,FBtr0302586	179648	85974	0	**1,06320**
cp309	FBgn0086690	FBtr0305977,FBtr0305978,FBtr0305979	2467	1180	7,18769E-51	**1,06397**
RpL35	FBgn0029785	FBtr0070800,FBtr0070801	39985	19116	0	**1,06468**
RpL9	FBgn0015756	FBtr0080163,FBtr0080164	54967	26270	0	**1,06515**
Hsp60	FBgn0015245	FBtr0073434,FBtr0073435	30921	14762	0	**1,06670**
Aats-glupro	FBgn0005674	FBtr0084511	7987	3810	1,11739E-159	**1,06786**
RpS9	FBgn0010408	FBtr0076423,FBtr0076424	14398	6868	2,20976E-287	**1,06791**
RpS9	FBgn0010408	FBtr0076425	14386	6861	2,57971E-287	**1,06817**
rhea	FBgn0260442	FBtr0076627,FBtr0305210,FBtr0305211, FBtr0305212	7750	3695	3,21227E-155	**1,06862**
CG10249	FBgn0027596	FBtr0087429,FBtr0112929,FBtr0303757	1129	538	5,09001E-24	**1,06937**
CG9331	FBgn0032889	FBtr0081419,FBtr0081420,FBtr0081421, FBtr0081417,FBtr0081418,FBtr0302942, FBtr0302943,FBtr0302944,FBtr0302945	3119	1486	5,74885E-65	**1,06965**
CG5548	FBgn0030605	FBtr0073960	1564	745	5,87926E-33	**1,06993**
CG11505	FBgn0035424	FBtr0073045,FBtr0073046,FBtr0308061	7724	3679	3,48202E-155	**1,07003**
Mrtf	FBgn0052296	FBtr0072944,FBtr0290067,FBtr0306331, FBtr0306332	926	441	7,12517E-20	**1,07023**
Rpt3	FBgn0028686	FBtr0073436	11484	5459	6,92312E-232	**1,07292**
Mdh1	FBgn0262782	FBtr0080050	5004	2377	1,15282E-104	**1,07394**
cic	FBgn0262582	FBtr0305029	7631	3622	4,92467E-155	**1,07509**
BM-40-SPARC	FBgn0026562	FBtr0085094	2615	1241	2,68763E-55	**1,07531**
hang	FBgn0026575	FBtr0074276,FBtr0074277	4626	2195	3,12667E-97	**1,07554**
CG30185	FBgn0050185	FBtr0072060	1568	744	1,79184E-33	**1,07555**
Taf10	FBgn0028398	FBtr0077717	1580	749	8,51566E-34	**1,07689**
RpL37a	FBgn0030616	FBtr0074027	64643	30634	0	**1,07736**
Arp11	FBgn0031050	FBtr0074760	1752	830	1,71734E-37	**1,07782**
trx	FBgn0003862	FBtr0082950,FBtr0082949,FBtr0082947, FBtr0082948,FBtr0100277	13768	6521	4,30880E-281	**1,07815**
RpS20	FBgn0019936	FBtr0083964	48284	22860	0	**1,07872**
CG8111	FBgn0035825	FBtr0076770	1234	584	8,45395E-27	**1,07930**
CG30499	FBgn0050499	FBtr0088954	2545	1203	1,72548E-54	**1,08103**
Bruce	FBgn0037808	FBtr0082239	16476	7785	0	**1,08160**
RpL11	FBgn0013325	FBtr0086533	43709	20647	0	**1,08200**
RpL10Ab	FBgn0036213	FBtr0076032,FBtr0304648	3961	1869	7,57611E-85	**1,08360**
RpII15	FBgn0004855	FBtr0082925	2361	1114	7,38044E-51	**1,08365**
tutl	FBgn0010473	FBtr0089271,FBtr0089269,FBtr0089270, FBtr0301863,FBtr0301864	1560	736	6,24925E-34	**1,08377**
Tim10	FBgn0027360	FBtr0071677,FBtr0071676	973	459	1,58722E-21	**1,08395**
proPO-A1	FBgn0261362	FBtr0302291	1904	898	3,17951E-41	**1,08425**
RpL36	FBgn0002579	FBtr0070158	1776	837	1,24978E-38	**1,08533**
CycG	FBgn0039858	FBtr0085801	3220	1517	2,06798E-69	**1,08584**
cher	FBgn0014141	FBtr0089471,FBtr0303364,FBtr0290014	12825	6042	2,75630E-266	**1,08586**
CG2200	FBgn0030447	FBtr0073721	1159	545	1,23436E-25	**1,08855**
RpL36	FBgn0002579	FBtr0070156	1797	845	2,38795E-39	**1,08857**
capt	FBgn0261458	FBtr0100022	1030	484	5,95350E-23	**1,08957**
Ahcy13	FBgn0014455	FBtr0074030	11618	5455	8,39892E-244	**1,09071**
Ppox	FBgn0020018	FBtr0084664	1430	669	4,94926E-32	**1,09594**
CG3321	FBgn0038224	FBtr0082931,FBtr0082932,FBtr0303795	4130	1931	5,80349E-91	**1,09679**
Scsalpha	FBgn0004888	FBtr0073151,FBtr0303096	5813	2713	1,59555E-128	**1,09939**
scf	FBgn0025682	FBtr0089942	1091	509	8,89578E-25	**1,09991**
vsg	FBgn0045823	FBtr0076399,FBtr0076401	1392	649	1,59137E-31	**1,10087**
Got2	FBgn0001125	FBtr0077867,FBtr0077868	903	421	9,86467E-21	**1,10091**
CG5261	FBgn0031912	FBtr0079444	858	400	9,27935E-20	**1,10098**
RpS15	FBgn0034138	FBtr0087123,FBtr0087124	47369	22078	0	**1,10133**
Pros29	FBgn0261394	FBtr0071522	9725	4531	4,48414E-209	**1,10187**
CG3500	FBgn0034849	FBtr0072038	894	416	1,33385E-20	**1,10369**
scf	FBgn0025682	FBtr0089940	1090	507	4,79831E-25	**1,10427**
RpL36	FBgn0002579	FBtr0070157	1774	825	3,14239E-40	**1,10454**
CG4542	FBgn0029906	FBtr0070970	1200	557	1,29857E-27	**1,10729**
CG33156	FBgn0053156	FBtr0087681,FBtr0087682,FBtr0087683, FBtr0087684,FBtr0113450	1178	546	2,71163E-27	**1,10937**
Smr	FBgn0024308	FBtr0073707	1827	846	6,59094E-42	**1,11075**
mRpL52	FBgn0033208	FBtr0088942	793	367	1,05539E-18	**1,11154**
RpS7	FBgn0039757	FBtr0089424	52580	24301	0	**1,11350**
Prosalpha5	FBgn0016697	FBtr0086910,FBtr0086911	4518	2088	4,74544E-103	**1,11356**
Rbcn-3A	FBgn0023458	FBtr0303341	10992	5071	2,52448E-243	**1,11611**
Sop2	FBgn0001961	FBtr0080570	978	451	4,31329E-23	**1,11671**
Aldh-III	FBgn0010548	FBtr0088993	1299	599	1,82255E-30	**1,11677**
RpL13	FBgn0011272	FBtr0079888,FBtr0100164	64576	29771	0	**1,11709**
CG30410	FBgn0050410	FBtr0301852	3924	1809	2,59922E-90	**1,11713**
Got2	FBgn0001125	FBtr0100359	1628	750	4,89251E-38	**1,11814**
		FBtr0302094,FBtr0302095,FBtr0302096, FBtr0302097,FBtr0302098,FBtr0302099, FBtr0302100,FBtr0302101,FBtr0302102, FBtr0302103	7429	3421	1,49151E-165	**1,11875**
vari	FBgn0250785	FBtr0089947,FBtr0089946	3150	1450	4,95283E-73	**1,11930**
CG3523	FBgn0027571	FBtr0077659,FBtr0290093	4259	1958	8,83308E-99	**1,12113**
vari	FBgn0250785	FBtr0089945	3006	1381	3,96900E-70	**1,12213**
RpL36A	FBgn0031980	FBtr0079546	36080	16561	0	**1,12341**
CG42669	FBgn0261551	FBtr0302685,FBtr0302683,FBtr0302684, FBtr0302678,FBtr0302679,FBtr0302682, FBtr0302676,FBtr0306701,FBtr0306702, FBtr0306703	1623	744	1,86271E-38	**1,12529**
RpS6	FBgn0261592	FBtr0071135,FBtr0071136	111250	50993	0	**1,12543**
RFeSP	FBgn0021906	FBtr0077914,FBtr0077915	743	340	3,79271E-18	**1,12783**
obst-B	FBgn0027600	FBtr0079934	3586	1640	1,31360E-81	**1,12868**
mRpS16	FBgn0033907	FBtr0087578	1052	481	1,82160E-25	**1,12903**
Cct5	FBgn0010621	FBtr0087987,FBtr0087988	31691	14486	0	**1,12941**
larp	FBgn0261618	FBtr0301180,FBtr0306389,FBtr0306390	2050	937	8,34373E-49	**1,12950**
CG8036	FBgn0037607	FBtr0081881	4799	2191	1,70528E-113	**1,13114**

tweek	FBgn0261671	FBtr0304927,FBtr0304928,FBtr0304929, FBtr0304930	6981	3187	1,34051E-159	**1,13123**
CR40728	FBgn0085769	FBtr0114219	3614	1648	5,89390E-86	**1,13288**
CG8036	FBgn0037607	FBtr0081882	4799	2188	5,09744E-114	**1,13312**
CG11873	FBgn0039633	FBtr0085365	14527	6623	0	**1,13318**
spen	FBgn0016977	FBtr0078122,FBtr0078121,FBtr0078123, FBtr0306341	20572	9374	0	**1,13395**
RpL32	FBgn0002626	FBtr0114555,FBtr0114556	38439	17501	0	**1,13513**
RpL32	FBgn0002626	FBtr0085592,FBtr0085593,FBtr0085594	38440	17501	0	**1,13517**
mRpS35	FBgn0035374	FBtr0301626	1007	458	8,85487E-25	**1,13664**
CG11151	FBgn0030519	FBtr0073819	2118	963	3,89143E-51	**1,13709**
vsg	FBgn0045823	FBtr0076398,FBtr0076400	1456	662	1,99537E-35	**1,13711**
CG17202	FBgn0038043	FBtr0082587	2755	1252	2,33591E-66	**1,13782**
dom	FBgn0020306	FBtr0071602,FBtr0071603,FBtr0071604, FBtr0301350	4751	2159	5,70525E-114	**1,13787**
eIF-3p40	FBgn0022023	FBtr0079030,FBtr0079031	20098	9133	0	**1,13789**
CG10320	FBgn0034645	FBtr0071663,FBtr0113101,FBtr0113102,F Btr0300679,FBtr0300680	874	397	1,07774E-21	**1,13849**
CG31937	FBgn0031360	FBtr0077884	1566	711	3,07450E-38	**1,13916**
CG34347	FBgn0085376	FBtr0290046	1106	502	2,90180E-27	**1,13959**
skpA	FBgn0025637	FBtr0070126	1350	612	3,64370E-33	**1,14136**
Hsp68	FBgn0001230	FBtr0084589	2533	1148	1,41021E-61	**1,14172**
Smr	FBgn0024308	FBtr0073706	1812	820	1,66556E-44	**1,14389**
mRpL49	FBgn0030433	FBtr0073691	747	338	7,63433E-19	**1,14409**
betaTub56D	FBgn0003887	FBtr0086536	22722	10277	0	**1,14467**
CG32038	FBgn0052038	FBtr0076462	991	448	9,69282E-25	**1,14539**
asrij	FBgn0034793	FBtr0071902	1308	591	1,64796E-32	**1,14613**
RpS15Ab	FBgn0033555	FBtr0088226	30053	13574	0	**1,14666**
CG2812	FBgn0034931	FBtr0072140	1674	756	2,00998E-41	**1,14684**
CG6746	FBgn0032394	FBtr0080313	1909	862	3,56162E-47	**1,14706**
Sop2	FBgn0001961	FBtr0080569	960	433	3,60115E-24	**1,14867**
CG4692	FBgn0035032	FBtr0072339,FBtr0072340	4614	2077	9,03463E-114	**1,15152**
Dpy-30L1	FBgn0032293	FBtr0080112	1865	839	1,34589E-46	**1,15243**
CG14619	FBgn0031187	FBtr0077346,FBtr0077347,FBtr0077348	872	392	2,67198E-22	**1,15347**
skpA	FBgn0025637	FBtr0070127	1375	618	1,31705E-34	**1,15375**
fwd	FBgn0004373	FBtr0072516	893	401	6,81503E-23	**1,15506**
Tpi	FBgn0086355	FBtr0085584	4545	2040	5,24417E-113	**1,15571**
CG11999	FBgn0037312	FBtr0078807	2133	957	1,46128E-53	**1,15629**
RpS3	FBgn0002622	FBtr0084410	127827	57350	0	**1,15633**
CG7267	FBgn0030079	FBtr0071354	2695	1207	8,72760E-68	**1,15886**
CG14184	FBgn0036932	FBtr0074872,FBtr0302366	934	418	4,00500E-24	**1,15992**
zormin	FBgn0052311	FBtr0089864,FBtr0110856,FBtr0110857,F Btr0110858	3785	1689	8,71380E-96	**1,16412**
TfIIA-S	FBgn0013347	FBtr0084526	5546	2474	3,45767E-140	**1,16460**
CG9350	FBgn0034576	FBtr0071530	1299	578	1,19570E-33	**1,16826**
RpL35A	FBgn0037328	FBtr0078769	24812	11033	0	**1,16921**
Cctgamma	FBgn0015019	FBtr0083338	827	367	6,41575E-22	**1,17211**
Tpi	FBgn0086355	FBtr0085582,FBtr0085583	4494	1994	1,74024E-115	**1,17233**
Act57B	FBgn0000044	FBtr0071519	4280	1898	3,44358E-110	**1,17313**
Act57B	FBgn0000044	FBtr0300946	4284	1899	1,81495E-110	**1,17372**
Mgstl	FBgn0025814	FBtr0077243	953	422	3,09209E-25	**1,17523**
Dhc64C	FBgn0261797	FBtr0273370,FBtr0073359	28549	12641	0	**1,17533**
MED14	FBgn0035145	FBtr0072554	1189	526	2,20497E-31	**1,17661**
CG8191	FBgn0030675	FBtr0074083	918	406	1,67769E-24	**1,17701**
CG13393	FBgn0032035	FBtr0079719	3245	1434	1,51959E-84	**1,17817**
Sin	FBgn0028402	FBtr0304820	1888	834	1,45531E-49	**1,17874**
ATPsyn-d	FBgn0016120	FBtr0083728	9102	4018	2,02952E-228	**1,17971**
CG10249	FBgn0027596	FBtr0087430	809	357	8,59212E-22	**1,18022**
Sin	FBgn0028402	FBtr0078288	1888	833	1,03530E-49	**1,18047**
m2	FBgn0002592	FBtr0084981	3767	1657	2,98810E-99	**1,18484**
CG3731	FBgn0038271	FBtr0083001	781	343	2,70443E-21	**1,18711**
ATPsyn-d	FBgn0016120	FBtr0083727	8970	3934	4,28410E-229	**1,18911**
r	FBgn0003189	FBtr0089734,FBtr0089733	8357	3665	1,63811E-213	**1,18917**
rut	FBgn0003301	FBtr0073992,FBtr0305581	3792	1659	1,87129E-101	**1,19265**
RpS14a	FBgn0004403	FBtr0071095	12812	5603	0	**1,19322**
ash2	FBgn0000139	FBtr0084660	1303	569	1,55793E-35	**1,19534**
CG13630	FBgn0039219	FBtr0084745	2572	1123	2,04401E-69	**1,19553**
poe	FBgn0011230	FBtr0079545	28310	12354	0	**1,19633**
levy	FBgn0034877	FBtr0072061	7922	3457	4,05860E-205	**1,19634**
osa	FBgn0261885	FBtr0301487,FBtr0089582,FBtr0089581	1117	487	9,55087E-31	**1,19764**
RpL40	FBgn0003941	FBtr0077470	52488	22857	0	**1,19935**
CR41602	FBgn0085813	FBtr0114268	10476	4558	3,56653E-273	**1,20062**
PHGPx	FBgn0035438	FBtr0073066,FBtr0073067,FBtr0073068	6224	2700	7,33921E-170	**1,20488**
CG13349	FBgn0033886	FBtr0087610,FBtr0087611,FBtr0114493,F Btr0114494	3589	1556	1,91705E-98	**1,20574**
CG13349	FBgn0033886	FBtr0114495	3590	1556	1,48786E-98	**1,20614**
nej	FBgn0261617	FBtr0302722,FBtr0302723,FBtr0071402	10720	4642	4,91976E-283	**1,20749**
CG30415	FBgn0250838	FBtr0072031	1305	565	1,77717E-36	**1,20773**
Trxr-1	FBgn0020653	FBtr0071168	1082	468	1,79786E-30	**1,20912**
CG14235	FBgn0031066	FBtr0074815,FBtr0074816,FBtr0074817	876	378	6,22016E-25	**1,21254**
CG5941	FBgn0029833	FBtr0070867,FBtr0305344	1859	801	3,49880E-52	**1,21465**
br	FBgn0000210	FBtr0070261,FBtr0070262,FBtr0070263, FBtr0070265,FBtr0070266,FBtr0300427, FBtr0300428,FBtr0300429,FBtr0300430, FBtr0303562,FBtr0303563,FBtr0303564	33111	14266	0	**1,21473**
RpS14a	FBgn0004403	FBtr0071094	12753	5493	0	**1,21517**
CG18809	FBgn0042132	FBtr0074810,FBtr0074811	1332	573	8,72816E-38	**1,21699**
CG11876	FBgn0039635	FBtr0085366,FBtr0085367,FBtr0085368, FBtr0085369	623	268	3,56711E-18	**1,21700**
pck	FBgn0013720	FBtr0100198	3369	1448	1,39936E-94	**1,21826**
pck	FBgn0013720	FBtr0070255,FBtr0070256	3367	1446	8,87143E-95	**1,21940**
RpS11	FBgn0033699	FBtr0088007	12208	5237	0	**1,22101**
Tom7	FBgn0033357	FBtr0088657	3873	1661	3,25915E-109	**1,22140**
CG42574	FBgn0260794	FBtr0303911,FBtr0303912,FBtr0303913, FBtr0303914,FBtr0303915,FBtr0303917, FBtr0301437	2345	1004	1,07407E-66	**1,22383**
CG34383	FBgn0085412	FBtr0112621,FBtr0112622,FBtr0112623,F Btr0112624,FBtr0290011	6241	2669	6,21303E-177	**1,22548**
CG7637	FBgn0033548	FBtr0088252	1664	711	7,13032E-48	**1,22673**
RpS11	FBgn0033699	FBtr0088006,FBtr0088007	55357	23650	0	**1,22693**
sea	FBgn0037912	FBtr0100464	6604	2819	4,73054E-188	**1,22816**
fabp	FBgn0037913	FBtr0100320,FBtr0100321,FBtr0100323	6604	2819	4,73054E-188	**1,22816**
olf186-F	FBgn0041585	FBtr0086796,FBtr0086797	707	301	4,82222E-21	**1,23195**

CG11438	FBgn0037164	FBtr0078504	2032	865	8,47996E-59	1,23213
wdb	FBgn0027492	FBtr0085203,FBtr0085204	610	259	2,30516E-18	1,23586
Rack1	FBgn0020618	FBtr0079565	130140	55225	0	1,23667
RpS18	FBgn0010411	FBtr0086273,FBtr0086274	60696	25697	0	1,24000
CG2310	FBgn0039665	FBtr0085433,FBtr0085434	4463	1889	3,60414E-130	1,24039
blot	FBgn0027660	FBtr0075232,FBtr0075233	1118	473	2,86418E-33	1,24101
Hsp26	FBgn0001225	FBtr0076496	16622	7030	0	1,24150
RpL35	FBgn0029785	FBtr0070801	7348	3106	1,76972E-206	1,24229
Act5C	FBgn0000042	FBtr0070823	8303	3508	1,67098E-233	1,24298
CG1354	FBgn0030151	FBtr0089384,FBtr0089385,FBtr0089383	1224	516	1,39495E-36	1,24616
RpS21	FBgn0015521	FBtr0077621,FBtr0077623,FBtr0273293	972	409	2,34572E-29	1,24886
CG7414	FBgn0037135	FBtr0303246,FBtr0303247	1006	423	2,11969E-30	1,24990
CG30415	FBgn0250838	FBtr0072030	1271	534	2,36104E-38	1,25105
CG7414	FBgn0037135	FBtr0078441,FBtr0303245	1007	423	1,62501E-30	1,25133
CG42574	FBgn0260794	FBtr0303916	2476	1039	3,84382E-74	1,25282
kek5	FBgn0031016	FBtr0074707	696	292	1,80572E-21	1,25312
sta	FBgn0003517	FBtr0070289,FBtr0070290,FBtr0070291	91013	38139	0	1,25481
CG2021	FBgn0035271	FBtr0072791	771	322	6,37152E-24	1,25967
RpL29	FBgn0016726	FBtr0071592,FBtr0071593,FBtr0305669	32177	13437	0	1,25982
CG3609	FBgn0031418	FBtr0077791,FBtr0303027	1608	671	4,40110E-49	1,26088
brm	FBgn0000212	FBtr0075523,FBtr0075525	607	253	4,14219E-19	1,26256
brm	FBgn0000212	FBtr0075524	607	252	2,84018E-19	1,26827
ksh	FBgn0040890	FBtr0300214	701	291	3,31346E-22	1,26840
LRP1	FBgn0053087	FBtr0088791	12347	5118	0	1,27051
mask	FBgn0043884	FBtr0084562,FBtr0084563,FBtr0305043	32498	13467	0	1,27092
CG8193	FBgn0033367	FBtr0088663	2911	1203	2,19217E-90	1,27488
Hsc70-4	FBgn0001219	FBtr0083057	18436	7616	0	1,27542
CG4692	FBgn0035032	FBtr0072340	1299	536	6,32366E-41	1,27710
kek5	FBgn0031016	FBtr0074708	675	277	5,54844E-22	1,28500
		FBtr0113128	696	285	9,54732E-23	1,28813
Cyt-b5	FBgn0033189	FBtr0088905	863	353	4,45226E-28	1,28969
Cctgamma	FBgn0015019	FBtr0083337,FBtr0302370,FBtr0302371	764	312	5,22363E-25	1,29203
CG3446	FBgn0029868	FBtr0070924	3847	1571	1,00584E-122	1,29205
CG13551	FBgn0040660	FBtr0072033,FBtr0072034	826	337	5,11396E-27	1,29339
RpL18A	FBgn0010409	FBtr0086947	60777	24791	0	1,29371
CG13551	FBgn0040660	FBtr0072035	832	339	2,88453E-27	1,29530
N	FBgn0004647	FBtr0070507,FBtr0304659	50238	20453	0	1,29647
Mgstl	FBgn0025814	FBtr0077242,FBtr0077243	1080	439	1,96015E-35	1,29874
Tfb1	FBgn0033929	FBtr0305616	718	291	5,61648E-24	1,30296
Tfb1	FBgn0033929	FBtr0087548,FBtr0087549	718	291	5,61648E-24	1,30296
CG5938	FBgn0046247	FBtr0085159,FBtr0085160	765	310	1,81692E-25	1,30319
RpL3	FBgn0020910	FBtr0082347,FBtr0082346,FBtr0302586	5585	2260	9,04915E-182	1,30523
Tfb1	FBgn0033929	FBtr0087550	754	305	2,94374E-25	1,30576
tyf	FBgn0026083	FBtr0070643,FBtr0300931,FBtr0305180, FBtr0305181,FBtr0305182	1940	783	4,54019E-64	1,30897
olf186-F	FBgn0041585	FBtr0086795	548	221	9,45318E-19	1,31013
Aldh-III	FBgn0010548	FBtr0300477	640	258	1,13612E-21	1,31070
p16-ARC	FBgn0031437	FBtr0077694	3989	1608	3,70245E-131	1,31076
RpL3	FBgn0020910	FBtr0082347	36900	14850	0	1,31316
RpL26	FBgn0036825	FBtr0075066	53130	21381	0	1,31320
CR31144	FBgn0051144	FBtr0303864	36902	14849	0	1,31333
shot	FBgn0013733	FBtr0301964,FBtr0087621,FBtr0087617, FBtr0087618,FBtr0087619,FBtr0087620, FBtr0087616,FBtr0273222,FBtr0273223, FBtr0273224,FBtr0273225,FBtr0273226, FBtr0301591,FBtr0301592,FBtr0301593, FBtr0304847,FBtr0304848,FBtr0304849, FBtr0304850,FBtr0304851,FBtr0304852, FBtr0304853	17016	6807	0	1,32180
CG42238	FBgn0250867	FBtr0290305	700	280	4,31219E-24	1,32193
CG4692	FBgn0035032	FBtr0072339	1205	481	4,20700E-41	1,32492
vfl	FBgn0259789	FBtr0307536,FBtr0307537	16920	6742	0	1,32748
RpL18	FBgn0035753	FBtr0076892	51658	20582	0	1,32761
CG34417	FBgn0085446	FBtr0112703	4622	1839	1,30786E-156	1,32960
CG9674	FBgn0036663	FBtr0075341,FBtr0075344,FBtr0305915, FBtr0305916	5183	2052	1,65759E-177	1,33676
CG9205	FBgn0035181	FBtr0072621,FBtr0072622	1486	588	1,90608E-51	1,33755
RpS14b	FBgn0004404	FBtr0071096	17193	6788	0	1,34076
RpL8	FBgn0261602	FBtr0072925	1847	729	3,67771E-64	1,34119
CG1620	FBgn0033183	FBtr0088983,FBtr0300830	2336	921	3,34598E-81	1,34277
faf	FBgn0005632	FBtr0307549	998	393	3,47063E-35	1,34451
RpL28	FBgn0035422	FBtr0073095,FBtr0073097,FBtr0073098, FBtr0302300,FBtr0302301,FBtr0306567	58476	22974	0	1,34784
faf	FBgn0005632	FBtr0085843,FBtr0085844,FBtr0307547, FBtr0307548,FBtr0307550	996	391	1,99334E-35	1,34898
RpL8	FBgn0261602	FBtr0072924,FBtr0072925	77654	30467	0	1,34981
RpL8	FBgn0261602	FBtr0072924	1818	713	4,22117E-64	1,35038
Ten-m	FBgn0004449	FBtr0078509,FBtr0306107	13224	5180	0	1,35213
vnc	FBgn0263251	FBtr0076348,FBtr0076350,FBtr0076349	514	201	8,97391E-19	1,35457
CG42455	FBgn0259932	FBtr0300238,FBtr0300239	514	201	8,97391E-19	1,35457
CG3214	FBgn0031436	FBtr0273393	663	259	4,65282E-24	1,35606
Sod	FBgn0003462	FBtr0076229	16330	6364	0	1,35952
Pdsw	FBgn0021967	FBtr0077520,FBtr0077521	3079	1199	9,65291E-110	1,36063
HERC2	FBgn0031107	FBtr0301344	10653	4147	0	1,36112
CG9065	FBgn0030610	FBtr0073966	532	207	1,14323E-19	1,36180
Ef2b	FBgn0000559	FBtr0085911	11062	4304	0	1,36186
Aldh-III	FBgn0010548	FBtr0300478	513	199	3,80716E-19	1,36619
RpS18	FBgn0010411	FBtr0086273	727	281	5,44742E-27	1,37139
CG34422	FBgn0085451	FBtr0112723	3153	1217	1,49290E-114	1,37340
CoVa	FBgn0019624	FBtr0082474	7578	2914	4,11407E-264	1,37882
CoVa	FBgn0019624	FBtr0082473	7473	2850	5,86938E-265	1,39073
Got2	FBgn0001125	FBtr0077867	678	258	5,23090E-26	1,39391
CG6543	FBgn0033879	FBtr0087644	568	216	5,14521E-22	1,39486
kis	FBgn0086902	FBtr0299837	1729	654	6,09444E-66	1,40258
CG3214	FBgn0031436	FBtr0077745	717	271	7,16269E-28	1,40368
ft	FBgn0001075	FBtr0077478	43774	16531	0	1,40490
RpL17	FBgn0029897	FBtr0070982	702	265	2,35785E-27	1,40548
CG16936	FBgn0027590	FBtr0305309	477	180	6,32678E-19	1,40599
CG16936	FBgn0027590	FBtr0072436	462	174	2,06832E-18	1,40881
scu	FBgn0021765	FBtr0074511	11634	4370	0	1,41264
CG9027	FBgn0033631	FBtr0089939,FBtr0089938,FBtr0110897	1662	624	2,11347E-64	1,41330
RpL13A	FBgn0037351	FBtr0078705	65012	24399	0	1,41388
RpS26	FBgn0261597	FBtr0081089	67583	25308	0	1,41707

RpS26	FBgn0261597	FBtr0081090	67583	25308	0	**1,41707**
RpS26	FBgn0261597	FBtr0081091	67591	25311	0	**1,41707**
RpS18	FBgn0010411	FBtr0086274	802	300	1,15051E-31	**1,41864**
CG6543	FBgn0033879	FBtr0087643	594	222	1,10233E-23	**1,41990**
TfIIA-L	FBgn0011289	FBtr0085155,FBtr0085156	504	188	2,38395E-20	**1,42269**
kis	FBgn0086902	FBtr0078144	704	261	2,16974E-28	**1,43153**
CG5794	FBgn0039214	FBtr0084667,FBtr0084668,FBtr0306172	7786	2874	1,08681E-294	**1,43782**
RpS7	FBgn0039757	FBtr0089423	5477	2021	3,11347E-218	**1,43832**
CG9775	FBgn0037261	FBtr0301297,FBtr0078894	835	308	7,12469E-34	**1,43885**
uif	FBgn0031879	FBtr0079354,FBtr0307072,FBtr0307073, FBtr0307074,FBtr0290119,FBtr0302162	53062	19565	0	**1,43940**
Msp-300	FBgn0261836	FBtr0303385,FBtr0303387,FBtr0303388, FBtr0303389	20108	7412	0	**1,43983**
CG34417	FBgn0085446	FBtr0112703,FBtr0112704,FBtr0112706,F Btr0112707,FBtr0303205,FBtr0303206,F Btr0301323,FBtr0301324	642	234	6,02278E-27	**1,45606**
CG1702	FBgn0031117	FBtr0077310,FBtr0300210	580	211	1,95504E-24	**1,45881**
RpL12	FBgn0034968	FBtr0072175,FBtr0072174	7359	2676	1,06329E-286	**1,45943**
tweek	FBgn0261671	FBtr0304928,FBtr0304929	564	205	6,36952E-24	**1,46007**
RpS7	FBgn0039757	FBtr0089424,FBtr0089425,FBtr0089422	5340	1937	3,10484E-220	**1,46302**
l(1)G0230	FBgn0028342	FBtr0071438	9175	3325	0	**1,46435**
tweek	FBgn0261671	FBtr0304927,FBtr0304930	563	204	5,36343E-24	**1,46457**
dikar	FBgn0261934	FBtr0303751	914	330	1,02140E-38	**1,46973**
RpL12	FBgn0034968	FBtr0072176	7369	2652	9,52401E-293	**1,47439**
Ten-a	FBgn0259240	FBtr0300202,FBtr0300203,FBtr0300204, FBtr0299858,FBtr0299859,FBtr0299860, FBtr0308221,FBtr0308222,FBtr0308223	459	165	6,01706E-20	**1,47603**
CG1746	FBgn0039830	FBtr0085773	426	153	1,16010E-18	**1,47732**
CG1607	FBgn0039844	FBtr0085786	530	190	4,05734E-23	**1,47999**
CG13319	FBgn0033781	FBtr0300890	1128	403	1,51085E-48	**1,48492**
CG13319	FBgn0033781	FBtr0087788	1128	403	1,51085E-48	**1,48492**
mask	FBgn0043884	FBtr0305043	3444	1230	1,27091E-146	**1,48543**
CG34306	FBgn0085335	FBtr0112502	468	167	1,29914E-20	**1,48666**
Cpr49Ag	FBgn0033730	FBtr0087922	1121	400	2,31493E-48	**1,48671**
mask	FBgn0043884	FBtr0084562,FBtr0084563	3443	1227	3,00523E-147	**1,48853**
CR40502	FBgn0085737	FBtr0114187	1517	540	1,83393E-65	**1,49019**
l(2)03709	FBgn0010551	FBtr0086614,FBtr0086615,FBtr0112861, FBtr0112862	411	146	2,32033E-18	**1,49317**
brat	FBgn0010300	FBtr0081159	567	201	3,69156E-25	**1,49615**
verm	FBgn0261341	FBtr0074956,FBtr0302333,FBtr0302334	771	273	5,76557E-34	**1,49783**
RpS12	FBgn0260441	FBtr0075878	39217	13793	0	**1,50754**
CG8184	FBgn0030674	FBtr0074082	32770	11476	0	**1,51376**
futsch	FBgn0259108	FBtr0112628,FBtr0307597,FBtr0307598	552	193	4,45728E-25	**1,51607**
mamo	FBgn0263115	FBtr0112554,FBtr0307299,FBtr0307298, FBtr0112555,FBtr0303571	644	225	3,05928E-29	**1,51714**
Hsp60	FBgn0015245	FBtr0073434	2117	739	1,92599E-94	**1,51838**
trx	FBgn0003862	FBtr0082950,FBtr0082947	814	284	7,73322E-37	**1,51914**
Mi-2	FBgn0262519	FBtr0074919,FBtr0302046	777	271	3,22826E-35	**1,51962**
Mi-2	FBgn0262519	FBtr0100394	778	271	2,35543E-35	**1,52148**
trol	FBgn0261451	FBtr0091965,FBtr0305158,FBtr0305159, FBtr0305160,FBtr0305161,FBtr0305162, FBtr0305164,FBtr0305167,FBtr0305168, FBtr0305169,FBtr0305170,FBtr0305171, FBtr0305172,FBtr0305173,FBtr0305174, FBtr0305175,FBtr0305176	472	163	5,23314E-22	**1,53391**
pix	FBgn0086706	FBtr0076558	1642	566	4,56731E-75	**1,53658**
RpS8	FBgn0039713	FBtr0085489,FBtr0306645	79075	27184	0	**1,54046**
CG42574	FBgn0260794	FBtr0303911,FBtr0303913,FBtr0303915, FBtr0303916,FBtr0301437	1586	544	3,45290E-73	**1,54371**
Cyp4e2	FBgn0014469	FBtr0088745	950	323	8,81515E-45	**1,55639**
btsz	FBgn0053555	FBtr0091530,FBtr0273200,FBtr0306612	2600	883	1,30122E-121	**1,55803**
CR33222	FBgn0053222	FBtr0300652	3189	1083	4,43155E-149	**1,55807**
CR12628	FBgn0042701	FBtr0085952	1066	362	2,33199E-50	**1,55815**
Bet5	FBgn0260860	FBtr0085570	748	254	1,34826E-35	**1,55821**
Hsp70Bb	FBgn0013278	FBtr0082637	1484	495	5,24905E-72	**1,58399**
eIF-3p40	FBgn0022023	FBtr0079030	2971	986	1,06406E-144	**1,59129**
Sac1	FBgn0035195	FBtr0072652	417	138	4,15926E-21	**1,59538**
RpL17	FBgn0029897	FBtr0070980,FBtr0070981,FBtr0070982, FBtr0070983	64301	21266	0	**1,59629**
Hsp70Ab	FBgn0013276	FBtr0082482	6355	2096	5,0911E-312	**1,60025**
l(2)35Di	FBgn0001989	FBtr0080771,FBtr0080772	2139	702	2,34782E-106	**1,60739**
CG42574	FBgn0260794	FBtr0303913	2258	739	7,95111E-113	**1,61140**
CG42574	FBgn0260794	FBtr0303912,FBtr0303915,FBtr0301437	2258	739	7,95111E-113	**1,61140**
CG42574	FBgn0260794	FBtr0303911,FBtr0303914,FBtr0303916, FBtr0303917	2268	742	2,76007E-113	**1,61193**
Hsp70Aa	FBgn0013275	FBtr0082512	6244	2041	2,2398E-311	**1,61319**
w	FBgn0003996	FBtr0070490	24501	7981	0	**1,61820**
LBR	FBgn0034657	FBtr0071713	1067	347	2,98977E-54	**1,62055**
pix	FBgn0086706	FBtr0076557	2667	865	3,47800E-135	**1,62445**
NP15.6	FBgn0027785	FBtr0083712	3717	1205	3,10931E-188	**1,62511**
Hsp70Bc	FBgn0013279	FBtr0082638	1532	496	3,53658E-78	**1,62700**
CG42497	FBgn0260223	FBtr0300626	3700	1191	1,60227E-189	**1,63535**
CG13044	FBgn0036599	FBtr0075470	1672	537	2,29718E-86	**1,63858**
rg	FBgn0086911	FBtr0110993,FBtr0290088,FBtr0299589	352	113	8,07683E-19	**1,63925**
CG14566	FBgn0037127	FBtr0078453	1311	419	1,93231E-68	**1,64565**
RpL17	FBgn0029897	FBtr0070981	626	200	4,35608E-33	**1,64616**
LBR	FBgn0034657	FBtr0071711,FBtr0071712	1047	334	5,25938E-55	**1,64834**
RpS17	FBgn0005533	FBtr0076479	34716	11037	0	**1,65325**
RpS30	FBgn0038834	FBtr0083969	1394	442	1,45428E-73	**1,65711**
eIF-3p40	FBgn0022023	FBtr0079031	2814	891	3,27338E-148	**1,65912**
RpS10b	FBgn0261593	FBtr0308192	5661	1792	9,82692E-298	**1,65949**
sta	FBgn0003517	FBtr0070291	42546	13449	0	**1,66152**
sta	FBgn0003517	FBtr0070289,FBtr0070290	42480	13414	0	**1,66304**
CG10320	FBgn0034645	FBtr0300679,FBtr0300680	441	139	4,20980E-24	**1,66569**
CG10320	FBgn0034645	FBtr0113101,FBtr0113102	441	139	4,20980E-24	**1,66569**
CG10320	FBgn0034645	FBtr0071663	441	139	4,20980E-24	**1,66569**
RpS10b	FBgn0261593	FBtr0074731,FBtr0074732	5626	1765	3,36163E-300	**1,67244**
RpS13	FBgn0010265	FBtr0079724	1490	466	2,02153E-80	**1,67691**
RpS13	FBgn0010265	FBtr0100541	1490	466	2,02153E-80	**1,67691**
CG11943	FBgn0031078	FBtr0074798	509	159	4,97075E-28	**1,67864**
CG11943	FBgn0031078	FBtr0074799	509	159	4,97075E-28	**1,67864**
Doa	FBgn0259220	FBtr0299747	934	290	2,69728E-51	**1,68737**
RpLP1	FBgn0002593	FBtr0078056	66904	20638	0	**1,69679**

RpL17	FBgn0029897	FBtr0070983	600	184	6,88621E-34	**1,70526**
Hsp70Ba	FBgn0013277	FBtr0082679	2890	882	2,99430E-161	**1,71222**
RNaseP:RNA	FBgn0046696	FBtr0085775	364	111	5,77067E-21	**1,71338**
RpS6	FBgn0261592	FBtr0071135	20267	6147	0	**1,72118**
CG42574	FBgn0260794	FBtr0303914	1016	308	9,88814E-58	**1,72190**
CG15523	FBgn0039727	FBtr0085505,FBtr0300825	440	133	1,29931E-25	**1,72608**
RpS6	FBgn0261592	FBtr0071136	20076	6034	0	**1,73429**
Megalin	FBgn0261260	FBtr0302153	16691	4974	0	**1,74659**
RpS23	FBgn0033912	FBtr0087575	56586	16835	0	**1,74898**
CG2310	FBgn0039665	FBtr0085434	585	174	1,81391E-34	**1,74935**
CR41540	FBgn0085797	FBtr0114251	649	193	3,31210E-38	**1,74962**
CG7580	FBgn0036728	FBtr0075222,FBtr0302509,FBtr0302510	1100	324	3,19133E-65	**1,76344**
glo	FBgn0259139	FBtr0089918,FBtr0089919	449	132	3,98137E-27	**1,76618**
Mical	FBgn0053208	FBtr0082210,FBtr0082211,FBtr0082212, FBtr0082206,FBtr0082207,FBtr0082208, FBtr0082209,FBtr0273185,FBtr0306607, FBtr0306608,FBtr0306609,FBtr0306610	1203	352	6,55911E-72	**1,77299**
Mical	FBgn0053208	FBtr0082213	1203	352	6,55911E-72	**1,77299**
RpS30	FBgn0038834	FBtr0083970	1351	394	5,52606E-81	**1,77776**
cher	FBgn0014141	FBtr0089471,FBtr0290014	699	202	6,76225E-43	**1,79094**
Hsp70Bbb	FBgn0051354	FBtr0082636	1490	425	3,83321E-92	**1,80978**
Vha68-2	FBgn0020367	FBtr0080418	1183	336	9,76048E-74	**1,81592**
CG1753	FBgn0031148	FBtr0077244	346	98	3,12821E-22	**1,81992**
Vha68-2	FBgn0020367	FBtr0080419	1180	331	8,17851E-75	**1,83388**
CR34335	FBgn0085364	FBtr0307366	59881	16764	0	**1,83673**
Sp1	FBgn0020378	FBtr0071381	409	114	1,22853E-26	**1,84307**
Sp1	FBgn0020378	FBtr0273354	402	112	3,14236E-26	**1,84370**
nej	FBgn0261617	FBtr0302722	7129	1956	0	**1,86579**
nej	FBgn0261617	FBtr0302723,FBtr0071402	7128	1955	0	**1,86633**
trol	FBgn0261451	FBtr0305158,FBtr0305159,FBtr0305160, FBtr0305161,FBtr0305167,FBtr0305168, FBtr0305169,FBtr0305170,FBtr0305171, FBtr0305172,FBtr0305173,FBtr0305174, FBtr0305175	909	249	1,15029E-59	**1,86813**
CR41583	FBgn0085805	FBtr0114259	278	75	3,65309E-19	**1,89012**
Tim10	FBgn0027360	FBtr0071676	2727	732	1,88149E-182	**1,89740**
Tim10	FBgn0027360	FBtr0071677	2666	713	3,03812E-179	**1,90270**
l(2)03659	FBgn0010549	FBtr0302504	792	209	1,15172E-54	**1,92200**
sls	FBgn0086906	FBtr0072848,FBtr0072849	4301	1125	2,75893E-297	**1,93475**
CG1746	FBgn0039830	FBtr0085774,FBtr0306237	3273	832	1,54529E-234	**1,97596**
CG1746	FBgn0039830	FBtr0085772,FBtr0085773,FBtr0305120, FBtr0305121	3271	830	7,90917E-235	**1,97855**
RpL32	FBgn0002626	FBtr0085594	11065	2806	0	**1,97942**
RpL32	FBgn0002626	FBtr0085592,FBtr0085593,FBtr0114555, FBtr0114556	11065	2805	0	**1,97993**
mRpL33	FBgn0040907	FBtr0070655	701	177	3,09869E-51	**1,98567**
Jon65Aiv	FBgn0250815	FBtr0077040	5615	5	0	**10,13310**
CG30031	FBgn0050031	FBtr0088123	1877	1	0	**10,87420**
Jon65Aiii	FBgn0035665	FBtr0077041	4035	2	0	**10,97840**
dikar	FBgn0261934	FBtr0303748,FBtr0303749,FBtr0303750, FBtr0303751	658	164	8,63195E-49	**2,00439**
kis	FBgn0086902	FBtr0078144,FBtr0299837	23745	5823	0	**2,02779**
rg	FBgn0086911	FBtr0070745,FBtr0110993,FBtr0290088, FBtr0299588	419	101	1,22908E-32	**2,05259**
CG7834	FBgn0039697	FBtr0085536	645	155	5,81319E-50	**2,05703**
CG7834	FBgn0039697	FBtr0085535	661	158	1,88600E-51	**2,06473**
rno	FBgn0035106	FBtr0072532,FBtr0305548,FBtr0305549	1413	336	8,51227E-110	**2,07223**
Mhc	FBgn0086783	FBtr0080895,FBtr0080896,FBtr0080897, FBtr0080898,FBtr0080899,FBtr0080900, FBtr0080901,FBtr0080902,FBtr0080903, FBtr0080905,FBtr0080906,FBtr0080907, FBtr0301827,FBtr0301828,FBtr0301829, FBtr0307492,FBtr0307493,FBtr0307494, FBtr0307495,FBtr0307496	461	109	1,51503E-36	**2,08044**
CG5527	FBgn0039564	FBtr0085255	683	158	5,07519E-55	**2,11196**
stan	FBgn0024836	FBtr0303223,FBtr0304899,FBtr0304900	2038	471	4,54002E-163	**2,11336**
RpL9	FBgn0015756	FBtr0080164	5422	1250	0	**2,11690**
RpL9	FBgn0015756	FBtr0080163	5366	1230	0	**2,12519**
RpL27	FBgn0039359	FBtr0084932	115859	26028	0	**2,15423**
Obp83g	FBgn0046875	FBtr0078617	237	53	2,65578E-20	**2,16082**
CR40677	FBgn0085765	FBtr0114215	272	59	7,58356E-24	**2,20482**
stan	FBgn0024836	FBtr0088214,FBtr0300578	1717	369	2,24361E-148	**2,21820**
CR40560	FBgn0085743	FBtr0114193	512	110	9,16523E-45	**2,21864**
trol	FBgn0261451	FBtr0091965,FBtr0305158,FBtr0305159, FBtr0305160,FBtr0305161,FBtr0305162, FBtr0305164,FBtr0305170,FBtr0305171, FBtr0305172,FBtr0305173,FBtr0305174, FBtr0305175,FBtr0305176	317	67	2,41028E-28	**2,24225**
Pdsw	FBgn0021967	FBtr0077521	239	50	9,43386E-22	**2,25701**
CR41544	FBgn0085799	FBtr0114253	763	157	4,48760E-69	**2,28092**
CG13731	FBgn0036717	FBtr0306800	317	65	2,76307E-29	**2,28597**
Doa	FBgn0259220	FBtr0299751	1914	391	1,17758E-173	**2,29135**
dp	FBgn0053196	FBtr0290006,FBtr0305136,FBtr0305137, FBtr0305138,FBtr0305139,FBtr0305140, FBtr0305141,FBtr0305142,FBtr0305143, FBtr0305144,FBtr0305145,FBtr0305146	24449	4829	0	**2,33998**
CR40639	FBgn0085758	FBtr0114208	872	171	1,44483E-82	**2,35033**
RpL31	FBgn0025286	FBtr0088525	22162	4332	0	**2,35498**
LBR	FBgn0034657	FBtr0071711	277	54	7,16803E-27	**2,35885**
RpL31	FBgn0025286	FBtr0088526,FBtr0088527	22083	4294	0	**2,36254**
RpL31	FBgn0025286	FBtr0088527	47798	9249	0	**2,36958**
RpL31	FBgn0025286	FBtr0088526,FBtr0088525	47797	9247	0	**2,36986**
spen	FBgn0016977	FBtr0078122	326	63	1,35009E-31	**2,37145**
spen	FBgn0016977	FBtr0078121,FBtr0306341	326	63	1,35009E-31	**2,37145**
CR40642	FBgn0085761	FBtr0114211	839	159	8,87050E-82	**2,39964**
trol	FBgn0261451	FBtr0305158,FBtr0305159,FBtr0305160, FBtr0305161,FBtr0305163,FBtr0305167, FBtr0305168,FBtr0305169,FBtr0305170, FBtr0305171,FBtr0305172,FBtr0305173, FBtr0305174,FBtr0305175	860	159	1,03851E-85	**2,43531**
CR40640	FBgn0085759	FBtr0114209	808	149	1,35899E-80	**2,43904**
CR40641	FBgn0085760	FBtr0114210	857	158	1,52143E-85	**2,43937**
CG7580	FBgn0036728	FBtr0075222,FBtr0302510	3336	604	0	**2,46550**

CG7580	FBgn0036728	FBtr0302509	3937	678	0	**2,53774**
CR41539	FBgn0085796	FBtr0114250	1050	179	1,43038E-111	**2,55236**
Doa	FBgn0259220	FBtr0299743	659	111	5,85723E-71	**2,56972**
CR41613	FBgn0085822	FBtr0114280	865	143	4,27492E-94	**2,59668**
shot	FBgn0013733	FBtr0087617,FBtr0087619,FBtr0087616, FBtr0273222,FBtr0273223,FBtr0273224, FBtr0273225,FBtr0273226,FBtr0301591, FBtr0301592,FBtr0301593,FBtr0304847, FBtr0304848,FBtr0304849,FBtr0304850, FBtr0304851,FBtr0304852,FBtr0304853	301	49	1,33127E-33	**2,61891**
Msp-300	FBgn0261836	FBtr0303383,FBtr0303385,FBtr0303388, FBtr0303389	1211	196	9,03802E-134	**2,62727**
CG5021	FBgn0035944	FBtr0301140	266	42	1,89011E-30	**2,66297**
CG5021	FBgn0035944	FBtr0076514	266	42	1,89011E-30	**2,66297**
CG5021	FBgn0035944	FBtr0076513,FBtr0301139	266	42	1,89011E-30	**2,66297**
dp	FBgn0053196	FBtr0290006,FBtr0305136,FBtr0305137, FBtr0305138,FBtr0305139,FBtr0305140, FBtr0305141,FBtr0305143,FBtr0305144, FBtr0305145,FBtr0305146	1069	165	3,63714E-122	**2,69572**
CR40766	FBgn0085773	FBtr0114223	576	86	9,73860E-68	**2,74366**
LBR	FBgn0034657	FBtr0071712	255	38	2,93383E-30	**2,74643**
RpS19a	FBgn0010412	FBtr0074313	5172	770	0	**2,74779**
Msp-300	FBgn0261836	FBtr0303384	8863	1318	0	**2,74944**
CR40668	FBgn0085764	FBtr0114214	1273	189	1,84661E-149	**2,75177**
Msp-300	FBgn0261836	FBtr0303387	8847	1307	0	**2,75893**
CR41535	FBgn0085795	FBtr0114249	1557	220	1,13033E-188	**2,82319**
shot	FBgn0013733	FBtr0087621	2321	309	6,23090E-292	**2,90907**
shot	FBgn0013733	FBtr0301964,FBtr0087621,FBtr0087617, FBtr0087618,FBtr0087619,FBtr0087620, FBtr0087616,FBtr0273222,FBtr0273223, FBtr0273224,FBtr0273225,FBtr0273226, FBtr0301591,FBtr0301592,FBtr0301593, FBtr0304847,FBtr0304848,FBtr0304849, FBtr0304850,FBtr0304852,FBtr0304853	3476	451	0	**2,94623**
shot	FBgn0013733	FBtr0087617,FBtr0087618,FBtr0087619, FBtr0087616,FBtr0273222,FBtr0273223, FBtr0273224,FBtr0273225,FBtr0273226, FBtr0301591,FBtr0301592,FBtr0304849, FBtr0304850	218	27	4,71026E-29	**3,01330**
CG7580	FBgn0036728	FBtr0075222	601	74	2,13652E-79	**3,02177**
CG7580	FBgn0036728	FBtr0302510	601	74	2,13652E-79	**3,02177**
RpS19a	FBgn0010412	FBtr0074312,FBtr0074311	4923	576	0	**3,09540**
dp	FBgn0053196	FBtr0290006,FBtr0305136,FBtr0305138, FBtr0305139,FBtr0305140,FBtr0305141, FBtr0305142,FBtr0305143,FBtr0305144, FBtr0305146	481	56	8,38778E-66	**3,10254**
CG6793	FBgn0036242	FBtr0076051	153	17	7,82312E-22	**3,16992**
7SLRNA:CR42652	FBgn0261504	FBtr0302398	397	44	9,97671E-56	**3,17356**
CG34227	FBgn0085256	FBtr0112420	151	16	5,97235E-22	**3,23840**
Lcp4	FBgn0002535	FBtr0088744	549	58	7,46502E-79	**3,24268**
Lcp2	FBgn0002533	FBtr0088761	541	57	1,28545E-77	**3,24659**
CG42500	FBgn0260226	FBtr0300627	304	32	7,16354E-44	**3,24793**
CR41609	FBgn0085819	FBtr0114277	639	66	1,10696E-92	**3,27528**
CR40546	FBgn0085742	FBtr0114192	1289	133	2,05515E-186	**3,27675**
CR40596	FBgn0085753	FBtr0114203	1377	137	1,52824E-202	**3,32928**
Msp-300	FBgn0261836	FBtr0303383,FBtr0303384,FBtr0303385, FBtr0303386,FBtr0303387,FBtr0303388, FBtr0303389	770	76	5,32955E-114	**3,34079**
CG32249	FBgn0052249	FBtr0303003	124	12	5,98986E-19	**3,36923**
7SLRNA:CR32864	FBgn0000003	FBtr0081624	594	57	3,69132E-89	**3,38143**
CG31676	FBgn0051676	FBtr0081424	168	16	1,26871E-25	**3,39232**
Msp-300	FBgn0261836	FBtr0303383,FBtr0303385	148	14	9,40593E-23	**3,40210**
shot	FBgn0013733	FBtr0087617,FBtr0087616,FBtr0273222, FBtr0273223,FBtr0273224,FBtr0301591, FBtr0304849,FBtr0304850	245	21	6,66763E-39	**3,54432**
zye	FBgn0036985	FBtr0078191	199	17	9,68261E-32	**3,54916**
RpS4	FBgn0011284	FBtr0075884	970	82	2,32340E-153	**3,56429**
shot	FBgn0013733	FBtr0087618,FBtr0087619,FBtr0273225, FBtr0273226,FBtr0301592	363	28	1,30971E-59	**3,69647**
CG11854	FBgn0039299	FBtr0084811	124	9	3,20425E-21	**3,78427**
Sgs3	FBgn0003373	FBtr0076096	131	9	1,29458E-22	**3,86350**
RpS4	FBgn0011284	FBtr0075885	911	62	5,90811E-156	**3,87711**
dp	FBgn0053196	FBtr0290006,FBtr0305136,FBtr0305137, FBtr0305139,FBtr0305140,FBtr0305141, FBtr0305142,FBtr0305143,FBtr0305144, FBtr0305146	407	25	2,48580E-72	**4,02503**
dp	FBgn0053196	FBtr0290006,FBtr0305136,FBtr0305137, FBtr0305138,FBtr0305139,FBtr0305140, FBtr0305143,FBtr0305146	345	21	1,46952E-61	**4,03814**
Obp56d	FBgn0034470	FBtr0086477	284	16	7,19216E-52	**4,14975**
fzo	FBgn0011596	FBtr0084399	125	7	5,78661E-23	**4,15843**
dp	FBgn0053196	FBtr0290006,FBtr0305136,FBtr0305137, FBtr0305138,FBtr0305139,FBtr0305140, FBtr0305141,FBtr0305142,FBtr0305143, FBtr0305144	2921	157	0	**4,21763**
Lcp1	FBgn0002531	FBtr0088763	1190	63	8,83966E-220	**4,23947**
dp	FBgn0053196	FBtr0290006,FBtr0305136,FBtr0305137, FBtr0305138,FBtr0305139,FBtr0305140, FBtr0305144,FBtr0305146	495	26	7,91398E-92	**4,25084**
CG13323	FBgn0033788	FBtr0087797	1012	53	1,71492E-187	**4,25507**
Lcp3	FBgn0002534	FBtr0088743	401	21	1,85618E-74	**4,25514**
RNaseMRP:RNA	FBgn0065098	FBtr0091662	505	11	1,10730E-110	**5,52071**
Jon25Bii	FBgn0031654	FBtr0079055	384	6	2,09111E-87	**6**
CG8997	FBgn0028920	FBtr0080563	464	6	1,76286E-107	**6,27302**
CG7953	FBgn0028533	FBtr0080550	559	4	1,12901E-134	**7,12670**
CG14302	FBgn0038647	FBtr0083673	199	1	1,07591E-48	**7,63662**
CG12115	FBgn0030097	FBtr0071341	203	1	9,78933E-50	**7,66534**
CG33346	FBgn0053346	FBtr0302527	432	2	4,93881E-106	**7,75489**
CG13324	FBgn0033789	FBtr0087796	233	1	2,81714E-57	**7,86419**
CG8664	FBgn0030836	FBtr0074445	591	2	2,43871E-146	**8,20701**
yip7	FBgn0040060	FBtr0077038	1014	2	1,82092E-254	**8,98584**
CG30025	FBgn0050025	FBtr0088158	919	1	1,66024E-232	**9,84392**
CG10912	FBgn0034296	FBtr0086807	2431	0	0	**Infinity**

Jon25Bi	FBgn0020906	FBtr0079054	627	0	4,81400E-160	**Infinity**
CG42834	FBgn0262023	FBtr0303853	99	0	3,91641E-25	**Infinity**
Jon25Bi	FBgn0020906	FBtr0100432	627	0	4,81400E-160	**Infinity**
betaTry	FBgn0010357	FBtr0088122	77	0	1,61178E-19	**Infinity**
gammaTry	FBgn0010359	FBtr0088159	853	0	2,70298E-218	**Infinity**
Phae1	FBgn0263234	FBtr0080334	106	0	1,11263E-26	**Infinity**
CG31789	FBgn0051789	FBtr0081071	523	0	1,28207E-133	**Infinity**
fu12	FBgn0026718	FBtr0079703,FBtr0079704,FBtr0302160	131	0	3,39076E-33	**Infinity**
Mur29B	FBgn0051901	FBtr0079607	245	0	2,68673E-62	**Infinity**
dro2	FBgn0052279	FBtr0073059	348	0	9,41430E-89	**Infinity**
CG32302	FBgn0052302	FBtr0072881	875	0	5,91697E-224	**Infinity**
CG33333	FBgn0053333	FBtr0083441	251	0	6,34766E-64	**Infinity**
alphaTry	FBgn0003863	FBtr0088161	398	0	1,96420E-101	**Infinity**
CG8661	FBgn0030837	FBtr0074444	106	0	1,11263E-26	**Infinity**
CG15044	FBgn0030928	FBtr0074627	144	0	2,37484E-36	**Infinity**
CG14332	FBgn0038509	FBtr0083446	142	0	4,11540E-36	**Infinity**
CG15818	FBgn0031910	FBtr0079442	593	0	1,84237E-151	**Infinity**
CG6933	FBgn0036952	FBtr0074853,FBtr0074854,FBtr0074855	266	0	1,06148E-67	**Infinity**
deltaTry	FBgn0010358	FBtr0088124	1855	0	0	**Infinity**
CG7465	FBgn0035551	FBtr0073306	1184	0	2,83852E-303	**Infinity**
