# Supplementary material for: New Partners in Regulation of Gene Expression: The Enhancer of Trithorax and Polycomb Corto Interacts with Methylated Ribosomal Protein L12 Via Its Chromodomain
Source: PLoS Genet. 2012 Oct 11;8(10):e1003006. doi: 10.1371/journal.pgen.1003006 (PMC3469418; doi:10.1371/journal.pgen.1003006)
Supplement: Table S5 — Genes down-regulated in sd::Gal4>UAS::FH-cortoCD vs sd::Gal4/+. FC: Fold Change. (PDF) [file pgen.1003006.s009.pdf]

Table S5

Flybase Gene Symbol	Flybase ID Genes	Flybase ID Transcripts	Number of reads		Adjusted *P*-value	log_2(FC)
			sd::Gal4>UAS::FH-cortoCD	*sd::Gal4/+*		
CaMKI	FBgn0016126	FBtr0089069,FBtr0089066,FBtr0089067, FBtr0089068	250	500	6,56626E-30	**-1,00000**
Atf6	FBgn0033010	FBtr0086079,FBtr0086080	1038	2078	4,33038E-122	**-1,00139**
CG11266	FBgn0031883	FBtr0089637,FBtr0089635,FBtr0089636	432	866	1,91378E-51	**-1,00334**
CG11266	FBgn0031883	FBtr0089638,FBtr0089639,FBtr0089640	429	861	3,28916E-51	**-1,00504**
eyg	FBgn0000625	FBtr0075979	248	498	4,70554E-30	**-1,00581**
Dcp2	FBgn0036534	FBtr0304975	6152	12369	0,00000E+00	**-1,00760**
par-1	FBgn0260934	FBtr0100391,FBtr0100392,FBtr0301504	730	1468	3,36598E-87	**-1,00788**
Scm	FBgn0003334	FBtr0082102	768	1546	6,75490E-92	**-1,00936**
gish	FBgn0250823	FBtr0301304,FBtr0305071,FBtr0305072	1312	2644	6,46761E-157	**-1,01095**
tna	FBgn0026160	FBtr0301658	491	995	4,23945E-60	**-1,01897**
Nipped-B	FBgn0026401	FBtr0301454,FBtr0301455,FBtr0301456	572	1162	2,51391E-70	**-1,02252**
RecQ5	FBgn0027375	FBtr0075714,FBtr0075715,FBtr0100362	329	669	6,64369E-41	**-1,02392**
HmgZ	FBgn0010228	FBtr0071673,FBtr0071674	4231	8618	0,00000E+00	**-1,02635**
CG14135	FBgn0036193	FBtr0290309	154	314	9,19721E-20	**-1,02783**
CG11486	FBgn0035397	FBtr0072998,FBtr0072999,FBtr0073000, FBtr0073001,FBtr0073002,FBtr0073003, FBtr0072991,FBtr0072992,FBtr0113130, FBtr0113131	357	730	5,79395E-45	**-1,03197**
CG17528	FBgn0261387	FBtr0111276,FBtr0111275,FBtr0111277	238	487	2,28810E-30	**-1,03296**
Nipped-B	FBgn0026401	FBtr0111119,FBtr0111118	574	1175	4,78325E-72	**-1,03354**
pho	FBgn0002521	FBtr0089204	165	338	2,13210E-21	**-1,03456**
CR42722	FBgn0261639	FBtr0303009	270	556	6,67483E-35	**-1,04213**
heph	FBgn0011224	FBtr0085885	148	305	1,32839E-19	**-1,04321**
Sap47	FBgn0013334	FBtr0083204,FBtr0083205,FBtr0083206, FBtr0083207,FBtr0083208,FBtr0083209, FBtr0083210,FBtr0301655	929	1917	6,73693E-119	**-1,04510**
pAbp	FBgn0261619	FBtr0086740	788	1627	3,81698E-101	**-1,04595**
Bsg	FBgn0261822	FBtr0079570,FBtr0079574,FBtr0079567, FBtr0079566	457	944	5,02225E-59	**-1,04659**
CkIIbeta	FBgn0000259	FBtr0073558	3149	6510	0,00000E+00	**-1,04776**
Gef26	FBgn0021873	FBtr0300039	176	364	2,37699E-23	**-1,04836**
heph	FBgn0011224	FBtr0300268,FBtr0300269	234	484	1,08720E-30	**-1,04850**
Bsg	FBgn0261822	FBtr0079573	474	981	1,88276E-61	**-1,04937**
gro	FBgn0001139	FBtr0084962,FBtr0084963,FBtr0084964, FBtr0084965,FBtr0084966,FBtr0302951, FBtr0302952	926	1919	6,28211E-120	**-1,05127**
l(2)s5379	FBgn0010704	FBtr0100673	1785	3701	8,81470E-231	**-1,05199**
l(2)s5379	FBgn0010704	FBtr0077840	1785	3702	6,09765E-231	**-1,05238**
CG7971	FBgn0035253	FBtr0072806	283	587	2,53582E-37	**-1,05256**
hth	FBgn0001235	FBtr0082256,FBtr0082254,FBtr0082255, FBtr0082253,FBtr0301345,FBtr0301956	2636	5471	0,00000E+00	**-1,05345**
sdt	FBgn0261873	FBtr0089978,FBtr0100376,FBtr0111034, FBtr0089975,FBtr0100375,FBtr0089974, FBtr0308218,FBtr0308219,FBtr0308220	588	1224	2,93396E-77	**-1,05772**
lolal	FBgn0022238	FBtr0086776,FBtr0086777,FBtr0086778, FBtr0086779,FBtr0300483,FBtr0300484, FBtr0305270	1665	3469	3,68144E-218	**-1,05900**
pncr013:4	FBgn0262731	FBtr0091952	490	1021	9,36144E-65	**-1,05913**
CG6700	FBgn0032305	FBtr0080187	2046	4276	4,15016E-270	**-1,06346**
prominin-like	FBgn0026189	FBtr0073118,FBtr0306258	2132	4461	2,38759E-282	**-1,06516**
Pdk1	FBgn0020386	FBtr0072464,FBtr0072465	739	1548	1,19573E-98	**-1,06676**
CG13025	FBgn0036660	FBtr0075346	709	1486	9,81035E-95	**-1,06758**
Mitf	FBgn0263112	FBtr0307326,FBtr0307327,FBtr0307329, FBtr0307330	239	501	1,64678E-32	**-1,06780**
Bsg	FBgn0261822	FBtr0079568,FBtr0079569,FBtr0079571, FBtr0079572	537	1126	4,67877E-72	**-1,06821**
Adar	FBgn0026086	FBtr0070299,FBtr0070300,FBtr0100557, FBtr0307895,FBtr0305498	196	411	8,23339E-27	**-1,06828**
Cf2	FBgn0000286	FBtr0089647,FBtr0089648,FBtr0089649	528	1111	1,58197E-71	**-1,07325**
bun	FBgn0259176	FBtr0299656	469	987	1,25064E-63	**-1,07346**
CrebA	FBgn0004396	FBtr0075557	768	1618	4,33277E-104	**-1,07503**
unk	FBgn0004395	FBtr0305573	1399	2953	7,62875E-190	**-1,07779**
cib	FBgn0026084	FBtr0070641,FBtr0307206	10217	21574	0,00000E+00	**-1,07832**
Antp	FBgn0260642	FBtr0081654,FBtr0081655	477	1009	1,57313E-65	**-1,08087**
Gs1	FBgn0001142	FBtr0078115	259	548	5,69284E-36	**-1,08122**
Rbp2	FBgn0262734	FBtr0074279,FBtr0074280	8823	18674	0,00000E+00	**-1,08169**
CG8116	FBgn0037614	FBtr0300543	164	348	3,07728E-23	**-1,08539**
CG4502	FBgn0031896	FBtr0079417	384	816	1,38407E-53	**-1,08746**
CG40196	FBgn0058196	FBtr0113831	253	538	1,12422E-35	**-1,08847**
Sdc	FBgn0010415	FBtr0071707,FBtr0301557	455	968	1,56036E-63	**-1,08914**
Sdc	FBgn0010415	FBtr0071706,FBtr0071705,FBtr0273206, FBtr0273207	453	964	2,24892E-63	**-1,08952**
pncr013:4	FBgn0262731	FBtr0303019,FBtr0303020,FBtr0303021	221	471	1,85836E-31	**-1,09168**
Dyrk3	FBgn0027101	FBtr0100406	621	1324	7,11001E-87	**-1,09224**
CG4502	FBgn0031896	FBtr0079416	379	809	1,56795E-53	**-1,09394**
Gs1	FBgn0001142	FBtr0300568	221	474	5,98769E-32	**-1,10084**
Parp	FBgn0010247	FBtr0113885	2203	4726	5,8334E-312	**-1,10115**
CG12054	FBgn0039831	FBtr0305118	1800	3863	3,19430E-255	**-1,10172**
gish	FBgn0250823	FBtr0301304,FBtr0083262,FBtr0083261, FBtr0083264,FBtr0100331,FBtr0100332, FBtr0100333,FBtr0305071,FBtr0305072	160	344	2,05306E-23	**-1,10434**
CG11180	FBgn0034528	FBtr0086254	678	1463	6,48815E-98	**-1,10957**
		FBtr0301360,FBtr0301361	3239	6999	0,00000E+00	**-1,11160**
		FBtr0303296	3239	6999	0,00000E+00	**-1,11160**
CG1115	FBgn0037299	FBtr0305001	148	320	3,52764E-22	**-1,11247**
Cf2	FBgn0000286	FBtr0304887	539	1166	1,49484E-78	**-1,11321**
hth	FBgn0001235	FBtr0100454	4826	10441	0,00000E+00	**-1,11336**
A2bp1	FBgn0052062	FBtr0305093,FBtr0305096	1616	3499	1,32866E-234	**-1,11452**
CG1115	FBgn0037299	FBtr0078777,FBtr0305002	168	364	3,64102E-25	**-1,11548**
akirin	FBgn0082598	FBtr0302544	4448	9640	0,00000E+00	**-1,11588**

myoglianin	FBgn0026199	FBtr0089092,FBtr0089093,FBtr0089094, FBtr0089095	1167	2537	2,67258E-171	**-1,12032**
how	FBgn0017397	FBtr0084177	1913	4160	9,86398E-281	**-1,12075**
UbcD2	FBgn0015320	FBtr0080115,FBtr0080116	870	1897	7,55026E-129	**-1,12463**
shi	FBgn0003392	FBtr0111036,FBtr0111037,FBtr0074118,FBtr0074119,FBtr0074121,FBtr0074122,FBtr0301597	596	1300	1,27784E-88	**-1,12513**
cals	FBgn0039928	FBtr0089207	142	310	9,62498E-22	**-1,12638**
R	FBgn0004636	FBtr0072867,FBtr0303154	5306	11589	0,00000E+00	**-1,12706**
Mkk4	FBgn0024326	FBtr0300443	788	1722	1,43309E-117	**-1,12782**
Nos	FBgn0011676	FBtr0100484	513	1126	1,30630E-77	**-1,13418**
Sh3beta	FBgn0035772	FBtr0302548	225	495	1,00933E-34	**-1,13750**
bowl	FBgn0004893	FBtr0077490,FBtr0077491,FBtr0077492, FBtr0307026,FBtr0307027,FBtr0307028, FBtr0307029	539	1186	4,81152E-82	**-1,13775**
Mkk4	FBgn0024326	FBtr0081892	827	1820	1,76362E-125	**-1,13798**
ogre	FBgn0004646	FBtr0071036	1968	4345	2,41522E-300	**-1,14263**
CG5059	FBgn0037007	FBtr0078216,FBtr0078217,FBtr0078218, FBtr0078219	1265	2794	1,97969E-193	**-1,14319**
Dsp1	FBgn0011764	FBtr0089262,FBtr0289960	1896	4190	3,74759E-290	**-1,14399**
Dyb	FBgn0033739	FBtr0087930,FBtr0087929,FBtr0100298, FBtr0305077	275	608	8,95658E-43	**-1,14464**
TBPH	FBgn0025790	FBtr0089624,FBtr0089626,FBtr0089627, FBtr0301643,FBtr0301644	161	356	2,60566E-25	**-1,14482**
Ptp10D	FBgn0004370	FBtr0073524,FBtr0273235	861	1904	2,33165E-132	**-1,14495**
eIF5	FBgn0030719	FBtr0074147,FBtr0074146,FBtr0074144, FBtr0074148,FBtr0074145,FBtr0074150	164	363	7,16997E-26	**-1,14627**
prominin-like	FBgn0026189	FBtr0073119,FBtr0073120	966	2144	1,12338E-149	**-1,15021**
CkIalpha	FBgn0015024	FBtr0073681,FBtr0073682,FBtr0300380	5036	11209	0,00000E+00	**-1,15431**
CR42723	FBgn0261640	FBtr0303010	237	528	1,27183E-37	**-1,15565**
Galpha49B	FBgn0004435	FBtr0087829,FBtr0087830	417	930	7,93344E-66	**-1,15718**
kn	FBgn0001319	FBtr0087465,FBtr0112810,FBtr0301400	257	575	2,83418E-41	**-1,16179**
Bsg	FBgn0261822	FBtr0079570,FBtr0079568,FBtr0079569, FBtr0079573,FBtr0079567,FBtr0079571, FBtr0079572,FBtr0079566	1764	3947	9,57366E-279	**-1,16191**
tup	FBgn0003896	FBtr0081112	224	503	2,47060E-36	**-1,16706**
qkr58E-3	FBgn0022984	FBtr0290254	1092	2461	5,28351E-176	**-1,17227**
CG8500	FBgn0037754	FBtr0082139	192	433	1,25381E-31	**-1,17326**
mrj	FBgn0034091	FBtr0087193,FBtr0087194,FBtr0087195, FBtr0087196,FBtr0113084,FBtr0306652, FBtr0306653	623	1405	7,10353E-101	**-1,17327**
TotA	FBgn0028396	FBtr0083971	117	264	1,45357E-19	**-1,17403**
Gs1	FBgn0001142	FBtr0078114	224	506	7,69237E-37	**-1,17564**
how	FBgn0017397	FBtr0301401	1792	4049	3,78457E-290	**-1,17599**
CG11727	FBgn0262740	FBtr0073547	840	1902	4,30508E-137	**-1,17906**
lin19	FBgn0015509	FBtr0088846	140	317	1,95354E-23	**-1,17906**
tup	FBgn0003896	FBtr0081111	222	503	7,20278E-37	**-1,18000**
pho	FBgn0002521	FBtr0089204,FBtr0089205	1815	4113	4,29367E-296	**-1,18022**
Mhcl	FBgn0026059	FBtr0083231	176	399	1,89475E-29	**-1,18081**
CG11266	FBgn0031883	FBtr0089638,FBtr0089639,FBtr0089635	228	517	8,36609E-38	**-1,18113**
CaMKI	FBgn0016126	FBtr0089065	552	1255	2,73006E-91	**-1,18495**
Hr46	FBgn0000448	FBtr0306346	405	922	2,46024E-67	**-1,18684**
CG5065	FBgn0034145	FBtr0087108,FBtr0302206	161	367	2,40451E-27	**-1,18872**
CG4768	FBgn0030790	FBtr0074332	470	1073	1,10401E-78	**-1,19092**
mRpS5	FBgn0044510	FBtr0111147,FBtr0111146	489	1117	6,09039E-82	**-1,19172**
tna	FBgn0026160	FBtr0076267	876	2005	6,77293E-147	**-1,19460**
CG11266	FBgn0031883	FBtr0089637	228	522	7,67132E-39	**-1,19502**
tal-2A	FBgn0259731	FBtr0299998	442	1015	5,74508E-75	**-1,19936**
tal-1A	FBgn0259730	FBtr0299997	442	1015	5,74508E-75	**-1,19936**
tal-3A	FBgn0259732	FBtr0299999	442	1015	5,74508E-75	**-1,19936**
tal-AA	FBgn0259733	FBtr0299996	442	1015	5,74508E-75	**-1,19936**
mt:Cyt-b	FBgn0013678	FBtr0100884	14497	33560	0,00000E+00	**-1,21099**
Pdk1	FBgn0020386	FBtr0072470,FBtr0072471,FBtr0072466, FBtr0072467	895	2088	2,96546E-157	**-1,22216**
CG30343	FBgn0050343	FBtr0308205	202	474	1,19602E-36	**-1,23053**
qkr58E-3	FBgn0022984	FBtr0307214	1156	2715	2,27542E-206	**-1,23181**
CG11727	FBgn0262740	FBtr0300733	663	1561	2,60548E-119	**-1,23539**
CR41597	FBgn0085810	FBtr0114264	218	514	7,05753E-40	**-1,23744**
CG4662	FBgn0038735	FBtr0083814	119	281	2,77533E-22	**-1,23961**
CG17715	FBgn0041004	FBtr0111249	893	2109	1,11451E-161	**-1,23983**
E2f	FBgn0011766	FBtr0084119	306	723	5,50342E-56	**-1,24046**
Zyx	FBgn0011642	FBtr0089210,FBtr0089213,FBtr0089215	256	605	6,64457E-47	**-1,24079**
Zyx	FBgn0011642	FBtr0089211,FBtr0089212,FBtr0089214, FBtr0089216	297	702	2,62206E-54	**-1,24101**
CG17683	FBgn0262115	FBtr0111301	158	374	2,17093E-29	**-1,24311**
mt:CoIII	FBgn0013676	FBtr0100868	33823	80212	0,00000E+00	**-1,24581**
AP-1gamma	FBgn0030089	FBtr0071295,FBtr0071297,FBtr0071298, FBtr0112965	99	235	8,37405E-19	**-1,24716**
CG15923	FBgn0038814	FBtr0113254	877	2089	3,57269E-162	**-1,25216**
zip	FBgn0005634	FBtr0072399,FBtr0100466,FBtr0100467, FBtr0302572,FBtr0302573,FBtr0302574, FBtr0302575,FBtr0306576	1131	2712	1,96335E-212	**-1,26176**
gus	FBgn0026238	FBtr0089755,FBtr0089752,FBtr0089754, FBtr0089757,FBtr0089753,FBtr0089756	558	1339	2,36463E-105	**-1,26282**
CG17715	FBgn0041004	FBtr0111247	621	1491	2,61716E-117	**-1,26362**
gro	FBgn0001139	FBtr0305047	1898	4562	0,00000E+00	**-1,26519**
PRL-1	FBgn0024734	FBtr0080857,FBtr0080856	3306	8009	0,00000E+00	**-1,27654**
myoglianin	FBgn0026199	FBtr0089095	110	267	5,02911E-22	**-1,27934**
syd	FBgn0024187	FBtr0076769,FBtr0300412,FBtr0300413	129	314	8,16745E-26	**-1,28339**
Pi4KIIalpha	FBgn0037339	FBtr0078755,FBtr0078756,FBtr0078757	111	272	1,20779E-22	**-1,29305**
Hr46	FBgn0000448	FBtr0306345	864	2122	7,22224E-172	**-1,29632**
PMCA	FBgn0259214	FBtr0304046,FBtr0304047,FBtr0304048, FBtr0304049,FBtr0300554,FBtr0300555, FBtr0300556,FBtr0300557	306	753	1,07181E-61	**-1,29912**
CG8419	FBgn0031999	FBtr0079589	164	404	1,56062E-33	**-1,30066**
sky	FBgn0032901	FBtr0081445	310	764	1,08040E-62	**-1,30130**

CG12567	FBgn0039958	FBtr0113704,FBtr0113705,FBtr0300702, FBtr0300703,FBtr0300704	533	1316	1,19764E-107	**-1,30395**
plexA	FBgn0025741	FBtr0089225,FBtr0089226,FBtr0089223	230	573	7,52185E-48	**-1,31690**
CadN	FBgn0015609	FBtr0081016,FBtr0081015,FBtr0100313, FBtr0100315	170	424	9,70881E-36	**-1,31853**
gish	FBgn0250823	FBtr0083262,FBtr0083261,FBtr0083265, FBtr0083264,FBtr0100332,FBtr0100333	924	2311	8,11501E-192	**-1,32255**
grk	FBgn0001137	FBtr0079708	135	338	9,55854E-29	**-1,32406**
lin19	FBgn0015509	FBtr0088845	121	304	5,12092E-26	**-1,32906**
CG6357	FBgn0033875	FBtr0087638	267	671	1,28653E-56	**-1,32947**
CrebA	FBgn0004396	FBtr0075558	1399	3520	2,46196E-294	**-1,33118**
Zyx	FBgn0011642	FBtr0089210,FBtr0089213	181	457	5,03067E-39	**-1,33620**
CG17698	FBgn0040056	FBtr0111168,FBtr0111167	372	941	1,10358E-79	**-1,33889**
dbr	FBgn0067779	FBtr0306536,FBtr0306538	152	386	2,55554E-33	**-1,34453**
qkr54B	FBgn0022987	FBtr0306248	707	1804	7,07324E-154	**-1,35142**
vn	FBgn0003984	FBtr0077082	283	725	1,26624E-62	**-1,35718**
Ptp10D	FBgn0004370	FBtr0073525,FBtr0073522	366	942	1,05130E-81	**-1,36388**
bw	FBgn0000241	FBtr0072117	134	345	2,44673E-30	**-1,36436**
eIF-4B	FBgn0020660	FBtr0113679	182	469	4,02730E-41	**-1,36565**
H	FBgn0001169	FBtr0083915,FBtr0083916	225	580	1,15099E-50	**-1,36613**
crq	FBgn0015924	FBtr0078087	644	1670	1,60109E-145	**-1,37472**
CG17683	FBgn0262115	FBtr0111298,FBtr0111299	94	244	6,34165E-22	**-1,37615**
Zyx	FBgn0011642	FBtr0089212,FBtr0089215	182	476	1,95322E-42	**-1,38702**
Lsd-1	FBgn0039114	FBtr0084455,FBtr0084456,FBtr0084457	76	199	3,26991E-18	**-1,38870**
Zyx	FBgn0011642	FBtr0089211,FBtr0089216	181	474	2,23363E-42	**-1,38890**
inx2	FBgn0027108	FBtr0071005,FBtr0071006	15078	39521	0,00000E+00	**-1,39017**
Muc11A	FBgn0052656	FBtr0089803	144	378	4,09263E-34	**-1,39232**
Mnt	FBgn0023215	FBtr0307278,FBtr0301823	803	2113	1,68494E-187	**-1,39582**
Hsc70-3	FBgn0001218	FBtr0073608	85	225	8,05487E-21	**-1,40439**
Pur-alpha	FBgn0022361	FBtr0089996,FBtr0089995,FBtr0089994, FBtr0089993	514	1363	2,88923E-122	**-1,40695**
CG30343	FBgn0050343	FBtr0088594	293	778	3,55503E-70	**-1,40887**
sgg	FBgn0003371	FBtr0070475,FBtr0070476,FBtr0301966	1341	3564	7,5676E-320	**-1,41019**
Cklalpha	FBgn0015024	FBtr0073680	5372	14301	0,00000E+00	**-1,41258**
CG17528	FBgn0261387	FBtr0111274	85	227	3,35784E-21	**-1,41716**
CaMKI	FBgn0016126	FBtr0089067	181	484	1,89474E-44	**-1,41902**
stai	FBgn0051641	FBtr0304908	2437	6517	0,00000E+00	**-1,41910**
stai	FBgn0051641	FBtr0079196,FBtr0079197,FBtr0079198	2424	6490	0,00000E+00	**-1,42083**
sgg	FBgn0003371	FBtr0070471,FBtr0070472,FBtr0070473, FBtr0070466,FBtr0070468,FBtr0070469, FBtr0070470	1345	3607	0,00000E+00	**-1,42319**
CG42258	FBgn0259143	FBtr0299558,FBtr0299559	426	1149	2,40675E-105	**-1,43145**
lola	FBgn0005630	FBtr0089365,FBtr0089347,FBtr0089361, FBtr0089360,FBtr0089366,FBtr0089355, FBtr0089354,FBtr0089346,FBtr0089345, FBtr0089364	192	518	5,82406E-48	**-1,43185**
zip	FBgn0005634	FBtr0072398	1540	4169	0,00000E+00	**-1,43677**
fwe	FBgn0261722	FBtr0075543	91	250	4,98808E-24	**-1,45799**
		FBtr0082858	13736	37797	0,00000E+00	**-1,46031**
Antp	FBgn0260642	FBtr0081647,FBtr0081648,FBtr0081649, FBtr0081650,FBtr0081651,FBtr0081652, FBtr0081653,FBtr0081656	672	1855	1,08982E-174	**-1,46489**
pAbp	FBgn0261619	FBtr0086738	446	1240	6,88759E-118	**-1,47522**
pAbp	FBgn0261619	FBtr0086743,FBtr0086739	446	1240	6,88759E-118	**-1,47522**
CaMKII	FBgn0004624	FBtr0089218,FBtr0089219,FBtr0089217	554	1545	2,71559E-147	**-1,47965**
CaMKII	FBgn0004624	FBtr0100146,FBtr0100147,FBtr0100148, FBtr0300378	554	1546	1,74278E-147	**-1,48058**
CG8745	FBgn0036381	FBtr0075801	96	268	3,08241E-26	**-1,48113**
CR43241	FBgn0262886	FBtr0306297	98	274	9,98207E-27	**-1,48332**
Hr46	FBgn0000448	FBtr0088366,FBtr0088368,FBtr0112799, FBtr0302438	659	1848	2,53620E-177	**-1,48761**
CaMKI	FBgn0016126	FBtr0089069	70	198	8,66801E-20	**-1,50007**
Mbs	FBgn0005536	FBtr0112850,FBtr0112851,FBtr0112852,F Btr0301472,FBtr0301473,FBtr0301575,F Btr0308213,FBtr0308214,FBtr0308215,F Btr0308216	466	1340	1,53076E-132	**-1,52383**
CG41520	FBgn0087011	FBtr0114111,FBtr0114112,FBtr0302581,F Btr0302582	134	390	1,27207E-39	**-1,54124**
Fas3	FBgn0000636	FBtr0081051,FBtr0081052	2666	7762	0,00000E+00	**-1,54175**
CkIIbeta	FBgn0000259	FBtr0073562	63	185	1,95058E-19	**-1,55410**
Ank	FBgn0011747	FBtr0089174	157	465	6,28408E-48	**-1,56647**
CG17715	FBgn0041004	FBtr0111244,FBtr0111246,FBtr0111245,F Btr0111248,FBtr0306550,FBtr0306551	200	593	5,68733E-61	**-1,56803**
dbr	FBgn0067779	FBtr0078100,FBtr0306537,FBtr0306539	180	540	1,99059E-56	**-1,58496**
Nrg	FBgn0002968	FBtr0071207,FBtr0071209,FBtr0301762, FBtr0301764,FBtr0305914	1144	3445	0,00000E+00	**-1,59042**
Df31	FBgn0022893	FBtr0085919,FBtr0085920	27141	81808	0,00000E+00	**-1,59177**
Df31	FBgn0022893	FBtr0100293	27249	82324	0,00000E+00	**-1,59511**
pallidin	FBgn0036192	FBtr0300097,FBtr0300725,FBtr0300726	93	282	4,31882E-30	**-1,60039**
Cam	FBgn0000253	FBtr0088001,FBtr0088002	7691	23442	0,00000E+00	**-1,60785**
Galpha49B	FBgn0004435	FBtr0304955	1132	3452	0,00000E+00	**-1,60856**
mt:CoII	FBgn0013675	FBtr0100863	10541	32317	0,00000E+00	**-1,61628**
gish	FBgn0250823	FBtr0083263	1002	3086	0,00000E+00	**-1,62286**
Fbp1	FBgn0000639	FBtr0075750	8274	25614	0,00000E+00	**-1,63028**
Fbp1	FBgn0000639	FBtr0075749	8273	25613	0,00000E+00	**-1,63039**
Akap200	FBgn0027932	FBtr0079665,FBtr0079667	5459	16929	0,00000E+00	**-1,63279**
Akap200	FBgn0027932	FBtr0079664,FBtr0079666	5459	16932	0,00000E+00	**-1,63304**
eIF-4B	FBgn0020660	FBtr0113680	70	218	5,21822E-24	**-1,63890**
Mnt	FBgn0023215	FBtr0307277,FBtr0301822	882	2748	7,28101E-296	**-1,63953**
CG7115	FBgn0027515	FBtr0079523	98	306	1,38606E-33	**-1,64268**
CG11266	FBgn0031883	FBtr0089639,FBtr0089635	63	197	6,85753E-22	**-1,64477**
Pdp1	FBgn0016694	FBtr0076775,FBtr0076776,FBtr0076777, FBtr0076780,FBtr0076782,FBtr0300499, FBtr0300500	194	608	2,88267E-66	**-1,64801**
Rfabg	FBgn0087002	FBtr0089188	676	2127	1,72179E-231	**-1,65372**
CG7367	FBgn0031976	FBtr0306002	102	323	5,14858E-36	**-1,66297**
Pdp1	FBgn0016694	FBtr0306535	195	623	3,12539E-69	**-1,67576**

Hr39	FBgn0261239	FBtr0081480	302	966	3,61776E-107	**-1,67747**
mt:CoI	FBgn0013674	FBtr0100861	45828	146749	0,00000E+00	**-1,67905**
CG7367	FBgn0031976	FBtr0301128	143	460	1,05238E-51	**-1,68562**
Ank	FBgn0011747	FBtr0089173,FBtr0089171,FBtr0089172, FBtr0300497,FBtr0300498	133	429	1,99072E-48	**-1,68955**
swi2	FBgn0034262	FBtr0086889	88	284	2,22491E-32	**-1,69032**
Pabp2	FBgn0005648	FBtr0088786	143	462	3,99826E-52	**-1,69188**
Cam	FBgn0000253	FBtr0304963,FBtr0304964	8288	26840	0,00000E+00	**-1,69529**
Pdp1	FBgn0016694	FBtr0076776,FBtr0076777,FBtr0300499, FBtr0306535	80	265	7,38196E-31	**-1,72792**
Eph	FBgn0025936	FBtr0089082,FBtr0089083,FBtr0089084	370	1229	2,35036E-141	**-1,73189**
Akap200	FBgn0027932	FBtr0079665	95	324	8,68849E-39	**-1,76999**
Galpha49B	FBgn0004435	FBtr0087829,FBtr0087830,FBtr0087831, FBtr0087833,FBtr0087834,FBtr0304954	710	2445	1,24882E-290	**-1,78394**
ventrally-expressed-protein-D	FBgn0053200	FBtr0307213	48	167	8,87292E-21	**-1,79874**
H	FBgn0001169	FBtr0083914,FBtr0083917	541	1911	1,09283E-232	**-1,82063**
Eph	FBgn0025936	FBtr0089086	303	1076	8,65538E-132	**-1,82829**
myoglianin	FBgn0026199	FBtr0089092,FBtr0089093	105	373	3,33842E-46	**-1,82879**
CG14526	FBgn0027578	FBtr0301945	46	164	9,10662E-21	**-1,83399**
CG2225	FBgn0032957	FBtr0304885	133	476	5,49543E-59	**-1,83954**
		FBtr0076660	623	2241	3,80748E-277	**-1,84684**
Hr39	FBgn0261239	FBtr0081479,FBtr0081481	881	3193	0,00000E+00	**-1,85770**
CG32016	FBgn0052016	FBtr0089232	84	307	5,03507E-39	**-1,86978**
CG9894	FBgn0031453	FBtr0307080	4227	15511	0,00000E+00	**-1,87559**
Cyp6g1	FBgn0025454	FBtr0087992	66	243	3,09904E-31	**-1,88042**
CG9894	FBgn0031453	FBtr0077713,FBtr0077714,FBtr0307080	1452	5391	0,00000E+00	**-1,89251**
pncr013:4	FBgn0262731	FBtr0303019	94	351	2,11269E-45	**-1,90074**
UBL3	FBgn0026076	FBtr0074175	54	204	7,39942E-27	**-1,91754**
CG10417	FBgn0033021	FBtr0086092	90	341	1,07016E-44	**-1,92177**
CG9894	FBgn0031453	FBtr0077713,FBtr0077714	2930	11197	0,00000E+00	**-1,93414**
Fbp2	FBgn0000640	FBtr0079808	817	3125	0,00000E+00	**-1,93545**
CG32016	FBgn0052016	FBtr0089229	82	321	2,67820E-43	**-1,96888**
CG17471	FBgn0039924	FBtr0100544	60	238	1,42722E-32	**-1,98793**
CG17471	FBgn0039924	FBtr0100543	60	241	2,92884E-33	**-2,00600**
CG32016	FBgn0052016	FBtr0089233	105	424	2,21138E-58	**-2,01367**
CG5958	FBgn0031913	FBtr0079466	32	135	1,47629E-19	**-2,07682**
CR43241	FBgn0262886	FBtr0306296	34	149	4,12348E-22	**-2,13171**
nimC2	FBgn0028939	FBtr0080589,FBtr0080588	69	304	7,11246E-45	**-2,13940**
CG12990	FBgn0030859	FBtr0074479	49	226	1,21449E-34	**-2,20547**
Rbp1-like	FBgn0030479	FBtr0073790	215	997	5,50079E-151	**-2,21326**
TpnC25D	FBgn0031692	FBtr0079063	38	178	9,44630E-28	**-2,22781**
E2f	FBgn0011766	FBtr0084118	120	569	1,13696E-87	**-2,24539**
CG10417	FBgn0033021	FBtr0086091	100	485	4,95101E-76	**-2,27798**
CG40196	FBgn0058196	FBtr0113829,FBtr0113830,FBtr0301121	53	258	7,55758E-41	**-2,28331**
CG8979	FBgn0033669	FBtr0088052	64	316	2,18492E-50	**-2,30378**
Act5C	FBgn0000042	FBtr0070822,FBtr0100662,FBtr0100663	1371	7183	0,00000E+00	**-2,38936**
UBL3	FBgn0026076	FBtr0074174,FBtr0300787	94	533	7,59301E-92	**-2,50340**
Pabp2	FBgn0005648	FBtr0088785	941	5443	0,00000E+00	**-2,53214**
CG2233	FBgn0029990	FBtr0071123	192	1126	3,64705E-197	**-2,55203**
mt:tRNA:G	FBgn0013694	FBtr0100869	18	115	1,31510E-21	**-2,67557**
mt:tRNA:Y	FBgn0013710	FBtr0100860	15	96	3,07578E-18	**-2,67807**
CG3999	FBgn0037801	FBtr0082225	21	135	2,00174E-25	**-2,68450**
CG10006	FBgn0036461	FBtr0113171	19	124	1,43049E-23	**-2,70627**
CG32016	FBgn0052016	FBtr0089234	33	217	1,13543E-40	**-2,71716**
Zyx	FBgn0011642	FBtr0089210,FBtr0089211,FBtr0089212	39	257	3,55007E-48	**-2,72022**
Zyx	FBgn0011642	FBtr0089213,FBtr0089215,FBtr0089216	39	257	3,55007E-48	**-2,72022**
Zyx	FBgn0011642	FBtr0089214	38	255	2,51803E-48	**-2,74643**
mt:ND4	FBgn0262952	FBtr0100879	1500	11843	0,00000E+00	**-2,98100**
mt:tRNA:W	FBgn0013709	FBtr0100858	14	112	2,85095E-23	**-3,00000**
pncr013:4	FBgn0262731	FBtr0303020,FBtr0303021	77	623	1,71565E-127	**-3,01630**
mt:ND5	FBgn0013684	FBtr0100877	3108	26146	0,00000E+00	**-3,07253**
mt:ND3	FBgn0013681	FBtr0100870	99	871	2,46348E-184	**-3,13717**
CG10514	FBgn0039312	FBtr0084864	11	132	1,97103E-31	**-3,58496**
CG10513	FBgn0039311	FBtr0114505	6	78	2,91618E-19	**-3,70044**
CG11892	FBgn0039313	FBtr0089644	18	235	4,90467E-57	**-3,70659**
CG11892	FBgn0039313	FBtr0089645	18	235	4,90467E-57	**-3,70659**
mt:tRNA:C	FBgn0013690	FBtr0100859	11	144	3,36099E-35	**-3,71049**
CG32016	FBgn0052016	FBtr0089230,FBtr0089231	10	133	1,17438E-32	**-3,73335**
mt:tRNA:L:UUR	FBgn0013699	FBtr0100862	7	95	1,26312E-23	**-3,76250**
CG9259	FBgn0032913	FBtr0081464	16	232	7,35809E-58	**-3,85798**
mt:ATPase6	FBgn0013672	FBtr0100867	2522	36678	0,00000E+00	**-3,86227**
CG40351	FBgn0040022	FBtr0302248	4	75	4,45652E-20	**-4,22882**
CG40351	FBgn0040022	FBtr0113869,FBtr0302244	4	75	4,45652E-20	**-4,22882**
CG40351	FBgn0040022	FBtr0302243	4	93	6,84826E-26	**-4,53916**
CG40351	FBgn0040022	FBtr0113870,FBtr0113871	6	147	5,26998E-41	**-4,61471**
CG16727	FBgn0038719	FBtr0083780	15	386	7,41482E-108	**-4,68557**
mt:ND1	FBgn0013679	FBtr0100886	365	9585	0,00000E+00	**-4,71481**
CG3292	FBgn0034710	FBtr0071783	16	462	2,51399E-131	**-4,85175**
mt:ATPase8	FBgn0013673	FBtr0100866	567	16433	0,00000E+00	**-4,85710**
mt:lrRNA	FBgn0013686	FBtr0100888	32368	975604	0,00000E+00	**-4,91366**
mt:tRNA:P	FBgn0013702	FBtr0100882	2	62	3,78452E-18	**-4,95420**
Fst	FBgn0037724	FBtr0082101	12	374	1,64425E-107	**-4,96193**
mt:ND2	FBgn0013680	FBtr0100857	151	5273	0,00000E+00	**-5,12600**
CG3264	FBgn0034712	FBtr0071781	11	399	6,11024E-117	**-5,18081**
CG10505	FBgn0034612	FBtr0071619	4	146	5,00948E-43	**-5,18982**
CG7882	FBgn0033047	FBtr0085966	2	81	2,62446E-24	**-5,33985**
Mur18B	FBgn0030999	FBtr0074672	4	182	8,03058E-55	**-5,50779**
mt:ND6	FBgn0013685	FBtr0100883	66	3640	0,00000E+00	**-5,78533**
mt:srRNA	FBgn0013688	FBtr0100890	5	357	1,91917E-111	**-6,15785**
mt:tRNA:L:CUN	FBgn0013698	FBtr0100887	18	1386	0,00000E+00	**-6,26679**
CG17752	FBgn0038718	FBtr0083779	2	157	2,08322E-49	**-6,29462**
mt:ND4L	FBgn0013683	FBtr0100880	33	2698	0,00000E+00	**-6,35328**
CG42235	FBgn0250757	FBtr0290137,FBtr0290138,FBtr0290139, FBtr0290140,FBtr0290141	0	121	8,77010E-41	**-Infinity**

CG42235	FBgn0250757	FBtr0290139		0	104	4,86609E-35	**-Infinity**
CG42235	FBgn0250757	FBtr0290140		0	92	5,45626E-31	**-Infinity**
CG2187	FBgn0017448	FBtr0085861,FBtr0303376		0	60	3,18975E-20	**-Infinity**
