# Supplementary material for: New Partners in Regulation of Gene Expression: The Enhancer of Trithorax and Polycomb Corto Interacts with Methylated Ribosomal Protein L12 Via Its Chromodomain
Source: PLoS Genet. 2012 Oct 11;8(10):e1003006. doi: 10.1371/journal.pgen.1003006 (PMC3469418; doi:10.1371/journal.pgen.1003006)
Supplement: Table S6 — Genes down-regulated in sd::Gal4>UAS::RpL12-Myc vs sd::Gal4/+. FC: Fold Change. (PDF) [file pgen.1003006.s010.pdf]

**Table S6**

| Flybase Gene Symbol | Flybase ID Genes | Flybase ID Transcripts | Number of read sd::Gal4>UAS::RpL12-Myc | sd::Gal4/+ | Adjusted *P*-value | log$_2$(FC) |
|---|---|---|---|---|---|---|
| CkIIbeta | FBgn0000259 | FBtr0308084 | 151 | 302 | 4,25264E-20 | -1 |
| Tao-1 | FBgn0031030 | FBtr0303999 | 1664 | 3330 | 5,16696E-213 | -1,00087 |
| eyg | FBgn0000625 | FBtr0100170 | 241 | 483 | 1,41536E-31 | -1,00299 |
| CG14464 | FBgn0033000 | FBtr0299923 | 771 | 1546 | 1,48315E-99 | -1,00374 |
| eIF-4a | FBgn0001942 | FBtr0079176 | 6582 | 13214 | 0 | -1,00547 |
| Sh3beta | FBgn0035772 | FBtr0076873,FBtr0302547,FBtr0302548 | 1276 | 2566 | 1,35724E-165 | -1,00789 |
| Pkn | FBgn0020621 | FBtr0088601,FBtr0088603,FBtr0088604,FBtr0088605,FBtr0302604,FBtr0112897,FBtr0301511 | 1305 | 2625 | 1,62687E-169 | -1,00827 |
| gro | FBgn0001139 | FBtr0084962,FBtr0084963,FBtr0084964,FBtr0084965,FBtr0084966,FBtr0302951,FBtr0302952 | 953 | 1919 | 2,54324E-124 | -1,00981 |
| CG13025 | FBgn0036660 | FBtr0075346 | 736 | 1486 | 6,14527E-97 | -1,01366 |
| CG6084 | FBgn0086254 | FBtr0076139 | 285 | 576 | 3,98708E-38 | -1,01511 |
| Tsp42Ea | FBgn0029508 | FBtr0086171,FBtr0086172,FBtr0086173 | 183 | 370 | 9,90642E-25 | -1,01568 |
| CG6091 | FBgn0036180 | FBtr0273305,FBtr0273306 | 264 | 534 | 1,72904E-35 | -1,01630 |
| gish | FBgn0250823 | FBtr0301304,FBtr0083262,FBtr0083261,FBtr0083264,FBtr0100331,FBtr0100332,FBtr0100333,FBtr0305071,FBtr0305072 | 170 | 344 | 4,73567E-23 | -1,01687 |
| Pur-alpha | FBgn0022361 | FBtr0089995,FBtr0089993 | 201 | 407 | 2,77302E-27 | -1,01783 |
| Pur-alpha | FBgn0022361 | FBtr0089996,FBtr0089994 | 200 | 405 | 4,90691E-27 | -1,01792 |
| mask | FBgn0043884 | FBtr0084563 | 1369 | 2774 | 2,21313E-181 | -1,01885 |
| CG17698 | FBgn0040056 | FBtr0111166,FBtr0111168,FBtr0111167 | 568 | 1152 | 6,90771E-76 | -1,02018 |
| mRpS21 | FBgn0044511 | FBtr0082764 | 200 | 406 | 2,33562E-27 | -1,02148 |
| CG7337 | FBgn0031374 | FBtr0077811,FBtr0110882,FBtr0110883,FBtr0113011 | 232 | 471 | 1,62174E-31 | -1,02160 |
| eIF-4a | FBgn0001942 | FBtr0079178 | 6666 | 13540 | 0 | -1,02233 |
| gus | FBgn0026238 | FBtr0089755,FBtr0089752,FBtr0089754,FBtr0089753,FBtr0089756 | 192 | 390 | 2,97285E-26 | -1,02237 |
| vtd | FBgn0260987 | FBtr0113746 | 1837 | 3736 | 1,64579E-245 | -1,02414 |
| alph | FBgn0086361 | FBtr0085443 | 1613 | 3283 | 3,94811E-216 | -1,02527 |
| CG4662 | FBgn0038735 | FBtr0083814 | 138 | 281 | 3,03576E-19 | -1,02590 |
| itp | FBgn0035023 | FBtr0300187,FBtr0290219 | 508 | 1035 | 1,01188E-68 | -1,02673 |
| CG42342 | FBgn0259244 | FBtr0299895,FBtr0299897 | 158 | 322 | 5,91379E-22 | -1,02714 |
| 4EHP | FBgn0053100 | FBtr0300475,FBtr0303159 | 179 | 365 | 9,71646E-25 | -1,02794 |
| Imp | FBgn0262735 | FBtr0305150 | 3339 | 6817 | 0 | -1,02972 |
| Cf2 | FBgn0000286 | FBtr0089647,FBtr0089648,FBtr0089649 | 543 | 1111 | 2,97210E-74 | -1,03283 |
| CG13360 | FBgn0025620 | FBtr0070147 | 152 | 311 | 2,56838E-21 | -1,03284 |
| Csk | FBgn0262081 | FBtr0299728,FBtr0300548,FBtr0300546,FBtr0300547,FBtr0300549 | 1521 | 3114 | 4,36575E-207 | -1,03375 |
| Nedd4 | FBgn0259174 | FBtr0299642,FBtr0299645,FBtr0299647,FBtr0300519 | 676 | 1384 | 2,18570E-92 | -1,03375 |
| CG31121 | FBgn0051121 | FBtr0084723 | 285 | 584 | 2,00509E-39 | -1,03501 |
| eyg | FBgn0000625 | FBtr0075979 | 243 | 498 | 7,58304E-34 | -1,03519 |
| CG4612 | FBgn0035016 | FBtr0306136 | 3326 | 6819 | 0 | -1,03577 |
| Sdc | FBgn0010415 | FBtr0273207,FBtr0305897 | 4229 | 8671 | 0 | -1,03588 |
| CG4768 | FBgn0030790 | FBtr0074332 | 523 | 1073 | 5,00838E-72 | -1,03677 |
| Ranbp16 | FBgn0053180 | FBtr0303646 | 187 | 384 | 2,36964E-26 | -1,03807 |
| CG6700 | FBgn0032305 | FBtr0301710 | 1168 | 2400 | 1,10371E-160 | -1,03899 |
| bbg | FBgn0087007 | FBtr0273424,FBtr0273425,FBtr0273426 | 145 | 298 | 1,32010E-20 | -1,03926 |
| Bsg | FBgn0261822 | FBtr0079570,FBtr0079574,FBtr0079567,FBtr0079566 | 459 | 944 | 9,72579E-64 | -1,04029 |
| CG15535 | FBgn0039764 | FBtr0085629 | 138 | 284 | 9,87170E-20 | -1,04122 |
| qkr54B | FBgn0022987 | FBtr0086923,FBtr0086925 | 133 | 274 | 3,54544E-19 | -1,04275 |
| CaMKI | FBgn0016126 | FBtr0089069,FBtr0089063,FBtr0089064,FBtr0089065,FBtr0089066,FBtr0089067,FBtr0089068 | 2301 | 4751 | 4,7632E-320 | -1,04597 |
| CkIalpha | FBgn0015024 | FBtr0073680,FBtr0300380 | 845 | 1745 | 5,89448E-118 | -1,04620 |
| CG4612 | FBgn0035016 | FBtr0306137 | 3394 | 7019 | 0 | -1,04828 |
| Stlk | FBgn0046692 | FBtr0111111 | 899 | 1860 | 3,86625E-126 | -1,04891 |
| R | FBgn0004636 | FBtr0072867,FBtr0303154 | 5599 | 11589 | 0 | -1,04952 |
| Sdc | FBgn0010415 | FBtr0071706,FBtr0071705,FBtr0273206,FBtr0273207 | 465 | 964 | 5,25968E-66 | -1,05180 |
| CG8949 | FBgn0030812 | FBtr0074412,FBtr0304839 | 1469 | 3046 | 7,80014E-207 | -1,05208 |
| Sdc | FBgn0010415 | FBtr0071707,FBtr0301557 | 466 | 968 | 1,98752E-66 | -1,05468 |
| mub | FBgn0262737 | FBtr0304980 | 4130 | 8594 | 0 | -1,05719 |
| E2f | FBgn0011766 | FBtr0084119 | 347 | 723 | 4,71004E-50 | -1,05906 |
| CG17490 | FBgn0040009 | FBtr0111259 | 340 | 709 | 3,54907E-49 | -1,06025 |
| CG17883 | FBgn0040005 | FBtr0111291,FBtr0111292 | 603 | 1259 | 7,25742E-87 | -1,06205 |
| CaMKI | FBgn0016126 | FBtr0089069,FBtr0089066,FBtr0089067,FBtr0089068 | 239 | 500 | 5,08635E-35 | -1,06492 |
| Haspin | FBgn0046706 | FBtr0113784 | 954 | 1996 | 9,74021E-138 | -1,06505 |
| ewg | FBgn0005427 | FBtr0089441,FBtr0308208,FBtr0308209 | 720 | 1508 | 1,96217E-104 | -1,06657 |
| Bsg | FBgn0261822 | FBtr0079573 | 468 | 981 | 3,00647E-68 | -1,06774 |
| Scm | FBgn0003334 | FBtr0082102 | 734 | 1546 | 5,01310E-108 | -1,07469 |
| Rbp1-like | FBgn0030479 | FBtr0304001 | 1704 | 3590 | 6,59884E-250 | -1,07506 |
| Wnt4 | FBgn0010453 | FBtr0089291 | 224 | 472 | 1,55779E-33 | -1,07529 |
| CG7668 | FBgn0036929 | FBtr0074904,FBtr0074905 | 267 | 563 | 7,36560E-40 | -1,07630 |
| Patronin | FBgn0263197 | FBtr0300603,FBtr0273447,FBtr0086950,FBtr0300604 | 619 | 1306 | 1,28358E-91 | -1,07714 |
| nimC2 | FBgn0028939 | FBtr0080589,FBtr0080588 | 144 | 304 | 5,12689E-22 | -1,07800 |
| CG2316 | FBgn0039890 | FBtr0089152 | 204 | 431 | 7,94365E-31 | -1,07912 |
| fwe | FBgn0261722 | FBtr0075541 | 225 | 476 | 4,21292E-34 | -1,08104 |
| csw | FBgn0000382 | FBtr0070380 | 760 | 1608 | 4,82866E-113 | -1,08120 |
| l(2)s5379 | FBgn0010704 | FBtr0100673 | 1745 | 3701 | 1,76400E-260 | -1,08469 |
| kn | FBgn0001319 | FBtr0112809 | 430 | 912 | 1,19272E-64 | -1,08470 |
| l(2)s5379 | FBgn0010704 | FBtr0077840 | 1745 | 3702 | 1,19135E-260 | -1,08508 |
| knrl | FBgn0001323 | FBtr0078212 | 572 | 1215 | 3,69085E-86 | -1,08687 |
| Bsg | FBgn0261822 | FBtr0079568,FBtr0079569,FBtr0079571,FBtr0079572 | 530 | 1126 | 6,65705E-80 | -1,08714 |
| N | FBgn0004647 | FBtr0070507 | 2603 | 5537 | 0 | -1,08893 |
| smg | FBgn0016070 | FBtr0076550 | 242 | 515 | 4,22397E-37 | -1,08957 |
| tna | FBgn0026160 | FBtr0076267 | 942 | 2005 | 3,33567E-142 | -1,08980 |
| Gef26 | FBgn0021873 | FBtr0300039 | 171 | 364 | 1,90582E-26 | -1,08994 |
| CG18812 | FBgn0042135 | FBtr0088935,FBtr0088936,FBtr0088937 | 394 | 839 | 4,83348E-60 | -1,09048 |

| | | | | | | |
|---|---|---|---|---|---|---|
| ***mp*** | FBgn0260660 | FBtr0301105,FBtr0301106,FBtr0301107,FBtr0301108,FBtr0301109,FBtr0301110,FBtr0301111,FBtr0301113,FBtr0301114,FBtr0306638 | 161 | 343 | 5,23325E-25 | **-1,09115** |
| ***cic*** | FBgn0262582 | FBtr0305030 | 1618 | 3448 | 1,65213E-244 | **-1,09155** |
| ***Rfabg*** | FBgn0087002 | FBtr0089188 | 998 | 2127 | 4,77183E-151 | **-1,09171** |
| ***CkIIbeta*** | FBgn0000259 | FBtr0307896 | 2782 | 5931 | 0 | **-1,09215** |
| ***CG11180*** | FBgn0034528 | FBtr0086254 | 686 | 1463 | 2,42496E-104 | **-1,09265** |
| ***cib*** | FBgn0026084 | FBtr0070641,FBtr0307206 | 10104 | 21574 | 0 | **-1,09437** |
| ***Cf2*** | FBgn0000286 | FBtr0304887 | 546 | 1166 | 1,72396E-83 | **-1,09459** |
| ***Mmp1*** | FBgn0035049 | FBtr0306656 | 418 | 893 | 4,43436E-64 | **-1,09516** |
| ***Sap47*** | FBgn0013334 | FBtr0083204,FBtr0083205,FBtr0083206,FBtr0083207,FBtr0083208,FBtr0083209,FBtr0083210,FBtr0301655 | 897 | 1917 | 6,66569E-137 | **-1,09567** |
| ***rl*** | FBgn0003256 | FBtr0113701,FBtr0113702 | 334 | 714 | 2,12481E-51 | **-1,09608** |
| ***unk*** | FBgn0004395 | FBtr0305571,FBtr0305572 | 1040 | 2225 | 5,22280E-159 | **-1,09722** |
| ***smg*** | FBgn0016070 | FBtr0076551,FBtr0290104 | 242 | 519 | 8,88267E-38 | **-1,10073** |
| ***CG30343*** | FBgn0050343 | FBtr0308205 | 221 | 474 | 1,18113E-34 | **-1,10084** |
| ***Sdc*** | FBgn0010415 | FBtr0071706,FBtr0071707,FBtr0071705,FBtr0273206,FBtr0273207,FBtr0301557 | 137 | 294 | 7,93932E-22 | **-1,10164** |
| ***jim*** | FBgn0027339 | FBtr0078582,FBtr0078581 | 1235 | 2654 | 7,39003E-191 | **-1,10366** |
| ***CG1115*** | FBgn0037299 | FBtr0078777,FBtr0305002 | 169 | 364 | 5,69829E-27 | **-1,10692** |
| ***CG5059*** | FBgn0037007 | FBtr0078216,FBtr0078217,FBtr0078218,FBtr0078219 | 1297 | 2794 | 1,04596E-201 | **-1,10715** |
| ***CG18812*** | FBgn0042135 | FBtr0306239 | 380 | 819 | 1,14834E-59 | **-1,10786** |
| ***Mmp1*** | FBgn0035049 | FBtr0273263,FBtr0304005,FBtr0304008,FBtr0304009,FBtr0304010,FBtr0304011 | 411 | 888 | 6,48101E-65 | **-1,11142** |
| ***PMCA*** | FBgn0259214 | FBtr0304050 | 2618 | 5673 | 0 | **-1,11565** |
| ***dbr*** | FBgn0067779 | FBtr0078100,FBtr0306537,FBtr0306539 | 249 | 540 | 5,10906E-40 | **-1,11681** |
| ***zfh2*** | FBgn0004607 | FBtr0307167 | 1034 | 2245 | 3,41884E-164 | **-1,11848** |
| ***CG11486*** | FBgn0035397 | FBtr0072998,FBtr0072999,FBtr0073000,FBtr0073001,FBtr0073002,FBtr0073003,FBtr0072991,FBtr0072992,FBtr0113130,FBtr0113131 | 336 | 730 | 5,97164E-54 | **-1,11944** |
| ***akirin*** | FBgn0082598 | FBtr0302544 | 4432 | 9640 | 0 | **-1,12108** |
| ***grh*** | FBgn0259211 | FBtr0300539 | 2135 | 4644 | 0 | **-1,12113** |
| ***Pi4KIIalpha*** | FBgn0037339 | FBtr0078755,FBtr0078756,FBtr0078757 | 125 | 272 | 1,42300E-20 | **-1,12168** |
| ***Dsp1*** | FBgn0011764 | FBtr0089262,FBtr0289960 | 1923 | 4190 | 1,76311E-307 | **-1,12359** |
| ***Pdk1*** | FBgn0020386 | FBtr0072464,FBtr0072465 | 710 | 1548 | 3,46048E-114 | **-1,12451** |
| ***Sh3beta*** | FBgn0035772 | FBtr0302548 | 227 | 495 | 5,46115E-37 | **-1,12474** |
| ***mim*** | FBgn0053558 | FBtr0302577,FBtr0302578,FBtr0113470,FBtr0302035,FBtr0306614,FBtr0306615,FBtr0306616 | 110 | 240 | 2,01466E-18 | **-1,12553** |
| ***Nipped-B*** | FBgn0026401 | FBtr0111119,FBtr0111118 | 538 | 1175 | 5,24400E-87 | **-1,12698** |
| ***Eph*** | FBgn0025936 | FBtr0301285 | 861 | 1883 | 2,45807E-139 | **-1,12895** |
| ***sdt*** | FBgn0261873 | FBtr0308217 | 575 | 1260 | 9,51224E-94 | **-1,13179** |
| ***CG1115*** | FBgn0037299 | FBtr0305001 | 146 | 320 | 2,17364E-24 | **-1,13210** |
| ***CG9821*** | FBgn0037636 | FBtr0081931 | 11169 | 24493 | 0 | **-1,13287** |
| ***CG40351*** | FBgn0040022 | FBtr0113869,FBtr0113870,FBtr0113871,FBtr0302243,FBtr0302244,FBtr0302245,FBtr0302246,FBtr0302247,FBtr0302248 | 2333 | 5119 | 0 | **-1,13368** |
| ***PRL-1*** | FBgn0024734 | FBtr0080857,FBtr0080856 | 3641 | 8009 | 0 | **-1,13729** |
| ***Nipped-B*** | FBgn0026401 | FBtr0301454,FBtr0301455,FBtr0301456 | 528 | 1162 | 4,02952E-87 | **-1,13800** |
| ***mim*** | FBgn0053558 | FBtr0302579,FBtr0302034 | 159 | 350 | 9,81333E-27 | **-1,13833** |
| ***CG11266*** | FBgn0031883 | FBtr0089638,FBtr0089639,FBtr0089640 | 391 | 861 | 8,46007E-65 | **-1,13884** |
| ***CG40191*** | FBgn0058191 | FBtr0113825,FBtr0113826 | 113 | 249 | 2,42924E-19 | **-1,13982** |
| ***CG2698*** | FBgn0037536 | FBtr0308076 | 176 | 388 | 9,93943E-30 | **-1,14048** |
| ***CG8949*** | FBgn0030812 | FBtr0304840 | 1481 | 3271 | 4,99217E-245 | **-1,14316** |
| ***CG11266*** | FBgn0031883 | FBtr0089637,FBtr0089635,FBtr0089636 | 392 | 866 | 2,19011E-65 | **-1,14351** |
| ***Dyrk3*** | FBgn0027101 | FBtr0100404 | 167 | 369 | 2,25841E-28 | **-1,14377** |
| ***Dyrk3*** | FBgn0027101 | FBtr0100405 | 167 | 369 | 2,25841E-28 | **-1,14377** |
| ***fbl6*** | FBgn0033609 | FBtr0088179,FBtr0088180 | 429 | 949 | 8,63769E-72 | **-1,14543** |
| ***fs(1)h*** | FBgn0004656 | FBtr0071118,FBtr0071120,FBtr0071121,FBtr0071122 | 1051 | 2329 | 1,57576E-175 | **-1,14795** |
| ***CG15628*** | FBgn0031632 | FBtr0077384 | 846 | 1877 | 7,06022E-142 | **-1,14970** |
| ***CG40191*** | FBgn0058191 | FBtr0113827 | 114 | 253 | 9,37541E-20 | **-1,15010** |
| ***prominin-like*** | FBgn0026189 | FBtr0073119,FBtr0073120 | 965 | 2144 | 2,29549E-162 | **-1,15170** |
| ***sgg*** | FBgn0003371 | FBtr0111035,FBtr0070471,FBtr0070472,FBtr0070473,FBtr0070474,FBtr0070475,FBtr0070476,FBtr0070466,FBtr0070467,FBtr0070468,FBtr0070469,FBtr0070470,FBtr0301966,FBtr0302185 | 188 | 418 | 2,34799E-32 | **-1,15277** |
| ***mub*** | FBgn0262737 | FBtr0078468,FBtr0304981,FBtr0304982 | 2777 | 6185 | 0 | **-1,15525** |
| ***unk*** | FBgn0004395 | FBtr0084402 | 1094 | 2439 | 1,56421E-185 | **-1,15668** |
| ***CG6700*** | FBgn0032305 | FBtr0080187 | 1914 | 4276 | 0 | **-1,15967** |
| ***CG31360*** | FBgn0051360 | FBtr0083480 | 258 | 577 | 1,09425E-44 | **-1,16120** |
| ***CkIIbeta*** | FBgn0000259 | FBtr0073558 | 2904 | 6510 | 0 | **-1,16462** |
| ***Lis-1*** | FBgn0015754 | FBtr0087236,FBtr0087238,FBtr0087239,FBtr0304747 | 103 | 231 | 1,99239E-18 | **-1,16525** |
| ***mrj*** | FBgn0034091 | FBtr0087193,FBtr0087194,FBtr0087195,FBtr0087196,FBtr0113084,FBtr0306652,FBtr0306653 | 626 | 1405 | 3,59501E-108 | **-1,16634** |
| ***Bsg*** | FBgn0261822 | FBtr0079570,FBtr0079568,FBtr0079569,FBtr0079573,FBtr0079567,FBtr0079571,FBtr0079572,FBtr0079566 | 1757 | 3947 | 3,01030E-303 | **-1,16764** |
| ***prominin-like*** | FBgn0026189 | FBtr0073118,FBtr0306258 | 1979 | 4461 | 0 | **-1,17260** |
| ***CG40178*** | FBgn0058178 | FBtr0113817,FBtr0113818 | 373 | 841 | 1,42394E-65 | **-1,17293** |
| ***ens*** | FBgn0035500 | FBtr0073269,FBtr0073266,FBtr0073268,FBtr0073267,FBtr0073265 | 1535 | 3462 | 1,23333E-267 | **-1,17337** |
| ***snmRNA:838*** | FBgn0065081 | FBtr0091797 | 409 | 925 | 2,95653E-72 | **-1,17735** |
| ***ovo*** | FBgn0003028 | FBtr0070740,FBtr0070738,FBtr0070739,FBtr0100408 | 1071 | 2425 | 1,30572E-188 | **-1,17903** |
| ***mp*** | FBgn0260660 | FBtr0301958 | 262 | 594 | 6,42873E-47 | **-1,18090** |
| ***Parp*** | FBgn0010247 | FBtr0113885 | 2081 | 4726 | 0 | **-1,18334** |
| ***alpha-Man-I*** | FBgn0259170 | FBtr0300512,FBtr0299632,FBtr0299633,FBtr0300513,FBtr0300514,FBtr0300515,FBtr0300516 | 420 | 955 | 3,50134E-75 | **-1,18511** |
| ***CG7971*** | FBgn0035253 | FBtr0072806 | 258 | 587 | 1,26070E-46 | **-1,18599** |
| ***Fbp2*** | FBgn0000640 | FBtr0079808 | 1368 | 3125 | 3,71057E-246 | **-1,19179** |
| ***Dcp2*** | FBgn0036534 | FBtr0304975 | 5414 | 12369 | 0 | **-1,19196** |
| ***CG40160*** | FBgn0058160 | FBtr0301802 | 101 | 231 | 5,46026E-19 | **-1,19354** |
| ***ovo*** | FBgn0003028 | FBtr0301914 | 1839 | 4223 | 0 | **-1,19935** |

| | | | | | | |
|---|---|---|---|---|---|---|
| ***Pten*** | FBgn0026379 | FBtr0089900,FBtr0089901,FBtr0089904, FBtr0089905,FBtr0300562,FBtr0301521 | 111 | 255 | 5,90781E-21 | **-1,19994** |
| ***CkIalpha*** | FBgn0015024 | FBtr0073681,FBtr0073682,FBtr0300380 | 4876 | 11209 | 0 | **-1,20089** |
| ***Atf6*** | FBgn0033010 | FBtr0086081 | 1218 | 2808 | 3,49403E-224 | **-1,20503** |
| ***CR42723*** | FBgn0261640 | FBtr0303010 | 229 | 528 | 9,01143E-43 | **-1,20519** |
| ***bun*** | FBgn0259176 | FBtr0299656 | 428 | 987 | 2,51707E-79 | **-1,20544** |
| | | FBtr0303296 | 3035 | 6999 | 0 | **-1,20545** |
| | | FBtr0301360,FBtr0301361 | 3035 | 6999 | 0 | **-1,20545** |
| ***rl*** | FBgn0003256 | FBtr0113699 | 111 | 256 | 3,92375E-21 | **-1,20558** |
| ***hth*** | FBgn0001235 | FBtr0082256,FBtr0082254,FBtr0082255, FBtr0082253,FBtr0301345,FBtr0301956 | 2368 | 5471 | 0 | **-1,20814** |
| ***eIF5*** | FBgn0030719 | FBtr0074147,FBtr0074146,FBtr0074144, FBtr0074148,FBtr0074145,FBtr0074150 | 157 | 363 | 9,02092E-30 | **-1,20921** |
| ***Rbp2*** | FBgn0262734 | FBtr0074279,FBtr0074280 | 8054 | 18674 | 0 | **-1,21325** |
| ***CR42722*** | FBgn0261639 | FBtr0303009 | 239 | 556 | 1,25553E-45 | **-1,21807** |
| ***vfl*** | FBgn0259789 | FBtr0307536 | 917 | 2134 | 8,26687E-173 | **-1,21857** |
| ***Ank*** | FBgn0011747 | FBtr0089173 | 131 | 305 | 2,05851E-25 | **-1,21924** |
| ***ci*** | FBgn0004859 | FBtr0089178 | 725 | 1692 | 1,28452E-137 | **-1,22268** |
| ***Dyrk3*** | FBgn0027101 | FBtr0100402,FBtr0100403,FBtr0100404, FBtr0100405 | 403 | 943 | 2,18515E-77 | **-1,22648** |
| ***syd*** | FBgn0024187 | FBtr0076769,FBtr0300412,FBtr0300413 | 134 | 314 | 2,37657E-26 | **-1,22853** |
| ***par-1*** | FBgn0260934 | FBtr0086452,FBtr0086459,FBtr0086453, FBtr0086454,FBtr0086455,FBtr0086457, FBtr0086458,FBtr0100390,FBtr0086460, FBtr0301505,FBtr0301506 | 1462 | 3426 | 1,06454E-279 | **-1,22858** |
| ***Adar*** | FBgn0026086 | FBtr0305499 | 1148 | 2694 | 1,52968E-220 | **-1,23063** |
| ***CG17159*** | FBgn0039945 | FBtr0113717,FBtr0113721 | 702 | 1649 | 1,81151E-135 | **-1,23205** |
| ***sm*** | FBgn0003435 | FBtr0086492,FBtr0100232,FBtr0301609, FBtr0304654,FBtr0304655,FBtr0304656 | 125 | 294 | 8,06989E-25 | **-1,23389** |
| ***CG7115*** | FBgn0027515 | FBtr0079523 | 130 | 306 | 6,93494E-26 | **-1,23502** |
| ***Tsp39D*** | FBgn0032943 | FBtr0273404 | 1442 | 3405 | 7,33644E-281 | **-1,23958** |
| ***Zyx*** | FBgn0011642 | FBtr0089210,FBtr0089211,FBtr0089212, FBtr0089213,FBtr0089214,FBtr0089215, FBtr0089216 | 1261 | 2978 | 7,19690E-246 | **-1,23978** |
| ***gish*** | FBgn0250823 | FBtr0301304,FBtr0305071,FBtr0305072 | 1118 | 2644 | 7,94552E-219 | **-1,24180** |
| ***Asator*** | FBgn0039908 | FBtr0300342,FBtr0300344 | 385 | 912 | 3,84307E-76 | **-1,24418** |
| ***fwe*** | FBgn0261722 | FBtr0075543 | 105 | 250 | 1,28715E-21 | **-1,25154** |
| ***sdt*** | FBgn0261873 | FBtr0089978,FBtr0100376,FBtr0111034,F Btr0089975,FBtr0100375,FBtr0089974,F Btr0308218,FBtr0308219,FBtr0308220 | 514 | 1224 | 1,36825E-102 | **-1,25176** |
| ***CG5065*** | FBgn0034145 | FBtr0087108,FBtr0302206 | 154 | 367 | 2,24687E-31 | **-1,25285** |
| ***PP2A-B'*** | FBgn0042693 | FBtr0290319 | 321 | 765 | 1,95058E-64 | **-1,25289** |
| ***Nos*** | FBgn0011676 | FBtr0100484 | 472 | 1126 | 9,60926E-95 | **-1,25435** |
| ***CG2233*** | FBgn0029990 | FBtr0071123 | 472 | 1126 | 9,60926E-95 | **-1,25435** |
| ***ogre*** | FBgn0004646 | FBtr0071036 | 1819 | 4345 | 0 | **-1,25621** |
| ***cals*** | FBgn0039928 | FBtr0089207 | 129 | 310 | 6,39671E-27 | **-1,26490** |
| ***dally*** | FBgn0011577 | FBtr0305901 | 2835 | 6822 | 0 | **-1,26685** |
| ***RecQ5*** | FBgn0027375 | FBtr0075714,FBtr0075715,FBtr0100362 | 278 | 669 | 2,80617E-57 | **-1,26692** |
| ***sky*** | FBgn0032901 | FBtr0081442,FBtr0081443,FBtr0081444, FBtr0081446,FBtr0081447,FBtr0081440, FBtr0081441,FBtr0301961 | 657 | 1586 | 3,66855E-135 | **-1,27143** |
| ***norpA*** | FBgn0262738 | FBtr0100670,FBtr0070651 | 895 | 2165 | 7,09472E-185 | **-1,27441** |
| ***Pdk1*** | FBgn0020386 | FBtr0072470,FBtr0072471,FBtr0072466, FBtr0072467 | 863 | 2088 | 2,76865E-178 | **-1,27469** |
| ***CG32350*** | FBgn0052350 | FBtr0070046 | 1234 | 2989 | 2,67323E-255 | **-1,27632** |
| ***myoglianin*** | FBgn0026199 | FBtr0089092,FBtr0089093,FBtr0089094, FBtr0089095 | 1046 | 2537 | 2,74952E-217 | **-1,27824** |
| ***TBPH*** | FBgn0025790 | FBtr0089624,FBtr0089626,FBtr0089627, FBtr0301643,FBtr0301644 | 146 | 356 | 2,16580E-31 | **-1,28591** |
| ***Mkk4*** | FBgn0024326 | FBtr0300443 | 706 | 1722 | 7,66679E-149 | **-1,28635** |
| ***par-1*** | FBgn0260934 | FBtr0100391,FBtr0100392,FBtr0301504 | 601 | 1468 | 3,71299E-127 | **-1,28842** |
| ***rl*** | FBgn0003256 | FBtr0113700 | 97 | 237 | 2,94223E-21 | **-1,28883** |
| ***CdGAPr*** | FBgn0032821 | FBtr0300966 | 221 | 540 | 2,40085E-47 | **-1,28891** |
| ***unk*** | FBgn0004395 | FBtr0305573 | 1206 | 2953 | 7,19489E-256 | **-1,29195** |
| ***RpL38*** | FBgn0040007 | FBtr0111120 | 9077 | 22236 | 0 | **-1,29261** |
| ***Dyrk3*** | FBgn0027101 | FBtr0100402,FBtr0100406 | 173 | 424 | 1,83968E-37 | **-1,29329** |
| ***CR41604*** | FBgn0085814 | FBtr0114270 | 408 | 1000 | 2,58936E-87 | **-1,29336** |
| ***CdGAPr*** | FBgn0032821 | FBtr0300967 | 220 | 540 | 1,81284E-47 | **-1,29546** |
| ***bowl*** | FBgn0004893 | FBtr0077490,FBtr0077491,FBtr0077492, FBtr0307026,FBtr0307027,FBtr0307028, FBtr0307029 | 483 | 1186 | 1,16326E-103 | **-1,29601** |
| ***heph*** | FBgn0011224 | FBtr0085885 | 124 | 305 | 2,52222E-27 | **-1,29847** |
| ***CG42724*** | FBgn0261641 | FBtr0303012,FBtr0303018 | 119 | 294 | 1,90589E-26 | **-1,30485** |
| ***CG42724*** | FBgn0261641 | FBtr0303013,FBtr0303016 | 119 | 294 | 1,90589E-26 | **-1,30485** |
| ***CG17528*** | FBgn0261387 | FBtr0111276,FBtr0111275,FBtr0111277 | 197 | 487 | 2,37831E-43 | **-1,30573** |
| ***Mkk4*** | FBgn0024326 | FBtr0081892 | 735 | 1820 | 2,55020E-160 | **-1,30812** |
| ***gro*** | FBgn0001139 | FBtr0305047 | 1834 | 4562 | 0 | **-1,31467** |
| ***CG4502*** | FBgn0031896 | FBtr0079417 | 327 | 816 | 4,82499E-73 | **-1,31928** |
| ***pncr013:4*** | FBgn0262731 | FBtr0091952 | 409 | 1021 | 3,70007E-91 | **-1,31981** |
| ***CG4502*** | FBgn0031896 | FBtr0079416 | 324 | 809 | 1,79217E-72 | **-1,32015** |
| ***stai*** | FBgn0051641 | FBtr0304908 | 2607 | 6517 | 0 | **-1,32182** |
| ***CR41604*** | FBgn0085814 | FBtr0114271,FBtr0114272 | 412 | 1034 | 4,75262E-93 | **-1,32752** |
| ***CR41604*** | FBgn0085814 | FBtr0114269 | 411 | 1032 | 8,44174E-93 | **-1,32823** |
| ***stai*** | FBgn0051641 | FBtr0079196,FBtr0079197,FBtr0079198 | 2583 | 6490 | 0 | **-1,32917** |
| ***Hrb98DE*** | FBgn0001215 | FBtr0085300,FBtr0085303 | 83 | 209 | 2,07856E-19 | **-1,33232** |
| ***Mhcl*** | FBgn0026059 | FBtr0083231 | 158 | 399 | 1,10417E-36 | **-1,33646** |
| ***plexA*** | FBgn0025741 | FBtr0089224,FBtr0100296 | 106 | 268 | 7,41937E-25 | **-1,33817** |
| ***Marf*** | FBgn0029870 | FBtr0070908,FBtr0070910 | 104 | 264 | 1,00277E-24 | **-1,34395** |
| ***clumsy*** | FBgn0026255 | FBtr0081476,FBtr0110895 | 137 | 349 | 1,62519E-32 | **-1,34905** |
| ***zip*** | FBgn0005634 | FBtr0072399,FBtr0100466,FBtr0100467, FBtr0302572,FBtr0302573,FBtr0302574, FBtr0302575,FBtr0306576 | 1064 | 2712 | 1,38663E-247 | **-1,34986** |
| ***mp*** | FBgn0260660 | FBtr0308095 | 104 | 266 | 4,11726E-25 | **-1,35484** |
| ***CG41454*** | FBgn0084017 | FBtr0111173 | 78 | 200 | 4,13435E-19 | **-1,35845** |
| ***CrebA*** | FBgn0004396 | FBtr0075557 | 631 | 1618 | 5,28727E-149 | **-1,35850** |
| ***myoglianin*** | FBgn0026199 | FBtr0089094 | 294 | 755 | 5,04510E-70 | **-1,36066** |
| ***Hr46*** | FBgn0000448 | FBtr0306346 | 358 | 922 | 1,09639E-85 | **-1,36481** |
| ***Atf6*** | FBgn0033010 | FBtr0086079,FBtr0086080 | 805 | 2078 | 8,45723E-193 | **-1,36813** |
| ***Dh44-R2*** | FBgn0033744 | FBtr0113075 | 143 | 370 | 5,37724E-35 | **-1,37151** |
| ***CG12054*** | FBgn0039831 | FBtr0305118 | 1490 | 3863 | 0 | **-1,37441** |
| ***CG11727*** | FBgn0262740 | FBtr0073547 | 733 | 1902 | 8,34279E-178 | **-1,37563** |
| ***CR41597*** | FBgn0085810 | FBtr0114264 | 198 | 514 | 1,42652E-48 | **-1,37627** |
| ***CG8116*** | FBgn0037614 | FBtr0300543 | 133 | 348 | 1,96894E-33 | **-1,38766** |

| | | | | | | |
|---|---|---|---|---|---|---|
| ***hth*** | FBgn0001235 | FBtr0100454 | 3987 | 10441 | 0 | **-1,38888** |
| ***pho*** | FBgn0002521 | FBtr0089204,FBtr0089205 | 1568 | 4113 | 0 | **-1,39127** |
| ***PMCA*** | FBgn0259214 | FBtr0304046,FBtr0304047,FBtr0304048, FBtr0304049,FBtr0300554,FBtr0300555, FBtr0300556,FBtr0300557 | 287 | 753 | 9,53534E-72 | **-1,39160** |
| ***vn*** | FBgn0003984 | FBtr0077082 | 276 | 725 | 3,85431E-69 | **-1,39331** |
| ***CaMKI*** | FBgn0016126 | FBtr0089065 | 476 | 1255 | 9,72904E-120 | **-1,39865** |
| ***CG11266*** | FBgn0031883 | FBtr0089638,FBtr0089639,FBtr0089635 | 195 | 517 | 3,75925E-50 | **-1,40669** |
| ***Ptp10D*** | FBgn0004370 | FBtr0073524,FBtr0273235 | 718 | 1904 | 1,31837E-182 | **-1,40698** |
| ***mRpS5*** | FBgn0044510 | FBtr0111147,FBtr0111146 | 420 | 1117 | 7,13268E-108 | **-1,41117** |
| ***H*** | FBgn0001169 | FBtr0083915,FBtr0083916 | 217 | 580 | 1,01908E-56 | **-1,41836** |
| ***Nhe3*** | FBgn0028703 | FBtr0273247,FBtr0273248,FBtr0273250 | 162 | 433 | 1,48752E-42 | **-1,41837** |
| ***UbcD2*** | FBgn0015320 | FBtr0080115,FBtr0080116 | 709 | 1897 | 7,24710E-184 | **-1,41986** |
| ***CG11266*** | FBgn0031883 | FBtr0089637 | 195 | 522 | 3,88705E-51 | **-1,42058** |
| ***CG30343*** | FBgn0050343 | FBtr0088594 | 290 | 778 | 4,86016E-76 | **-1,42372** |
| ***Adar*** | FBgn0026086 | FBtr0070299,FBtr0070300,FBtr0100557, FBtr0307895,FBtr0305498 | 151 | 411 | 3,84228E-41 | **-1,44459** |
| ***pUf68*** | FBgn0028577 | FBtr0072708,FBtr0072710,FBtr0300418 | 95 | 260 | 1,49000E-26 | **-1,45251** |
| ***bw*** | FBgn0000241 | FBtr0072117 | 126 | 345 | 5,22632E-35 | **-1,45317** |
| ***A2bp1*** | FBgn0052062 | FBtr0305093,FBtr0305096 | 1273 | 3499 | 0 | **-1,45871** |
| ***CG32709*** | FBgn0052709 | FBtr0305288 | 68 | 187 | 1,71352E-19 | **-1,45943** |
| ***Cam*** | FBgn0000253 | FBtr0088001,FBtr0088002 | 8477 | 23442 | 0 | **-1,46747** |
| ***sky*** | FBgn0032901 | FBtr0081445 | 276 | 764 | 1,75575E-77 | **-1,46890** |
| ***crq*** | FBgn0015924 | FBtr0078087 | 603 | 1670 | 1,00872E-168 | **-1,46962** |
| ***Galpha49B*** | FBgn0004435 | FBtr0087829,FBtr0087830 | 334 | 930 | 8,17659E-95 | **-1,47738** |
| ***CG41520*** | FBgn0087011 | FBtr0114111,FBtr0114112,FBtr0302581,F Btr0302582 | 140 | 390 | 3,62801E-40 | **-1,47805** |
| ***tal-1A*** | FBgn0259730 | FBtr0299997 | 364 | 1015 | 1,24397E-103 | **-1,47947** |
| ***tal-2A*** | FBgn0259731 | FBtr0299998 | 364 | 1015 | 1,24397E-103 | **-1,47947** |
| ***tal-3A*** | FBgn0259732 | FBtr0299999 | 364 | 1015 | 1,24397E-103 | **-1,47947** |
| ***tal-AA*** | FBgn0259733 | FBtr0299996 | 364 | 1015 | 1,24397E-103 | **-1,47947** |
| ***CadN*** | FBgn0015609 | FBtr0081016,FBtr0081015,FBtr0100313, FBtr0100315 | 152 | 424 | 8,51733E-44 | **-1,47999** |
| ***CG32856*** | FBgn0052856 | FBtr0083239,FBtr0302551 | 86 | 240 | 4,48799E-25 | **-1,48063** |
| ***pho*** | FBgn0002521 | FBtr0089204 | 121 | 338 | 3,94237E-35 | **-1,48202** |
| ***CkIalpha*** | FBgn0015024 | FBtr0073680 | 5117 | 14301 | 0 | **-1,48275** |
| ***eIF-4B*** | FBgn0020660 | FBtr0113680 | 78 | 218 | 7,65373E-23 | **-1,48278** |
| ***AP-1gamma*** | FBgn0030089 | FBtr0071295,FBtr0071297,FBtr0071298, FBtr0112965 | 84 | 235 | 1,47814E-24 | **-1,48420** |
| ***heph*** | FBgn0011224 | FBtr0300268,FBtr0300269 | 173 | 484 | 5,90781E-50 | **-1,48424** |
| ***pncr013:4*** | FBgn0262731 | FBtr0303019,FBtr0303020,FBtr0303021 | 168 | 471 | 1,11139E-48 | **-1,48727** |
| ***Df31*** | FBgn0022893 | FBtr0085919,FBtr0085920 | 29123 | 81808 | 0 | **-1,49008** |
| ***Df31*** | FBgn0022893 | FBtr0100293 | 29264 | 82324 | 0 | **-1,49219** |
| ***CG11727*** | FBgn0262740 | FBtr0300733 | 554 | 1561 | 9,18083E-161 | **-1,49451** |
| ***how*** | FBgn0017397 | FBtr0084177 | 1467 | 4160 | 0 | **-1,50371** |
| ***CaMKI*** | FBgn0016126 | FBtr0089066 | 67 | 190 | 1,87120E-20 | **-1,50377** |
| ***Zyx*** | FBgn0011642 | FBtr0089210,FBtr0089213 | 160 | 457 | 2,71772E-48 | **-1,51412** |
| ***qkr58E-3*** | FBgn0022984 | FBtr0290254 | 859 | 2461 | 6,85726E-258 | **-1,51851** |
| ***Dyrk3*** | FBgn0027101 | FBtr0100406 | 461 | 1324 | 1,91524E-139 | **-1,52206** |
| ***Akap200*** | FBgn0027932 | FBtr0079665 | 112 | 324 | 6,47659E-35 | **-1,53250** |
| ***how*** | FBgn0017397 | FBtr0301401 | 1394 | 4049 | 0 | **-1,53834** |
| ***Mitf*** | FBgn0263112 | FBtr0307326,FBtr0307327,FBtr0307329, FBtr0307330 | 172 | 501 | 5,31867E-54 | **-1,54240** |
| ***Ptp10D*** | FBgn0004370 | FBtr0073525,FBtr0073522 | 323 | 942 | 4,79061E-101 | **-1,54419** |
| ***gus*** | FBgn0026238 | FBtr0089755,FBtr0089752,FBtr0089754, FBtr0089757,FBtr0089753,FBtr0089756 | 457 | 1339 | 4,93461E-144 | **-1,55089** |
| ***lin19*** | FBgn0015509 | FBtr0088846 | 108 | 317 | 9,87245E-35 | **-1,55345** |
| ***slow*** | FBgn0035539 | FBtr0073291 | 84 | 247 | 3,17946E-27 | **-1,55605** |
| ***Zyx*** | FBgn0011642 | FBtr0089211,FBtr0089216 | 161 | 474 | 1,25213E-51 | **-1,55783** |
| ***gish*** | FBgn0250823 | FBtr0083262,FBtr0083261,FBtr0083265, FBtr0083264,FBtr0100332,FBtr0100333 | 783 | 2311 | 4,43206E-250 | **-1,56143** |
| ***Zyx*** | FBgn0011642 | FBtr0089212,FBtr0089215 | 161 | 476 | 3,08894E-52 | **-1,56390** |
| ***Mbs*** | FBgn0005536 | FBtr0112850,FBtr0112851,FBtr0112852,F Btr0301472,FBtr0301473,FBtr0301575,F Btr0308213,FBtr0308214,FBtr0308215,F Btr0308216 | 453 | 1340 | 1,62726E-145 | **-1,56465** |
| ***Antp*** | FBgn0260642 | FBtr0081654,FBtr0081655 | 340 | 1009 | 4,03823E-110 | **-1,56932** |
| ***inx2*** | FBgn0027108 | FBtr0071005,FBtr0071006 | 13280 | 39521 | 0 | **-1,57336** |
| ***qkr58E-3*** | FBgn0022984 | FBtr0307214 | 909 | 2715 | 2,23244E-297 | **-1,57860** |
| ***zip*** | FBgn0005634 | FBtr0072398 | 1395 | 4169 | 0 | **-1,57944** |
| ***Cam*** | FBgn0000253 | FBtr0304963,FBtr0304964 | 8960 | 26840 | 0 | **-1,58281** |
| ***Hr46*** | FBgn0000448 | FBtr0306345 | 706 | 2122 | 4,88718E-234 | **-1,58768** |
| ***shi*** | FBgn0003392 | FBtr0111036,FBtr0111037,FBtr0074118,F Btr0074119,FBtr0074121,FBtr0074122,F Btr0301597 | 432 | 1300 | 8,43643E-144 | **-1,58941** |
| ***CR43241*** | FBgn0262886 | FBtr0306297 | 91 | 274 | 7,23096E-31 | **-1,59024** |
| ***CG40228*** | FBgn0063670 | FBtr0113841 | 600 | 1813 | 3,02493E-201 | **-1,59534** |
| ***CG8500*** | FBgn0037754 | FBtr0082139 | 143 | 433 | 9,14005E-49 | **-1,59835** |
| ***Akap200*** | FBgn0027932 | FBtr0079665,FBtr0079667 | 5576 | 16929 | 0 | **-1,60219** |
| ***Akap200*** | FBgn0027932 | FBtr0079664,FBtr0079666 | 5576 | 16932 | 0 | **-1,60245** |
| ***Zyx*** | FBgn0011642 | FBtr0089210,FBtr0089213,FBtr0089215 | 199 | 605 | 4,20877E-68 | **-1,60417** |
| ***Dyb*** | FBgn0033739 | FBtr0087930,FBtr0087929,FBtr0100298, FBtr0305077 | 199 | 608 | 9,41844E-69 | **-1,61130** |
| ***CG40196*** | FBgn0058196 | FBtr0113831 | 176 | 538 | 7,09221E-61 | **-1,61203** |
| ***unc-13*** | FBgn0025726 | FBtr0089245 | 210 | 644 | 5,21925E-73 | **-1,61667** |
| ***lin19*** | FBgn0015509 | FBtr0088845 | 99 | 304 | 5,74404E-35 | **-1,61857** |
| ***CG42258*** | FBgn0259143 | FBtr0299558,FBtr0299559 | 372 | 1149 | 1,20800E-130 | **-1,62700** |
| ***CG15923*** | FBgn0038814 | FBtr0113254 | 676 | 2089 | 4,11093E-237 | **-1,62772** |
| ***pAbp*** | FBgn0261619 | FBtr0086740 | 525 | 1627 | 2,16402E-185 | **-1,63182** |
| ***Zyx*** | FBgn0011642 | FBtr0089211,FBtr0089212,FBtr0089214, FBtr0089216 | 225 | 702 | 8,38688E-81 | **-1,64155** |
| ***unc-13*** | FBgn0025726 | FBtr0089246,FBtr0089247 | 210 | 658 | 3,30170E-76 | **-1,64770** |
| ***CG17528*** | FBgn0261387 | FBtr0111274 | 72 | 227 | 5,86429E-27 | **-1,65662** |
| ***CG17698*** | FBgn0040056 | FBtr0111168,FBtr0111167 | 298 | 941 | 1,49195E-109 | **-1,65888** |
| ***mt:Cyt-b*** | FBgn0013678 | FBtr0100884 | 10582 | 33560 | 0 | **-1,66513** |
| ***CG11266*** | FBgn0031883 | FBtr0089639,FBtr0089635 | 62 | 197 | 1,30601E-23 | **-1,66786** |
| ***spz4*** | FBgn0032362 | FBtr0305261 | 55 | 175 | 3,34641E-21 | **-1,66985** |
| ***CkIIbeta*** | FBgn0000259 | FBtr0073562 | 58 | 185 | 2,16939E-22 | **-1,67340** |
| ***CG17715*** | FBgn0041004 | FBtr0111247 | 465 | 1491 | 1,33298E-175 | **-1,68098** |
| ***CG17683*** | FBgn0262115 | FBtr0111301 | 116 | 374 | 8,00901E-45 | **-1,68891** |
| ***CrebA*** | FBgn0004396 | FBtr0075558 | 1076 | 3520 | 0 | **-1,70990** |
| ***qkr54B*** | FBgn0022987 | FBtr0306248 | 550 | 1804 | 3,30019E-217 | **-1,71370** |

| | | | | | | |
|---|---|---|---|---|---|---|
| Hr46 | FBgn0000448 | FBtr0088366,FBtr0088368,FBtr0112799, FBtr0302438 | 563 | 1848 | 1,26810E-222 | -1,71476 |
| pncr013:4 | FBgn0262731 | FBtr0303020 | 52 | 171 | 2,41338E-21 | -1,71741 |
| CG17715 | FBgn0041004 | FBtr0111249 | 640 | 2109 | 7,61203E-255 | -1,72042 |
| clumsy | FBgn0026255 | FBtr0081476 | 44 | 145 | 2,87485E-18 | -1,72048 |
| sgg | FBgn0003371 | FBtr0070475,FBtr0070476,FBtr0301966 | 1077 | 3564 | 0 | -1,72648 |
| | | FBtr0082858 | 11411 | 37797 | 0 | -1,72785 |
| sgg | FBgn0003371 | FBtr0070471,FBtr0070472,FBtr0070473, FBtr0070466,FBtr0070468,FBtr0070469, FBtr0070470 | 1085 | 3607 | 0 | -1,73310 |
| mt:CoIII | FBgn0013676 | FBtr0100868 | 24108 | 80212 | 0 | -1,73431 |
| Pabp2 | FBgn0005648 | FBtr0088786 | 136 | 462 | 6,85597E-58 | -1,76429 |
| CG12567 | FBgn0039958 | FBtr0113704,FBtr0113705,FBtr0300702,FBtr0300703,FBtr0300704 | 386 | 1316 | 2,79252E-164 | -1,76949 |
| CG9894 | FBgn0031453 | FBtr0077713,FBtr0077714,FBtr0307080 | 1577 | 5391 | 0 | -1,77337 |
| myoglianin | FBgn0026199 | FBtr0089095 | 77 | 267 | 2,47473E-34 | -1,79391 |
| Pdp1 | FBgn0016694 | FBtr0076776,FBtr0076777,FBtr0300499, FBtr0306535 | 76 | 265 | 2,51395E-34 | -1,80192 |
| Pur-alpha | FBgn0022361 | FBtr0089996,FBtr0089995,FBtr0089994, FBtr0089993 | 389 | 1363 | 1,89792E-174 | -1,80894 |
| Cyp6g1 | FBgn0025454 | FBtr0087992 | 69 | 243 | 1,02594E-31 | -1,81629 |
| pallidin | FBgn0036192 | FBtr0300097,FBtr0300725,FBtr0300726 | 80 | 282 | 7,27341E-37 | -1,81762 |
| CaMKII | FBgn0004624 | FBtr0089218,FBtr0089219,FBtr0089217 | 438 | 1545 | 9,24175E-199 | -1,81860 |
| CaMKII | FBgn0004624 | FBtr0100146,FBtr0100147,FBtr0100148, FBtr0300378 | 438 | 1546 | 5,50608E-199 | -1,81954 |
| CG17683 | FBgn0262115 | FBtr0111298,FBtr0111299 | 68 | 244 | 2,17454E-32 | -1,84327 |
| Mnt | FBgn0023215 | FBtr0307278,FBtr0301823 | 588 | 2113 | 4,46420E-276 | -1,84540 |
| Nrg | FBgn0002968 | FBtr0071207,FBtr0071209,FBtr0301762, FBtr0301764,FBtr0305914 | 955 | 3445 | 0 | -1,85093 |
| eIF4G | FBgn0023213 | FBtr0089243,FBtr0112904 | 120 | 435 | 7,55305E-58 | -1,85798 |
| gish | FBgn0250823 | FBtr0083263 | 845 | 3086 | 0 | -1,86871 |
| eIF4G | FBgn0023213 | FBtr0289951 | 120 | 439 | 8,85716E-59 | -1,87119 |
| CaMKI | FBgn0016126 | FBtr0089067 | 131 | 484 | 2,69554E-65 | -1,88544 |
| CaMKI | FBgn0016126 | FBtr0089069 | 53 | 198 | 1,99656E-27 | -1,90144 |
| tlk | FBgn0086899 | FBtr0299580,FBtr0299582,FBtr0301659 | 44 | 165 | 5,18156E-23 | -1,90689 |
| piwi | FBgn0004872 | FBtr0080166 | 38 | 143 | 5,90831E-20 | -1,91194 |
| UBL3 | FBgn0026076 | FBtr0074175 | 54 | 204 | 2,24984E-28 | -1,91754 |
| eIF-4B | FBgn0020660 | FBtr0113679 | 123 | 469 | 8,35145E-65 | -1,93093 |
| mt:CoI | FBgn0013674 | FBtr0100861 | 38379 | 146749 | 0 | -1,93496 |
| Antp | FBgn0260642 | FBtr0081647,FBtr0081648,FBtr0081649, FBtr0081650,FBtr0081651,FBtr0081652, FBtr0081653,FBtr0081656 | 485 | 1855 | 2,26490E-255 | -1,93536 |
| | | FBtr0076660 | 582 | 2241 | 4,0709E-310 | -1,94505 |
| Fas3 | FBgn0000636 | FBtr0081051,FBtr0081052 | 1972 | 7762 | 0 | -1,97677 |
| kn | FBgn0001319 | FBtr0087465,FBtr0112810,FBtr0301400 | 146 | 575 | 2,00160E-81 | -1,97759 |
| Eph | FBgn0025936 | FBtr0089082,FBtr0089083,FBtr0089084 | 310 | 1229 | 2,62596E-174 | -1,98714 |
| Ank | FBgn0011747 | FBtr0089174 | 117 | 465 | 1,75090E-66 | -1,99072 |
| grk | FBgn0001137 | FBtr0079708 | 85 | 338 | 1,74675E-48 | -1,99149 |
| plexA | FBgn0025741 | FBtr0089225,FBtr0089226,FBtr0089223 | 144 | 573 | 6,82466E-82 | -1,99247 |
| CG2316 | FBgn0039890 | FBtr0089148 | 33 | 132 | 1,62356E-19 | -2 |
| CG32016 | FBgn0052016 | FBtr0089229 | 80 | 321 | 2,16540E-46 | -2,00450 |
| CG8419 | FBgn0031999 | FBtr0079589 | 100 | 404 | 1,44467E-58 | -2,01436 |
| lola | FBgn0005630 | FBtr0089365,FBtr0089347,FBtr0089361, FBtr0089360,FBtr0089366,FBtr0089355, FBtr0089354,FBtr0089346,FBtr0089345, FBtr0089364 | 128 | 518 | 6,20061E-75 | -2,01681 |
| Galpha49B | FBgn0004435 | FBtr0304955 | 828 | 3452 | 0 | -2,05973 |
| CG17715 | FBgn0041004 | FBtr0111244,FBtr0111246,FBtr0111245,FBtr0111248,FBtr0306550,FBtr0306551 | 142 | 593 | 1,14625E-87 | -2,06214 |
| Eph | FBgn0025936 | FBtr0089086 | 257 | 1076 | 6,00570E-159 | -2,06584 |
| Ank | FBgn0011747 | FBtr0089172 | 29 | 122 | 8,17736E-19 | -2,07276 |
| Mnt | FBgn0023215 | FBtr0307277,FBtr0301822 | 646 | 2748 | 0 | -2,08878 |
| Rbp1-like | FBgn0030479 | FBtr0073790 | 233 | 997 | 8,48509E-150 | -2,09726 |
| CG9894 | FBgn0031453 | FBtr0307080 | 3611 | 15511 | 0 | -2,10282 |
| swi2 | FBgn0034262 | FBtr0086889 | 66 | 284 | 3,01724E-43 | -2,10535 |
| Ank | FBgn0011747 | FBtr0089173,FBtr0089171,FBtr0089172, FBtr0300497,FBtr0300498 | 99 | 429 | 2,47363E-65 | -2,11548 |
| CG9894 | FBgn0031453 | FBtr0077713,FBtr0077714 | 2522 | 11197 | 0 | -2,15047 |
| H | FBgn0001169 | FBtr0083914,FBtr0083917 | 426 | 1911 | 1,96911E-296 | -2,16540 |
| CG17471 | FBgn0039924 | FBtr0100543 | 53 | 241 | 3,07592E-38 | -2,18497 |
| CG17471 | FBgn0039924 | FBtr0100544 | 52 | 238 | 5,01616E-38 | -2,19438 |
| Hr39 | FBgn0261239 | FBtr0081480 | 209 | 966 | 2,89772E-153 | -2,20852 |
| pncr013:4 | FBgn0262731 | FBtr0303019 | 75 | 351 | 1,52964E-56 | -2,22651 |
| CG32016 | FBgn0052016 | FBtr0089233 | 90 | 424 | 2,16374E-68 | -2,23607 |
| mt:CoII | FBgn0013675 | FBtr0100863 | 6779 | 32317 | 0 | -2,25315 |
| CG10006 | FBgn0036461 | FBtr0113171 | 26 | 124 | 1,12738E-20 | -2,25376 |
| CG10417 | FBgn0033021 | FBtr0086092 | 71 | 341 | 6,05536E-56 | -2,26388 |
| pncr013:4 | FBgn0262731 | FBtr0091952,FBtr0303019,FBtr0303020, FBtr0303021 | 39 | 190 | 1,40467E-31 | -2,28445 |
| CG32016 | FBgn0052016 | FBtr0089232 | 63 | 307 | 7,74506E-51 | -2,28481 |
| CR43241 | FBgn0262886 | FBtr0306296 | 30 | 149 | 2,98909E-25 | -2,31228 |
| Pdp1 | FBgn0016694 | FBtr0076775,FBtr0076776,FBtr0076777, FBtr0076780,FBtr0076782,FBtr0300499, FBtr0300500 | 120 | 608 | 1,55930E-102 | -2,34104 |
| Galpha49B | FBgn0004435 | FBtr0087829,FBtr0087830,FBtr0087831, FBtr0087833,FBtr0087834,FBtr0304954 | 481 | 2445 | 0 | -2,34573 |
| Pdp1 | FBgn0016694 | FBtr0306535 | 122 | 623 | 1,68750E-105 | -2,35235 |
| myoglianin | FBgn0026199 | FBtr0089092,FBtr0089093 | 70 | 373 | 5,15266E-65 | -2,41375 |
| Hr39 | FBgn0261239 | FBtr0081479,FBtr0081481 | 593 | 3193 | 0 | -2,42881 |
| CG2225 | FBgn0032957 | FBtr0304885 | 87 | 476 | 5,94596E-84 | -2,45187 |
| CG2316 | FBgn0039890 | FBtr0089147 | 25 | 140 | 1,36617E-25 | -2,48543 |
| Pabp2 | FBgn0005648 | FBtr0088785 | 918 | 5443 | 0 | -2,56784 |
| how | FBgn0017397 | FBtr0100514 | 21 | 128 | 1,99577E-24 | -2,60768 |
| pAbp | FBgn0261619 | FBtr0086743,FBtr0086739 | 201 | 1240 | 5,79967E-233 | -2,62507 |
| pAbp | FBgn0261619 | FBtr0086738 | 201 | 1240 | 5,79967E-233 | -2,62507 |
| ventrally-expressed-protein-D | FBgn0053200 | FBtr0307213 | 27 | 167 | 5,16887E-32 | -2,62882 |
| CG2316 | FBgn0039890 | FBtr0089146 | 17 | 108 | 3,98726E-21 | -2,66742 |
| CG40196 | FBgn0058196 | FBtr0113829,FBtr0113830,FBtr0301121 | 40 | 258 | 6,03165E-50 | -2,68930 |
| UBL3 | FBgn0026076 | FBtr0074174,FBtr0300787 | 82 | 533 | 5,23642E-103 | -2,70044 |
| CG10417 | FBgn0033021 | FBtr0086091 | 74 | 485 | 2,98528E-94 | -2,71239 |
| CG7367 | FBgn0031976 | FBtr0306002 | 49 | 323 | 3,43181E-63 | -2,72068 |
| CG7367 | FBgn0031976 | FBtr0301128 | 69 | 460 | 5,56643E-90 | -2,73697 |
| CG3999 | FBgn0037801 | FBtr0082225 | 20 | 135 | 5,58658E-27 | -2,75489 |

| | | | | | | |
|---|---|---|---|---|---|---|
| ***CR30055*** | FBgn0050055 | FBtr0303919 | 13 | 90 | 2,25888E-18 | **-2,79141** |
| ***E2f*** | FBgn0011766 | FBtr0084118 | 82 | 569 | 1,82339E-113 | **-2,79473** |
| ***spo*** | FBgn0003486 | FBtr0077130 | 12 | 91 | 2,20890E-19 | **-2,92283** |
| ***mt:tRNA:Y*** | FBgn0013710 | FBtr0100860 | 12 | 96 | 9,13081E-21 | **-3** |
| ***CG15406*** | FBgn0031517 | FBtr0077580 | 17 | 149 | 3,68354E-33 | **-3,13171** |
| ***pncr013:4*** | FBgn0262731 | FBtr0303020,FBtr0303021 | 65 | 623 | 1,52988E-141 | **-3,26072** |
| ***TpnC25D*** | FBgn0031692 | FBtr0079063 | 18 | 178 | 2,61405E-41 | **-3,30581** |
| ***Act5C*** | FBgn0000042 | FBtr0070822,FBtr0100662,FBtr0100663 | 709 | 7183 | 0 | **-3,34073** |
| ***CG8979*** | FBgn0033669 | FBtr0088052 | 31 | 316 | 2,36749E-73 | **-3,34958** |
| ***CG32016*** | FBgn0052016 | FBtr0089234 | 21 | 217 | 7,63743E-51 | **-3,36923** |
| ***mt:tRNA:L:UUR*** | FBgn0013699 | FBtr0100862 | 9 | 95 | 1,35926E-22 | **-3,39993** |
| ***mt:ND4*** | FBgn0262952 | FBtr0100879 | 1109 | 11843 | 0 | **-3,41670** |
| ***CG32016*** | FBgn0052016 | FBtr0089230,FBtr0089231 | 12 | 133 | 3,91783E-32 | **-3,47032** |
| ***CG12990*** | FBgn0030859 | FBtr0074479 | 18 | 226 | 3,04199E-56 | **-3,65025** |
| ***mt:ND5*** | FBgn0013684 | FBtr0100877 | 1906 | 26146 | 0 | **-3,77797** |
| ***mt:ND3*** | FBgn0013681 | FBtr0100870 | 57 | 871 | 1,88953E-226 | **-3,93364** |
| ***CG9259*** | FBgn0032913 | FBtr0081464 | 13 | 232 | 8,41808E-63 | **-4,15754** |
| ***mt:tRNA:C*** | FBgn0013690 | FBtr0100859 | 8 | 144 | 3,09840E-39 | **-4,16993** |
| ***Zyx*** | FBgn0011642 | FBtr0089214 | 14 | 255 | 2,89275E-69 | **-4,18700** |
| ***Zyx*** | FBgn0011642 | FBtr0089213,FBtr0089215,FBtr0089216 | 14 | 257 | 6,47050E-70 | **-4,19827** |
| ***Zyx*** | FBgn0011642 | FBtr0089210,FBtr0089211,FBtr0089212 | 14 | 257 | 6,47050E-70 | **-4,19827** |
| ***CG10505*** | FBgn0034612 | FBtr0071619 | 7 | 146 | 6,57442E-41 | **-4,38247** |
| ***mt:ATPase6*** | FBgn0013672 | FBtr0100867 | 1667 | 36678 | 0 | **-4,45959** |
| ***mt:tRNA:W*** | FBgn0013709 | FBtr0100858 | 5 | 112 | 6,96342E-32 | **-4,48543** |
| ***mt:tRNA:G*** | FBgn0013694 | FBtr0100869 | 5 | 115 | 7,21270E-33 | **-4,52356** |
| ***CG15155*** | FBgn0032669 | FBtr0081048 | 3 | 85 | 3,48753E-25 | **-4,82443** |
| ***Fst*** | FBgn0037724 | FBtr0082101 | 11 | 374 | 4,24785E-112 | **-5,08746** |
| ***mt:ND1*** | FBgn0013679 | FBtr0100886 | 268 | 9585 | 0 | **-5,16047** |
| ***CG3264*** | FBgn0034712 | FBtr0071781 | 10 | 399 | 7,96223E-122 | **-5,31832** |
| ***CG7882*** | FBgn0033047 | FBtr0085966 | 2 | 81 | 4,83172E-25 | **-5,33985** |
| ***mt:lrRNA*** | FBgn0013686 | FBtr0100888 | 21890 | 975604 | 0 | **-5,47795** |
| ***Mur18B*** | FBgn0030999 | FBtr0074672 | 4 | 182 | 1,75026E-56 | **-5,50779** |
| ***CG40351*** | FBgn0040022 | FBtr0302243 | 2 | 93 | 4,43424E-29 | **-5,53916** |
| ***mt:ATPase8*** | FBgn0013673 | FBtr0100866 | 290 | 16433 | 0 | **-5,82440** |
| ***CG11892*** | FBgn0039313 | FBtr0089644 | 4 | 235 | 1,72731E-74 | **-5,87652** |
| ***CG11892*** | FBgn0039313 | FBtr0089645 | 4 | 235 | 1,72731E-74 | **-5,87652** |
| ***mt:srRNA*** | FBgn0013688 | FBtr0100890 | 6 | 357 | 3,71141E-113 | **-5,89482** |
| ***mt:ND2*** | FBgn0013680 | FBtr0100857 | 74 | 5273 | 0 | **-6,15495** |
| ***CG40351*** | FBgn0040022 | FBtr0113870,FBtr0113871 | 2 | 147 | 1,97588E-47 | **-6,19967** |
| ***CG40351*** | FBgn0040022 | FBtr0302248 | 1 | 75 | 2,36728E-24 | **-6,22882** |
| ***CG40351*** | FBgn0040022 | FBtr0113869,FBtr0302244 | 1 | 75 | 2,36728E-24 | **-6,22882** |
| ***mt:tRNA:L:CUN*** | FBgn0013698 | FBtr0100887 | 14 | 1386 | 0 | **-6,62936** |
| ***mt:ND6*** | FBgn0013685 | FBtr0100883 | 33 | 3640 | 0 | **-6,78533** |
| ***CG3292*** | FBgn0034710 | FBtr0071783 | 4 | 462 | 1,82753E-152 | **-6,85175** |
| ***mt:ND4L*** | FBgn0013683 | FBtr0100880 | 19 | 2698 | 0 | **-7,14975** |
| ***Muc11A*** | FBgn0052656 | FBtr0089803 | 2 | 378 | 4,55348E-127 | **-7,56224** |
| ***CG42235*** | FBgn0250757 | FBtr0290139 | 0 | 104 | 5,31274E-36 | **-Infinity** |
| ***CG10513*** | FBgn0039311 | FBtr0114505 | 0 | 78 | 5,38516E-27 | **-Infinity** |
| ***CG16727*** | FBgn0038719 | FBtr0083780 | 0 | 386 | 3,41807E-134 | **-Infinity** |
| ***CG17752*** | FBgn0038718 | FBtr0083779 | 0 | 157 | 2,05423E-54 | **-Infinity** |
| ***CG14292*** | FBgn0038658 | FBtr0083725 | 0 | 55 | 4,66333E-19 | **-Infinity** |
| ***CG42235*** | FBgn0250757 | FBtr0290140 | 0 | 92 | 7,66522E-32 | **-Infinity** |
| ***CG42235*** | FBgn0250757 | FBtr0290137,FBtr0290138,FBtr0290139, FBtr0290140,FBtr0290141 | 0 | 121 | 6,66973E-42 | **-Infinity** |
| ***mt:tRNA:P*** | FBgn0013702 | FBtr0100882 | 0 | 62 | 1,79899E-21 | **-Infinity** |
| ***CG10514*** | FBgn0039312 | FBtr0084864 | 0 | 132 | 9,98338E-46 | **-Infinity** |
