# Supplementary material for: New Partners in Regulation of Gene Expression: The Enhancer of Trithorax and Polycomb Corto Interacts with Methylated Ribosomal Protein L12 Via Its Chromodomain
Source: PLoS Genet. 2012 Oct 11;8(10):e1003006. doi: 10.1371/journal.pgen.1003006 (PMC3469418; doi:10.1371/journal.pgen.1003006)
Supplement: Table S7 — Genes de-regulated in sd::Gal4>UAS::FH-cortoCD vs sd::Gal4/+ and sd::Gal4>UAS::RpL12-Myc vs sd::Gal4/+. (PDF) [file pgen.1003006.s011.pdf]

Table S7

Genes up-regulated in *sd::Gal4>UAS::FH-cortoCD* wing discs	Genes up-regulated in *sd::Gal4>UAS::RpL12-Myc* wing discs	Shared up-regulated genes	Genes down-regulated in *sd::Gal4>UAS::FH-cortoCD* wing discs	Genes down-regulated in *sd::Gal4>UAS::RpL12-Myc* wing discs	Shared down-regulated genes
14-3-3zeta	7SLRNA:CR32864	7SLRNA:CR32864	A2bp1	4EHP	A2bp1
7SLRNA:CR32864	7SLRNA:CR42652	7SLRNA:CR42652	Act5C	A2bp1	Act5C
7SLRNA:CR42652	Aats-ala	Act57B	Adar	Act5C	Adar
Abi	Aats-glupro	Act5C	Akap200	Adar	Akap200
Act57B	Act57B	Aldh-III	akirin	Akap200	akirin
Act5C	Act5C	Arc-p20	Ank	akirin	Ank
Actn	Ahcy13	Arp11	Antp	alph	Antp
Aldh-III	Aldh-III	asrij	AP-1gamma	alpha-Man-I	AP-1gamma
Arc-p20	alphaTry	ATPsyn-d	Atf6	Ank	Atf6
Arf79F	Arc-p20	B52	bowl	Antp	bowl
Arp11	Arp11	Bet5	Bsg	AP-1gamma	Bsg
asrij	ash2	betaTub56D	bun	Asator	bun
ATPsyn-Cf6	asrij	bou	bw	Atf6	bw
ATPsyn-d	ATPsyn-b	br	CadN	bbg	CadN
awd	ATPsyn-d	brat	cals	bowl	cals
B52	B52	brm	Cam	Bsg	Cam
Bet5	Bet5	Bruce	CaMKI	bun	CaMKI
betaTub56D	betaTry	btsz	CaMKII	bw	CaMKII
bou	betaTub56D	capt	Cf2	CadN	Cf2
br	blot	Cctgamma	CG10006	cals	CG10006
brat	blw	CG10320	CG10417	Cam	CG10417
brm	BM-40-SPARC	CG10527	CG10505	CaMKI	CG10505
Bruce	bou	CG11151	CG10513	CaMKII	CG10513
btsz	br	CG11438	CG10514	CdGAPr	CG10514
capt	brat	CG11505	CG1115	Cf2	CG1115
Cchl	brm	CG11854	CG11180	CG10006	CG11180
Cctgamma	Bruce	CG11873	CG11266	CG10417	CG11266
CG10320	btsz	CG11876	CG11486	CG10505	CG11486
CG10418	capt	CG11943	CG11727	CG10513	CG11727
CG10527	Cct5	CG11999	CG11892	CG10514	CG11892
CG10664	Cctgamma	CG12203	CG12054	CG1115	CG12054
CG11015	CG10249	CG12859	CG12567	CG11180	CG12567
CG11151	CG10320	CG13044	CG12990	CG11266	CG12990
CG11267	CG10527	CG13185	CG13025	CG11486	CG13025
CG11438	CG10912	CG13319	CG14135	CG11727	CG15923
CG11455	CG11151	CG13349	CG14526	CG11892	CG16727
CG11505	CG11438	CG13393	CG15923	CG12054	CG17471
CG11699	CG11505	CG1354	CG16727	CG12567	CG17528
CG11752	CG11854	CG13551	CG17471	CG12990	CG17683
CG11753	CG11873	CG13630	CG17528	CG13025	CG17698
CG11854	CG11876	CG13731	CG17683	CG13360	CG17715
CG11873	CG11943	CG14184	CG17698	CG14292	CG17752
CG11876	CG11999	CG14235	CG17715	CG14464	CG2225
CG11943	CG12115	CG14302	CG17752	CG15155	CG2233
CG11999	CG12125	CG14332	CG2187	CG15406	CG30343
CG12203	CG12203	CG14566	CG2225	CG15535	CG32016
CG12384	CG12859	CG15523	CG2233	CG15628	CG3264
CG12432	CG13044	CG1607	CG30343	CG15923	CG3292
CG12848	CG13185	CG16936	CG32016	CG16727	CG3999
CG12859	CG13319	CG17202	CG3264	CG17159	CG40196
CG12935	CG13323	CG1746	CG3292	CG17471	CG40351
CG13041	CG13324	CG1753	CG3999	CG17490	CG41520
CG13044	CG13349	CG18809	CG40196	CG17528	CG42235
CG13053	CG13393	CG2021	CG40351	CG17683	CG42258
CG13185	CG1354	CG2200	CG41520	CG17698	CG4502
CG13319	CG13551	CG2310	CG42235	CG17715	CG4662
CG13349	CG13630	CG2812	CG42258	CG17752	CG4768
CG13364	CG13731	CG30025	CG4502	CG17883	CG5059
CG13393	CG14184	CG30031	CG4662	CG18812	CG5065
CG1354	CG14235	CG30185	CG4768	CG2225	CG6700
CG13551	CG14302	CG30410	CG5059	CG2233	CG7115
CG13630	CG14332	CG30415	CG5065	CG2316	CG7367
CG13731	CG14566	CG30499	CG5958	CG2698	CG7882
CG13751	CG14619	CG32038	CG6357	CG30343	CG7971
CG13926	CG15044	CG3214	CG6700	CG31121	CG8116
CG13993	CG15523	CG3321	CG7115	CG31360	CG8419
CG14057	CG15818	CG33333	CG7367	CG32016	CG8500
CG14104	CG1607	CG34227	CG7882	CG32350	CG8979
CG14184	CG1620	CG34306	CG7971	CG3264	CG9259
CG14235	CG16936	CG34347	CG8116	CG32709	CG9894
CG14302	CG1702	CG34383	CG8419	CG32856	cib
CG14332	CG17121	CG34417	CG8500	CG3292	CkIalpha
CG14482	CG17202	CG34422	CG8745	CG3999	CkIIbeta
CG14566	CG1746	CG3446	CG8979	CG40160	CR41597
CG14997	CG1753	CG3500	CG9259	CG40178	CR42722
CG15012	CG18809	CG3609	CG9894	CG40191	CR42723
CG15237	CG1943	CG3731	cib	CG40196	CR43241
CG15523	CG2021	CG42238	CkIalpha	CG40228	CrebA
CG1607	CG2200	CG42455	CkIIbeta	CG40351	crq
CG16936	CG2310	CG42497	CR41597	CG41454	Cyp6g1
CG1707	CG2812	CG42500	CR42722	CG41520	dbr
CG17202	CG30025	CG42574	CR42723	CG42235	Dcp2
CG17343	CG30031	CG42834	CR43241	CG42258	Df31
CG17374	CG30185	CG4692	CrebA	CG42342	Dsp1
CG1746	CG30410	CG5021	crq	CG42724	Dyb
CG17508	CG30415	CG5261	Cyp6g1	CG4502	Dyrk3
CG1753	CG30499	CG5446	dbr	CG4612	E2f
CG17680	CG31676	CG5527	Dcp2	CG4662	eIF-4B
CG17776	CG31789	CG5548	Df31	CG4768	eIF5
CG18343	CG31937	CG5794	Dsp1	CG5059	Eph
CG18809	CG32038	CG5903	Dyb	CG5065	eyg
CG1969	CG3214	CG5938	Dyrk3	CG6084	Fas3

CG2021	CG32249	CG5941	E2f	CG6091	Fbp2
CG2200	CG32302	CG6543	eIF-4B	CG6700	Fst
CG2310	CG32529	CG6746	eIF5	CG7115	fwe
CG2812	CG33156	CG6793	Eph	CG7337	Galpha49B
CG2862	CG3321	CG7267	eyg	CG7367	Gef26
CG30025	CG33333	CG7414	Fas3	CG7668	gish
CG30031	CG33346	CG7580	Fbp1	CG7882	grk
CG30185	CG34227	CG7637	Fbp2	CG7971	gro
CG30410	CG34306	CG7834	Fst	CG8116	gus
CG30415	CG34347	CG8036	fwe	CG8419	H
CG30423	CG34383	CG8184	Galpha49B	CG8500	heph
CG30499	CG34417	CG8191	Gef26	CG8949	how
CG31126	CG34422	CG8664	gish	CG8979	Hr39
CG31548	CG3446	CG9027	grk	CG9259	Hr46
CG32038	CG3500	CG9065	gro	CG9821	hth
CG32069	CG3523	CG9205	Gs1	CG9894	inx2
CG3214	CG3609	CG9350	gus	ci	kn
CG32212	CG3731	CG9603	H	cib	l(2)s5379
CG32276	CG42238	CG9674	heph	cic	lin19
CG3321	CG42455	CG9775	HmgZ	CkIalpha	lola
CG33333	CG42497	cher	how	CkIIbeta	Mbs
CG34227	CG42500	cic	Hr39	clumsy	Mhcl
CG34242	CG42574	CoVa	Hr46	CR30055	Mitf
CG34250	CG42669	Cpr49Ag	Hsc70-3	CR41597	Mkk4
CG34306	CG42834	CR12628	hth	CR41604	Mnt
CG34347	CG4542	CR31144	inx2	CR42722	mrj
CG34383	CG4692	CR33222	kn	CR42723	mRpS5
CG34417	CG4769	CR34335	l(2)s5379	CR43241	mt:ATPase6
CG34422	CG5021	CR40502	lin19	CrebA	mt:ATPase8
CG34439	CG5261	CR40546	lola	crq	mt:CoI
CG3446	CG5446	CR40560	lolal	Csk	mt:CoII
CG3500	CG5527	CR40596	Lsd-1	csw	mt:CoIII
CG3560	CG5548	CR40639	Mbs	Cyp6g1	mt:Cyt-b
CG3566	CG5794	CR40640	Mhcl	dally	mt:lrRNA
CG3609	CG5903	CR40641	Mitf	dbr	mt:ND1
CG3621	CG5938	CR40642	Mkk4	Dcp2	mt:ND2
CG3625	CG5941	CR40668	Mnt	Df31	mt:ND3
CG3731	CG6543	CR40677	mrj	Dh44-R2	mt:ND4
CG4036	CG6746	CR40728	mRpS5	Dsp1	mt:ND4L
CG42238	CG6769	CR40766	mt:ATPase6	Dyb	mt:ND5
CG42239	CG6793	CR41535	mt:ATPase8	Dyrk3	mt:ND6
CG42377	CG6933	CR41539	mt:CoI	E2f	mt:srRNA
CG42455	CG7267	CR41540	mt:CoII	eIF-4a	mt:tRNA:C
CG42497	CG7414	CR41544	mt:CoIII	eIF-4B	mt:tRNA:G
CG42500	CG7465	CR41548	mt:Cyt-b	eIF4G	mt:tRNA:L:CUN
CG42574	CG7580	CR41583	mt:lrRNA	eIF5	mt:tRNA:L:UUR
CG42834	CG7637	CR41602	mt:ND1	ens	mt:tRNA:P
CG4692	CG7834	CR41609	mt:ND2	Eph	mt:tRNA:W
CG5021	CG7953	CR41613	mt:ND3	ewg	mt:tRNA:Y
CG5261	CG8036	Cyt-b5	mt:ND4	eyg	Muc11A
CG5446	CG8111	deltaTry	mt:ND4L	Fas3	Mur18B
CG5527	CG8184	Dhc64C	mt:ND5	fbl6	myoglianin
CG5548	CG8191	dikar	mt:ND6	Fbp2	nimC2
CG5569	CG8193	Doa	mt:srRNA	fs(1)h	Nipped-B
CG5703	CG8399	dom	mt:tRNA:C	Fst	Nos
CG5794	CG8661	dp	mt:tRNA:G	fwe	Nrg
CG5903	CG8664	Ef2b	mt:tRNA:L:CUN	Galpha49B	ogre
CG5938	CG8997	eIF-3p40	mt:tRNA:L:UUR	Gef26	pAbp
CG5941	CG9027	fabp	mt:tRNA:P	gish	Pabp2
CG6543	CG9065	ft	mt:tRNA:W	grh	pallidin
CG6746	CG9172	futsch	mt:tRNA:Y	grk	par-1
CG6793	CG9205	fwd	Muc11A	gro	Parp
CG6878	CG9331	gammaTry	Mur18B	gus	Pdk1
CG6891	CG9350	glo	myoglianin	H	Pdp1
CG7181	CG9436	Got2	nimC2	Haspin	pho
CG7267	CG9603	hang	Nipped-B	heph	Pi4KIIalpha
CG7414	CG9674	HERC2	Nos	how	plexA
CG7580	CG9775	Hsc70-4	Nrg	Hr39	PMCA
CG7603	cher	Hsp26	ogre	Hr46	pncr013:4
CG7630	chic	Hsp60	pAbp	Hrb98DE	PRL-1
CG7637	cic	Hsp68	Pabp2	hth	prominin-like
CG7834	CoVa	Hsp70Aa	pallidin	Imp	Ptp10D
CG8036	cp309	Hsp70Ab	par-1	inx2	Pur-alpha
CG8184	Cpr49Ag	Hsp70Ba	Parp	itp	qkr54B
CG8191	CR12628	Hsp70Bb	Pdk1	jim	qkr58E-3
CG8204	CR31144	Hsp70Bbb	Pdp1	kn	R
CG8206	CR33222	Hsp70Bc	pho	knrl	Rbp1-like
CG8664	CR34335	Jon25Bi	Pi4KIIalpha	l(2)s5379	Rbp2
CG8891	CR40502	Jon65Aiii	plexA	lin19	RecQ5
CG9027	CR40546	Jon65Aiv	PMCA	Lis-1	Rfabg
CG9034	CR40560	kek5	pncr013:4	lola	Sap47
CG9065	CR40596	kis	PRL-1	Marf	Scm
CG9205	CR40639	ksh	prominin-like	mask	Sdc
CG9240	CR40640	l(1)G0230	Ptp10D	Mbs	sdt
CG9336	CR40641	l(2)35Di	Pur-alpha	Mhcl	sgg
CG9350	CR40642	larp	qkr54B	mim	Sh3beta
CG9603	CR40668	LBR	qkr58E-3	Mitf	shi
CG9669	CR40677	Lcp1	R	Mkk4	sky
CG9674	CR40728	Lcp2	Rbp1-like	Mmp1	stai
CG9775	CR40766	Lcp3	Rbp2	Mnt	swi2
cher	CR41535	Lcp4	RecQ5	mp	syd
cic	CR41539	levy	Rfabg	mrj	tal-1A
cl	CR41540	LRP1	Sap47	mRpS21	tal-2A
CoVa	CR41544	m2	Scm	mRpS5	tal-3A
Cpr49Ag	CR41548	mamo	Sdc	mt:ATPase6	tal-AA
Cpr49Ah	CR41583	mask	sdt	mt:ATPase8	TBPH
Cpr65Ec	CR41602	Megalin	sgg	mt:CoI	tna
Cpr66D	CR41609	Mgstl	Sh3beta	mt:CoII	TpnC25D
Cpr78E	CR41613	Mhc	shi	mt:CoIII	UbcD2
CR12628	CycG	Mi-2	sky	mt:Cyt-b	UBL3
CR31144	Cyp4e2	Mical	stai	mt:lrRNA	unk
CR33222	Cyt-b5	mRpL22	swi2	mt:ND1	ventrally-expressed-protein-D
CR34335	deltaTry	mRpL27	syd	mt:ND2	vn

CR40469	*Dhc64C*	*mRpL33*	*tal-1A*	*mt:ND3*	*zip*
CR40502	*dikar*	*mRpL49*	*tal-2A*	*mt:ND4*	*Zyx*
CR40546	*Doa*	*mRpL52*	*tal-3A*	*mt:ND4L*	
CR40560	*dom*	*mRpS16*	*tal-AA*	*mt:ND5*	
CR40596	*dp*	*Msp-300*	*TBPH*	*mt:ND6*	
CR40621	*Dph5*	*N*	*tna*	*mt:srRNA*	
CR40639	*Dpy-30L1*	*nej*	*TotA*	*mt:tRNA:C*	
CR40640	*dro2*	*nocte*	*TpnC25D*	*mt:tRNA:G*	
CR40641	*Ef1beta*	*NP15.6*	*tup*	*mt:tRNA:L:CUN*	
CR40642	*Ef2b*	*Obp56d*	*UbcD2*	*mt:tRNA:L:UUR*	
CR40668	*EfTuM*	*Obp83g*	*UBL3*	*mt:tRNA:P*	
CR40677	*eIF-2alpha*	*obst-B*	*unk*	*mt:tRNA:W*	
CR40728	*eIF-3p40*	*olf186-F*	*ventrally-expressed-protein-D*	*mt:tRNA:Y*	
CR40766	*fabp*	*osa*	*vn*	*mub*	
CR40959	*faf*	*Oscp*	*zip*	*Muc11A*	
CR40963	*Fer1HCH*	*p16-ARC*	*Zyx*	*Mur18B*	
CR41535	*ft*	*pck*		*myoglianin*	
CR41539	*fu12*	*pcx*		*N*	
CR41540	*futsch*	*Pdsw*		*Nedd4*	
CR41544	*fwd*	*PHGPx*		*Nhe3*	
CR41548	*fzo*	*pix*		*nimC2*	
CR41583	*gammaTry*	*poe*		*Nipped-B*	
CR41602	*glo*	*Pros29*		*norpA*	
CR41609	*Got2*	*Rack1*		*Nos*	
CR41613	*hang*	*Rbcn-3A*		*Nrg*	
CR43334	*HERC2*	*RFeSP*		*ogre*	
Cyp4g1	*Hsc70-4*	*rg*		*ovo*	
Cypl	*Hsp26*	*rhea*		*pAbp*	
Cyt-b5	*Hsp60*	*RNaseMRP:RNA*		*Pabp2*	
deltaTry	*Hsp68*	*RNaseP:RNA*		*pallidin*	
Dhc64C	*Hsp70Aa*	*rno*		*par-1*	
dikar	*Hsp70Ab*	*Roc1a*		*Parp*	
Doa	*Hsp70Ba*	*RpII15*		*Patronin*	
dom	*Hsp70Bb*	*RpL10Ab*		*Pdk1*	
dp	*Hsp70Bbb*	*RpL11*		*Pdp1*	
Dsp1	*Hsp70Bc*	*RpL12*		*pho*	
Ef2b	*Ing3*	*RpL13*		*Pi4KIIalpha*	
eIF-3p40	*Jon25Bi*	*RpL13A*		*piwi*	
Elongin-C	*Jon25Bii*	*RpL17*		*Pkn*	
fabp	*Jon65Aiii*	*RpL18*		*plexA*	
fd68A	*Jon65Aiv*	*RpL18A*		*PMCA*	
ft	*kek5*	*RpL24*		*pncr013:4*	
futsch	*kis*	*RpL26*		*PP2A-B'*	
fwd	*ksh*	*RpL27*		*PRL-1*	
gammaTry	*kst*	*RpL28*		*prominin-like*	
glo	*l(1)G0230*	*RpL29*		*Pten*	
Got2	*l(1)G0334*	*RpL3*		*Ptp10D*	
GstD1	*l(2)03659*	*RpL31*		*pUf68*	
GstS1	*l(2)03709*	*RpL32*		*Pur-alpha*	
hang	*l(2)35Di*	*RpL34a*		*qkr54B*	
HERC2	*l(2)tid*	*RpL34b*		*qkr58E-3*	
hoip	*larp*	*RpL35*		*R*	
HP4	*LBR*	*RpL35A*		*Ranbp16*	
Hsc70-4	*Lcp1*	*RpL36*		*Rbp1-like*	
Hsp26	*Lcp2*	*RpL36A*		*Rbp2*	
Hsp60	*Lcp3*	*RpL37A*		*RecQ5*	
Hsp68	*Lcp4*	*RpL40*		*Rfabg*	
Hsp70Aa	*levy*	*RpL8*		*rl*	
Hsp70Ab	*LRP1*	*RpL9*		*RpL38*	
Hsp70Ba	*m2*	*RpLP1*		*Sap47*	
Hsp70Bb	*mamo*	*RpS10b*		*Scm*	
Hsp70Bbb	*mask*	*RpS11*		*Sdc*	
Hsp70Bc	*Mdh1*	*RpS12*		*sdt*	
HSPC300	*MED14*	*RpS13*		*sgg*	
Idh	*Megalin*	*RpS14a*		*Sh3beta*	
janA	*Mgstl*	*RpS14b*		*shi*	
Jon25Bi	*Mhc*	*RpS15*		*sky*	
Jon65Aiii	*Mi-2*	*RpS15Ab*		*slow*	
Jon65Aiv	*Mical*	*RpS17*		*sm*	
kek5	*MrgBP*	*RpS18*		*smg*	
kis	*mRpL22*	*RpS19a*		*snmRNA:838*	
ksh	*mRpL27*	*RpS20*		*spo*	
l(1)G0230	*mRpL33*	*RpS21*		*spz4*	
l(1)G0255	*mRpL35*	*RpS23*		*stai*	
l(2)35Di	*mRpL49*	*RpS26*		*Stlk*	
l(2)37Cg	*mRpL52*	*RpS3*		*swi2*	
l(2)efl	*mRpS16*	*RpS30*		*syd*	
larp	*mRpS29*	*RpS4*		*tal-1A*	
LBR	*mRpS35*	*RpS5a*		*tal-2A*	
Lcp1	*Mrtf*	*RpS6*		*tal-3A*	
Lcp2	*Msp-300*	*RpS7*		*tal-AA*	
Lcp3	*Mur29B*	*RpS8*		*Tao-1*	
Lcp4	*N*	*RpS9*		*TBPH*	
levy	*nej*	*rut*		*tlk*	
Lin29	*nocte*	*scu*		*tna*	
LRP1	*NP15.6*	*sea*		*TpnC25D*	
m2	*Obp56d*	*shot*		*Tsp39D*	
malpha	*Obp83g*	*skpA*		*Tsp42Ea*	
mamo	*obst-B*	*sls*		*UbcD2*	
mask	*olf186-F*	*Smr*		*UBL3*	
Megalin	*osa*	*Sod*		*unc-13*	
mei-P26	*Oscp*	*spen*		*unk*	
Mgstl	*p16-ARC*	*sta*		*ventrally-expressed-protein-D*	
Mhc	*pck*	*stan*		*vfl*	
Mi-2	*pcx*	*Taf10*		*vn*	
Mical	*Pdsw*	*Tao-1*		*vtd*	
Mlc2	*Pglym78*	*Ten-a*		*Wnt4*	
mRpL17	*Phae1*	*Ten-m*		*zfh2*	
mRpL18	*PHGPx*	*Tfb1*		*zip*	
mRpL22	*pix*	*TfIIA-S*		*Zyx*	
mRpL27	*poe*	*Tim10*			
mRpL33	*Ppox*	*Tom7*			
mRpL42	*proPO-A1*	*Tpi*			

mRpL48	Pros29	trol
mRpL49	Prosalpha5	trx
mRpL51	px	Trxr-1
mRpL52	r	tutl
mRpL55	Rack1	tweek
mRpS11	raptor	tyf
mRpS14	Rbcn-3A	uif
mRpS16	RFeSP	Updo
Msp-300	rg	vfl
N	rhea	Vha68-2
Neb-cGP	RNaseMRP:RNA	vnc
nej	RNaseP:RNA	vsg
nocte	rno	w
Not1	Roc1a	yip7
NP15.6	RpII15	zormin
Nurf-38	RpL10Ab	zye
Nxt1	RpL11	
Obp56d	RpL12	
Obp83g	RpL13	
Obp99a	RpL13A	
obst-B	RpL17	
olf186-F	RpL18	
Or2a	RpL18A	
osa	RpL24	
Oscp	RpL26	
ox	RpL27	
p16-ARC	RpL28	
Pcd	RpL29	
pck	RpL3	
Pcmt	RpL31	
pcx	RpL32	
PDCD-5	RpL34a	
Pdsw	RpL34b	
PHGPx	RpL35	
pix	RpL35A	
ple	RpL36	
pncr002:3R	RpL36A	
poe	RpL37a	
primo-1	RpL40	
primo-2	RpL8	
Pros29	RpL9	
Prosbeta1	RpLP1	
Rack1	RpS10b	
Rbcn-3A	RpS11	
Rcd4	RpS12	
RFeSP	RpS13	
rg	RpS14a	
rhea	RpS14b	
RNaseMRP:RNA	RpS15	
RNaseP:RNA	RpS15Ab	
rno	RpS17	
Roc1a	RpS18	
Rpb11	RpS19a	
Rpb12	RpS20	
RpII15	RpS21	
RpL10Ab	RpS23	
RpL11	RpS26	
RpL12	RpS3	
RpL13	RpS30	
RpL13A	RpS4	
RpL14	RpS5a	
RpL17	RpS6	
RpL18	RpS7	
RpL18A	RpS8	
RpL24	RpS9	
RpL26	Rpt3	
RpL27	rut	
RpL28	Sac1	
RpL29	scf	
RpL3	Scsalpha	
RpL30	scu	
RpL31	sdt	
RpL32	sea	
RpL34a	Sgs3	
RpL34b	shot	
RpL35	Sin	
RpL35A	skpA	
RpL36	sls	
RpL36A	Smr	
RpL37A	Sod	
RpL40	Sop2	
RpL41	Sp1	
RpL7A	spen	
RpL8	Spn27A	
RpL9	Spp	
RpLP1	sta	
RpS10b	stan	
RpS11	Taf10	
RpS12	Tao-1	
RpS13	Ten-a	
RpS14a	Ten-m	
RpS14b	Tfb1	
RpS15	TfIIA-L	
RpS15Ab	TfIIA-S	
RpS17	Tig	
RpS18	Tim10	
RpS19a	Tom7	
RpS20	Tpi	
RpS21	trol	
RpS23	trx	
RpS26	Trxr-1	
RpS28b	Tsp	
RpS3	tutl	

RpS30	*tweek*
RpS4	*tyf*
RpS5a	*Ubp64E*
RpS6	*uif*
RpS7	*Updo*
RpS8	*vari*
RpS9	*vav*
rut	*verm*
scu	*vfl*
SdhC	*Vha68-2*
sdk	*vnc*
sea	*vsg*
shot	*w*
skpA	*wdb*
sli	*yip7*
sls	*zormin*
SmD2	*Zw*
SmF	*zye*
Smr	
sni	
snRNA:U12:73B	
Sod	
Spase12	
Spase22-23	
spen	
sphinx	
sta	
stan	
stv	
sun	
Taf10	
Tao-1	
Ten-a	
Ten-m	
Tfb1	
TfIIA-S	
Tim10	
Tina-1	
Tom7	
Tpi	
trol	
trx	
Trxr-1	
tutl	
tweek	
tyf	
uif	
Updo	
vfl	
Vha100-2	
Vha44	
Vha68-2	
vnc	
Vps28	
vsg	
w	
wb	
Wnk	
yip7	
zormin	
zye	
