# Supplementary material for: New Partners in Regulation of Gene Expression: The Enhancer of Trithorax and Polycomb Corto Interacts with Methylated Ribosomal Protein L12 Via Its Chromodomain
Source: PLoS Genet. 2012 Oct 11;8(10):e1003006. doi: 10.1371/journal.pgen.1003006 (PMC3469418; doi:10.1371/journal.pgen.1003006)
Supplement: Table S8 — Ontology of genes up-regulated in sd::Gal4>UAS::FH-cortoCD vs sd::Gal4/+. (PDF) [file pgen.1003006.s012.pdf]

Table S8

sd::Gal4>UAS::FH-cortoCD	**up-regulated genes**					
**GO ID**	**Description**	**Category**	**GO CortoCD up**	**GO total**	**padj dh**	**Enrichment**
GO:0006412	translation	Biological process	105	511	5,33000E-59	7,13183
GO:0007052	mitotic spindle organization	Biological process	39	209	4,87000E-19	6,47665
GO:0000022	mitotic spindle elongation	Biological process	35	84	5,92000E-30	14,46180
GO:0022008	neurogenesis	Biological process	29	557	8,61400E-03	1,80707
GO:0000398	nuclear mRNA splicing, via spliceosome	Biological process	28	417	3,88092E-04	2,33053
GO:0009408	response to heat	Biological process	23	93	8,59000E-14	8,58375
GO:0007411	axon guidance	Biological process	21	278	6,46852E-04	2,62184
GO:0008340	determination of adult lifespan	Biological process	20	160	8,54000E-07	4,33853
GO:0048813	dendrite morphogenesis	Biological process	20	197	2,03000E-05	3,52368
GO:0001666	response to hypoxia	Biological process	13	41	3,21000E-09	11,00500
GO:0015992	proton transport	Biological process	12	40	3,01000E-08	10,41250
GO:0006457	protein folding	Biological process	12	140	6,45273E-03	2,97499
GO:0006120	mitochondrial electron transport, NADH to ubiquinone	Biological process	11	34	6,07000E-08	11,22910
GO:0006123	mitochondrial electron transport, cytochrome c to oxygen	Biological process	9	17	1,09000E-08	18,37490
GO:0016339	calcium-dependent cell-cell adhesion	Biological process	9	43	6,61000E-05	7,26451
GO:0042067	establishment of ommatidial planar polarity	Biological process	8	45	5,74748E-04	6,17035
GO:0001736	establishment of planar polarity	Biological process	8	55	2,01248E-03	5,04847
GO:0015986	ATP synthesis coupled proton transport	Biological process	7	25	1,08471E-04	9,71830
GO:0016458	gene silencing	Biological process	7	32	4,62982E-04	7,59242
GO:0008587	imaginal disc-derived wing margin morphogenesis	Biological process	7	38	1,27531E-03	6,39362
GO:0016203	muscle attachment	Biological process	7	40	1,69778E-03	6,07394
GO:0007156	homophilic cell adhesion	Biological process	7	46	3,60910E-03	5,28169
GO:0006122	mitochondrial electron transport, ubiquinol to cytochrome c	Biological process	6	13	2,02000E-05	16,01920
GO:0001737	establishment of imaginal disc-derived wing hair orientation	Biological process	6	37	6,45273E-03	5,62836
GO:0006464	protein modification process	Biological process	6	37	6,45273E-03	5,62836
GO:0006417	regulation of translation	Biological process	5	17	1,31501E-03	10,20830
GO:0007016	cytoskeletal anchoring at plasma membrane	Biological process	5	20	2,72343E-03	8,67706
GO:0042066	perineurial glial growth	Biological process	4	11	2,59640E-03	12,62120
GO:0043524	negative regulation of neuron apoptosis	Biological process	4	12	3,57898E-03	11,56940
GO:0007519	skeletal muscle tissue development	Biological process	4	15	8,06719E-03	9,25553
GO:0046331	lateral inhibition	Biological process	4	16	9,03428E-03	8,67706
GO:0003735	structural constituent of ribosome	Molecular function	136	326	8,78000E-126	14,47950
GO:0008553	hydrogen-exporting ATPase activity, phosphorylative mechanism	Molecular function	32	113	2,81000E-21	9,82888
GO:0003779	actin binding	Molecular function	26	202	6,02000E-09	4,46739
GO:0005214	structural constituent of chitin-based cuticle	Molecular function	21	256	2,56151E-04	2,84716
GO:0004129	cytochrome-c oxidase activity	Molecular function	18	42	2,05000E-15	14,87500
GO:0004364	glutathione transferase activity	Molecular function	14	66	1,46000E-07	7,36235
GO:0005200	structural constituent of cytoskeleton	Molecular function	14	67	1,67000E-07	7,25246
GO:0003954	NADH dehydrogenase activity	Molecular function	11	37	1,48000E-07	10,31870
GO:0008121	ubiquinol-cytochrome-c reductase activity	Molecular function	10	18	6,75000E-10	19,28230
GO:0008137	NADH dehydrogenase (ubiquinone) activity	Molecular function	9	52	2,89676E-04	6,00719
GO:0008010	structural constituent of chitin-based larval cuticle	Molecular function	9	61	8,08884E-04	5,12089
GO:0042623	ATPase activity, coupled	Molecular function	9	87	8,10495E-03	3,59051
GO:0008017	microtubule binding	Molecular function	9	88	8,18785E-03	3,54970
GO:0004550	nucleoside diphosphate kinase activity	Molecular function	8	12	9,23000E-09	23,13880
GO:0003713	transcription coactivator activity	Molecular function	8	44	4,95016E-04	6,31059
GO:0003899	DNA-directed RNA polymerase activity	Molecular function	8	65	5,60861E-03	4,27178
GO:0003993	acid phosphatase activity	Molecular function	4	15	8,06719E-03	9,25553
GO:0005811	lipid particle	Cellular component	75	339	1,69000E-43	7,67881
GO:0005875	microtubule associated complex	Cellular component	48	359	2,31000E-17	4,64065
GO:0022625	cytosolic large ribosomal subunit	Cellular component	47	79	4,15000E-50	20,64920
GO:0005737	cytoplasm	Cellular component	43	859	1,96990E-03	1,73743
GO:0022627	cytosolic small ribosomal subunit	Cellular component	40	63	3,83000E-44	22,03700
GO:0005840	ribosome	Cellular component	30	65	3,22000E-27	16,01920
GO:0005739	mitochondrion	Cellular component	23	333	1,03516E-03	2,39726
GO:0016021	integral to membrane	Cellular component	21	1302	8,18785E-03	0,55981
GO:0005747	mitochondrial respiratory chain complex I	Cellular component	19	71	4,99000E-12	9,28812
GO:0030532	small nuclear ribonucleoprotein complex	Cellular component	15	104	6,75000E-06	5,00599
GO:0005762	mitochondrial large ribosomal subunit	Cellular component	14	66	1,46000E-07	7,36235
GO:0005751	mitochondrial respiratory chain complex IV	Cellular component	13	24	1,08000E-12	18,80030
GO:0005750	mitochondrial respiratory chain complex III	Cellular component	11	20	7,80000E-11	19,08950
GO:0005681	spliceosomal complex	Cellular component	11	107	2,72980E-03	3,56814
GO:0030018	Z disc	Cellular component	9	24	3,64000E-07	13,01560
GO:0000276	mitochondrial proton-transporting ATP synthase complex, coupling factor F(o	Cellular component	7	17	6,75000E-06	14,29160
GO:0016589	NURF complex	Cellular component	6	21	4,01453E-04	9,91664
GO:0005884	actin filament	Cellular component	6	29	1,96990E-03	7,18101
GO:0005665	DNA-directed RNA polymerase II, core complex	Cellular component	6	34	4,32027E-03	6,12498
GO:0000221	vacuolar proton-transporting V-type ATPase, V1 domain	Cellular component	6	36	5,75482E-03	5,78470
GO:0016585	chromatin remodeling complex	Cellular component	4	9	1,18597E-03	15,42590
