# Supplementary material for: New Partners in Regulation of Gene Expression: The Enhancer of Trithorax and Polycomb Corto Interacts with Methylated Ribosomal Protein L12 Via Its Chromodomain
Source: PLoS Genet. 2012 Oct 11;8(10):e1003006. doi: 10.1371/journal.pgen.1003006 (PMC3469418; doi:10.1371/journal.pgen.1003006)
Supplement: Table S9 — Ontology of genes down-regulated in sd::Gal4>UAS::FH-cortoCD vs sd::Gal4/+. (PDF) [file pgen.1003006.s013.pdf]

Table S9

sd::Gal4>UAS::FH-cortoCD	**Down-regulated genes**					
**GO ID**	**Description**	**Category**	**GO CortoCD down**	**GO total**	**padj dh**	**Enrichment**
GO:0006468	protein phosphorylation	Biological process	39	545	1,63623E-14	4,90240
GO:0006355	regulation of transcription, DNA-dependent	Biological process	25	520	4,66378E-06	3,29365
GO:0007411	axon guidance	Biological process	16	278	7,19313E-05	3,94290
GO:0006470	protein dephosphorylation	Biological process	11	181	9,76596E-04	4,16346
GO:0007391	dorsal closure	Biological process	11	222	3,63564E-03	3,39453
GO:0000910	cytokinesis	Biological process	10	144	7,40291E-04	4,75749
GO:0007314	oocyte anterior/posterior axis specification	Biological process	8	38	2,62282E-06	14,42270
GO:0016339	calcium-dependent cell-cell adhesion	Biological process	8	43	6,15976E-06	12,74560
GO:0008045	motor axon guidance	Biological process	8	58	5,06639E-05	9,44936
GO:0007623	circadian rhythm	Biological process	8	106	1,98046E-03	5,17040
GO:0007173	epidermal growth factor receptor signaling pathway	Biological process	7	51	1,75769E-04	9,40304
GO:0016319	mushroom body development	Biological process	7	88	3,00987E-03	5,44949
GO:0048096	chromatin-mediated maintenance of transcription	Biological process	6	21	1,33277E-05	19,57370
GO:0045197	establishment or maintenance of epithelial cell apical/basal polarity	Biological process	6	37	2,67995E-04	11,10940
GO:0000122	negative regulation of transcription from RNA polymerase II promoter	Biological process	6	81	8,93779E-03	5,07466
GO:0016325	oocyte microtubule cytoskeleton organization	Biological process	5	20	1,75769E-04	17,12700
GO:0007613	memory	Biological process	5	26	5,98728E-04	13,17460
GO:0016199	axon midline choice point recognition	Biological process	5	29	9,41246E-04	11,81170
GO:0007279	pole cell formation	Biological process	5	36	2,15059E-03	9,51498
GO:0006897	endocytosis	Biological process	5	49	6,23756E-03	6,99060
GO:0045879	negative regulation of smoothened signaling pathway	Biological process	4	19	1,99002E-03	14,42270
GO:0006471	protein ADP-ribosylation	Biological process	4	21	2,59000E-03	13,04910
GO:0016458	gene silencing	Biological process	4	32	9,28695E-03	8,56348
GO:0008284	positive regulation of cell proliferation	Biological process	4	32	9,28695E-03	8,56348
GO:0045196	establishment or maintenance of neuroblast polarity	Biological process	3	10	3,76314E-03	20,55240
GO:0010628	positive regulation of gene expression	Biological process	3	11	4,95626E-03	18,68400
GO:0008069	dorsal/ventral axis specification, ovarian follicular epithelium	Biological process	3	11	4,95626E-03	18,68400
GO:0035293	chitin-based larval cuticle pattern formation	Biological process	3	12	6,20375E-03	17,12700
GO:0046665	amnioserosa maintenance	Biological process	3	13	7,13897E-03	15,80950
GO:0006541	glutamine metabolic process	Biological process	3	13	7,13897E-03	15,80950
GO:0006508	proteolysis	Biological process	1	767	1,62465E-03	0,08932
GO:0004674	protein serine/threonine kinase activity	Molecular function	35	373	2,57122E-16	6,42835
GO:0003700	sequence-specific DNA binding transcription factor activity	Molecular function	26	570	6,38596E-06	3,12492
GO:0003677	DNA binding	Molecular function	22	720	5,29163E-03	2,09330
GO:0008137	NADH dehydrogenase (ubiquinone) activity	Molecular function	19	52	2,04418E-19	25,03170
GO:0003729	mRNA binding	Molecular function	14	275	7,30575E-04	3,48767
GO:0005154	epidermal growth factor receptor binding	Molecular function	10	18	2,12265E-12	38,05990
GO:0000166	nucleotide binding	Molecular function	10	179	2,80619E-03	3,82725
GO:0008143	poly(A) RNA binding	Molecular function	9	13	2,19504E-12	47,42850
GO:0004129	cytochrome-c oxidase activity	Molecular function	9	42	4,55577E-07	14,68030
GO:0004879	ligand-dependent nuclear receptor activity	Molecular function	8	65	9,51982E-05	8,43174
GO:0004714	transmembrane receptor protein tyrosine kinase activity	Molecular function	7	30	6,60813E-06	15,98520
GO:0004683	calmodulin-dependent protein kinase activity	Molecular function	6	9	5,63042E-08	45,67190
GO:0003714	transcription corepressor activity	Molecular function	6	35	2,08578E-04	11,74420
GO:0005516	calmodulin binding	Molecular function	6	53	1,61019E-03	7,75561
GO:0042393	histone binding	Molecular function	5	15	5,06639E-05	22,83600
GO:0005044	scavenger receptor activity	Molecular function	5	42	3,63564E-03	8,15570
GO:0004385	guanylate kinase activity	Molecular function	4	14	7,27467E-04	19,57370
GO:0004708	MAP kinase kinase activity	Molecular function	4	17	1,38602E-03	16,11950
GO:0008187	poly-pyrimidine tract binding	Molecular function	3	13	7,13897E-03	15,80950
GO:0005001	transmembrane receptor protein tyrosine phosphatase activity	Molecular function	3	14	8,74457E-03	14,68030
GO:0005634	nucleus	Cellular component	73	1905	6,47121E-14	2,62524
GO:0005886	plasma membrane	Cellular component	36	594	2,03122E-11	4,15199
GO:0005739	mitochondrion	Cellular component	25	333	1,35150E-09	5,14323
GO:0005811	lipid particle	Cellular component	20	339	4,01418E-06	4,04176
GO:0005921	gap junction	Cellular component	8	31	6,10760E-07	17,67940
GO:0005747	mitochondrial respiratory chain complex I	Cellular component	7	71	1,10459E-03	6,75430
GO:0045169	fusome	Cellular component	5	54	8,93779E-03	6,34332
GO:0016323	basolateral plasma membrane	Cellular component	4	16	1,10459E-03	17,12700
GO:0043186	P granule	Cellular component	4	31	8,73491E-03	8,83972
