# Supplementary material for: New Partners in Regulation of Gene Expression: The Enhancer of Trithorax and Polycomb Corto Interacts with Methylated Ribosomal Protein L12 Via Its Chromodomain
Source: PLoS Genet. 2012 Oct 11;8(10):e1003006. doi: 10.1371/journal.pgen.1003006 (PMC3469418; doi:10.1371/journal.pgen.1003006)
Supplement: Table S10 — Ontology of genes up-regulated in sd::Gal4>UAS:RpL12-Myc vs sd::Gal4/+. (PDF) [file pgen.1003006.s014.pdf]

Table S10

sd::Gal4>UAS::RpL12-Myc	**Up-regulated genes**					
**GO ID**	**Description**	**Category**	**GO RpL12 up**	**GO total**	**padj dh**	**Enrichment**
GO:0006412	translation	Biological process	90	511	7,27440E-47	6,58457
GO:0006508	proteolysis	Biological process	36	767	4,01363E-03	1,75474
GO:0007052	mitotic spindle organization	Biological process	35	209	1,70654E-16	6,26077
GO:0000022	mitotic spindle elongation	Biological process	32	84	8,16536E-27	14,24220
GO:0009408	response to heat	Biological process	23	93	1,87494E-14	9,24593
GO:0008340	determination of adult lifespan	Biological process	20	160	3,83347E-07	4,67321
GO:0048813	dendrite morphogenesis	Biological process	17	197	3,51081E-04	3,22618
GO:0007411	axon guidance	Biological process	17	278	7,52396E-03	2,28618
GO:0007424	open tracheal system development	Biological process	15	182	1,21880E-03	3,08124
GO:0001666	response to hypoxia	Biological process	13	41	1,62555E-09	11,85400
GO:0015992	proton transport	Biological process	12	40	1,75521E-08	11,21570
GO:0006367	transcription initiation from RNA polymerase II promoter	Biological process	11	132	5,97420E-03	3,11548
GO:0006457	protein folding	Biological process	11	140	8,22940E-03	2,93745
GO:0007015	actin filament organization	Biological process	10	79	6,98586E-04	4,73237
GO:0006511	ubiquitin-dependent protein catabolic process	Biological process	10	83	9,78955E-04	4,50430
GO:0006120	mitochondrial electron transport, NADH to ubiquinone	Biological process	9	34	6,59158E-06	9,89622
GO:0016339	calcium-dependent cell-cell adhesion	Biological process	9	43	4,27003E-05	7,82492
GO:0007409	axonogenesis	Biological process	9	77	2,27425E-03	4,36976
GO:0006413	translational initiation	Biological process	9	92	5,97420E-03	3,65730
GO:0006414	translational elongation	Biological process	8	35	7,13606E-05	8,54531
GO:0042067	establishment of ommatidial planar polarity	Biological process	8	45	3,75813E-04	6,64635
GO:0001736	establishment of planar polarity	Biological process	8	55	1,24435E-03	5,43792
GO:0016579	protein deubiquitination	Biological process	7	19	1,20699E-05	13,77370
GO:0015986	ATP synthesis coupled proton transport	Biological process	7	25	7,13606E-05	10,46800
GO:0016458	gene silencing	Biological process	7	32	3,51081E-04	8,17813
GO:0008587	imaginal disc-derived wing margin morphogenesis	Biological process	7	38	9,11490E-04	6,88684
GO:0016203	muscle attachment	Biological process	7	40	1,11856E-03	6,54250
GO:0007156	homophilic cell adhesion	Biological process	7	46	2,27425E-03	5,68913
GO:0048149	behavioral response to ethanol	Biological process	7	50	3,45183E-03	5,23400
GO:0007474	imaginal disc-derived wing vein specification	Biological process	7	52	4,13972E-03	5,03269
GO:0006123	mitochondrial electron transport, cytochrome c to oxygen	Biological process	6	17	8,09576E-05	13,19500
GO:0001737	establishment of imaginal disc-derived wing hair orientation	Biological process	6	37	4,01363E-03	6,06255
GO:0045197	establishment or maintenance of epithelial cell apical/basal polarity	Biological process	6	37	4,01363E-03	6,06255
GO:0006417	regulation of translation	Biological process	5	17	9,78955E-04	10,99580
GO:0035159	regulation of tube length, open tracheal system	Biological process	5	27	5,96129E-03	6,92328
GO:0042051	compound eye photoreceptor development	Biological process	5	28	6,47966E-03	6,67602
GO:0042066	perineurial glial growth	Biological process	4	11	1,91047E-03	13,59480
GO:0006122	mitochondrial electron transport, ubiquinol to cytochrome c	Biological process	4	13	3,36270E-03	11,50330
GO:0007519	skeletal muscle tissue development	Biological process	4	15	5,37984E-03	9,96952
GO:0046331	lateral inhibition	Biological process	4	16	6,44084E-03	9,34643
GO:0019933	cAMP-mediated signaling	Biological process	4	18	8,80931E-03	8,30794
GO:0006468	protein phosphorylation	Biological process	3	545	2,27425E-03	0,20579
GO:0003735	structural constituent of ribosome	Molecular function	115	326	1,08289E-98	13,18820
GO:0005524	ATP binding	Molecular function	35	714	2,50026E-03	1,83263
GO:0003779	actin binding	Molecular function	29	202	7,60716E-12	5,36726
GO:0008553	hydrogen-exporting ATPase activity, phosphorylative mechanism	Molecular function	27	113	1,36658E-16	8,93287
GO:0005200	structural constituent of cytoskeleton	Molecular function	14	67	9,58383E-08	7,81194
GO:0004129	cytochrome-c oxidase activity	Molecular function	12	42	3,11327E-08	10,68160
GO:0042623	ATPase activity, coupled	Molecular function	10	87	1,25097E-03	4,29721
GO:0003743	translation initiation factor activity	Molecular function	10	113	6,46167E-03	3,30847
GO:0003954	NADH dehydrogenase activity	Molecular function	9	37	1,25147E-05	9,09382
GO:0003746	translation elongation factor activity	Molecular function	9	37	1,25147E-05	9,09382
GO:0008017	microtubule binding	Molecular function	9	88	4,79116E-03	3,82354
GO:0004175	endopeptidase activity	Molecular function	9	88	4,79116E-03	3,82354
GO:0003713	transcription coactivator activity	Molecular function	8	44	3,51081E-04	6,79740
GO:0008137	NADH dehydrogenase (ubiquinone) activity	Molecular function	8	52	9,76577E-04	5,75165
GO:0008010	structural constituent of chitin-based larval cuticle	Molecular function	8	61	2,27425E-03	4,90304
GO:0016887	ATPase activity	Molecular function	7	54	4,97431E-03	4,84630
GO:0004013	adenosylhomocysteinase activity	Molecular function	6	9	1,18288E-06	24,92380
GO:0004385	guanylate kinase activity	Molecular function	5	14	3,94988E-04	13,35200
GO:0008092	cytoskeletal protein binding	Molecular function	5	30	8,22940E-03	6,23095
GO:0005041	low-density lipoprotein receptor activity	Molecular function	4	12	2,50026E-03	12,46190
GO:0004843	ubiquitin-specific protease activity	Molecular function	4	13	3,36270E-03	11,50330
GO:0008121	ubiquinol-cytochrome-c reductase activity	Molecular function	4	18	8,80931E-03	8,30794
GO:0005811	lipid particle	Cellular component	79	339	4,47378E-50	8,71231
GO:0005875	microtubule associated complex	Cellular component	49	359	1,91400E-19	5,10279
GO:0022627	cytosolic small ribosomal subunit	Cellular component	39	63	1,18762E-43	23,14350
GO:0022625	cytosolic large ribosomal subunit	Cellular component	39	79	2,21393E-38	18,45620
GO:0005737	cytoplasm	Cellular component	39	859	4,29338E-03	1,69737
GO:0005840	ribosome	Cellular component	25	65	6,93650E-21	14,37910
GO:0005739	mitochondrion	Cellular component	22	333	9,78955E-04	2,46993
GO:0005747	mitochondrial respiratory chain complex I	Cellular component	14	71	2,01709E-07	7,37183
GO:0005751	mitochondrial respiratory chain complex IV	Cellular component	10	24	1,39890E-08	15,57740
GO:0005578	proteinaceous extracellular matrix	Cellular component	10	62	1,09686E-04	6,02995
GO:0030018	Z disc	Cellular component	8	24	4,85239E-06	12,46190
GO:0000276	mitochondrial proton-transporting ATP synthase complex, coupling factor F(o	Cellular component	7	17	5,50267E-06	15,39410
GO:0005750	mitochondrial respiratory chain complex III	Cellular component	6	20	2,17718E-04	11,21570
GO:0005884	actin filament	Cellular component	6	29	1,27088E-03	7,73498
GO:0005850	eukaryotic translation initiation factor 2 complex	Cellular component	5	11	1,24633E-04	16,99350
GO:0016585	chromatin remodeling complex	Cellular component	4	9	9,72184E-04	16,61590
GO:0005832	chaperonin-containing T-complex	Cellular component	4	10	1,27088E-03	14,95430
GO:0005853	eukaryotic translation elongation factor 1 complex	Cellular component	4	13	3,36270E-03	11,50330

Coleno
