# Supplementary material for: New Partners in Regulation of Gene Expression: The Enhancer of Trithorax and Polycomb Corto Interacts with Methylated Ribosomal Protein L12 Via Its Chromodomain
Source: PLoS Genet. 2012 Oct 11;8(10):e1003006. doi: 10.1371/journal.pgen.1003006 (PMC3469418; doi:10.1371/journal.pgen.1003006)
Supplement: Table S11 — Ontology of genes down-regulated in sd::Gal4>UAS::RpL12-Myc vs sd::Gal4/+. (PDF) [file pgen.1003006.s015.pdf]

Table S11

sd::Gal4>UAS::RpL12-Myc	**Down-regulated genes**					
**GO ID**	**Description**	**Category**	**GO RpL12 down**	**GO total**	**padj dh**	**Enrichment**
GO:0006468	protein phosphorylation	Biological process	57	545	4,69367E-23	5,22337
GO:0006355	regulation of transcription, DNA-dependent	Biological process	32	520	6,74682E-07	3,07340
GO:0006470	protein dephosphorylation	Biological process	19	181	2,56991E-07	5,24261
GO:0007411	axon guidance	Biological process	19	278	6,72468E-05	3,41335
GO:0048477	oogenesis	Biological process	17	367	6,03879E-03	2,31342
GO:0007391	dorsal closure	Biological process	15	222	5,75198E-04	3,37451
GO:0006413	translational initiation	Biological process	13	92	1,66781E-06	7,05713
GO:0016055	Wnt receptor signaling pathway	Biological process	12	117	8,31679E-05	5,12233
GO:0007476	imaginal disc-derived wing morphogenesis	Biological process	12	173	1,87101E-03	3,46424
GO:0007173	epidermal growth factor receptor signaling pathway	Biological process	11	51	2,77660E-07	10,77200
GO:0000381	regulation of alternative nuclear mRNA splicing, via spliceosome	Biological process	11	72	6,55709E-06	7,63014
GO:0000122	negative regulation of transcription from RNA polymerase II promoter	Biological process	11	81	1,83948E-05	6,78235
GO:0007155	cell adhesion	Biological process	11	141	1,34620E-03	3,89624
GO:0007314	oocyte anterior/posterior axis specification	Biological process	10	38	2,29308E-07	13,14280
GO:0007399	nervous system development	Biological process	10	133	2,92065E-03	3,75509
GO:0006915	apoptosis	Biological process	10	136	3,26456E-03	3,67226
GO:0000910	cytokinesis	Biological process	10	144	4,28099E-03	3,46825
GO:0008045	motor axon guidance	Biological process	9	58	5,54803E-05	7,74974
GO:0007224	smoothened signaling pathway	Biological process	9	82	5,75198E-04	5,48152
GO:0007298	border follicle cell migration	Biological process	9	130	7,16409E-03	3,45757
GO:0048749	compound eye development	Biological process	9	134	8,07655E-03	3,35436
GO:0016339	calcium-dependent cell-cell adhesion	Biological process	8	43	5,25417E-05	9,29167
GO:0045944	positive regulation of transcription from RNA polymerase II promoter	Biological process	8	62	5,40715E-04	6,44423
GO:0007602	phototransduction	Biological process	8	85	2,87563E-03	4,70049
GO:0016319	mushroom body development	Biological process	8	88	3,23070E-03	4,54025
GO:0008360	regulation of cell shape	Biological process	8	100	5,86915E-03	3,99542
GO:0007623	circadian rhythm	Biological process	8	106	7,71901E-03	3,76926
GO:0007064	mitotic sister chromatid cohesion	Biological process	7	10	1,42119E-08	34,95990
GO:0016199	axon midline choice point recognition	Biological process	7	29	3,97220E-05	12,05510
GO:0045746	negative regulation of Notch signaling pathway	Biological process	7	33	8,03797E-05	10,59390
GO:0045197	establishment or maintenance of epithelial cell apical/basal polarity	Biological process	7	37	1,59120E-04	9,44863
GO:0008293	torso signaling pathway	Biological process	7	43	3,82851E-04	8,13021
GO:0007268	synaptic transmission	Biological process	7	62	2,42711E-03	5,63870
GO:0030707	ovarian follicle cell development	Biological process	7	65	2,92065E-03	5,37845
GO:0048096	chromatin-mediated maintenance of transcription	Biological process	6	21	6,72468E-05	14,26940
GO:0008284	positive regulation of cell proliferation	Biological process	6	32	5,89399E-04	9,36427
GO:0007279	pole cell formation	Biological process	6	36	1,01318E-03	8,32379
GO:0008587	imaginal disc-derived wing margin morphogenesis	Biological process	6	38	1,23771E-03	7,88570
GO:0030718	germ-line stem cell maintenance	Biological process	6	39	1,39728E-03	7,68350
GO:0051726	regulation of cell cycle	Biological process	6	49	3,68634E-03	6,11544
GO:0046843	dorsal appendage formation	Biological process	6	51	4,00089E-03	5,87562
GO:0007474	imaginal disc-derived wing vein specification	Biological process	6	52	4,34342E-03	5,76262
GO:0002121	inter-male aggressive behavior	Biological process	6	58	6,87108E-03	5,16649
GO:0016325	oocyte microtubule cytoskeleton organization	Biological process	5	20	6,06526E-04	12,48570
GO:0007169	transmembrane receptor protein tyrosine kinase signaling pathway	Biological process	5	20	6,06526E-04	12,48570
GO:0030713	ovarian follicle cell stalk formation	Biological process	5	20	6,06526E-04	12,48570
GO:0008101	decapentaplegic receptor signaling pathway	Biological process	5	23	1,06795E-03	10,85710
GO:0007613	memory	Biological process	5	26	1,77020E-03	9,60437
GO:0008286	insulin receptor signaling pathway	Biological process	5	36	5,36510E-03	6,93649
GO:0007294	germarium-derived oocyte fate determination	Biological process	5	40	7,71901E-03	6,24284
GO:0019730	antimicrobial humoral response	Biological process	5	42	8,54005E-03	5,94557
GO:0008069	dorsal/ventral axis specification, ovarian follicular epithelium	Biological process	4	11	7,31454E-04	18,16100
GO:0045926	negative regulation of growth	Biological process	4	11	7,31454E-04	18,16100
GO:0010628	positive regulation of gene expression	Biological process	4	11	7,31454E-04	18,16100
GO:0031290	retinal ganglion cell axon guidance	Biological process	4	12	1,01318E-03	16,64760
GO:0048132	female germ-line stem cell division	Biological process	4	15	2,13635E-03	13,31810
GO:0046331	lateral inhibition	Biological process	4	16	2,66674E-03	12,48570
GO:0046621	negative regulation of organ growth	Biological process	4	17	3,06087E-03	11,75120
GO:0045879	negative regulation of smoothened signaling pathway	Biological process	4	19	4,00089E-03	10,51430
GO:0006471	protein ADP-ribosylation	Biological process	4	21	5,65133E-03	9,51290
GO:0000165	MAPKKK cascade	Biological process	4	24	8,07655E-03	8,32379
GO:0016360	sensory organ precursor cell fate determination	Biological process	4	25	8,78077E-03	7,99084
GO:0008347	glial cell migration	Biological process	4	26	9,79784E-03	7,68350
GO:0006376	mRNA splice site selection	Biological process	3	9	5,06747E-03	16,64760
GO:0045196	establishment or maintenance of neuroblast polarity	Biological process	3	10	6,56233E-03	14,98280
GO:0046580	negative regulation of Ras protein signal transduction	Biological process	3	10	6,56233E-03	14,98280
GO:0030308	negative regulation of cell growth	Biological process	3	12	9,48980E-03	12,48570
GO:0035293	chitin-based larval cuticle pattern formation	Biological process	3	12	9,48980E-03	12,48570
GO:0006508	proteolysis	Biological process	2	767	3,24236E-04	0,13023
GO:0004674	protein serine/threonine kinase activity	Molecular function	48	373	4,69367E-23	6,42695
GO:0005515	protein binding	Molecular function	43	705	4,52933E-09	3,04615
GO:0003700	sequence-specific DNA binding transcription factor activity	Molecular function	34	570	5,25573E-07	2,97904
GO:0005524	ATP binding	Molecular function	30	714	8,99587E-04	2,09843
GO:0003677	DNA binding	Molecular function	29	720	1,87598E-03	2,01158
GO:0003729	mRNA binding	Molecular function	26	275	4,54822E-09	4,72186
GO:0008137	NADH dehydrogenase (ubiquinone) activity	Molecular function	19	52	3,01023E-17	18,24830
GO:0004672	protein kinase activity	Molecular function	16	165	6,75288E-06	4,84293
GO:0043565	sequence-specific DNA binding	Molecular function	16	267	1,03752E-03	2,99282
GO:0000166	nucleotide binding	Molecular function	15	179	6,72468E-05	4,18515
GO:0005509	calcium ion binding	Molecular function	14	251	3,68634E-03	2,78565
GO:0003743	translation initiation factor activity	Molecular function	13	113	1,29579E-05	5,74563
GO:0008143	poly(A) RNA binding	Molecular function	11	13	2,66284E-15	42,25920
GO:0004879	ligand-dependent nuclear receptor activity	Molecular function	11	65	2,78185E-06	8,45185
GO:0005154	epidermal growth factor receptor binding	Molecular function	10	18	5,74607E-11	27,74600
GO:0003723	RNA binding	Molecular function	10	130	2,66674E-03	3,84175
GO:0004129	cytochrome-c oxidase activity	Molecular function	9	42	4,83721E-06	10,70200
GO:0004714	transmembrane receptor protein tyrosine kinase activity	Molecular function	7	30	4,92600E-05	11,65330
GO:0003730	mRNA 3'-UTR binding	Molecular function	7	34	9,45156E-05	10,28230
GO:0005516	calmodulin binding	Molecular function	7	53	1,07070E-03	6,59621
GO:0004722	protein serine/threonine phosphatase activity	Molecular function	7	84	8,07655E-03	4,16190
GO:0004683	calmodulin-dependent protein kinase activity	Molecular function	6	9	3,38762E-07	33,29520
GO:0008190	eukaryotic initiation factor 4E binding	Molecular function	6	15	9,95901E-06	19,97710
GO:0003714	transcription corepressor activity	Molecular function	6	35	8,89303E-04	8,56161
GO:0042393	histone binding	Molecular function	5	15	1,67326E-04	16,64760
GO:0008138	protein tyrosine/serine/threonine phosphatase activity	Molecular function	5	37	5,89118E-03	6,74902
GO:0005044	scavenger receptor activity	Molecular function	5	42	8,54005E-03	5,94557
GO:0004385	guanylate kinase activity	Molecular function	4	14	1,71959E-03	14,26940
GO:0004708	MAP kinase kinase activity	Molecular function	4	17	3,06087E-03	11,75120
GO:0004707	MAP kinase activity	Molecular function	4	23	7,33059E-03	8,68570
GO:0019904	protein domain specific binding	Molecular function	3	10	6,56233E-03	14,98280

GO:0004721	phosphoprotein phosphatase activity	Molecular function	3	12	9,48980E-03	12,48570
GO:0008601	protein phosphatase type 2A regulator activity	Molecular function	3	12	9,48980E-03	12,48570
GO:0000340	RNA 7-methylguanosine cap binding	Molecular function	3	12	9,48980E-03	12,48570
GO:0004252	serine-type endopeptidase activity	Molecular function	1	511	3,06087E-03	0,09774
GO:0005634	nucleus	Cellular component	112	1905	4,66931E-26	2,93627
GO:0005886	plasma membrane	Cellular component	40	594	1,37168E-09	3,36315
GO:0005737	cytoplasm	Cellular component	31	859	4,60124E-03	1,80236
GO:0005739	mitochondrion	Cellular component	22	333	2,39279E-05	3,29952
GO:0005921	gap junction	Cellular component	8	31	5,12663E-06	12,88850
GO:0008278	cohesin complex	Cellular component	7	20	3,42686E-06	17,48000
GO:0030529	ribonucleoprotein complex	Cellular component	7	37	1,59120E-04	9,44863
GO:0005747	mitochondrial respiratory chain complex I	Cellular component	7	71	4,00089E-03	4,92393
GO:0016028	rhabdomere	Cellular component	6	47	3,06087E-03	6,37567
GO:0043234	protein complex	Cellular component	6	58	6,87108E-03	5,16649
GO:0016281	eukaryotic translation initiation factor 4F complex	Cellular component	5	23	1,06795E-03	10,85710
GO:0005654	nucleoplasm	Cellular component	5	33	3,83253E-03	7,56708
GO:0016323	basolateral plasma membrane	Cellular component	4	16	2,66674E-03	12,48570
GO:0008287	protein serine/threonine phosphatase complex	Cellular component	3	10	6,56233E-03	14,98280
