# Supplementary material for: New Partners in Regulation of Gene Expression: The Enhancer of Trithorax and Polycomb Corto Interacts with Methylated Ribosomal Protein L12 Via Its Chromodomain
Source: PLoS Genet. 2012 Oct 11;8(10):e1003006. doi: 10.1371/journal.pgen.1003006 (PMC3469418; doi:10.1371/journal.pgen.1003006)
Supplement: Table S12 — Primers used for constructs and qPCRs. Note that an ATG and two nuclear localization signals (underlined) were added to the forward and reverse primers of cortoCD, respectively. For RpL12 mutants, the mutated triplet is underlined. (PDF) [file pgen.1003006.s016.pdf]

Supplementary Table 12: Primers used for constructs and qPCRs.

Note that an ATG and two nuclear localization signals (underlined) were added to the forward and reverse primers of *cortoCD*, respectively. For *RpL12* mutants, the mutated triplet is underlined.

**Constructs**	**Forward Primer (5' → 3')**	**Reverse Primer (5' → 3')**
corto	CACCATGACGATGGCCGCCTGT	CACGTTGTAGCAGGAGATCTGCG
cortoCD	CACCATGCAGCAGCAACACCACCGCCAT	GCCCACCTTGCGCTTCTTCTTTGGATCCACCTTGCGCTTCTTCTTTGGATCCTGCGACATCTGCTGGTGCTG
corto∆CD	GACGGCCAGTCTTAAGCTCGGG	TACCCGGGCGCTGTTGCTGCTGGCAGTCTG
AbdB-m	GATTTGTGCTTCGCTCTGTTCG	GGCGACACGATGTTTTGATTGTG
RpL12	CACCATGCCTCCCAAATTCGACCCAA	GACCATCTTGCGCAGCAGACGG
RpL12K3A	CACCATGCCTCCCGCCTTCGACCCAAC	GTTGGGTCGAAGGCGGGAGGCATGGTG
RpL12K10A	CACCCGGAAGTTGCCTTGGTGTACCTG	CAGGTACACCAAGGCAACTTCCGTTGG
RpL12K39A	GGTCTGTCGCCCGCCAAAATCGGTGATG	CATCACCGATTTTGGCGGGCGACAGACC
RpL12R66A	CCATCCAGAACGCCCAGGCCGCCATC	GATGGCGGCCTGGGCGTTCTGGATGG
RpL12K82A	CGCTGATCATCGCCGCTCTGAAGGA	TCCTTCAGAGCGGCGATGATCAGCG
HP1CD	CACCATGGGCAAGAAAATCGACAA CCC	TTCTTCGGACTTTCGCTTGCTTGC
RpS4	CACCATGGCTCGTGGCCCCAAGAAG	GTGGGTCTTGGCGGCCAGAC
RpS10	CACCATGTTCATTCCAAAAGCCAAT	ATTGTCGTAGCGAGAAGCAC
RpS14	CACCATGGCACCCAGGAAGGCTAAA	CAGACGACGACCACGGCGAC

**PCRs**	**Forward Primer (5' → 3')**	**Reverse Primer (5' → 3')**
2Ls	ATATCCTTAACCCCGGCATC	
2La		TGTGGATCCCATACCGTTTT
hsp70-200	TGCCAGAAAGAAAACTCGAGAAA	
hsp70-108		GACAGAGTGAGAGAGCAATAGTACAGAGA
hsp70+4	CAATTCAAACAAGCAAAGTGAACAC	
hsp70+112		TGATTCACTTTAACTTGCACTTTA
hsp70+645	ATATCTGGGCGAGAGCATCACA	
hsp70+718		GTAGCCTGGCGCTGGGAGTC
hsp70+1649	GGGTGTGCCCCAGATAGAAG	
hsp+1754		TGTCGTTCTTGATCGTGATGTTC
