# Supplementary material for: New Partners in Regulation of Gene Expression: The Enhancer of Trithorax and Polycomb Corto Interacts with Methylated Ribosomal Protein L12 Via Its Chromodomain
Source: PLoS Genet. 2012 Oct 11;8(10):e1003006. doi: 10.1371/journal.pgen.1003006 (PMC3469418; doi:10.1371/journal.pgen.1003006)
Supplement: Table S13 — Sequences of the RpL12 peptides used in this study. (PDF) [file pgen.1003006.s017.pdf]

**Table S13**

| Name | Sequence |
|---|---|
| RpL12um | NH2-**PPKFDPTEVKLVYLRCVGGEVGA**-COOH |
| RpL12K3me2 | NH2-**PPK(me2)FDPTEVKLVYLRCVGGEVGA**-COOH |
| RpL12K3me3 | NH2-**PPK(me3)FDPTEVKLVYLRCVGGEVGA**-COOH |
| RpL12K10me3 | NH2-**PPKFDPTEVK(me3)LVYLRCVGGEVGA**-COOH |
| RpL12K3A | NH2-**PPAFDPTEVKLVYLRCVGGEVGA**-COOH |
