# Supplementary material for: New Partners in Regulation of Gene Expression: The Enhancer of Trithorax and Polycomb Corto Interacts with Methylated Ribosomal Protein L12 Via Its Chromodomain
Source: PLoS Genet. 2012 Oct 11;8(10):e1003006. doi: 10.1371/journal.pgen.1003006 (PMC3469418; doi:10.1371/journal.pgen.1003006)
Supplement: Table S14 — Read count data for sequencing experiments. For each edge of Paired-End sequenced samples (PE1 and PE2), the number of raw reads (raw), reads passing quality control filters (QC filter), uniq alignments (unig align) and the number of alignment used to estimate transcript abundance (transcript) are given. (PDF) [file pgen.1003006.s018.pdf]

Table S14

Sample	PE1 raw	PE1 QC filter	PE1 uniq align	PE1 transcript	PE2 raw	PE2 QC filter	PE2 uniq align	PE2 transcript
w1118	14,959,294	13,526,593	12,090,029	10,916,840	14,959,294	12,919,001	11,501,663	10,327,827
sd::Gal4/+	20,113,728	18,068,426	16,281,367	14,226,767	20,113,728	17,801,481	15,947,510	13,766,500
sd::Gal4>UAS::FH-cortoCD	27,021,927	25,744,406	19,924,474	17,536,650	27,021,927	25,501,106	19,605,738	17,237,366
sd::Gal4>UAS::RpL12-Myc	29,352,398	27,894,821	20,185,717	17,976,210	29,352,398	27,701,450	19,827,704	17,635,823
