# Supplementary material for: New Partners in Regulation of Gene Expression: The Enhancer of Trithorax and Polycomb Corto Interacts with Methylated Ribosomal Protein L12 Via Its Chromodomain
Source: PLoS Genet. 2012 Oct 11;8(10):e1003006. doi: 10.1371/journal.pgen.1003006 (PMC3469418; doi:10.1371/journal.pgen.1003006)
Supplement: Text S1 — Supporting methods. (DOC) [file pgen.1003006.s019.doc]

**Text S1. Supporting methods.**

**Cloning and site-directed mutagenesis**

*corto* and its chromodomain (CD) were amplified from clone NB67 [1]. *RpL12* was amplified from cDNAs of *w^1118^* embryonic cDNAs. Amplifications were performed with *Pfu* DNA Polymerase (Fermentas) and primers indicated in Table S12. Amplicons were cloned into *pENTR/D*, checked by sequencing, and introduced into Gateway expression vectors by LR recombination (Invitrogen). Vectors *pARW* (mRFP tag), *pAGW* (EGFP tag), *pAFHW* (FLAG and HA tags) and *pAWM* (Myc tag) were used for expression in S2 cells, and vectors *pPFHW* (FLAG and HA tags) and *pPWM* (Myc tag) were used for transgenic lines (https://dgrc.cgb. indiana.edu/vectors). *pENTR/D-cortoΔCD* was obtained by digestion of *pENTR/D-corto* with *SmaI* and *AflII* which removed a fragment from *corto* ATG to the end of the chromodomain. The open vector was ligated to a PCR fragment obtained with primers *cortoΔCD* (Table S12) that covers the region upstream of the chromodomain. *RpL12* mutagenesis was performed by PCR amplification of *pENTR/D-RpL12* using primers indicated in Table S12. After digestion of template DNA by *DpnI*, PCR products were used to transform *E. coli DH5α*. Mutated cDNA were checked by sequencing, then transferred into *pARW* by LR recombination. The *pGEX-corto* vector and its derivatives, as well as preparation and induction of GST fusion proteins, were previously described [1].

**Immunostaining of S2 cells**

S2R+ cells were fixed in 1% paraformaldehyde 10 min on ice, rinsed twice with PBS, permeabilized 15 min in 0.5% Triton and washed. They were blocked in 5% bovine serum albumin in PBS and incubated overnight with primary antibodies at a 1:50 dilution for rat Corto antibodies [1], 1:100 for rabbit anti-PH antibodies (kind gift from Dr. G. Cavalli), and 1:200 dilution for mouse anti-RNAPolII (Diagenode, AC.55-100). After washing, they were incubated with fluorescent secondary antibody at a 1:1000 dilution during 1 h (Alexa Fluor 488 goat anti-rat IgG A-11006, Alexa Fluor 594 goat anti-mouse IgG A-11032, Alexa Fluor 594 goat anti-rabbit IgG A-11037, Molecular Probes), washed, stained with DAPI and mounted in Mowiol/DABCO. Images were acquired with a Carl Zeiss Confocal SP5 miscroscope.

1. Salvaing J, Lopez A, Boivin A, Deutsch JS, Peronnet F (2003) The Drosophila Corto protein interacts with Polycomb-group proteins and the GAGA factor. Nucleic Acids Res 31: 2873-2882.
